# Mild and selective base-free C–H arylation of heteroarenes: experiment and computation†
†Electronic supplementary information (ESI) available. See DOI: 10.1039/c6sc02595a



**DOI:** 10.1039/c6sc02595a

**Published:** 2016-09-05

**Authors:** Hannes P. L. Gemoets, Indrek Kalvet, Alexander V. Nyuchev, Nico Erdmann, Volker Hessel, Franziska Schoenebeck, Timothy Noël

**Affiliations:** a Department of Chemical Engineering and Chemistry , Micro Flow Chemistry & Process Technology , Eindhoven University of Technology , Den Dolech 2 , 5612 AZ Eindhoven , The Netherlands . Email: t.noel@tue.nl; b Institute of Organic Chemistry , RWTH Aachen University , Landoltweg 1 , 52074 Aachen , Germany . Email: franziska.schoenebeck@rwth-aachen.de; c Department of Chemistry , N. I. Lobachevsky State University of Nizhny Novgorod , 23 Gagarin Avenue , 603950 Nizhny Novgorod , Russian Federation

## Abstract

A mild and selective C–H arylation strategy for indoles, benzofurans and benzothiophenes is described. The arylation method engages aryldiazonium salts as arylating reagents in equimolar amounts. The protocol is operationally simple, base free, moisture tolerant and air tolerant. It utilizes low palladium loadings (0.5 to 2.0 mol% Pd), short reaction times, green solvents (EtOAc/2-MeTHF or MeOH) and is carried out at room temperature, providing a broad substrate scope (47 examples) and excellent selectivity (C-2 arylation for indoles and benzofurans, C-3 arylation for benzothiophenes). Mechanistic experiments and DFT calculations support a Heck–Matsuda type coupling mechanism.

## Abstract 2

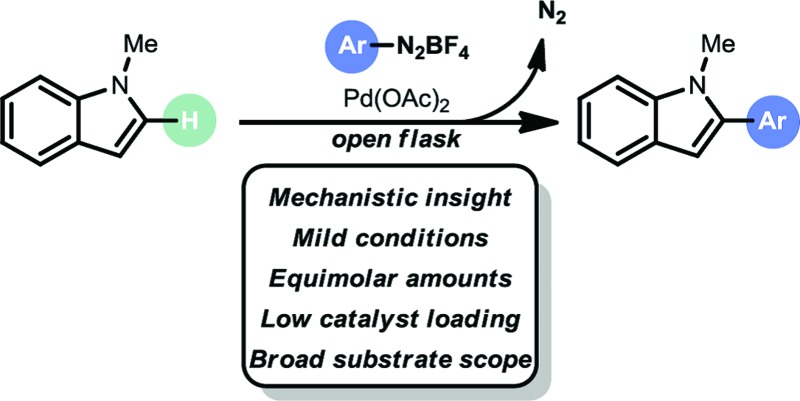
A mild and selective C–H arylation strategy for indoles, benzofurans and benzothiophenes is described.

## Introduction

The ubiquity of the heterobiaryl motif in pharmaceuticals, agrochemicals and materials illustrates its scientific and commercial value.^1^ Traditionally, these moieties have been prepared *via* cross-coupling strategies which require pre-functionalized substrates.^2^ However, over the last decade, transition metal-catalyzed C–H arylation protocols have been developed to enable the formation of C–C bonds.^3^ In contrast to classical cross-coupling chemistry, C–H arylation strategies enable direct functionalization of simple heteroarenes.

**Scheme 1:**
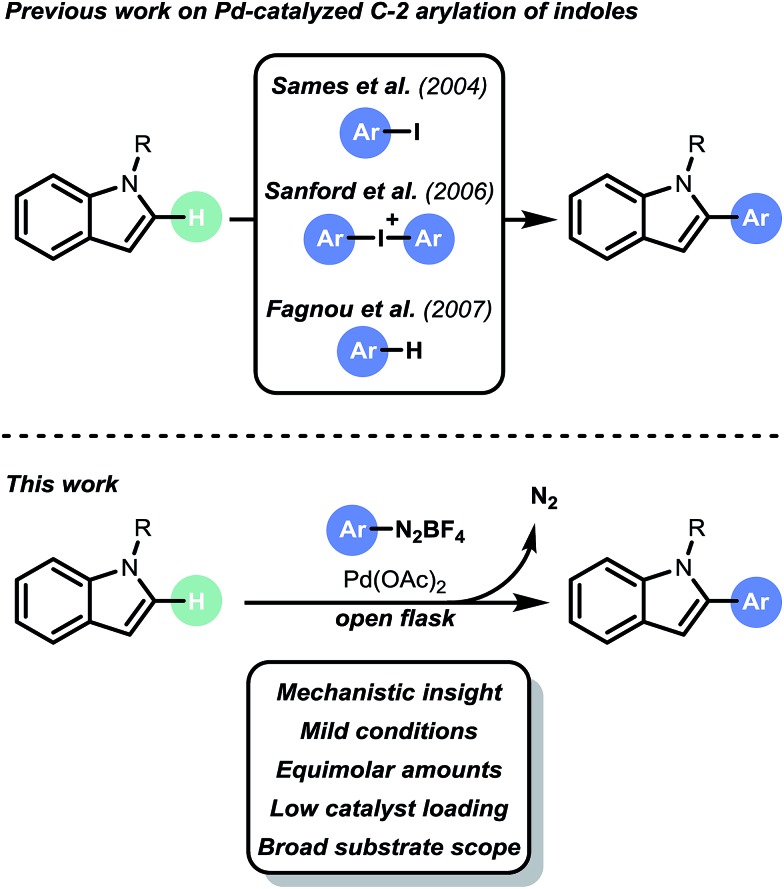
Pd-catalyzed C-2 C–H arylation of indoles.

The direct arylation of heteroarenes can be achieved *via* radical pathways, *e.g.*, visible light photoredox catalysis^4^ and Meerwein arylation.^5^ However, these methods suffer from a number of disadvantages, including long reaction times, large excesses of substrates, selectivity issues and limited substrate scopes. Recently, there has been an increase in the number of new methods, particularly in the use of metal-catalyzed processes.^6^ In particular, the work by Gaunt,^7^ Sames,^8^ Sanford,^9^ DeBoef,^10^ Glorius,^11^ Ackermann,^12^ Fagnou^13^ and Larrosa^14^ has increased the number of useful C–H arylation transformations to enable heteroaryl-(hetero)aryl bond formation. Furthermore, these examples have deepened our fundamental understanding of the underlying challenges inherent in such processes. However, the state of the art is still far from competitive with classical cross coupling strategies, *e.g.* Suzuki–Miyaura cross coupling. Current hurdles include harsh reaction conditions (*i.e.* high temperature), the necessity of stoichiometric amounts of oxidants and/or additives, use of toxic solvent systems, limited selectivity and high catalyst loadings (typically 5 to 10 mol%). Consequently, the development of new, mild and broadly applicable C–H arylation strategies is still highly desirable.^15^ We anticipated that the design of a mild and selective C–H arylation protocol for heteroaromatics (*i.e.* indoles, benzofurans and benzothiophenes) could be of high interest for API synthesis (*e.g.* Bazedoxifene,^16^ Saprisartan^17^ and Raloxifene^18^). Recently, Correia *et al.* described a Pd-based arylation of heteroarenes using aryldiazonium salts.^6*i*^ However, the protocol suffered from high catalyst loadings (10 to 20 mol% Pd), limited scope and impractical reaction conditions (*e.g.* biphasic reaction conditions, large excesses of reagents, and high reaction temperatures). Herein, we describe the development of a mild and selective palladium-based C–H arylation strategy (Scheme 1). Notable features of our open flask protocol are its operational simplicity in conjunction with low catalyst loadings, broad substrate scope, green solvent system, and short reaction times. No additional oxidants or additives are required. The strategy uses equimolar amounts or slight excesses of aryldiazonium salts as convenient arylating reagents.^6l,19^ Kinetic studies and DFT calculations suggest that a Heck–Matsuda-type mechanism occurs under our reaction conditions.

## Results and discussion

### Optimization of reaction conditions

**Fig. 1:**
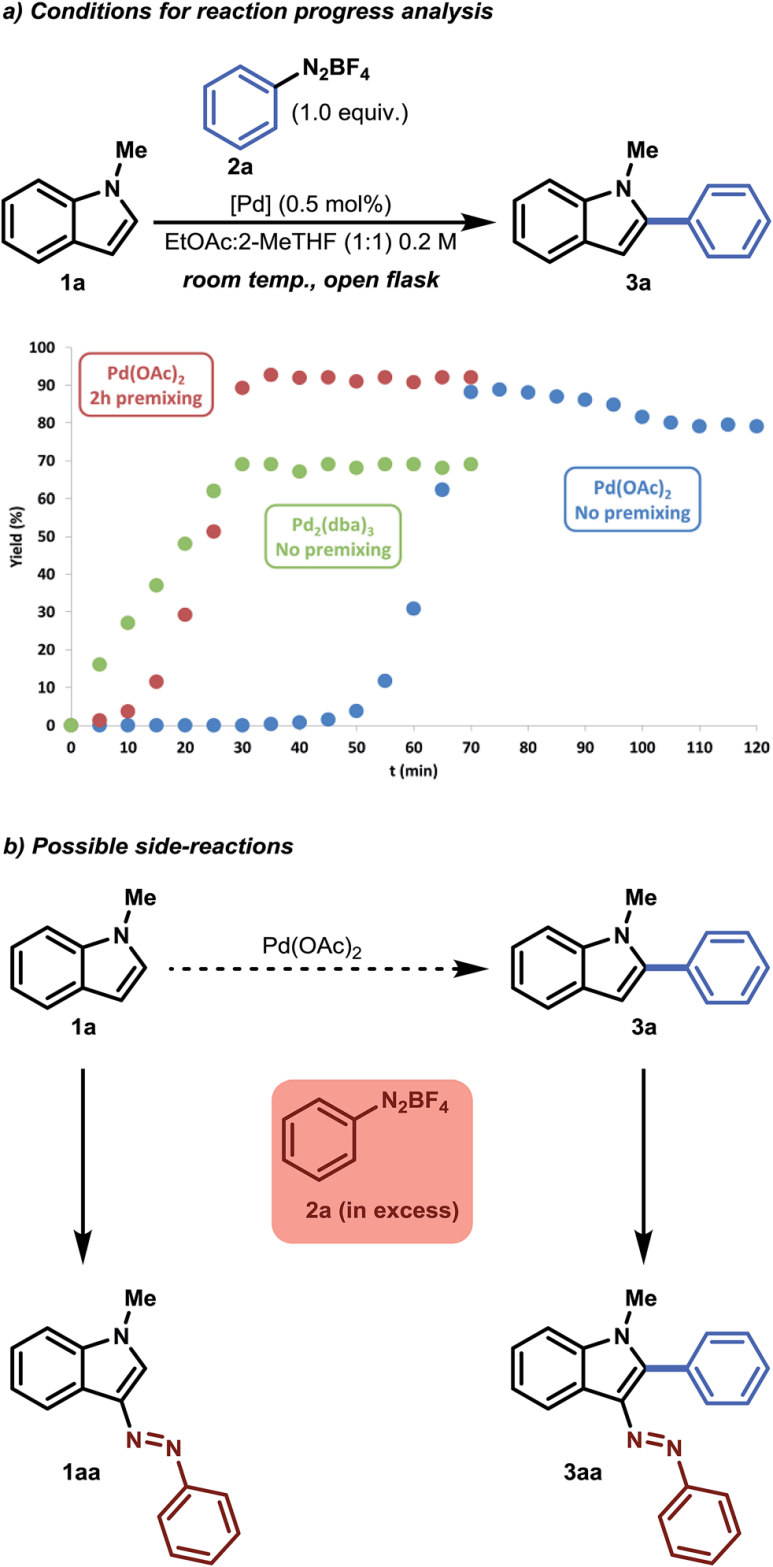
(a) Yield as a function of time. In the case of Pd(OAc)_2_ with no premixing (blue series), 1.1 equiv. of benzenediazonium tetrafluoroborate was used. (b) Observed side-reactions occurring in excess benzenediazonium tetrafluoroborate.

We commenced our optimization studies with the Pd-catalyzed C–H arylation of 1-methylindole (**1a**), which was reacted with 1.2 equivalents of benzenediazonium tetrafluoroborate (**2a**) in the presence of 10 mol% Pd(OAc)_2_ in DMF. A satisfying yield of 66% for 1-methyl-2-phenylindole (**3a**) was obtained within only 30 minutes of reaction time at room temperature (Table 1, Entry 1). The main byproducts were 3-(arylazo)-1-methylindole (**1aa**) and 2-aryl-3-(arylazo)-1-methylindole (**3aa**), due to the uncatalyzed electrophilic substitution reaction between the highly electrophilic nitrogen of the aryldiazonium salt and the C-3 position of 1-methylindole (Fig. 1b). Lowering the catalyst loading to 5 mol% Pd(OAc)_2_ in DMF resulted in a significant drop in yield (34%). An overnight control experiment showed that no product formation was observed in the absence of a palladium catalyst (Table 1, Entry 3). Using polar protic solvents (*e.g.* i-PrOH) resulted in generally high reactivity and moderate yields. A considerable amount of byproduct formation was consistently observed (see ESI†). It was generally found that carrying out the reaction in less polar (aprotic) solvents (*e.g.* DMF → THF → 1,4-dioxane) resulted in reduced byproduct formation. After solvent screening, THF was considered to be the best solvent (77%), combining both the desired reactivity and selectivity (Table 1, Entry 5). A control experiment using Schlenk techniques indicated that the catalyst was not affected by air and moisture (Table 1, Entry 5). Therefore, all the following experiments could be performed as open-flask reactions, making this procedure appealing for future scale-up. A catalyst survey demonstrated that only Pd(OAc)_2_, Pd(TFA)_2_ and, to a lesser extent, Pd_2_(dba)_3_ were active catalysts for this chemical transformation (Table 1, Entries 6–8). The use of Pd(OAc)_2_ was preferred over Pd(TFA)_2_ due to its cost efficiency and stability. Further optimization studies showed that it was possible to lower the catalyst loading further to 0.5 mol% in THF (Table 1, Entry 9). Even lower catalyst loading resulted in only trace amounts of product (Table 1, Entry 10). Finally, 2-MeTHF was evaluated. This solvent is recognized as a green solvent for synthetic organic chemistry because it can be readily produced from furfural, a common biomass material.^20^ Satisfyingly, 2-MeTHF showed even better selectivity for the desired product (87%), although an increased reaction time of 2 hours was required to obtain full conversion (Table 1, Entry 11). Continued optimization studies with green solvents revealed that the reaction time could be halved by using EtOAc : 2-MeTHF (1 : 1) as a solvent mixture (Table 1, Entry 12). Indeed, this solvent combination proved to be superior, as it enabled further lowering of the catalyst loading to 0.2 mol% Pd(OAc)_2_ (Table 1, Entry 10 *vs.* 13). However, in the case of 0.2 mol% Pd(OAc)_2_, significant increases of **1aa** and **3aa** were observed because the reactivity toward the desired arylation was diminished. Therefore, 0.5 mol% Pd(OAc)_2_ was considered to be optimal.

**Table 1:** Optimization for the Pd-catalyzed C-2 arylation of 1-methylindole^*a*^

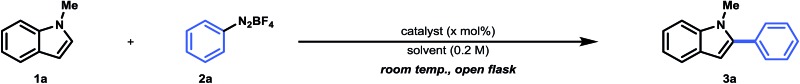				
Entry	Catalyst (mol%)	Solvent	Reaction time	Yield GC-FID (%)
1	Pd(OAc)_2_ (10.0)	DMF	30 min	66
2	Pd(OAc)_2_ (5.0)	DMF	30 min	34
3	—	DMF	16 h	0
4	Pd(OAc)_2_ (5.0)	Solvent^*b*^	30 min	<72
5	Pd(OAc)_2_ (5.0)	THF	30 min	77; 76^*c*^
6	Catalyst (5.0)^*d*^	THF	2 h	0
7	Pd(TFA)_2_ (5.0)	THF	30 min	76
8	Pd_2_(dba)_3_ (2.5)	THF	30 min	68
9	Pd(OAc)_2_ (0.5)	THF	1 h	81
10	Pd(OAc)_2_ (0.2)	THF	1 h	Trace
11	Pd(OAc)_2_ (0.5)	2-MeTHF	2 h	87
12	Pd(OAc)_2_ (0.5)	EtOAc : 2-MeTHF (1 : 1)	1 h	89
13	Pd(OAc)_2_ (0.2)	EtOAc : 2-MeTHF (1 : 1)	1 h	78
14^*e*^	Pd(OAc)_2_ (0.5)	EtOAc : 2-MeTHF (1 : 1)	30 min	**93; 90** ^*f*^

^*a*^Reaction conditions: catalyst, 0.5 mmol heteroarene and 1.2 equiv. benzenediazonium tetrafluoroborate in 2.5 mL solvent at rt, 0.1 equiv. decafluorobiphenyl as internal standard for GC-FID, open flask.

^*b*^Solvent: H_2_O, AcOH, EtOAc, propylene carbonate, DMF, acetone, MeCN, Et_2_O, 1,4-dioxane, MeOH, EtOH, i-PrOH *n*-BuOH, DCM, DCE, CHCl_3_, toluene.

^*c*^Schlenk line techniques used.

^*d*^Catalyst: 10% Pd/C, PdCl_2_, Cu(OAc)_2_, Cu(OTf)_2_, Pd[P(C_6_H_5_)_3_]_4_, (MeCN)_2_Pd(ii)Cl_2_ and (η^3^–C_3_H_5_)_2_Pd_2_Cl_2_, PEPPSI-SIPr.

^*e*^2 h premixing of Pd(OAc)_2_ with 1-methylindole, 1.0 equiv. of benzenediazonium tetrafluoroborate used.

^*f*^isolated yield.

In parallel with our optimization studies, a series of reaction progress kinetic experiments were performed to shed more light on the observed catalyst induction period. Unusual kinetics has often been reported in the field of C–H functionalization, but has seldom been investigated.^21^ Therefore, in order to obtain a more realistic view of this activation period, we monitored a series of reactions. As can be seen from Fig. 1a, an induction period of approximately 50 minutes was observed in the case of Pd(OAc)_2_ (Fig. 1a, blue series). As soon as the reaction began (>50 min), an initial acceleration occurred, resulting in S-curve behavior. It was postulated that a possible activation period could be necessary between the catalyst and the substrate. Therefore, premixing experiments were conducted. It was found that premixing 1-methylindole with Pd(OAc)_2_ (0.5 mol%) in EtOAc : 2-MeTHF (1 : 1) for 2 hours could eliminate this observed induction period (Fig. 1a, red series). We surmised that Pd(ii) is first reduced to a homogeneous Pd(0) complex and is stabilized by the π-donating character of 1-methylindole and/or by the ligand exchange of ^–^OAc with 2-MeTHF.^22^ Indeed, a reaction performed with Pd_2_(dba)_3_ as a stable homogenous Pd(0) substitute showed that neither an induction period nor an initial acceleration occurred (Fig. 1a, green series). However, lower yields were obtained with Pd_2_(dba)_3_. This result gives us a first glimpse of the possible catalytic mechanism, indicating that palladium in its homogeneous zero state can act as an active catalyst.

As expected, the product **3a** was even more prone to undergo a side reaction (*i.e.* an electrophilic substitution reaction) with benzenediazonium salt, as the inductive effect of the phenyl substituent makes the C-3 position more nucleophilic.^23^ This was especially noticeable when a slight excess of benzenediazonium tetrafluoroborate was used (Fig. 1a, blue series). A small yield of approximately 10% was observed after prolonged reaction time, which accounts for the 0.1 equivalent excess. To counteract this consecutive reaction, an equimolar amount (1.0 equiv. benzenediazonium tetrafluoroborate) was used. As a result, 90% of the desired product could be isolated (Table 1, Entry 14). Note that the reaction time could be halved again, to approximately 30 minutes, when using the premixing strategy. In addition, a slightly higher selectivity was obtained because side reactions were minimized. More information regarding reaction optimization and reaction progress analysis can be obtained from the ESI.†

### Mechanistic studies: DFT calculations and experimental investigations

Since heteroarenes are good nucleophiles, it would be reasonable to assume a mechanism in which Pd(ii) acts as an electrophile, consistent with numerous literature proposals in the context of C–H functionalization.^8a,24^ Similar to S_E_Ar, these reactions are expected to be C-3 selective for indoles. However, our methodology yields C-2 arylated indoles selectively and thus requires a subsequent C-3/C-2 isomerization. In this context, Gaunt and co-workers showed that the presence of acid would facilitate a switch from C-3 to C-2 in the Pd-catalyzed C–H olefination of indoles,^24^ proposing that under acidic conditions, C-3 deprotonation of the indole moiety would be slowed. However, such a scenario appears unlikely in our case. For example, progressive ^1^H-NMR spectroscopy with equimolar quantities of Pd(OAc)_2_ and 1-methylindole in d_8_-THF showed that neither the H_C-2_ or the H_C-3_ peaks of 1-methylindole were affected (see ESI Section 3.1.1†).^25^

**Scheme 2:**
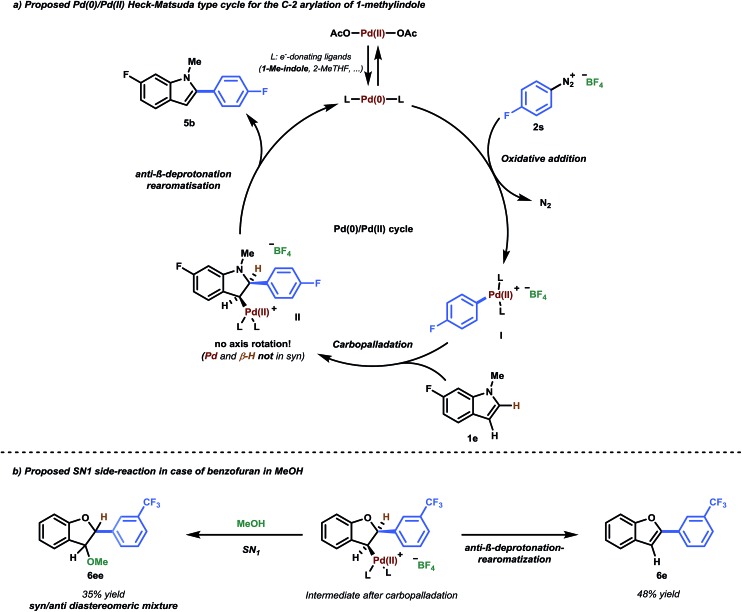
(a) Proposed Pd(0)/Pd(ii) Heck–Matsuda-type cycle for the C-2 arylation of 1-methylindole. (b) Observed SN_1_ side-reaction in the case of benzofuran in MeOH.

**Fig. 2:**
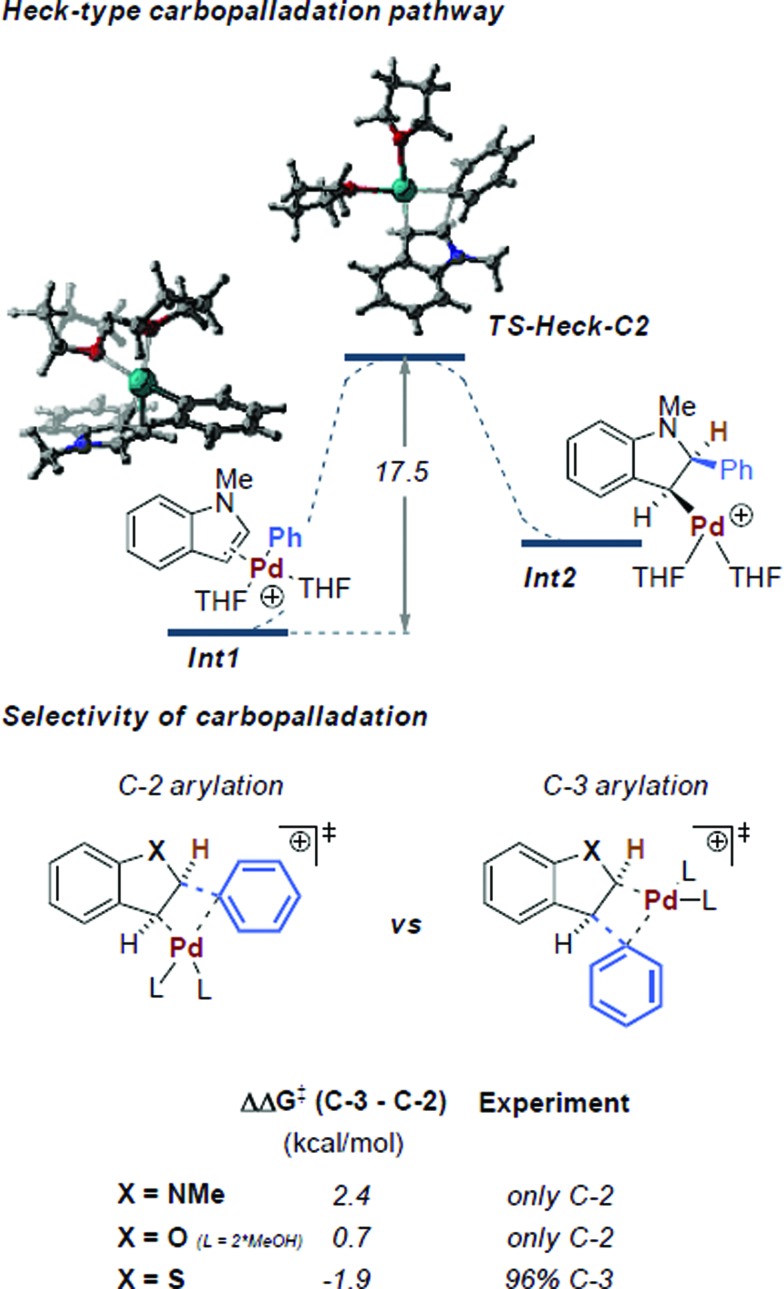
Heck-type carbopalladation pathway and the prediction of selectivity *via* its transition states at the CPCM (THF) M06L/def2TZVP//wB97X-D/6-31G(d) SDD level of theory.^31^ Coordination by two THF molecules was found to be the preferred ligation state of Pd.^29^ Free energies are shown in kcal mol^–1^.

Therefore, the employed Pd(OAc)_2_ likely serves as a pre-catalyst and is reduced to Pd(0) during the initiation period. Additionally, since we have shown that Pd(0) is catalytically active without any induction period (Table 1, Entry 8), it is reasonable to assume that the reaction proceeds *via* a Pd(0)/Pd(ii) catalytic cycle.^26^ This cycle starts with an initial oxidative addition of the highly activated aryl diazonium salt to Pd(0) to yield a cationic Pd(ii) complex which should subsequently serve as an electrophile in the reaction with the substrate (Scheme 2, **I**). The overall product selectivity would then again be determined by the C-3 to C-2 migration of Pd.^24^ However, our efforts to computationally locate the C-2 Pd complex yielded a structure that is 9.1 kcal mol^–1^ higher in energy than the preferred η^2^ π-complex **Int1** (Fig. 2), suggesting that the migration is disfavored.^27^

Intermediate **Int1** may alternatively undergo a Heck-type carbopalladation reaction.^28^ Our calculations suggest this process to be energetically feasible, being characterized by a relatively facile free energy barrier of 17.5 kcal mol^–1^ (Fig. 2). Thus, we subsequently calculated the expected selectivities (C-3 *versus* C-2) for C–H arylation for a carbopalladation mechanism. We considered several possible solvent coordinations to the cationic Pd; we determined that the coordination of two THF molecules is likely preferred.^29^ Our computed selectivities are in agreement with experiments. Complete C-2 selectivity was experimentally observed for 1-methylindole and benzofuran, consistent with our computational results (ΔΔ*G* ^‡^ = 2.4 kcal mol^–1^ and 0.7 kcal mol^–1^ in favor of C-2, respectively).^30^ By contrast, benzothiophene yielded the C-3 arylated product exclusively, which was also reproduced by computations (ΔΔ*G* ^‡^ = 1.9 kcal mol^–1^ in favor of C-3) (see Fig. 2).

The carbopalladation step in the traditional Heck-type reaction would be followed by *syn*-β-hydride elimination. Due to the rigidity of the ring system, however, there is no possibility of conventional *syn*-β-hydride elimination from the formed intermediate **Int2**. In contrast, it has been previously suggested that a base or solvent assisted anti-β-deprotonation rearomatisation could occur.^14a,28b,32^ While that step may also be involved in our case, due to the ionic and complex natures of the intermediates involved, an adequate computational description of the system would pose a number of difficulties.^28b,33^ However, *in situ* ^1^H and ^19^F NMR analysis of the reaction have given us initial insights into the likely nature of the processes involved (see ESI Section 3.1.2 for a detailed description†). The data indicate that additional signals, assigned as BF_3_·2Me–THF and HF, appear in the ^19^F NMR spectrum at the same rate as the product **5b**. Moreover, when using an alternative counterion for the aryldiazonium salt (*e.g.*, 4-methoxybenzenediazonium mesylate), no product was observed (Table 2, **3h**). It is therefore hypothesized that the BF_4_^–^ counterion of the aryldiazonium salt plays a non-negligible role in the reaction mechanism, *i.e.* acting as a pseudo-base in the anti-β-deprotonation rearomatisation step. In addition, a crude ^1^H-NMR spectrum acquired from the reaction mixture (using THF-d_8_ as solvent) indicates that the lost proton appears quantitatively as a broad signal at 9.0 ppm (See ESI Section 2.3).

**Table 2:** Scope for the C-2 arylation of indoles^*a*^

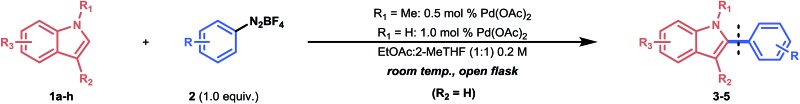
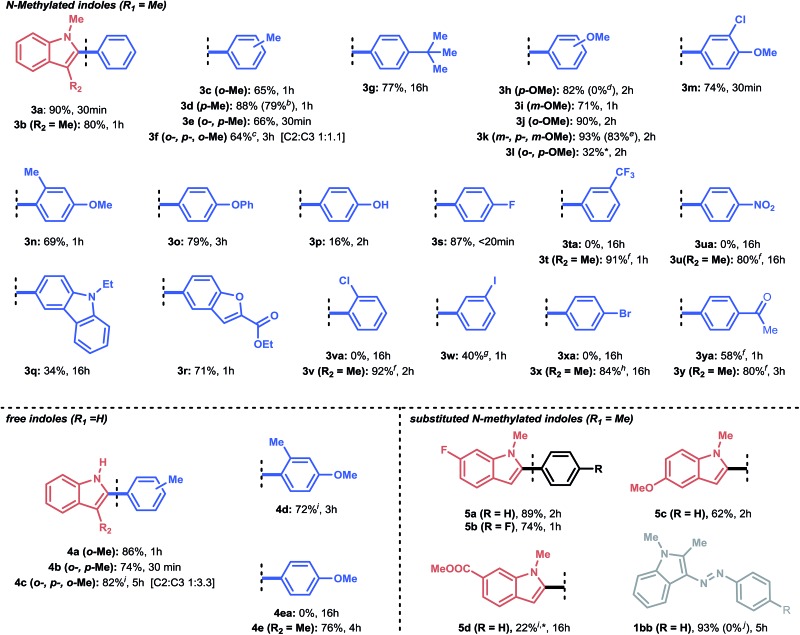

^*a*^Reaction conditions: 0.5 to 1.0 mol% Pd(OAc)_2_, 1.0 mmol heteroarene and 1.0 equiv. aryldiazonium salt in 5 mL EtOAc : 2-MeTHF (1 : 1) at rt, open flask, 2 h premixing of Pd(OAc)_2_ with heteroarene.

^*b*^Pd_2_(dba)_3_ as catalyst, 1 h reaction.

^*c*^1 mol% Pd(OAc)_2_, 1.2 equiv. aryldiazonium salt.

^*d*^4-Methoxybenzenediazonium mesylate was used.

^*e*^Gram-scale experiment (10.0 mmol) yielded 2.47 g (83%), 4 h reaction time in 2-MeTHF as solvent.

^*f*^1 mol% Pd(OAc)_2_.

^*g*^2 mol% Pd(OAc)_2_.

^*h*^2 mol% Pd(OAc)_2_, 1.2 equiv. aryldiazonium salt.

^*i*^1.2 equiv. aryldiazonium salt at 40 °C; *no full conversion obtained.

^*j*^0.01 M and 100 mol% Pd_2_(dba)_3_ was used.

Alternatively, a radical mechanism could be envisioned for this transformation. However, a large excess (5 to 100 equiv.) of the heteroarene substrate is generally required for satisfying results under such conditions. In our case, optimal results were achieved with equimolar quantities. In addition, test reactions *via* the radical pathway^34^ did not lead to the desired product. Moreover, radical scavenging tests failed to trap any radical intermediates (See ESI Section 3.2 for details). Finally, in radical chemistry, mixtures of C-2 and C-3 arylation are frequently observed,^35^ while our system displays complete selectivity.

### Synthetic scope: heteroarenes and aryldiazonium salts

With the optimized conditions in hand, we next explored the scope of our developed methodology on indoles (Table 2). These substrates were reacted with equimolar amounts of aryldiazonium salts in the presence of 0.5 mol% Pd(OAc)_2_ in the case of 1-methylindoles and 1.0 mol% of Pd(OAc)_2_ for NH-indoles. A 1 : 1 mixture of EtOAc : 2-MeTHF was used as the solvent. A broad set of substituted aryldiazonium substrates (**3a–y**) could be successfully coupled with 1-methylindole. Indole arylation with aryldiazonium salts bearing alkyl substituents (**3c–g**, **4a**, **b**) proceeded well for both N-protected and free indoles, even in the presence of sterically demanding *ortho*-methyl substituents (**3c**, **3e**). When using the more sterically hindered mesitylenediazonium tetrafluoroborate as the arylating agent, a mixture (C-2 and C-3 arylated product) was found for both the *N*-methylated and the free indoles (**3f**, **4c**). Selectivity towards the C-3 arylated product was prevalent in **4c** (C-2 : C-3 1 : 3.3). For all other reactions, complete selectivity towards the C-2 arylated product was observed. Next, a scope of aryldiazonium salts, containing hydroxy-, phenoxy- and methoxy-substituents, was explored (**3h–p**). It was demonstrated that aryldiazonium salts bearing a free hydroxyl group showed some reactivity, although in lower yield (**3p**, 16%). A *para*-phenoxy group as an electron-donating substituent on the aryldiazonium salt resulted in good reactivity (**3o**, 79%).

Moreover, all methoxy-containing aryldiazonium salts (**3h–n**) showed good to excellent reactivity (69% to 93%), except for **3l**, where no full conversion could be obtained. The yields obtained for compounds **3h–n** showcase the applicability of our methodology for the C-2 arylation of indoles with arylating agents bearing methoxy-substituents, which are often reported to be cumbersome.^7,8b,9b^ These substituents are functional handles which can be engaged in nickel-catalyzed cross-coupling chemistry *via* C–O activation.^36^ In addition, heterocyclic aryldiazonium salts were tolerated in this protocol: **3q** was obtained in moderate yield (34%) overnight, while for **3r**, a good yield (71%) was acquired within 1 hour reaction time. Notably, in the case of free NH-indoles (**4a–d**), an *ortho*-methyl substituent on the aryldiazonium salt proved necessary to avoid significant by-product formation (electrophilic substitution). However, it was found that by blocking the C-3 position of the NH-indole (*i.e.*, *via* methylation), this side-reaction could be completely avoided (**4ea** *vs.* **4e**).

Next, we explored a more challenging class of aryldiazonium salts bearing weakly (*e.g.*, F) to highly electron-withdrawing (*e.g.*, NO_2_) substituents (**3s–y**). Gratifyingly, 4-fluoro- and 3-iodobenzenediazonium tetrafluoroborate readily reacted with 1-methylindole (**3s**, **3w**). The latter (**3w**) is particularly appealing, since it indicates that palladium undergoes oxidative addition at the electrophilic diazonium site (instead of breaking the C–I bond) at room temperature. In contrast, aryldiazonium salts bearing *m*-CF_3_ (**3ta**), *p*-NO_2_ (**3ua**), *o*-Cl (**3va**) and *p*-Br (**3xa**) as substituents did not deliver any arylated product when 1-methylindole was used as the substrate. It was observed that these aryldiazonium salts were too prone to electrophilic substitution reactions, resulting in the rapid formation of 3-(arylazo)-1-methylindoles (see Fig. 1b). However, as in the NH-indole case, this side reaction could be efficiently overcome by blocking the C-3 position. Consequently, the arylation scope could be expanded to electron-withdrawing substituents (**3t**, **3u**, **3v**, **3x**) with high to excellent yields of the desired product (80% to 92%). This trend was also observed when aryldiazonium salts bearing an acyl moiety were used (**3ya** and **3y**): 58% of the target product (**3ya**) was obtained for 1-methylindole, while an improved result was obtained for the C-3 methylated indole (80% yield, **3y**).

Subsequently, several indole derivatives were subjected to the reaction conditions using benzenediazonium tetrafluoroborate as a benchmark coupling partner. For **5a** and **5c**, the reaction proceeded smoothly under equimolar conditions. **5d** proved more challenging (22% yield) due to the electron-withdrawing nature of the methyl carboxylate substituent, which renders it a less nucleophilic substrate. Interestingly, an experiment with 1,2-dimethylindole and benzenediazonium salt showed that no C-3 arylated product could be formed over 5 hours. Instead, the substrate was fully converted to the electrophilic substituted product **1bb** (93% yield). Moreover, during a control experiment with a stoichiometric amount of Pd_2_(dba)_3_, no **1bb** was formed. This indicates that the benzenediazonium salt preferably underwent oxidative addition (see ESI Section 3.4†).

Next, a gram scale experiment was conducted to test the scalability of this mild procedure. The reaction was carried out with equimolar quantities of reactants (10 mmol) and 0.5 mol% Pd(OAc)_2_ in 2-MeTHF. With a slightly longer reaction time of 4 hours, a satisfying yield of 83% (2.47 g) of **3k** was achieved under open flask conditions.

Having established a good coupling protocol for indoles, we subsequently examined the scope of benzofuran (**1i**) with various aryldiazonium salts (Table 3). Since benzofuran is not prone to electrophilic substitution, MeOH could be used as a more reactive solvent (see ESI Section 2.4†). These results are in agreement with the literature.^19*b*^ Felpin *et al.* demonstrated with DFT and experimental results that the cationic palladium intermediates in the Heck cycle are exoergic with MeOH as the solvent.^37^ Moreover, it was observed that the addition of 1.0 equivalent of TFA resulted in an impressive rate acceleration (overnight to 30 minutes) while maintaining its selectivity (**6e**, 81%). The use of the protic solvent MeOH resulted in the formation of 2-aryl-3-methoxy-2,3-dihydrobenzofuran (**6ee**). It was speculated that **6ee** was formed from the proposed carbopalladation intermediate **II** through a SN_1_ mechanism, resulting in the observed *syn*/*anti* diastereomeric mixture (Scheme 2b). However, a simple workup procedure consisting of 15 minutes of reflux under acidic conditions (*i.e.* acetyl chloride) was found to be sufficient to eliminate MeOH from the compound, affording the desired product in overall high yield. Benzofuran could be readily coupled with benzenediazonium tetrafluoroborate in good yield (**6a**, 70%).

**Table 3:** Scope of the reaction of aryldiazonium tetrafluoroborate with benzofuran and benzothiophene^*a*^

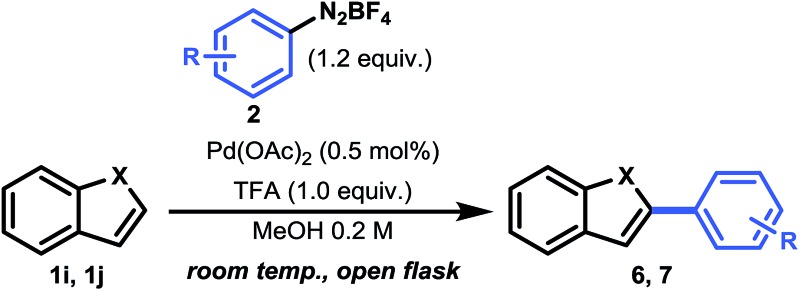
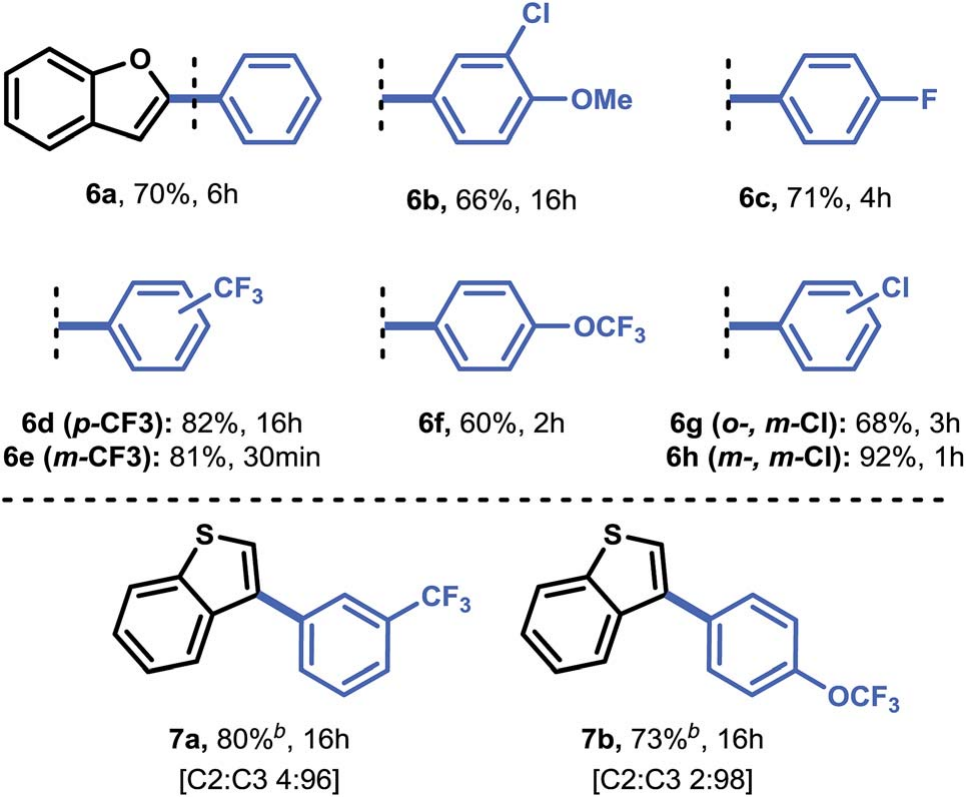

^*a*^Reaction conditions: 0.5 mol% Pd(OAc)_2_, 1.0 mmol heteroarene and 1.2 equiv. aryldiazonium tetrafluoroborate in 5 mL MeOH at rt, open flask, after full conversion: reflux with 5.0 equiv. acetyl chloride for 15 min.

^*b*^2.0 mol% Pd(OAc)_2_, 2.0 equiv. aryldiazonium tetrafluoroborate at 40 °C.

Next, we carried out several reactions by coupling benzofuran with several halogenated aryldiazonium tetrafluoroborates (**6b–h**). Satisfyingly, all reactions proceeded smoothly in the presence of only 0.5 mol% Pd(OAc)_2_, thus showcasing the mild reaction conditions of this protocol.

Finally, we turned our attention towards a more challenging heteroarene, *i.e.* benzothiophene (**1j**) (Table 3). Because benzothiophene is the least nucleophilic heteroarene investigated herein, it was necessary to use slightly higher catalyst loadings (2.0 mol%) and 2.0 equivalents of aryldiazonium salt in order to achieve full conversion. Operating at 40 °C was decisive to obtain a good yield for both **7a** (80%) and **7b** (73%). In agreement with literature reports and DFT calculations (see above), we observed a complete shift in selectivity from C-2 to C-3 arylation. For compound **7a**, a significant improvement in yield (80% *vs.* 69%) and a reduction in reaction time (16 h *vs.* 96 h) was observed, highlighting the relevance of our mild protocol.^11*b*^ Taken together, this C–H activation protocol for the direct arylation of heteroarenes provides a convenient pathway towards a broad range of heteroaromatic arylated derivatives.

### Application: synthesis of the drug precursor of Saprisartan

**Scheme 3:**
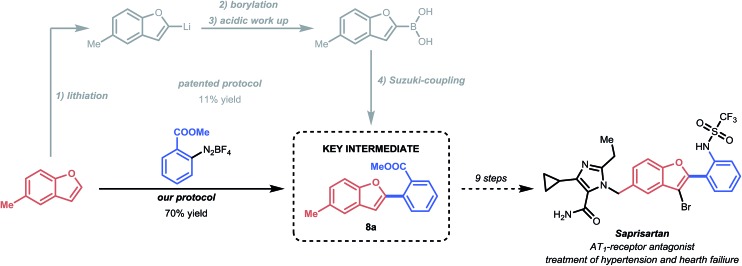
Synthesis of the drug precursor **8a** of Saprisartan.

To further illustrate the efficacy of this mild strategy, we applied the C–H arylation process to the synthesis of methyl 2-(5-methylbenzofuran-2-yl)benzoate (**8a**) (Scheme 3). Compound **8a** is a key intermediate in the total synthesis of Saprisartan, an approved drug belonging to the sartan family.^17^ Sartans act as AT_1_-antagonists and are among the most prescribed drugs for the treatment of hypertension and heart failure.^38^ Our mild procedure allowed us to successfully isolate the key intermediate **8a** in 70% yield, which compares favorably to the patented process, which requires four consecutive steps for the same transformation with an overall low yield of 11%.^17,39^

## Conclusion

In summary, we have developed a mild and selective protocol for the C–H arylation of heteroarenes, including indoles, benzofurans and benzothiophenes, with aryldiazonium salts. The protocol is operationally simple and is insensitive to air and moisture. It utilizes low palladium loadings (0.5 to 2 mol% Pd), short reaction times, green solvents (EtOAc/2-MeTHF or MeOH) and is carried out at room temperature. Notably, no oxidants or other additives are required. The substrate scope is broad and displays excellent selectivity (C-2 arylation for indoles and benzofurans, C-3 arylation for benzothiophenes). To illustrate the efficacy of this procedure, a key intermediate (**8a**) for the drug Saprisartan was synthesized, comparing favorably to the patented process (70% *vs.* 11%). Mechanistic experiments and DFT calculations support a Heck–Matsuda-type coupling mechanism. Moreover, the first experimental results indicate that BF_4_^–^ anions could be involved in the anti-β-deprotonation rearomatisation step of the catalytic cycle. We expect this protocol will find widespread application due to its mild character and excellent selectivity.
