# Supplementary material for: Mild and selective base-free C–H arylation of heteroarenes: experiment and computation
Source: Chem Sci. 2016 Sep 5;8(2):1046–55. doi: 10.1039/c6sc02595a (PMC5356503; doi:10.1039/c6sc02595a)
Supplement: Supplementary file 1 [file SC-008-C6SC02595A-s001.pdf]

Electronic Supplementary Material (ESI) for Chemical Science.
This journal is © The Royal Society of Chemistry 2016

Electronic Supporting Information (ESI)

Mild and selective base-free C–H arylation of heteroarenes: Experiment and computation.

Hannes P. L. Gemoets,[a] Indrek Kalvet,[b] Alexander V. Nyuchev,[a,c] Nico Erdmann,[a] Volker Hessel,[a] Franziska Schoenebeck,[b]* Timothy Noel[a]*

[a]Department of Chemical Engineering and Chemistry, Micro Flow Chemistry & Process Technology, Eindhoven University of Technology, Den Dolech 2, 5612 AZ Eindhoven, The Netherlands.

[b]Institute of Organic Chemistry, RWTH Aachen University, Landoltweg 1, 52074 Aachen, Germany.

[c]Department of Chemistry, N. I. Lobachevsky State University of Nizhny Novgorod, 23 Gagarin Avenue, 603950 Nizhny Novgorod, Russian Federation.

E-mail: t.noel@tue.nl for Timothy Noël

E-mail: franziska.schoenebeck@rwth-aachen.de for Franziska Schoeneboeck

Table of Contents

1. General information 3
2. Optimization of reaction conditions. 4
   2.1 Optimization for the Pd-catalyzed C–H arylation of 1-methylindole 4
   2.2 Reaction progress analysis 5
   2.3 ^{1}H-NMR of the crude reaction mixture 7
   2.4 Optimization for the Pd-catalyzed C–H arylation of benzofuran 8
3. Mechanistic Studies: DFT calculations and experimental investigations. 9
   3.1 Progress NMR experiments 9
      3.1.1 Progress ^{1}H-NMR of mixing stoichiometric $Pd(OAc)_2$ and 1-methylindole in d_8-THF 9
      3.1.2 Progress ^{19}F-NMR of the reaction 6-fluoro-1-methylindole with 4-fluorobenzenediazonium tetrafluoroborate in EtOAc: 2-MeTHF (1:1) 10
   3.2 Radical scavenging experiments 14
   3.3 Control experiments regarding the non-innocent role of BF_4^- 15
   3.4 Control experiments regarding the oxidative addition step 16
   3.5 DFT calculations: Computational methods 18
4. General procedure for the synthesis of aryldiazonium tetrafluoroborates 19
5. General procedure for the Pd-catalyzed C–H arylation of heteroarenes 21
   5.1 General procedure for the C–H arylation of 1-methylindole and derivatives (condition A) 21
   5.2 General procedure for the C–H arylation of NH-indole (conditions B) 21
   5.3 General procedure for the C–H arylation of benzofuran (condition C) 22
   5.4 General procedure for the C–H arylation of benzothiophene (condition D) 22
   5.5 Procedure for the gram scale C–H arylation of 1-methylindole 23
6. Procedure for synthesis of Saprisartan precursor 24
   6.1 Synthesis of 5-methylbenzofuran substrate 24
   6.2 Synthesis of Saprisartan precursor (8a) 24
7. Characterization data for the Pd-catalyzed C–H arylated heteroarenes 26
8. References 45
9. DFT calculations: XYZ and thermal Data 46
10. NMR spectra 59

1. General information

All components as well as reagents and solvents were used as received without further purification, unless stated otherwise. Reagents and solvents were bought from Sigma Aldrich and TCI and if applicable, kept under argon atmosphere. Technical solvents were bought from VWR International and used as received. Product isolation was performed using silica (60, F254, Merck™), and TLC analysis was performed using Silica on aluminum foils TLC plates (F254, Supelco Sigma-Aldrich™) with visualization under ultraviolet light (254 nm and 365 nm) or appropriate TLC staining. ^{1}H (400MHz or 500 MHz), ^{13}C (100MHz) and ^{19}F (376 MHz) NMR spectra were recorded on ambient temperature using a Bruker-Avance 400 or Mercury 400. ^{1}H NMR spectra are reported in parts per million (ppm) downfield relative to $CDCl_3$ (7.26 ppm) and all ^{13}C NMR spectra are reported in ppm relative to $CDCl_3$ (77.2 ppm). NMR spectra uses the following abbreviations to describe the multiplicity: s = singlet, d = doublet, t = triplet, q = quartet, p = pentet, h = hextet, hept = heptet, m = multiplet, dd = double doublet, td = triple doublet. Known products were characterized by comparing to the corresponding ^{1}H NMR and ^{13}C NMR from literature. GC analyses were performed on a GC-FID (Varian 430-GC) in combination with an auto sampler (Varian CP-8400) or GC-MS combination (Shimadzu GC-2010 Plus coupled to a Mass Spectrometer; Shimadzu GCMS-QP 2010 Ultra) with an auto sampler unit (AOC-20i, Shimadzu). Melting points were determined with a Buchi B-540 capillary melting point apparatus in open capillaries and are uncorrected. Infrared spectra were recorded on a Shimadzu IR Affinity-1 Fourier Transform Infrared Miracle 10 Single Reflection ATR Spectrometer.

2. Optimization of reaction conditions.

2.1 Optimization for the Pd-catalyzed C–H arylation of 1-methylindole

Table S 1: Optimization table for Pd-catalyzed C–H arylation of 1-methylindole

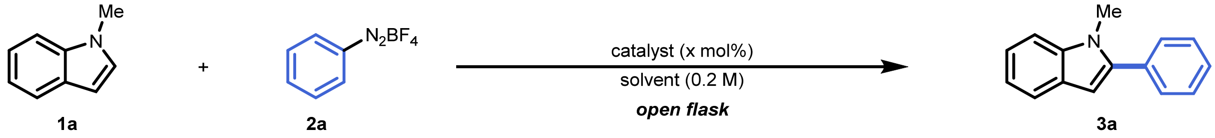

Entry	Catalyst (mol%)	Solvent	Reaction time	Yield GC-FID (%)
1	$Pd(OAc)_2$ (10.0), $Ru(bpy)_3Cl_2$ (2.5)	DMF	30 min	62[b];65[c]
2	$Ru(bpy)_3Cl_2$ (2.5)	DMF	2 h	0[b]
3	$Pd(OAc)_2$ (5.0)	H2O	30 min	trace
4	$Pd(OAc)_2$ (5.0)	AcOH	30 min	22
5	$Pd(OAc)_2$ (5.0)	MeOH	30 min	57
6	$Pd(OAc)_2$ (5.0)	EtOH	30 min	69
7	$Pd(OAc)_2$ (5.0)	iPrOH	30 min	72
8	$Pd(OAc)_2$ (5.0)	n-BuOH	30 min	38
9	$Pd(OAc)_2$ (5.0)	PC	30 min	35
10	$Pd(OAc)_2$ (5.0)	DMF	30 min	34
11	$Pd(OAc)_2$ (5.0)	EtOAc	30 min	22
12	$Pd(OAc)_2$ (5.0)	DCM	30 min	28
13	$Pd(OAc)_2$ (5.0)	DCE	30 min	27
14	$Pd(OAc)_2$ (5.0)	CHCl3	30 min	16
15	$Pd(OAc)_2$ (5.0)	1,4-dioxane	30 min	26
16	$Pd(OAc)_2$ (5.0)	solvent[d]	30 min	0
17	$Pd(OAc)_2$ (0.2)	EtOAc:2-MeTHF (1:0)	1 h	17
18	$Pd(OAc)_2$ (0.2)	EtOAc:2-MeTHF (3:1)	1 h	10
19	$Pd(OAc)_2$ (0.2)	EtOAc:2-MeTHF (1:3)	1 h	trace
20	$Pd(OAc)_2$ (0.2)	EtOAc:2-MeTHF (0:1)	1 h	trace
21[e]	$Pd(OAc)_2$ (0.5)	EtOAc:2-MeTHF (1:1)	1 h	trace
22[f]	$Pd(OAc)_2$ (0.5)	EtOAc:2-MeTHF (1:1)	1 h	72
23[g]	$Pd(OAc)_2$ (0.5)	EtOAc:2-MeTHF (1:1)	1 h	37

[a]reaction conditions: catalyst, 0.5 mmol heteroarene and 1.2 equiv. diazonium salt in 2.5 mL solvent, rt, 0.1 equiv. decafluorobiphenyl as internal standard for GC-FID, open flask; [b]CFL irradiation used as visilble light source; [c]no presence of light; [d]solvent: acetone, diethyl ether, MeCN, toluene; [e]0,1 M; [f]0.5 M; [g]1.0 M;

Control experiments revealed that there was no dual catalysis present (Table S 1, entries 1-2).[1] It was found that the reaction was catalyzed by only palladium. Moreover, in the case of $Ru(bpy)_3Cl_2$ in combination with visible light, a significant amount (<10%) of biphenyl formation was detected on GC-MS, indicating that the photo induced radical chemistry was working, although not participating in the palladium cycle. In the case only $Pd(OAc)_2$ was used, no biphenyl formation was detected. Table S 1, entries 3-16 show the calculated GC-FID yields for all other solvent tried during solvent screening.

It was demonstrated that the use of EtOAc:2-MeTHF (1:1) was superior as solvent mixture. Several other ratios between the two solvents were examined, but all resulted in lower yields (Table S 1, entries 17-20). A brief concentration screening was carried out (Table S 1, entries 21-23). Both as well as lower as higher concentration than 0.2M resulted in sluggish reaction conditions. Caution: 1.0M reaction resulted in uncontrollable heat buildup once the reaction started.

2.2 Reaction progress analysis

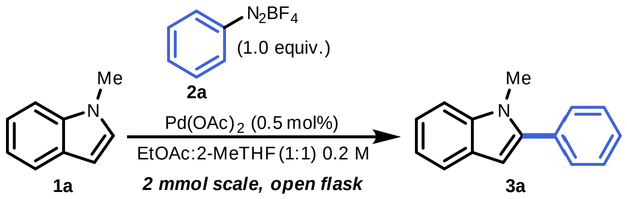

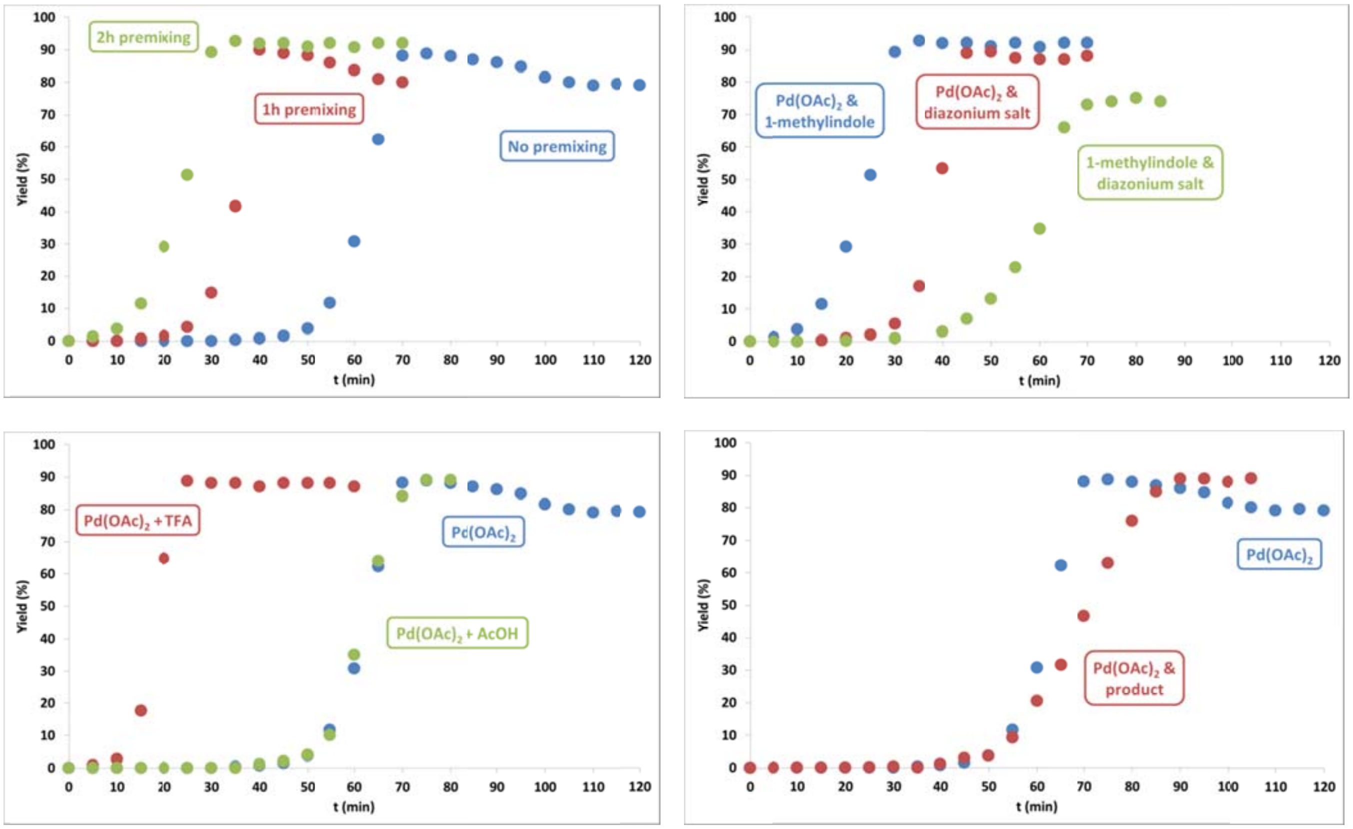

Figure S 1: Reaction progress analysis (yield as function of time). Upper left graph: A comparison of different premixing times. For no and 1h premixing, 1.1 equiv. of diazonium salt was used. Upper right graph: Different combinations of 2h premixing. Down left graph: Comparison between addition of TFA and AcOH (1.0 equiv.). Down right graph: Product addition (0.5 equiv.) to the reaction mixture.

For the reaction progress analysis, reactions were performed on a 2.0 mmol scale. Samples were taken every 5 minutes with a syringe and were immediately quenched by performing a micro-column with silica (in order to filter of catalyst and remaining aryldiazonium salt). EtOAc was used as eluent. The collected phase was extracted by an saturated $NaHCO_3$ (aq) solution. Finally, organic layer was analyzed on GC-FID. In order to calculate the appropriate yield, a calibration curve was made with decafluorobiphenyl as internal standard.

Upper left graph: Several experiments were conducted in order to find the necessary premixing time between $Pd(OAc)_2$ and 1-methylindole. No more induction time was present when premixing catalyst and substrate for 2 hours.

Upper right graph: Several combinations of premixing were conducted. In the case of premixing $Pd(OAc)_2$ with phenyldiazonium tetrafluoroborate for 2 hours, a slight shortening of induction period was observed. This can be related to the complexation of Pd(0) species with solvent. Although this effect was far less expressed than complexation with 1-methylindole. In case of premixing 1-methylindole with phenyldiazonium tetrafluoroborate, no decrease of induction period was observed. This observation correlates with theory of catalyst activation. After the reaction was complete, a slight lower yield was observed (approximate drop of 20%). This is due to byproduct (3-(arylazo)-1-methylindole **1aa**) formation during 2 hours premixing.

Down left graph: A remarkable observation was in the case of acid addition. The addition of 1.0 equiv. of TFA resulted in no more induction period (approx. same effect as 2h premixing $Pd(OAc)_2$). Although we are not sure, we consider that TFA acts as a better leaving group on the Pd(II) species, thereby allowing the reduction step of Pd(II) to Pd(0) to take place more easily. A control experiment with 1.0 equiv, of AcOH (excludes ligand effects) shows that indeed the induction period is unaffected by acidic conditions.

Down right graph: An experiment with the addition of 0.5 equiv. of 1-methyl-2-phenylindole (product) at start shows that there is a slight product inhibition behavior present during the reaction (less steep slope). This can be due to the fact that there is a competition between the ligation of substrate or product to the catalyst. As reported previously in the case of a C-H olefination protocol, the ligation state of the catalyst with product could lead to a slightly more stable complex, which in turn lower the catalyst performance for the desired reaction.[2] Although, no research has been done yet to proof this statement.

2.3 ¹H-NMR of the crude reaction mixture

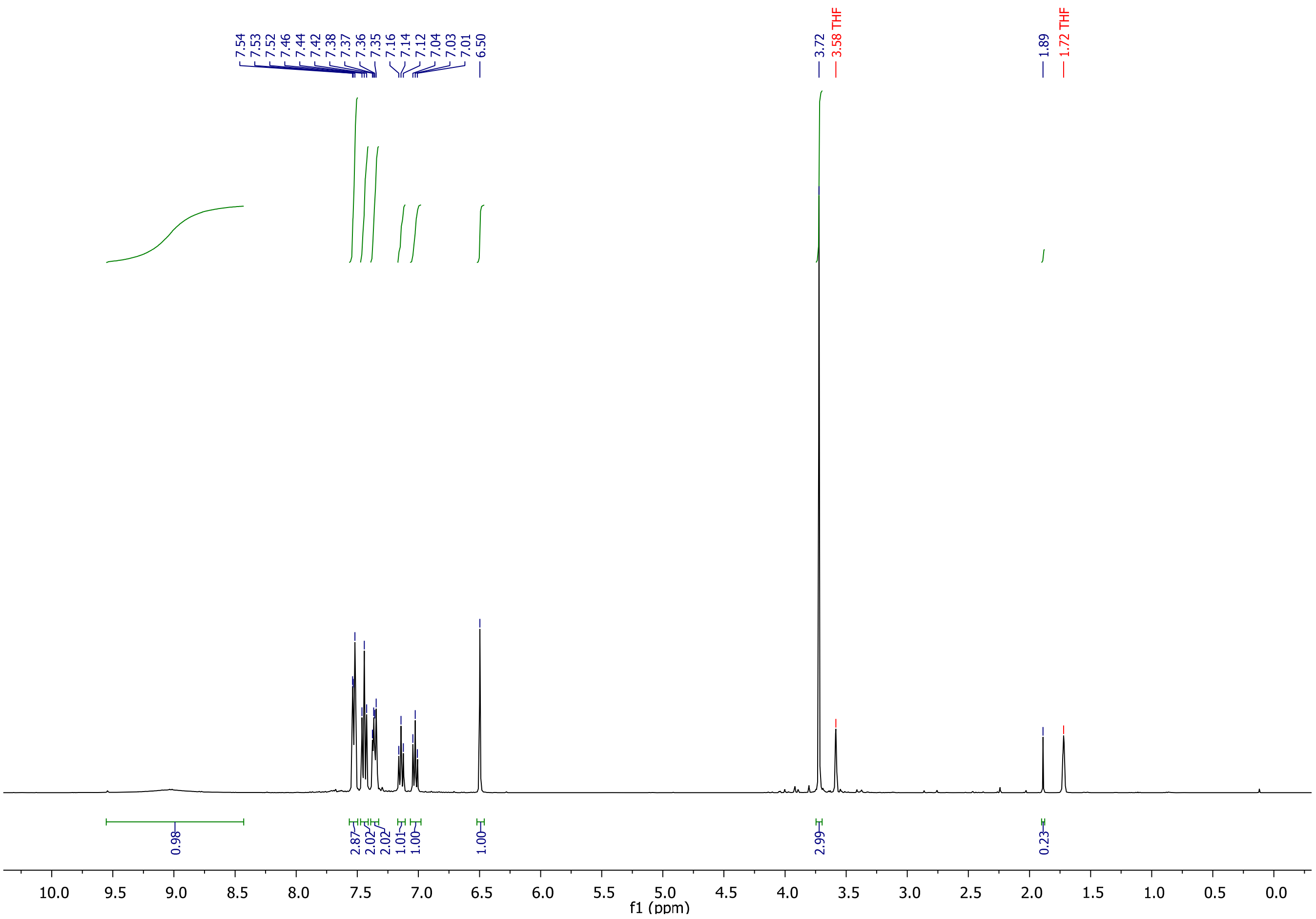

Figure S 2: ¹H-NMR taken of crude reaction mixture. Reaction conditions: 1.0 mol % Pd(OAc)2, 1.0 mmol 1-methylindole and 1.0 equiv. benzenediazonium salt in 5 mL d8-THF at rt, open flask, 1 h reaction;

A reaction was performed with the optimized reaction conditions in d8-THF. Note that, apart from the solvent residual peaks of d_8-THF, only the product signals are observed. In addition, a broad singlet at around 9 ppm is observed. Which is proposed to be the H^+ that is released after the anti-*ß*-deprotonation rearomatisation. With this crude ¹H-NMR we would like to emphasize the clean nature of our optimized reaction conditions.

2.4 Optimization for the Pd-catalyzed C–H arylation of benzofuran

Table S 2: Optimization table for Pd-catalyzed C–H arylation of benzofuran

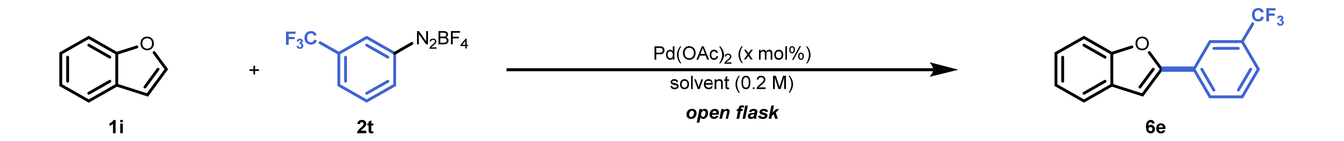

Entry	aryldiazonium salt (equiv.)	$Pd(OAc)_2$ (mol%)	Solvent	TFA (equiv.)	Reaction time	Isolated Yield (%)
1[b]	1.0	0.5	EtOAc:2-MeTHF (1:1)	-	overnight	17[c]
2	1.0	0.5	solvent[d]	-	overnight	<25[c]
3	1.0	0.5	MeOH	-	overnight	42[c]
4	1.0	0.5	MeOH	1.0	30 min	42
5	1.2	0.5	MeOH	1.0	30 min	48;52[c]
6	2.0	0.5	MeOH	1.0	30 min	48
7	1.2	1.0	MeOH	1.0	30 min	49
8[e]	1.2	0.5	MeOH	1.0	30 min	81

[a]reaction conditions: 0.5 – 1.0 mol % $Pd(OAc)_2$, 1.0 mmol heteroarene, 1.0 – 2.0 equiv. 3-trifluoromethylbenzenediazonium tetrafluoroborate and 0 – 1.0 equiv. TFA in 5 mL solvent at rt, [b]2h premixing of $Pd(OAc)_2$ with benzofuran; [c]F-NMR yield; [d]THF, EtOH, *i*PrOH, CF_3CH_2OH, EtOAc, MeCN; [e]after reaction 15 min reflux with acetyl chloride (5 equiv.);

A brief optimization in case of benzofuran was necessary, since optimized conditions for 1-methylindole did not give satisfying results (Table S 2, Entry 1). A solvent screening was conducted to find a more appropriate solvent (Table S 2, entries 2-3). It was found that MeOH offered the best results (42%). In the case of 1-methylindole, MeOH was considered a bad solvent since with polar protic solvents, the side reaction (electrophilic substitution) became significant (see main article). In the case of benzofuran no electrophilic substitution was observed, which allowed us to use the more reactive polar protic solvents. However, with the use of MeOH as solvent, overnight reaction was still necessary in order to obtain full conversion. Note, that it was found before in the optimization of 1-methylindole, that the addition of 1.0 equiv. of TFA helped accelerating the reaction (Figure S 1, down left graph), although it was not necessary in the case of 1-methylindole to add TFA (reaction was within 1 hour without TFA) for the scope. With this knowledge we considered that the addition of TFA could help accelerate the reaction in case of benzofuran. Satisfyingly, with the addition of TFA the reaction could be accelerated to only 30 minutes reaction time in order to obtain reaction completion (Table S 2, Entry 4). Selectivity was unaffected. Next, we sought out if it was possible to increase the yield of the desired product by using an excess of the 3-trifluoromethylbenzenediazonium tetrafluoroborate (Table S 2, entries 5-6). However, only a slight increase in the yield was noticed with the use of 1.2 equivalents. Next, a higher catalyst loading was carried out (Table S 2, Entry 7), but no increase was observed. What was found that there was a significant side reaction occurring with the solvent MeOH after the carbopalladation (this is discussed in the main article), forming a racemic mixture of 3-methoxy-2-(3-

trifluoromethyl)phenyl)-2,3-dihydrobenzofuran (**6ee**). Finally, after 15 minutes refluxing of the resulting reaction mixture with addition of 5.0 equiv. of acetyl chloride, the byproduct could easily be transformed to the desired product (Table S 2, Entry 8), obtaining a satisfying yield of 81%.

3. Mechanistic Studies: DFT calculations and experimental investigations.

3.1 Progress NMR experiments

3.1.1 Progress ^{1}H-NMR of mixing stoichiometric $Pd(OAc)_2$ and 1-methylindole in d_8-THF

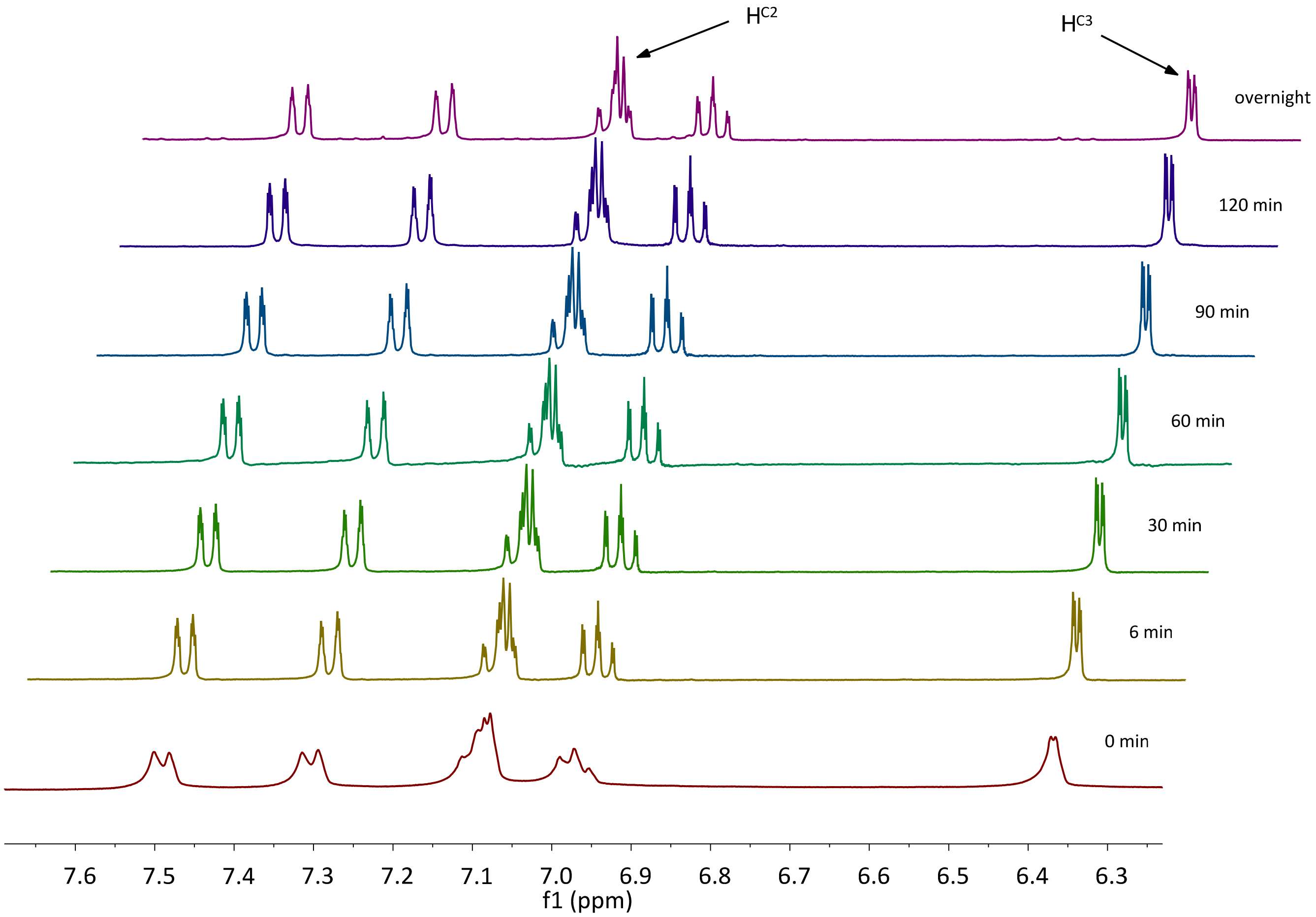

Figure S 3: Progress ^{1}H-NMR waterfall plot of stoichiometric experiment with $Pd(OAc)_2$ and 1-methylindole in d_8-THF

A waterfall plot was made of premixing stoichiometric amount of $Pd(OAc)_2$ and 1-methylindole in d_8-THF (0.2 M). Graphic at 0 min was taken before addition of $Pd(OAc)_2$. As can be seen from Figure S 3, both $H_{C\text{-}2}$ and $H_{C\text{-}3}$ remain unaffected throughout the plot. This indicates that there is no formal C–H activation occurring at the nucleophilic site of 1-methylindole. What was noted during progress ^{1}H-NMR is the change of color of reaction mixture (from brown to dark green). It was postulated that rather instead of C–H activation on the C-3 carbon of 1-methylindole, the latter substrate helped forming a non-covalent complex through its π-donating character, stabilizing formed homogeneous Pd(0) species, which in turn acts as the active catalyst for the oxidative addition with the aryldiazonium salt.

3.1.2 Progress ^{19}F-NMR of the reaction 6-fluoro-1-methylindole with 4-fluorobenzenediazonium tetrafluoroborate in EtOAc: 2-MeTHF (1:1)

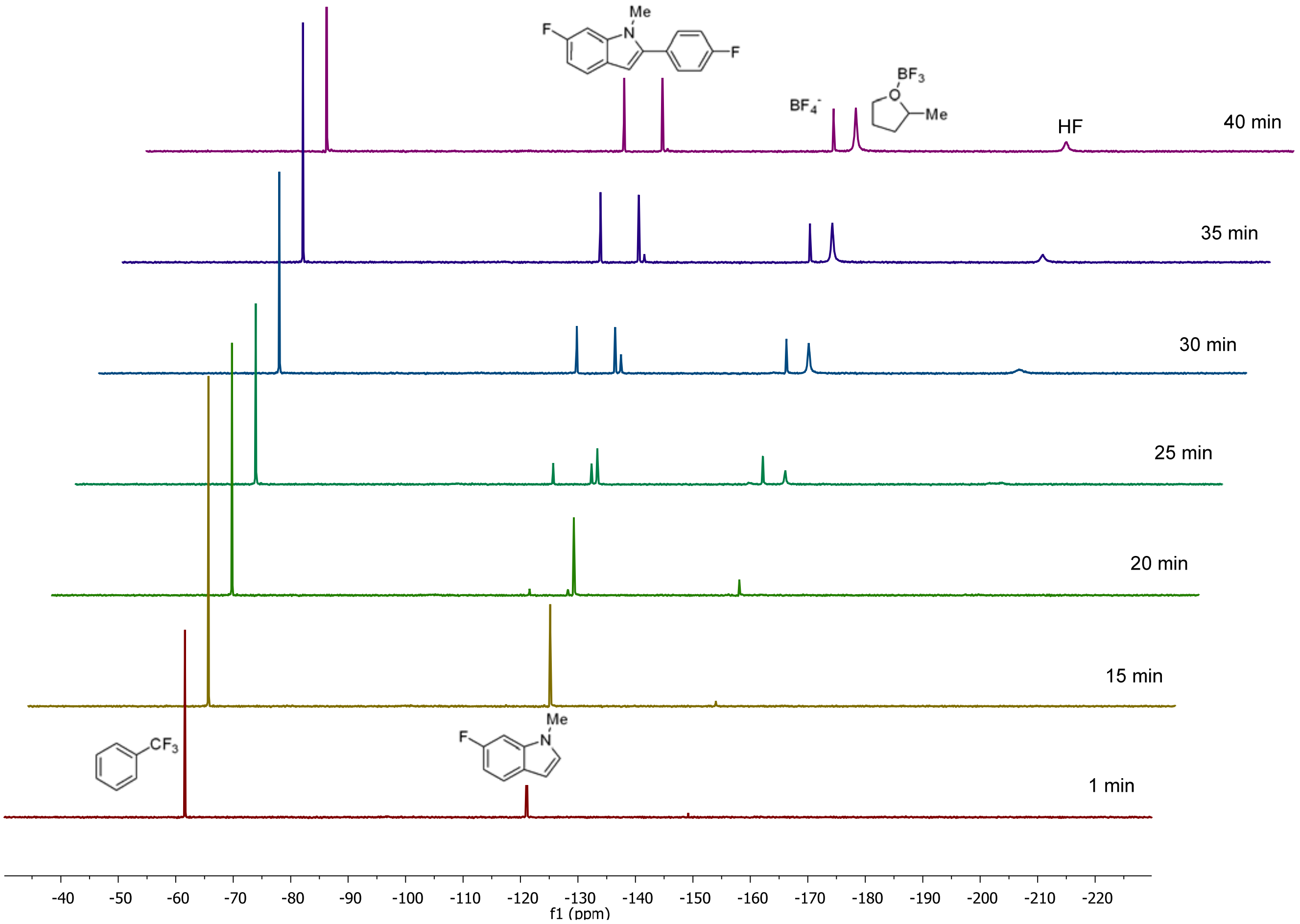

Figure S 4: Progress ^{19}F-NMR waterfall plot of the reaction 6-fluoro-1-methyl with 4-fluorobenzenediazonium tetrafluoroborate in EtOAc: 2-MeTHF (1:1)

A progress ^{19}F-NMR experiment was conducted in order to shed more light on the non-innocent behavior of the BF_4^- anion in the proposed Heck-catalytic cycle. The experiment was performed as following:

First a suspension of 4-fluorobenzenediazonium tetrafluoroborate (0.2 mmol) in 500 µL EtOAc:2-MeTHF (1:1) was loaded into the NMR tube. Note, that the 4-fluorobenzenediazonium tetrafluoroborate was not well soluble in the presented reaction mixture. Therefore, the fluorine signals F (-88.44 ppm) and BF4 (-151.97 ppm) of the diazonium salt cannot be used as quantitative parameters.

Next, a homogeneous solution of $Pd(OAc)_2$ (2.87 mol%), 6-fluoro-1-methylindole (0.2 mmol) and α,α,α-trifluorotoluene (0.067 mmol, as internal standard) in 500 µL EtOAc:2-MeTHF was added to the NMR tube.

The resulted reaction mixture was then monitored via NMR at room temperature. A ^{19}F-NMR of the reaction mixture was taken approximately every minute. Figure S 4 illustrates a waterfall plot of the ^{19}F-NMRs taken at 1, 15, 20, 25, 30, 35 and 40 minutes reaction time. Moreover, Figure S 5 represents the calculated yield of the observed compounds based on the its ^{19}F signal, as compared to the internal standard α,α,α-trifluorotoluene (-63.72 ppm). The ^{19}F signals in Figure S 6 at -155.82

ppm and -192.44 ppm could be distinguished as BF_3.2-MeTHF and HF respectively, when compared to the reference ^{19}F signals of $BF_3.OMe_2$ (Figure S 7) and generated HF (Figure S 8) in EtOAc:2-MeTHF (1:1). Gratifyingly, taken into account the fluorine signal ratios (1:3 for BF_3.2-MeTHF and 1:1 for HF compared to the -122.17 ppm signal of the product), a strong correlation was observed, indicating that both BF_3.2-MeTHF and HF are generated in the same rate as the desired product. As example, Figure S 6 illustrates this for 40 minutes reaction time (note integration of the ^{19}F signals). Derived from these experimental observations, we can postulate that the $BF4^-$ (which stabilizes the carbopalladation intermediate **II**) and the solvent 2-MeTHF both assist in the anti-*ß*-deprotonation rearomatisation, through the formation of HF and BF_3.2-MeTHF (see Scheme S 1). These experimental results present the first proof that $BF4^-$ anions play a non-innocent role in the Heck-catalytic cycle.

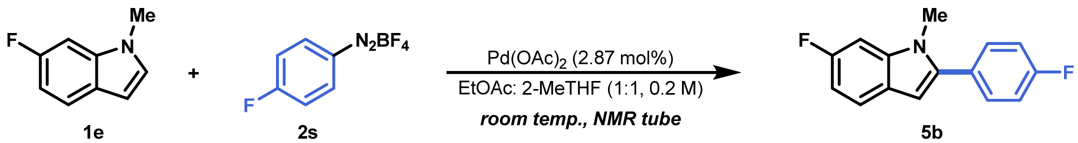

1e **2s** 1.0 equiv. **5b**

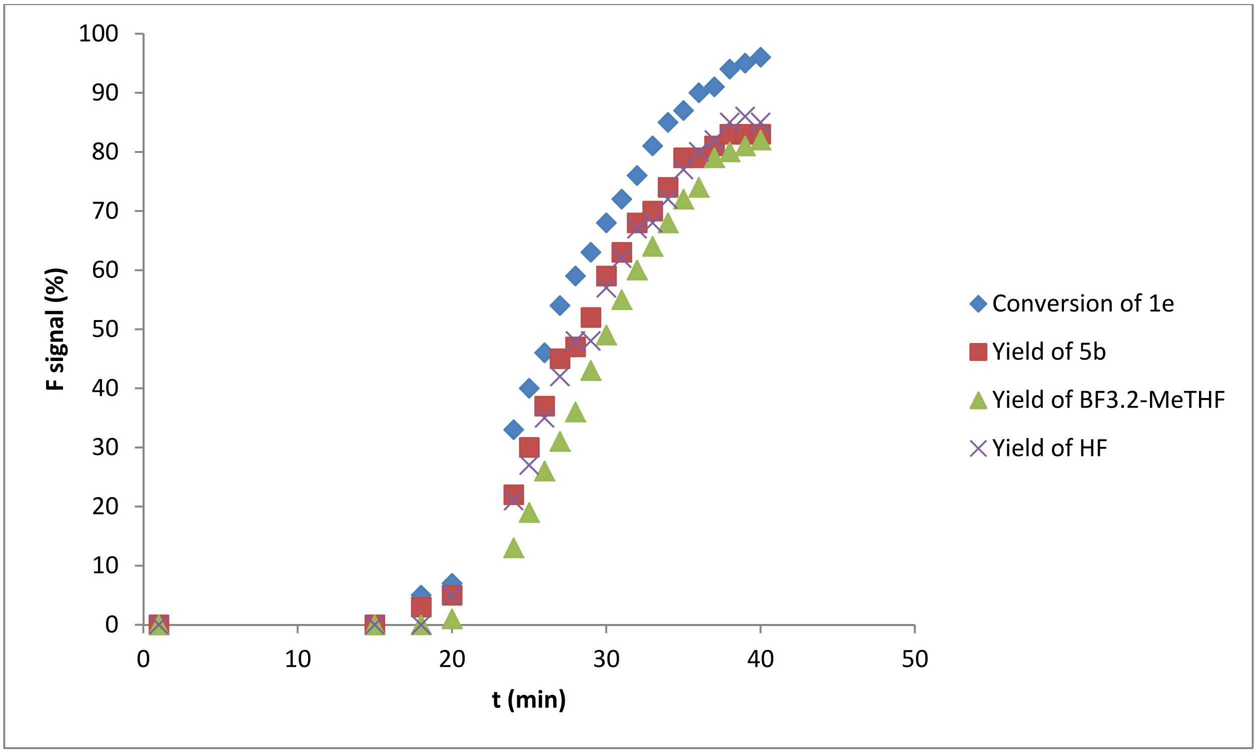

Figure S 5: ^{19}F-NMR monitoring of the reaction 6-fluoro-1-methyl with 4-fluorobenzenediazonium tetrafluoroborate in EtOAc: 2-MeTHF (1:1). Reaction conditions: 2.87 mol % $Pd(OAc)_2$, 0.2 mmol 6-fluoro-1-methylindole and 1.0 equiv. 4-fluorobenzenediazonium tetrafluoroborate in 1 mL EtOAc:2-MeTHF (1:1) at rt, NMR tube; Conversion and Yield was monitored by the ^{19}F signals of reaction mixture. α,α,α-trifluorotoluene (-63.72 ppm) was used as internal standard.

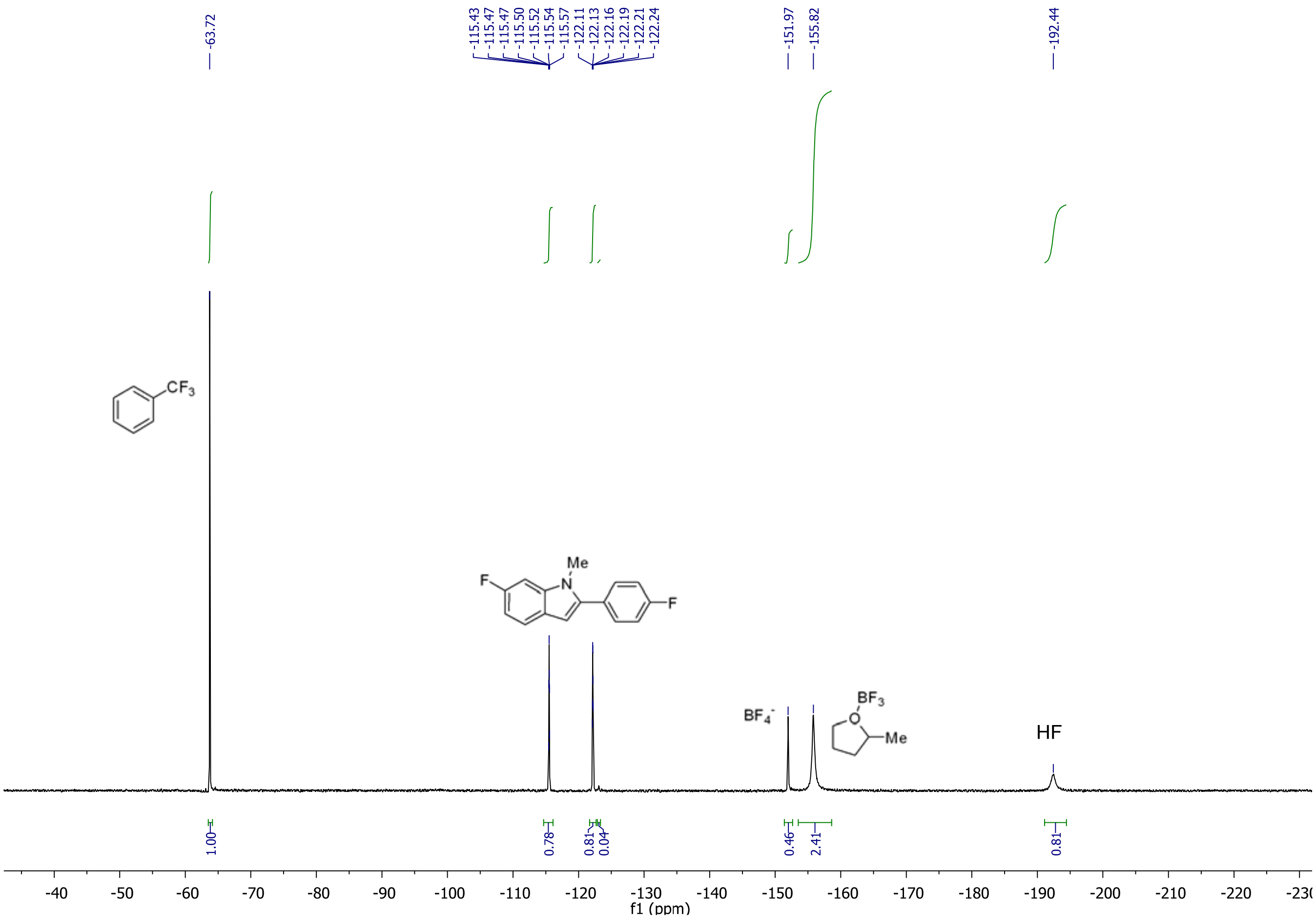

Figure S 6: ^{19}F-NMR of the reaction 6-fluoro-1-methyl with 4-fluorobenzenediazonium tetrafluoroborate in EtOAc: 2-MeTHF (1:1) after 40 min reaction time. Note, that a strong correlation was found in the integration of the signals of BF_3.2-MeTHF (-155.82 ppm) and F^- (-192.44 ppm) with 6-fluoro-1-methylindole (-115.50 and -122.17 ppm multiplets).

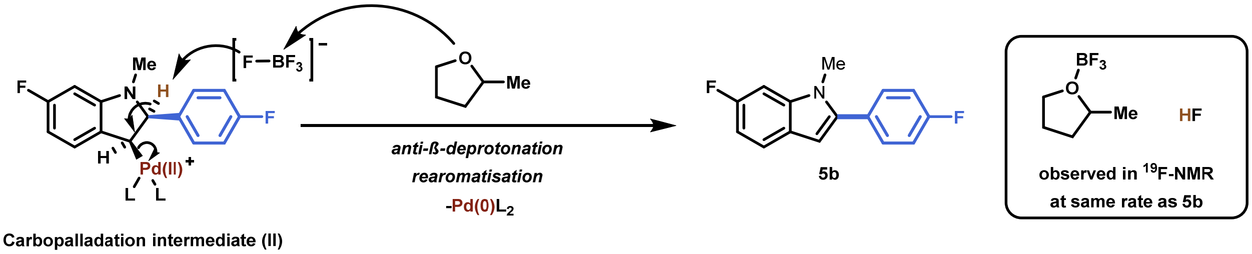

Scheme S 1: Proposed anti-*ß*-deprotonation rearomatisation assisted by the BF4- anion. Both BF_3.2-MeTHF and HF were observed in F-NMR in quantitative correlation to the desired product (**5b**).

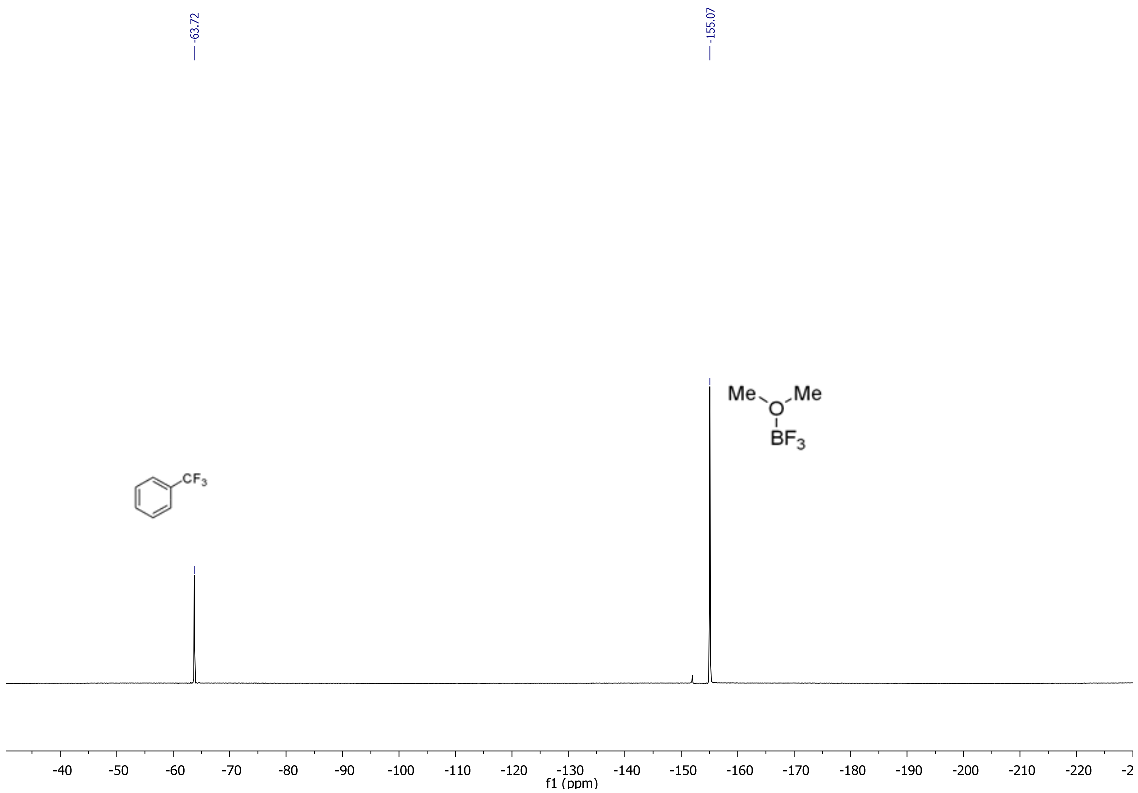

Figure S 7: ^{19}F-NMR of $BF_3.OMe_2$ dissolved in EtOAC:2-MeTHF (1:1) with α,α,α-trifluorotoluene (-63.72 ppm) used as internal standard. -155.07 ppm was identified as the ^{19}F signal of $BF_3.OMe_2$. Therefore, it is highly plausible that the signal at -155.82 ppm in the reaction mixture (Figure S 6) is that of BF_3.2-MeTHF.

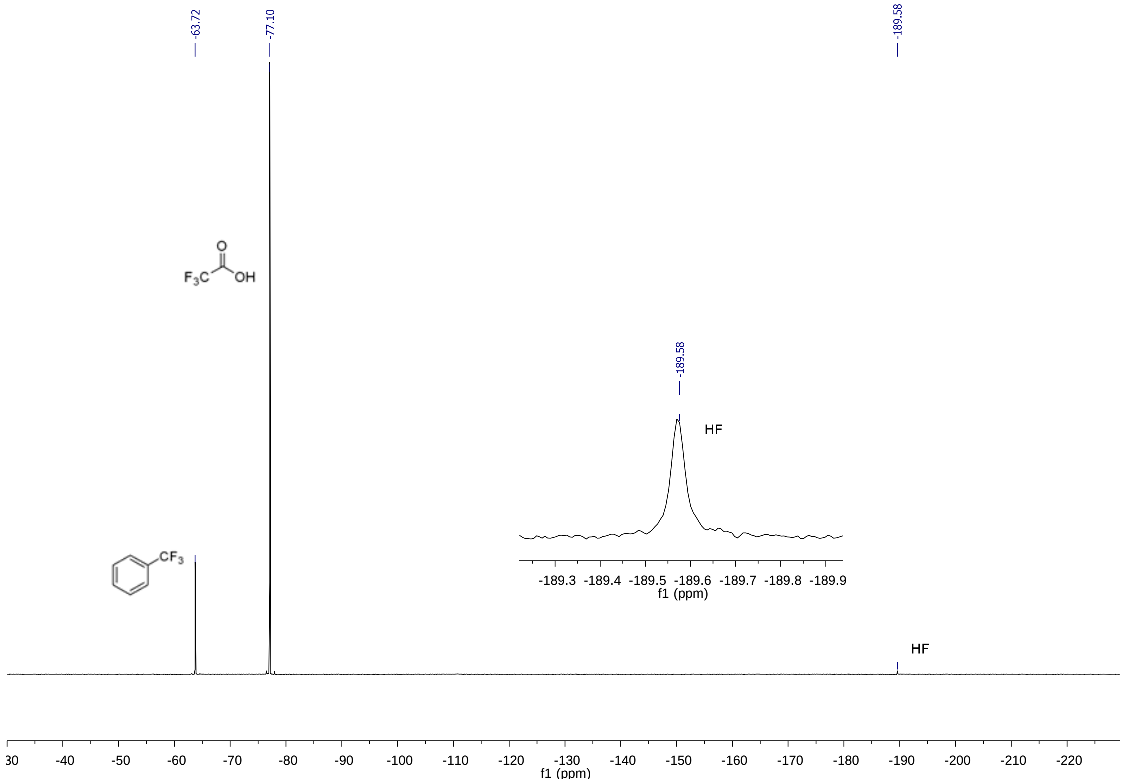

Figure S 8: ^{19}F-NMR of NaF (0.1 mmol) + trifluoroacetic acid (excess.) in 1 mL of EtOAC:2-MeTHF (1:1) with α,α,α-trifluorotoluene (-63.72 ppm) used as internal standard. -189.58 ppm was identified as the ^{19}F signal of *in situ* generated HF (derived from NaF + acid). Therefore, it is highly plausible that the signal at -192.44 ppm in the reaction mixture (Figure S 6) is that of *in situ* generated HF. Note: NaF was insoluble in EtOAc:2-MeTHF (1:1), a precipitate was present.

3.2 Radical scavenging experiments

Table S 3: Radical scavenging experiments

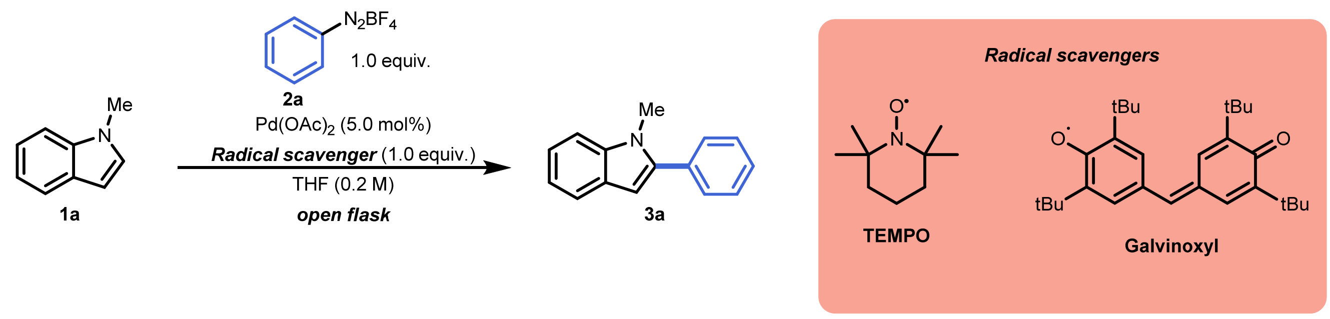

Entry	Radical scavenger (equiv.)	Addition radical scavenger	GC-MS yield before addition (%)	GC-MS yield after 30 min (%)	Comments for GC-MS
1	-	-	-	76	-
2	TEMPO (1.0)	5 min	5	5	No trapped intermediates
3	Galvinoxyl (1.0)	5 min	5	5	No trapped intermediates
4	TEMPO (1.0)	15 min	21	21	No trapped intermediates

[a]reaction conditions: 5.0 mol% $Pd(OAc)_2$, 0.5 mmol heteroarene and 1.0 equiv. diazonium salt in 2.5 mL solvent, rt, 0.1 equiv. decafluorobiphenyl as internal standard for GC-FID, open flask, 1.0 equiv. of radical scavenger added at indicated reaction time

Radical scavenging was carried out in order to find possible trapped intermediates. Radical scavengers used were TEMPO or Galvinoxyl (1.0 equiv.). Radical scavenger was added to the reaction mixture on a certain time after the reaction was started. Both scavenger were unable to trap any possible radical intermediates. Note, that upon addition of radical scavenger, the reaction stopped immediately (Table S 3, entries 2 – 4). However, this is more likely due to catalyst poisoning.

3.3 Control experiments regarding the non-innocent role of BF_4^-

Table S 4: Control experiments with other counterions[a]

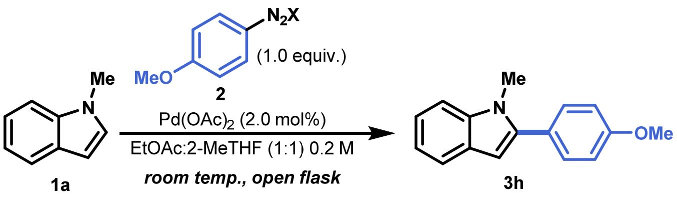

Entry	Counterion (X)	reaction time	isolated Yield (%)
1	BF_4^-	2 h	82[b]
2	OMs^-	16 h	0
3	OTs^-	2 h	62[c]

[a]Reaction conditions: 2.0 mol % $Pd(OAc)_2$, 0.5 mmol 1-methylindole and 1.0 equiv. 4-methoxybenzenediazonium salt in 2.5 mL EtOAc:2-MeTHF (1:1) at rt, open flask; [b]0.5 mol % $Pd(OAc)_2$; [c]addition of 0.5 equiv. BF_4^- (from a HBF_4 48wt% in H_2O solution) after 15 minutes;

Control experiments were conducted with 4-methoxybenzenediazonium mesylate (and tosylate) in order to verify the non-innocent behavior of BF_4^-. No desired product was observed using the latter aryldiazonium salts (Table S4). In case of 4-methoxybenzenediazonium tosylate, 0.5 equiv. of BF_4^- (as HBF4) was added to the reaction mixture after 15 minutes. Upon addition, immediate gas formation and color change was observed. After 2 hours, the reaction was completed and the desired product was obtained in 62% isolated yield.

The control experiments described above showcase that the presence of the counterion BF_4^- has a positive effect on the reaction performance. Thus, emphasizing the non-innocent behavior of BF_4^- (as hypothesized in the main manuscript).

3.4 Control experiments regarding the oxidative addition step

Table S 5: Trapping the aryldiazonium tetrafluoroborate via oxidative addition[a]

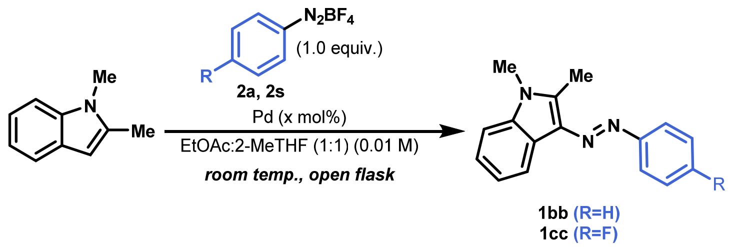

Entry	R	catalyst (mol %)	Reaction time	isolated Yield (%)
1	R = H	$Pd(OAc)_2$ (0.5)	2 h	93[b,c]
2	R = H	$Pd(OAc)_2$ (100)	16 h	42[b]
3	R = H	$Pd_2(dba)_3$ (100)	16 h	0[d]
4	R = H	$Pd(OAc)_2$ (0.5)	30 min	98[c]
5	R = H	$Pd(OAc)_2$ (100)	16 h	0

[a]Reaction conditions: 0.2 mmol 1,2-dimethylindole and 1.0 equiv. aryldiazonium tetrafluoroborate in 20 mL EtOAc:2-MeTHF (1:1) at rt, open flask; [b]2 h premixing of $Pd(OAc)_2$ with 1,2-dimethylindole; [c]0.2 M; [d]1 h premixing of $Pd_2(dba)_3$ with benzenediazonium tetrafluoroborate; Note: With high palladium loadings, 0.01 M was used to ensure a homogeneous reaction conditions;

In order to characterize the first step of the catalytic cycle (oxidative addition), the following experiments were conducted (Table S5). First, the reaction with 1,2-dimethylindole was repeated with 100 mol % $Pd(OAc)_2$ (Table S5, entries 1 and 2). As expected, a lower yield of the side product **1bb** was obtained (42% vs 93%) in case of 100 mol % $Pd(OAc)_2$. This can be explained by the fact that the uncatalyzed electrophilic substitution is competing with the oxidative addition. In Scheme S2, the competition between the oxidative addition with palladium and the uncatalyzed electrophilic substitution with 1,2-dimethylindole is illustrated:

- Case 1 (Table S5, entry 1): When only 0.5 mol% of Pd(OAc)2 is used, only a marginal amount (about 0.5 mol %) of the aryldiazonium salt will be occupied in the catalytic cycle (*Pathway A*). Note, that in the latter pathway the catalytic cycle cannot be completed due to the presence of the methyl substituent on the C2 carbon (of 1,2-dimethylindole). Therefore, the leftover aryldiazonium salt can now follow the 'slower' *Pathway B*, generating the side-product, hence the high yield of **1bb** (93%).
- Case 2 (Table S5, entry 2): In this case, the aryldiazonium salt will undergo oxidative addition (*Pathway A*). However, due to the observed induction period for the oxidative addition with Pd(II) sources (as previously discussed in the manuscript), *Pathway B* will still be feasible during this induction period. Therefore, it is reasonable that compound **1bb** is still observed, although in a lower amount (42%).

- Case 3 (Table S5, entry 3): When using $Pd_2(dba)_3$ it was previously shown (see manuscript) that no induction period was present for the oxidative addition step. Therefore, when conducting the experiment with 100 mol % $Pd_2(dba)_3$, no side product (**1bb**) formation was observed.

In order to further illustrate this observation, we conducted similar experiments with a more reactive aryldiazonium salt. Known from previous results (see manuscript), 4-fluorobenzenediazonium tetrafluoroborate did not show any induction period when using $Pd(OAc)_2$ as the catalyst. Therefore, the two reactions were conducted, one with 0.5 mol% $Pd(OAc)_2$ and another with 100 mol % $Pd(OAc)_2$ (in line with to case 1 and 2). In case of 0.5 mol % $Pd(OAc)_2$, 98% of **1cc** was obtained. However, in case of 100 mol % $Pd(OAc)_2$, no more side-product was observed (Table 2, entry 4 vs 5). This is in perfect alignment with our hypothesis.

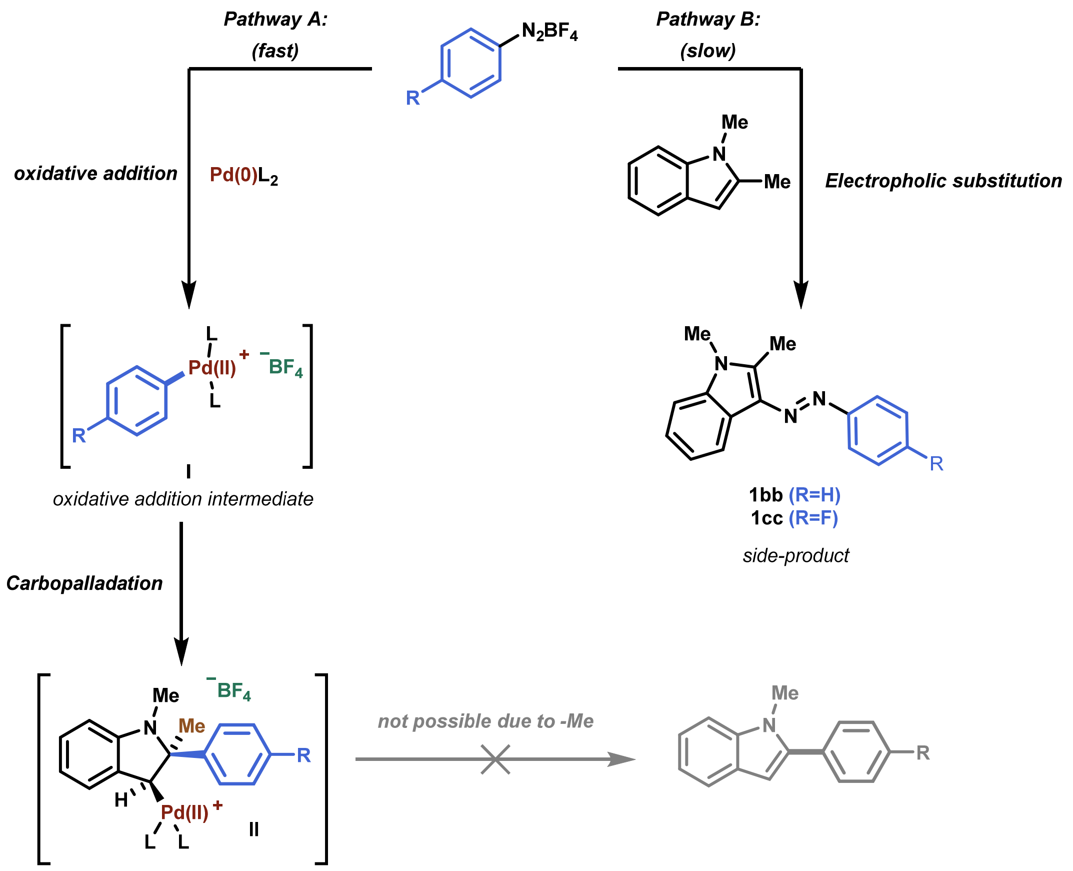

Scheme S 2: Competition reaction between Pathway A and B.

3.5 DFT calculations: Computational methods

All calculations were performed using the Gaussian 09 software.[3] Structural optimizations and frequency calculations were performed with ωB97X-D along with 6-31G(d) basis set on all atoms except Pd, I and the effective core potential (ECP) SDD on Pd and I atoms. Single point energy calculations were performed with M06L functional and def2-TZVP basis set on all atoms. Solvent effects of THF and MeOH were taken into account using the CPCM solvation model.

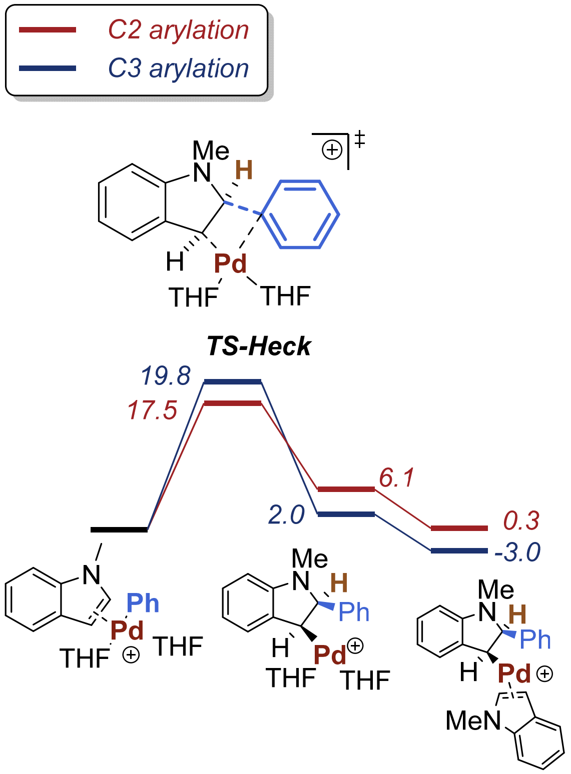

Figure S 9 Comparison of full pathways of the Heck-type carbopalladation of 1-methylindole. Ligand exchange with an additional substrate molecule makes the process thermodynamically feasible.

4. General procedure for the synthesis of aryldiazonium tetrafluoroborates

All aryldiazonium tetrafluoroborates were synthesized by following reported procedures:[4]

General procedure A.

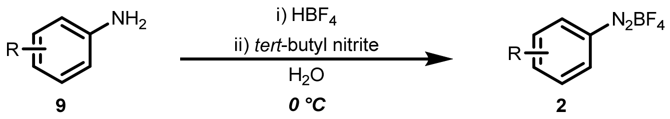

To a suspension of aniline (**9**) (10.0 mmol) in 5 mL water at rt was added HBF_4 (48wt% in water, 3.0 equiv.) and the reaction mixture was stirred for 2 min. The mixture was cooled to 0 °C and a solution of *tert*-butyl nitrite (1.2 equiv.) was added dropwise. After addition, the reaction mixture was stirred at 0 °C for 1 hour. The solids were filtered, washed with ice-cold diethyl ether to give the crude product. Recrystallization was done by dissolving crude product in a minimum of acetone followed by addition of ice-cold diethyl ether. Recrystallization was repeated until white solids were acquired (**2**).

In case the aryldiazonium tetrafluoroborate salt could not be precipitated by following general procedure A, general procedure B was carried out.

General procedure B.

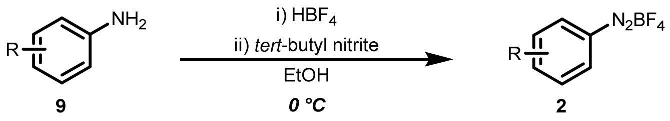

To a solution of aniline **9** (10.0 mmol) in 5 mL ethanol at rt was added HBF_4 (48% in water, 3.0 equiv.) and the reaction mixture was stirred for 2 min. The mixture was cooled to 0 °C and *tert*-butyl nitrite (1.5 equiv.) was added dropwise. After addition, the mixture was stirred at 0 °C for 2 hours. Diethyl ether was added to the reaction mixture and the resulting solids were filtered, washed with diethyl ether (3 x) and dried under high vacuum to give the title product (**2**).

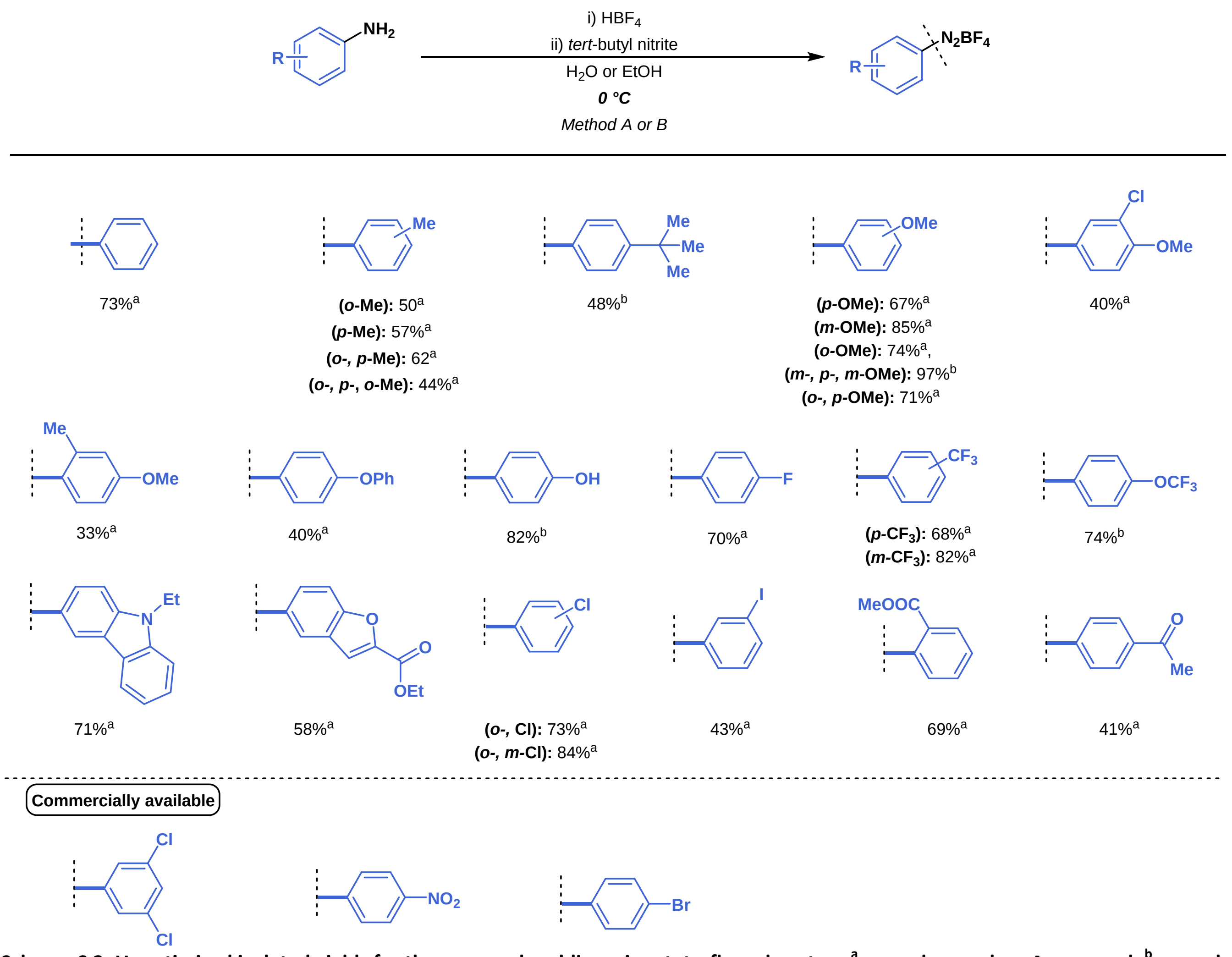

Scheme S 3: Unoptimized isolated yields for the prepared aryldiazonium tetrafluoroborates. [a]**general procedure A was used;** [b]**general procedure B was used.**

5. General procedure for the Pd-catalyzed C–H arylation of heteroarenes

Table S 6: Summary of conditions used for different substrates

Condition	Substrate	$Pd(OAc)_2$ (mol%)	Diazonium salt (equiv.)	Solvent (v:v)	Premixing (2 h)	TFA (equiv.)	Reflux with acetyl chloride	T (°C)
A	1-methylindole	0.5	1.0	EtOAc:2-MeTHF (1:1)	yes	-	No	25
B	NH-indole	1.0	1.0	EtOAc:2-MeTHF (1:1)	yes	-	No	25
C	Benzofuran	0.5	1.2	MeOH	no	1.0	Yes	25
D	benzothiophene	2.0	2.0	MeOH	no	1.0	No	40

5.1 General procedure for the C–H arylation of 1-methylindole and derivatives (condition A)

A stock solution was prepared by weighing $Pd(OAc)_2$ (5.6 mg, 0.5 mol%) and 1-methylindole (**1a**) (656 mg, 5.0 mmol) into a 50 mL round-bottom flask equipped with stirring bar. 25 mL freshly prepared EtOAc:2-MeTHF (1:1) mixture was added and the resulting solution was stirred for 2 h under air atmosphere at room temperature. Aryldiazonium salt (1.0 mmol, 1.0 equiv.) was weighted into a 20 mL reaction tube equipped with stirring bar. 5 mL of stock solution (containing 1.0 mmol of 1-methylindole and 0.5 mol% $Pd(OAc)_2$) was added immediately via syringe. Reaction mixture was stirred vigorously at room temperature until 1-methylindole was completely consumed (monitored by TLC). Reaction was quenched by addition of saturated NaHCO3 solution. The resulting mixture was moved to the separation funnel. Reaction vial was washed with EtOAc and added to the separating funnel. Layers were separated and the organic layer was washed with saturated aqueous $NaHCO_3$ and brine solution sequentially. Aqueous phase was washed with EtOAc. Remaining organic phase was dried over $MgSO_4$, filtered and concentrated under reduced pressure. Purification by flash chromatography afforded the product. The final product was characterized by ^1H NMR, ^{13}C NMR, ^{19}F NMR (if applicable), HRMS, IR and melting point analysis (if applicable).

5.2 General procedure for the C–H arylation of NH-indole (conditions B)

A stock solution was prepared by weighing $Pd(OAc)_2$ (11.2 mg, 1.0 mol%) and NH-indole (**1b**) (586 mg, 5.0 mmol) into a 50 mL round-bottom flask equipped with stirring bar. 25 mL freshly prepared EtOAc:2-MeTHF (1:1) mixture was added and the resulting solution was stirred for 2 h under air atmosphere at room temperature. Aryldiazonium salt (1.0 mmol, 1.0

equiv.) was weighted into a 20 mL reaction tube equipped with stirring bar. 5 mL of stock solution (containing 1.0 mmol of NH-indole and 1.0 mol% $Pd(OAc)_2$) was added immediately via syringe. Reaction mixture was stirred vigorously at room temperature until NH-indole was completely consumed (monitored by TLC). Reaction was quenched by addition of saturated NaHCO3 solution. The resulting mixture was moved to the separation funnel. Reaction vial was washed with EtOAc and added to the separating funnel. Layers were separated and the organic layer was washed with saturated aqueous NaHCO3 and brine solution sequentially. Aqueous phase was washed with EtOAc. Remaining organic phase was dried over MgSO4, filtered and concentrated under reduced pressure. Purification by flash chromatography afforded the product. The final product was characterized by 1H NMR, 13C NMR, 19F NMR (if applicable), HRMS, IR and melting point analysis (if applicable).

5.3 General procedure for the C–H arylation of benzofuran (condition C)

A stock solution was prepared by weighing $Pd(OAc)_2$ (5.6 mg, 0.5 mol%) and benzofuran (**1g**) (591 mg, 5.0 mmol) into a 25 mL volumetric flask. TFA (370 µL, 1.0 equiv.) was added and the volumetric flask was filled up to 25 mL with anhydrous MeOH. Aryldiazonium salt (1.2 mmol, 1.2 equiv.) was weighted into a 20 mL reaction tube equipped with stirring bar. 5 mL of stock solution (containing 1.0 mmol of benzofuran, 0.5 mol% $Pd(OAc)_2$ and 1.0 equiv. of TFA) was added immediately via syringe. Reaction mixture was stirred vigorously at room temperature until benzofuran was completely consumed (monitored by TLC). Resulting reaction mixture was moved to a 50 mL round-bottom flask and acetyl chloride (350 µL, 5.0 equiv.) was added dropwise. A reflux condenser was mounted on top of the round-bottom flask. The reaction mixture was heated and refluxed for about 15 minutes. After cooling down to room temperature, the reaction was quenched by addition of saturated NaHCO3 solution. The resulting mixture was moved to the separation funnel. Round-bottom flask was washed with EtOAc and added to the separating funnel. Layers were separated and the organic layer was washed with saturated aqueous NaHCO3 and brine solution sequentially. Aqueous phase was washed with EtOAc. Remaining organic phase was dried over MgSO4, filtered and concentrated under reduced pressure. Purification by flash chromatography afforded the product. The final product was characterized by 1H NMR, 13C NMR, 19F NMR (if applicable), HRMS, IR and melting point analysis (if applicable).

5.4 General procedure for the C–H arylation of benzothiophene (condition D)

A stock solution was prepared by weighing $Pd(OAc)_2$ (22.4 mg, 2.0 mol%) and benzothiophene (**1h**) (671 mg, 5.0 mmol) into a 25 mL volumetric flask. TFA (370 µL, 1.0 equiv.) was added and the volumetric flask was filled up to 25 mL with anhydrous MeOH. Aryldiazonium salt (2.0 mmol, 2.0 equiv.) was weighted into a 20 mL reaction tube equipped with stirring bar. 5 mL of stock solution (containing 1.0 mmol of benzothiophene, 2.0 mol% $Pd(OAc)_2$ and 1.0 equiv. of TFA) was added immediately via syringe. Reaction mixture was stirred vigorously at 40 °C until benzothiophene was completely consumed (monitored by TLC). The reaction was quenched by addition of saturated NaHCO3 solution. The resulting

mixture was moved to the separation funnel. Reaction vial was washed with EtOAc and added to the separating funnel. Layers were separated and the organic layer was washed with saturated aqueous NaHCO3 and brine solution sequentially. Aqueous phase was washed with EtOAc. Remaining organic phase was dried over MgSO4, filtered and concentrated under reduced pressure. Purification by flash chromatography afforded the product. The final product was characterized by 1H NMR, 13C NMR, 19F NMR (if applicable), HRMS, IR and melting point analysis (if applicable).

5.5 Procedure for the gram scale C–H arylation of 1-methylindole

Scheme S 4: Reaction conditions for gram scale C–H arylation of 1-methylindole

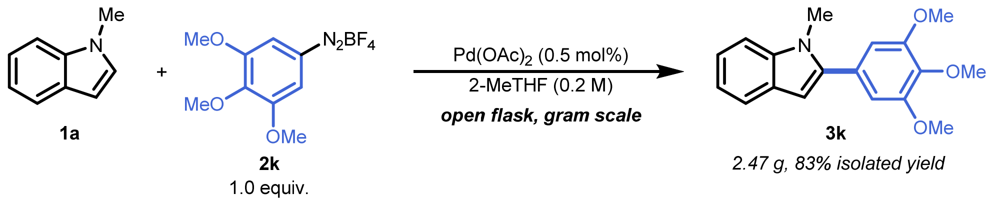

$Pd(OAc)_2$ (11.2 mg, 0.5 mol%) and 1-methylindole (**1a**) (1.31 g, 10.0 mmol) were weighted into a 250 mL round-bottom flask equipped with stirring bar. 50 mL anhydrous 2-MeTHF was added and the resulting solution was stirred for 2 h under air atmosphere at room temperature. 3,4,5-trimethoxyphenyldiazonium tetrafluoroborate (**2e**) (2.82 g, 1.0 equiv.) was weighted and added to the reaction mixture in portions. Reaction mixture was stirred vigorously at room temperature for 4 hours untill 1-methylindole was completely consumed (monitored by TLC). Reaction was quenched by addition of saturated NaHCO3 solution. The resulting mixture was moved to the separation funnel. Reaction vial was washed with EtOAc and added to the separating funnel. Layers were separated and the organic layer was washed with saturated aqueous $NaHCO_3$ and brine solution sequentially. Aqueous phase was washed with EtOAc. Remaining organic phase was dried over $MgSO_4$, filtered and concentrated under reduced pressure. Purification by flash chromatography on silica (5% EtOAc in petroleum ether) afforded 1-methyl-2-(3,4,5-trimethoxyphenyl)-*1H*-indole (**3k**) (2.47 g, 83%) as an off-white solid. Mp. 105 °C; ^1H NMR (400 MHz, Chloroform-d) δ 7.64 (dt, J = 8.0, 1.0 Hz, 1H), 7.37 (d, J = 8.0 Hz, 1H), 7.29 – 7.23 (m, 1H), 7.16 (ddd, J = 8.0, 7.1, 1.0 Hz, 1H), 6.72 (s, 2H), 6.56 (s, 1H), 3.94 (s, 3H), 3.92 (s, 6H), 3.77 (s, 3H); ^{13}C NMR (101 MHz, Chloroform-d) δ 153.5, 141.8, 138.5, 138.3, 128.6, 128.1, 122.0, 120.7, 120.2, 109.9, 107.0, 101.7, 61.3, 56.5, 31.5; IR (ATR, cm^{-1}): 2936, 2830, 1582, 1497, 1464, 1412, 1341, 1304, 1236, 1125, 1005; HRMS (ESI) calculated for $C_{18}H_{19}NO_3$ $[M+H]^+$: 298.1438; found: 298.1441.

6. Procedure for synthesis of Saprisartan precursor

6.1 Synthesis of 5-methylbenzofuran substrate

Preparation of 1-(2,2-diethoxyethoxy)-4-methylbenzene (3ab)[5]

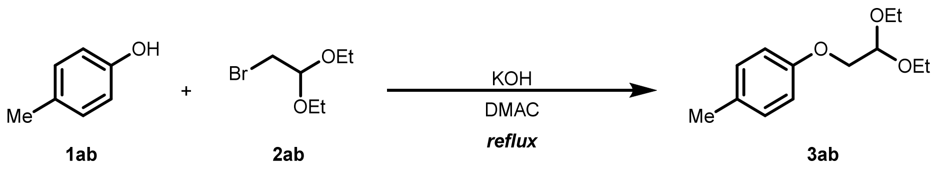

A 250 mL round-bottom flask was charged with p-cresol (**1ab)** (5.4 g, 50 mmol), DMAC (100 mL) and KOH (5.6 g, 100 mmol), followed by adding dropwise 2-bromoacetaldehyde diethyl acetal (**2ab**) (14.8 g, 75 mmol) at room temperature. After the addition, the mixture was stirred under refluxing for 2 hours until the reaction was completed (monitored by TLC). Then the mixture was cooled to room temperature and extracted with EtOAc and saturated brine solution. Next, the organic layer was washed subsequently with 5% NaOH aqueous solution, 10% HCl aqueous solution and saturated $NaHCO_3$ solution. Remaining organic phase was dried over $MgSO_4$, filtered and concentrated under reduced pressure. Purification by flash chromatography on silica (5% EtOAc in petroleum ether) afforded 1-(2,2-diethoxyethoxy)-4-methylbenzene (**3ab**) (10,6 g, 95% yield).

Preparation of 5-methylbenzofuran (1i)[6]

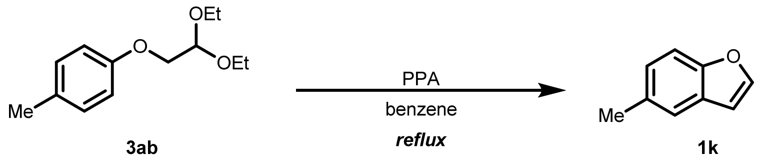

1-(2,2-diethoxyethoxy)-4-methylbenzene (**3ab**) (10.6 g, 47,5 mmol) and poly-phosphoric acid (10.2 g) were combined in 200mL of benzene and brought to reflux for 2 days. The reaction mixture was cooled to room temperature and extracted with EtOAc and saturated brine solution. Next, the organic layer was washed subsequently with 5% NaOH aqueous solution, 10% HCl aqueous solution and saturated $NaHCO_3$ solution. Remaining organic phase was dried over $MgSO_4$, filtered and concentrated under reduced pressure. Purification by flash chromatography on silica (petroleum ether) afforded 5-methylbenzofuran (**1k**)(2.0 g, 36% yield).

6.2 Synthesis of Saprisartan precursor (8a)

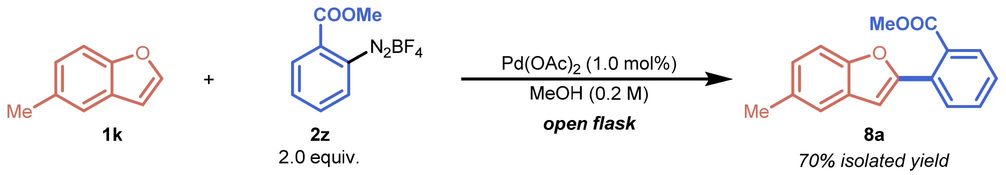

A stock solution was prepared by weighing $Pd(OAc)_2$ (11.2 mg, 1.0 mol%) and 5-methylbenzofuran (**1i**) (660 mg, 5.0 mmol) into a 25 mL volumetric flask. TFA (370 μL, 1.0 equiv.) was added and the volumetric flask was filled up to 25 mL with

anhydrous MeOH. *o*-COOMe-benzenediazonium tetrafluoroborate (**2y**) (2.0 mmol, 2.0 equiv.) was weighted into a 20 mL reaction tube equipped with stirring bar. 5 mL of stock solution (containing 1.0 mmol of 5-methylbenzofuran, 1.0 mol% $Pd(OAc)_2$ and 1.0 equiv. of TFA) was added immediately via syringe. Reaction mixture was stirred vigorously at 40 °C until 5-methylbenzofuran was completely consumed (2 hours). The reaction was quenched by addition of saturated NaHCO3 solution. The resulting mixture was moved to the separation funnel. Reaction vial was washed with EtOAc and added to the separating funnel. Layers were separated and the organic layer was washed with saturated aqueous NaHCO3 and brine solution sequentially. Aqueous phase was washed with EtOAc. Remaining organic phase was dried over MgSO4, filtered and concentrated under reduced pressure. Purification by flash chromatography on silica (5% EtOAc in petroleum ether) afforded methyl 2-(5-methylbenzofuran-2-yl)benzoate (**8a**) (186 mg, 70%) as a yellow oil; ^{1}H NMR (399 MHz, Chloroform-d) δ 7.78 – 7.69 (m, 2H), 7.55 (td, J = 7.6, 1.3 Hz, 1H), 7.44 (td, J = 7.6, 1.3 Hz, 1H), 7.41 – 7.36 (m, 2H), 7.12 (dd, J = 8.4, 1.4 Hz, 1H), 6.87 (s, 1H), 3.82 (s, 3H), 2.46 (s, 3H); ^{13}C NMR (100 MHz, Chloroform-d) δ 169.6, 154.8, 153.7, 132.5, 131.1, 131.0, 129.8, 129.4, 129.1, 129.0, 128.6, 126.0, 121.1, 110.7, 104.3, 52.6, 21.5; IR (ATR, cm-1): 2949, 2920, 1726, 1449, 1289, 1265, 1123, 1090, 918, 754; HRMS (ESI) calculated for $C_{17}H_{14}O_3$ $[M+H]^+$: 267.1016; found: 267.1016.

7. Characterization data for the Pd-catalyzed C–H arylated heteroarenes

1-methyl-2-phenyl-*1H*-indole (3a)[7]

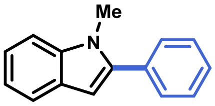

3a, 90% (1h)

Reaction conditions: 1.0 mmol scale, method A, 1 h reaction time; Purification by flash chromatography on silica (2% diethylether in petroleum ether) afforded 1-methyl-2-phenyl-*1H*-indole (186 mg, 90%) as white solids; Mp. 101 °C; ^{1}H-NMR (399 MHz, Chloroform-d) δ 7.63 (d, J = 7.8 Hz, 1H), 7.54 – 7.47 (m, 2H), 7.46 (t, J = 7.4 Hz, 2H), 7.42 – 7.33 (m, 2H), 7.23 (d, J = 7.8 Hz, 1H), 7.14 (t, J = 7.4 Hz, 1H), 6.56 (s, 1H), 3.74 (s, 3H); ^{13}C-NMR (100 MHz, Chloroform-d) δ 141.7, 138.4, 133.0, 129.5, 128.6, 128.1, 128.0, 121.8, 120.6, 120.0, 109.7, 101.8, 31.3; IR (ATR, cm-1): 3055, 2920, 2850, 1602, 1468, 1340, 1308, 796, 765, 748; HRMS (ESI) calculated for $C_{15}H_{14}N$ [M+H]+: 208.1121;; found: 208.1120.

1,3-dimethyl-2-phenyl-*1H*-indole (3b)[8]

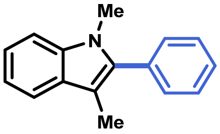

3b, 80% (1h)

Reaction conditions: 1.0 mmol scale, method A, 1h reaction time; Purification by flash chromatography on silica (petroleum ether) afforded 1,3-dimethyl-2-phenyl-*1H*-indole (176 mg, 80%) as colorless oil; ^{1}H NMR (400 MHz, Chloroform-d) δ 7.65 (d, J = 7.9 Hz, 1H), 7.53 (t, J = 7.6 Hz, 2H), 7.45 (t, J = 6.8 Hz, 3H), 7.37 (d, J = 8.1 Hz, 1H), 7.30 (t, J = 7.6 Hz, 1H), 7.20 (t, J = 7.6 Hz, 1H), 3.65 (s, 3H), 2.33 (s, 3H); ^{13}C NMR (100 MHz, Chloroform-d) δ 137.8, 137.3, 132.3, 130.8, 128.6, 128.45, 127.9, 121.9, 119.2, 118.9, 109.4, 108.7, 31.1, 9.5; IR (ATR, cm-1): 3049, 2914, 1468, 1354, 1236, 1005, 818, 737, 700; HRMS (ESI) calculated for $C_{16}H_{16}N$ $[M+H]^+$: 222.1277; found: 222.1275.

1-methyl-2-(2-methylphenyl)-1*H*-indole (3c)[9]

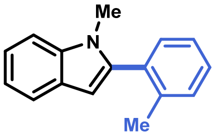

3c, 65% (1h)

Reaction conditions: 1.0 mmol scale, method A, 1 h reaction time; Purification by flash chromatography on silica (petroleum ether) afforded 1-methyl-2-(2-methylphenyl)-1*H*-indole (144 mg, 65%) as a yellowish solid; Mp. 90 °C; ^{1}H NMR (400 MHz, Chloroform-d) δ 7.83 (dd, *J* = 7.7, 1.0 Hz, 1H), 7.55 – 7.39 (m, 6H), 7.34 (td, *J* = 7.2, 1.0 Hz, 1H), 6.62 (s, 1H), 3.66 (s, 3H), 2.38 (s, 3H); ^{13}C NMR (101 MHz, Chloroform-d) δ 140.6, 138.2, 137.4, 132.7, 131.3, 130.2, 128.8, 128.1, 125.7, 121.4, 120.5,

119.8, 109.6, 101.7, 30.4, 20.2; IR (ATR, cm-1): 3419.8, 3053.7, 2954.6, 1603.3, 1573.1, 1465.5, 1431.4, 1360.2, 1336.7, 1313.5, 1274.2, 1235.0; HRMS (ESI) calculated for $C_{16}H_{16}N$ $[M+H]^+$: 222.1278; found: 222.1275.

1-methyl-2-(*p*-tolyl)-*1H*-indole (3d)[7]

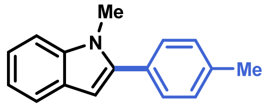

3d, 88% (1h)
79% (1 h)*
**Pd2(dba)3*

Reaction conditions: 1.0 mmol scale, method A, 1 h reaction time; Purification by flash chromatography on silica (1% diethyl ether in *n*-heptane) afforded 1-methyl-2-(*p*-tolyl)-*1H*-indole (195 mg, 88%) as white solids; Mp. 97 °C; ^{1}H NMR (500 MHz, Chloroform-d) δ 7.65 (dt, *J* = 7.8, 1.0 Hz, 1H), 7.45 – 7.40 (m, 2H), 7.37 (d, *J* = 8.2 Hz, 1H), 7.33 – 7.21 (m, 3H), 7.16 (ddd, *J* = 8.0, 7.1, 1.0 Hz, 1H), 6.56 (s, 1H), 3.75 (s, 3H), 2.44 (s, 3H); ^{13}C NMR (101 MHz, Chloroform-d) δ 141.8, 138.4, 137.9, 130.1, 129.5, 129.4, 128.2, 121.7, 120.5, 120.1, 109.7, 101.5, 31.3, 21.5; IR (ATR, cm-1): 3051, 3022, 2920, 1464, 1383, 1338, 1317, 827, 773, 734; HRMS (ESI) calculated for $C_{16}H_{16}N$ $[M+H]^+$: 222.1277; found: 222.1272.

2-(2',4'-dimethylphenyl)-1-methyl-1*H*-indole (3e)

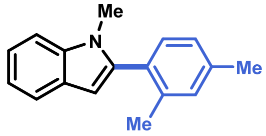

3e, 66% (0.5h)

Reaction conditions: 1.0 mmol scale, method A, 0.5 h reaction time; Purification by flash chromatography on silica (petroleum ether) afforded 2-(2',4'-dimethylphenyl)-1-methyl-1*H*-indole (155 mg, 66%) as a yellowish solid; Mp. 80 °C; ^{1}H NMR (400 MHz, Chloroform-d) δ 7.70 (d, J = 7.9 Hz, 1H), 7.41 (d, J = 8.1 Hz, 1H), 7.35 – 7.18 (m, 4H), 7.14 (d, J = 7.7 Hz, 1H), 6.48 (s, 1H), 3.57 (s, 3H), 2.46 (s, 3H), 2.23 (s, 3H); ^{13}C NMR (101 MHz, Chloroform-d) δ 140.6, 138.5, 137.9, 137.4, 131.1, 130.94, 129.7, 128.1, 126.4, 121.3, 120.4, 119.7, 109.5, 101.6, 30.3, 21.6, 20.1; IR (ATR, cm-1): 3042, 2918, 2853, 1466, 1377, 1362, 1335, 1312, 1130, 1003; HRMS (ESI) calculated for $C_{17}H_{18}N$ $[M+H]^+$: 236.1434; found: 236.1433.

2-mesityl-1-methyl-1*H*-indole (3f – C-2)

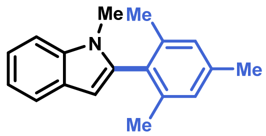

3f, 30% (3h)
[C2]

Reaction conditions: 1.0 mmol scale, slightly modified method A (1.0 mol% Pd, 1.2 equiv. aryldiazonium salt), 3 h reaction time; Purification by flash chromatography on silica (1% ethyl acetate in petroleum ether) afforded 2-mesityl-1-methyl-1*H*-

indole (76 mg, 30%) as white solids; Mp. 84 °C; ^{1}H NMR (399 MHz, Chloroform-d) δ 7.67 (d, *J* = 8.1 Hz, 1H), 7.38 (d, *J* = 8.1 Hz, 1H), 7.26 (td, *J* = 8.1, 7.0, 0.8 Hz, 1H), 7.17 (td, *J* = 8.1, 7.0, 0.8 Hz, 1H), 7.01 (s, 2H), 6.37 (s, 1H), 3.45 (s, 3H), 2.39 (s, 3H), 2.06 (s, 6H); ^{13}C NMR (100 MHz, Chloroform-d) δ 139.3, 138.8, 138.5, 137.2, 129.5, 128.5, 128.2, 121.0 , 120.5, 119.5, 109.5, 101.1, 29.8, 21.4, 20.5; IR (ATR, cm-1): 3053, 2918, 2853, 1612, 1464, 1335, 1310, 856, 779, 748; HRMS (ESI) calculated for $C_{18}H_{20}N$ $[M+H]^+$: 250.1591; found: 250.1597.

3-mesityl-1-methyl-1*H*-indole (3f – C-3)[10]

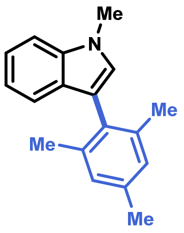

3f, 34% (3h)
[C3]

Reaction conditions: 1.0 mmol scale, slightly modified method A (1.0 mol% Pd, 1.2 equiv. aryldiazonium salt), 3 h reaction time; Purification by flash chromatography on silica (1% ethyl acetate in petroleum ether) afforded 3-mesityl-1-methyl-1*H*-indole (84 mg, 34%) as off-white solids; Mp. 106 °C; ^{1}H NMR (399 MHz, Chloroform-d) δ 7.38 (d, *J* = 8.2 Hz, 1H), 7.29 – 7.23 (m, 2H), 7.11 – 7.05 (m, 1H), 7.00 (s, 2H), 6.89 (s, 1H), 3.86 (s, 3H), 2.37 (s, 3H), 2.09 (s, 6H); ^{13}C NMR (100 MHz, Chloroform-d) δ 138.6, 137.0, 136.6, 131.2, 128.1, 128.0, 127.3, 121.6, 120.2, 119.2, 114.4, 109.3, 33.0, 29.9, 21.1; IR (ATR, cm-1): 2916, 2849, 1549, 1462, 1371, 1323, 1223, 847, 740; HRMS (ESI) calculated for $C_{18}H_{20}N$ $[M+H]^+$: 250.1591; found: 250.1591.

2-(4-(*tert*-butyl)phenyl)-1-methyl-1*H*-indole (3g)[11]

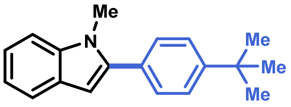

3g, 77% (16 h)

Reaction conditions: 1.0 mmol scale, method A, 16 h reaction time; Purification by flash chromatography on silica (2% diethyl ether in n-heptane) afforded 2-(4-(*tert*-butyl)phenyl)-1-methyl-1*H*-indole (202 mg, 77%) as white solids; Mp. 115 °C; ^{1}H NMR (400 MHz, Chloroform-d) δ 7.67 (d, *J* = 7.8 Hz, 1H), 7.51 (q, *J* = 8.5 Hz, 4H), 7.39 (d, *J* = 8.1 Hz, 1H), 7.32 – 7.23 (m, 1H), 7.17 (t, *J* = 7.4 Hz, 1H), 6.58 (s, 1H), 3.79 (s, 3H), 1.42 (s, 9H); ^{13}C NMR (101 MHz, Chloroform-d) δ 151.1, 141.8, 138.5, 130.1, 129.2, 128.2, 125.6, 121.7, 120.5, 112.0, 109.7, 101.5, 34.9, 31.5, 31.4; IR (ATR, cm-1): 3049, 2961, 2864, 1466, 1360, 1339, 1165, 847, 781, 737; HRMS (ESI) calculated for $C_{19}H_{22}N$ $[M+H]^+$: 264.1747; found: 264.1740.

2-(4-methoxyphenyl)-1-methyl-*1H*-indole (3h)[7]

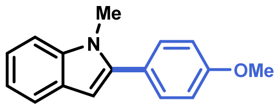

3h, 82% (2h)

Reaction conditions: 1.0 mmol scale, method A, 2 h reaction time; Purification by flash chromatography on silica (4% diethyl ether in petroleum ether) afforded 2-(4-methoxyphenyl)-1-methyl-*1H*-indole (194mg, 82%) as white solids; Mp. 120 °C; ^{1}H NMR (400 MHz, Chloroform-d) δ 7.62 (d, *J* = 7.8 Hz, 1H), 7.44 (d, *J* = 8.8 Hz, 2H), 7.35 (d, *J* = 8.2 Hz, 1H), 7.22 (td, *J* = 7.9, 7.0, 1.1 Hz, 1H), 7.13 (td, *J* = 7.7, 6.9, 1.0 Hz, 1H), 7.01 (d, *J* = 8.8 Hz, 2H), 6.50 (s, 1H), 3.87 (s, 3H), 3.73 (s, 3H); ^{13}C NMR (101 MHz, Chloroform-d) δ 159.6, 141.6, 138.3, 130.8, 128.2, 125.4, 121.6, 120.4, 119.9, 114.1, 109.7, 101.2, 55.5, 31.2; IR (ATR, cm-1): 3007, 2939, 2835, 1606, 1494, 1452, 1285, 1242, 1178, 744; HRMS (ESI) calculated for $C_{16}H_{15}NO$ $[M+H]^+$: 238.1227; found: 238.1230.

2-(3-methoxyphenyl)-1-methyl-1*H*-indole (3i)[12]

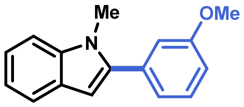

3i, 71% (1h)

Reaction conditions: 1.0 mmol scale, method A, 1 h reaction time; Purification by flash chromatography on silica (2% diethyl ether in petroleum ether) afforded 2-(3-methoxyphenyl)-1-methyl-1*H*-indole (168 mg, 71%) as a greenish solid; Mp. 63 °C; ^{1}H NMR (400 MHz, Chloroform-d) δ 7.67 (d, *J* = 8.0 Hz, 1H), 7.45 – 7.36 (m, 2H), 7.31 – 7.25 (m, 1H), 7.20 – 7.16 (m, 1H), 7.13 (dt, *J* = 8.0, 1.1 Hz, 1H), 7.10 – 7.08 (m, 1H), 7.00 – 6.96 (m, 1H), 6.61 (s, 1H), 3.89 (s, 3H), 3.78 (s, 3H); ^{13}C NMR (101 MHz, Chloroform-d) δ 159.8, 141.6, 138.5, 134.3, 129.7, 128.1, 122.0, 121.9, 120.7, 120.1, 115.2, 113.5, 109.8, 101.9, 55.5, 31.4; IR (ATR, cm-1): 2938, 1738, 1603, 1578, 1464, 1339, 1283, 1233, 1161, 1047; HRMS (ESI) calculated for $C_{16}H_{16}NO$ $[M+H]^+$: 238.1227; found: 238.1227.

2-(2-methoxyphenyl)-1-methyl-*1H*-indole (3j)[13]

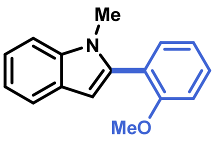

3j, 90% (2h)

Reaction conditions: 1.0 mmol scale, method A, 2 h reaction time; Purification by flash chromatography on silica (2% diethyl ether in petroleum ether) afforded 2-(2-methoxyphenyl)-1-methyl-*1H*-indole (214 mg, 90%) as a pink solid; Mp. 108 °C; ^{1}H NMR (400 MHz, Chloroform-d) δ 7.54 (ddd, J = 8.0, 1.2, 0.8 Hz, 1H), 7.34 – 7.23 (m, 3H), 7.13 (ddd, J = 8.3, 7.0, 1.1 Hz, 1H), 7.02 (ddd, J = 8.0, 7.0, 1.1 Hz, 1H), 6.94 (td, J = 7.5, 1.1 Hz, 1H), 6.89 (dd, J = 8.3, 1.0 Hz, 1H), 6.40 (s, 1H), 3.68 (s, 3H),

3.47 (s, 3H); ^{13}C NMR (101 MHz, Chloroform-d) δ 157.6, 138.7, 137.7, 132.6, 130.2, 128.1, 122.1, 121.4, 120.8, 120.5, 119.5, 111.0, 109.5, 101.8, 55.6, 30.8; IR (ATR, cm-1): 3049, 2936, 1738, 1464, 1435, 1339, 1312, 1246, 1115, 1024, 777, 746; HRMS (ESI) calculated for $C_{16}H_{16}NO$ $[M+H]^+$: 238.1226; found: 238.1224.

1-methyl-2-(3,4,5-trimethoxyphenyl)-1*H*-indole (3k)[14]

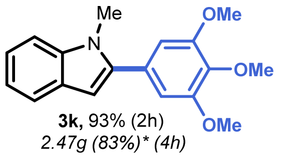

3k, 93% (2h)
2.47g (83%) (4h)*
**gram scale*

Reaction conditions: 1.0 mmol scale, method A, 2 h reaction time; Purification by flash chromatography on silica (10% ethyl acetate in petroleum ether) afforded 1-methyl-2-(3,4,5-trimethoxyphenyl)-1*H*-indole (277 mg, 93%) as a white solid; Mp. 105 °C; ^{1}H NMR (400 MHz, Chloroform-d) δ 7.64 (dt, *J* = 8.0, 1.0 Hz, 1H), 7.37 (d, *J* = 8.0 Hz, 1H), 7.29 – 7.23 (m, 1H), 7.16 (ddd, *J* = 8.0, 7.1, 1.0 Hz, 1H), 6.72 (s, 2H), 6.56 (s, 1H), 3.94 (s, 3H), 3.92 (s, 6H), 3.77 (s, 3H); ^{13}C NMR (101 MHz, Chloroform-d) δ 153.5, 141.8, 138.5, 138.3, 128.6, 128.1, 122.0, 120.7, 120.2, 109.9, 107.0, 101.7, 61.3, 56.5, 31.5; IR (ATR, cm-1): 2936, 2830, 1582, 1497, 1464, 1412, 1341, 1304, 1236, 1125, 1005; HRMS (ESI) calculated for $C_{18}H_{20}NO_3$ $[M+H]^+$: 298.1438; found: 298.1441.

2-(2,4-dimethoxyphenyl)-1-methyl-1*H*-indole (3l)

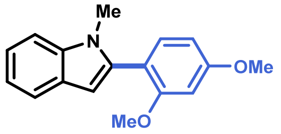

3l, 32% (2h)

Reaction conditions: 1.0 mmol scale, method A, 2 h reaction time; Purification by flash chromatography on silica (4% diethyl ether in petroleum ether) afforded 2-(2,4-dimethoxyphenyl)-1-methyl-1*H*-indole (86 mg, 32%) as a greenish solid; Mp. 125 °C; ^{1}H NMR (400 MHz, Chloroform-d) δ 7.65 (d, *J* = 7.9 Hz, 1H), 7.37 (d, *J* = 7.9 Hz, 1H), 7.33 – 7.28 (m, 1H), 7.27 – 7.22 (m, 1H), 7.14 (t, *J* = 7.4 Hz, 1H), 6.64 – 6.58 (m, 2H), 6.48 (s, 1H), 3.90 (s, 3H), 3.81 (s, 3H), 3.60 (s, 3H); ^{13}C NMR (101 MHz, Chloroform-d) δ 161.8, 158.9, 138.8, 137.8, 133.3, 128.2, 121.3, 120.6, 119.6, 115.0, 109.5, 104.7, 101.7, 98.9, 55.7, 55.6, 30.9; IR (ATR, cm-1): 2938, 2835, 1738, 1611, 1464, 1300, 1206, 1157, 1030, 833; HRMS (ESI) calculated for $C_{17}H_{18}NO_2$ $[M+H]^+$: 268.1332; found: 268.1332.

2-(3-chloro-4-methoxyphenyl)-1-methyl-1*H*-indole (3m)

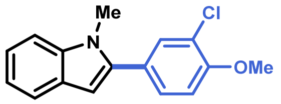

3m, 74% (0.5h)

Reaction conditions: 1.0 mmol scale, method A, 0.5 h reaction time; Purification by flash chromatography on silica (4% diethyl ether in petroleum ether) afforded 2-(3-chloro-4-methoxyphenyl)-1-methyl-1*H*-indole (200 mg, 74%) as a white solid; Mp. 119 °C; ^{1}H NMR (400 MHz, Chloroform-d) δ 7.65 (dt, *J* = 8.0, 1.0 Hz, 1H), 7.55 (d, *J* = 2.1 Hz, 1H), 7.40 – 7.35 (m, 2H), 7.27 (ddd, *J* = 8.0, 7.0, 1.0 Hz, 1H), 7.17 (ddd, *J* = 8.0, 7.0, 1.0 Hz, 1H), 7.03 (d, *J* = 8.5 Hz, 1H), 6.54 (s, 1H), 3.98 (s, 3H), 3.74 (s, 3H); ^{13}C NMR (101 MHz, Chloroform-d) δ 155.0, 140.1, 138.4, 131.2, 128.9, 128.0, 126.4, 122.7, 121.9, 120.6, 120.1, 112.1, 109.8, 101.8, 56.4, 31.3; IR (ATR, cm-1): 3055, 2926, 2839, 1491, 1464, 1431, 1288, 1256, 1063, 1020; HRMS (ESI) calculated for $C_{16}H_{15}ClNO$ [M+H]$^+$: 272.0837; found: 272.0831.

2-(4-methoxy-2-methylphenyl)-1-methyl-1*H*-indole (3n)

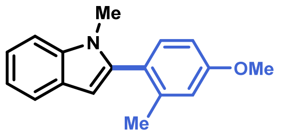

3n, 69% (1h)

Reaction conditions: 1.0 mmol scale, method A, 1 h reaction time; Purification by flash chromatography on silica (2% diethyl ether in petroleum ether) afforded 2-(4-methoxy-2-methylphenyl)-1-methyl-*1H*-indole (176 mg, 69%) as a white solid; Mp. 95 °C; ^{1}H NMR (400 MHz, Chloroform-d) δ 7.66 (dt, *J* = 7.5, 1.0 Hz, 1H), 7.37 (d, *J* = 8.3 Hz, 1H), 7.30 – 7.22 (m, 2H), 7.17 (td, *J* = 7.5, 7.1, 1.1 Hz, 1H), 6.90 (d, *J* = 2.7 Hz, 1H), 6.84 (dd, *J* = 8.3, 2.7 Hz, 1H), 6.44 (s, 1H), 3.88 (s, 3H), 3.53 (s, 3H), 2.21 (s, 3H); ^{13}C NMR (101 MHz, Chloroform-d) δ 160.0, 140.5, 139.8, 137.4, 132.4, 128.2, 125.1, 121.3, 120.4, 119.7, 115.6, 111.1, 109.5, 101.7, 55.5, 30.4, 20.5; IR (ATR, cm-1): 3001, 2938, 1738, 1609, 1464, 1366, 1292, 1234, 1207, 1161; HRMS (ESI) calculated for $C_{17}H_{18}NO$ [M+H]$^+$: 252.1383; found: 252.1379.

1-methyl -2-(4-phenoxyphenyl)-1*H*-indole (3o)

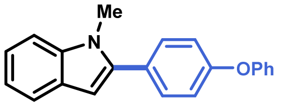

3o, 79% (3h)

Reaction conditions: 1.0 mmol scale, method A, 3 h reaction time; Purification by flash chromatography on silica (2% diethyl ether in petroleum ether) afforded 1-methyl -2-(4-phenoxyphenyl)-1*H*-indole (236 mg, 79%) as a white solid; Mp. 85 °C; ^{1}H NMR (400 MHz, Chloroform-d) δ 7.65 (d, *J* = 7.6 Hz, 1H), 7.52 – 7.46 (m, 2H), 7.44 – 7.35 (m, 3H), 7.27 (t, *J* = 7.6 Hz, 1H), 7.21 – 7.06 (m, 6H), 6.56 (s, 1H), 3.77 (s, 3H); ^{13}C NMR (101 MHz, Chloroform-d) δ 157.6, 156.9, 141.2, 138.4, 131.0, 130.1, 128.1, 127.8, 123.9, 121.8, 120.6, 120.0, 119.6, 118.6, 109.7, 101.6, 31.3; IR (ATR, cm-1): 3017, 2926, 1738, 1589, 1487, 1364, 1231, 1165, 783, 750; HRMS (ESI) calculated for $C_{21}H_{18}NO$ [M+H]$^+$: 300.1383; found: 300.1375.

2-(4'-hydroxyphenyl)-1-methyl-*1H*-indole (3p)[15]

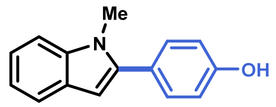

3p, 16% (2h)

Reaction conditions: 1.0 mmol scale, method A, 2 h reaction time; Purification by flash chromatography on silica (25% ethyl acetate in petroleum ether) afforded 2-(4'-hydroxyphenyl)-1-methyl-*1H*-indole (35 mg, 16%) as orange solid; Mp. 109 °C; ^{1} H NMR (399 MHz, Chloroform-d) δ 7.65 (d, J = 7.8 Hz, 1H), 7.43 – 7.35 (m, 3H), 7.30 – 7.23 (m, 1H), 7.17 (t, J = 7.4 Hz, 1H), 6.95 – 6.90 (m, 2H), 6.52 (s, 1H), 5.05 (s, 1H), 3.73 (s, 3H); ^{13}C NMR (100 MHz, Chloroform-d) δ 155.5, 141.5, 138.2, 131.0, 128.1, 125.6, 121.6, 120.4, 119.9, 115.6, 109.7, 101.1, 31.2; IR (ATR, cm-1): 3447, 3053, 2945, 1612, 1548, 1497, 1465, 1260, 1240, 1171; HRMS (ESI) calculated for $C_{15}H_{14}NO$ [M+H]$^{+}$: 224.1070; found: 224.1076.

9-ethyl-3-(1'-methyl-1'H-indol-2'-yl)-*9H*-carbazole (3q)

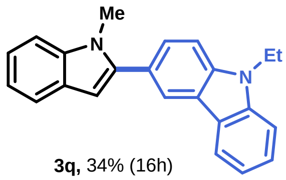

3q, 34% (16h)

Reaction conditions: 1.0 mmol scale, method A, 16 h reaction time; Purification by flash chromatography on silica (1% ethyl acetate in petroleum ether) afforded 9-ethyl-3-(1'-methyl-1'H-indol-2'-yl)-*9H*-carbazole (110 mg, 34%) as yellow solids; Mp. 137 °C; ^{1}H NMR (400 MHz, Chloroform-d) δ 8.25 (d, J = 1.2 Hz, 1H), 8.16 (d, J = 7.7 Hz, 1H), 7.69 (d, J = 7.7 Hz, 1H), 7.63 (dd, J = 8.4, 1.7 Hz, 1H), 7.57 – 7.45 (m, 3H), 7.42 (d, J = 8.2 Hz, 1H), 7.34 – 7.24 (m, 2H), 7.22 – 7.16 (m, 1H), 6.65 (s, 1H), 4.44 (q, J = 7.2 Hz, 2H), 3.82 (s, 3H), 1.51 (t, J = 7.2 Hz, 3H). ^{13}C NMR (101 MHz, Chloroform-d) δ 142.9, 140.5, 139.7, 138.3, 128.3, 127.4, 126.2, 123.6, 123.2, 122.9, 121.6, 121.4, 120.7, 120.4, 119.9, 119.3, 109.7, 108.8, 108.5, 101.4, 37.8, 31.3, 14.0; IR (ATR, cm-1): 3049, 2945, 2891, 1626, 1601, 1458, 1429, 1340, 1281, 1229, 789, 737; HRMS (ESI) calculated for $C_{23}H_{21}N_2$ [M+H]+: 325.1700; found: 325.1700.

Ethyl 5-(1-methyl-1*H*-indol-2-yl)benzofuran-2-carboxylate (3r)

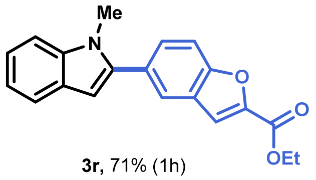

3r, 71% (1h)

Reaction conditions: 1.0 mmol scale, method A, 1 h reaction time; Purification by flash chromatography on silica (5% ethyl acetate in petroleum ether) afforded ethyl 5-(1-methyl-1*H*-indol-2-yl)benzofuran-2-carboxylate (227 mg, 71%) as off-white solids; Mp. 113 °C; ^{1}H NMR (399 MHz, Chloroform-d) δ 7.80 (d, *J* = 1.5 Hz, 1H), 7.68 (dd, *J* = 13.2, 8.2 Hz, 2H), 7.62 – 7.57 (m,

2H), 7.39 (d, J = 8.2 Hz, 1H), 7.29 (dd, J = 7.0, 1.1 Hz, 1H), 7.21 – 7.14 (m, 1H), 6.59 (s, 1H), 4.49 (q, J = 7.1 Hz, 2H), 3.75 (s, 3H), 1.46 (t, J = 7.1 Hz, 3H); ^{13}C NMR (100 MHz, Chloroform-d) δ 159.6, 155.5, 146.7, 141.2, 138.4, 129.4, 129.0, 128.1 , 127.4, 123.7, 122.0, 120.6, 120.1, 113.9, 112.6, 109.8, 102.2, 61.9, 31.3, 14.5; IR (ATR, cm-1): 3094, 2922, 2853, 1730, 1570, 1373, 1335, 1300, 1237, 1200; HRMS (ESI) calculated for $C_{20}H_{18}NO_3$ [M+H]$^+$: 320.1281; found: 320.1284.

2-(4-fluorophenyl)-1-methyl-1*H*-indole (3s)[11]

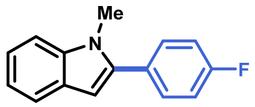

3s, 87% (20 min)

Reaction conditions: 1.0 mmol scale, method A, 20 min reaction time; Purification by flash chromatography on silica (1% ethyl acetate in petroleum ether) afforded 2-(4-fluorophenyl)-1-methyl-1*H*-indole (196 mg, 87%) as a white solid; Mp. 119 °C; ^{1}H NMR (400 MHz, Chloroform-d) δ 7.66 (dt, J = 7.8, 0.9 Hz, 1H), 7.52 – 7.47 (m, 2H), 7.39 (dd, J = 8.2, 1.2 Hz, 1H), 7.29 (ddd, J = 8.2, 7.0, 1.2 Hz, 1H), 7.23 – 7.15 (m, 3H), 6.56 (d, J = 0.6 Hz, 1H), 3.74 (s, 3H); ^{13}C NMR (101 MHz, Chloroform-d) δ 162.8 (d, J = 246.2 Hz), 140.6, 138.4, 131.2(d, J = 8.0 Hz), 129.1 (d, J = 3.4 Hz), 128.0, 122.0, 120.6, 120.1, 115.7 (d, J = 21.5 Hz), 109.8, 101.9, 31.2; ^{19}F NMR (282 MHz, Chloroform-*d*) δ -113.72 – -113.90 (m); IR (ATR, cm-1): 3026, 1738, 1495, 1464, 1366, 1217, 1155, 841, 787, 731; HRMS (ESI) calculated for $C_{15}H_{13}FN$ [M+H]$^+$: 226.1027; found: 226.1022.

1,3-dimethyl-2-(3-(trifluoromethyl)phenyl)-1H-indole (3t)

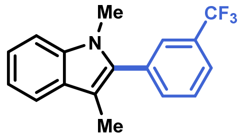

3t, 91% (1h)

Reaction conditions: 1.0 mmol scale, method B, 1 hour reaction time; Purification by flash chromatography on silica (petroleum ether Rf = 0.35) afforded 1,3-dimethyl-2-(3-(trifluoromethyl)phenyl)-1H-indole (263 mg, 91%) as a orange oil; ^{1}H NMR (400 MHz, Chloroform-d) δ 7.75 – 7.60 (m, 5H), 7.38 (d, J = 8.1 Hz, 1H), 7.33 (t, J = 7.3 Hz, 1H), 7.22 (t, J = 7.4 Hz, 1H), 3.65 (s, 3H), 2.33 (s, 3H); ^{13}C NMR (101 MHz, Chloroform-d) δ 137.6, 136.0, 134.0, 133.2, 131.1 (q, J = 32.4 Hz), 129.0, 128.5, 127.3 (q, J = 3.8 Hz), 124.6 (q, J = 3.8 Hz), 124.2 (q, J = 272.4 Hz), 122.5, 119.5, 119.2, 109.7, 109.5, 31.1, 9.4; ^{19}F NMR (376 MHz, Chloroform-d) δ -62.6; IR (ATR, cm^{-1}): 3057, 2916, 2860, 1614, 1589, 1415, 1361, 1321, 1310, 1231; HRMS (ESI) calculated for $C_{17}H_{15}F_3N$ [M+H]$^+$: 290.1151; found: 290.1147.

1,3-dimethyl-2-(4-nitrophenyl)-1H-indole (3u)

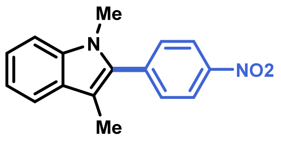

3u, 80% (16h)

Reaction conditions: 1.0 mmol scale, method B, 16 hours reaction time; Purification by flash chromatography on silica (2% ethyl acetate in petroleum ether Rf = 0.20) afforded 1,3-dimethyl-2-(4-nitrophenyl)-1H-indole (213 mg, 80%) as a orange solid; Mp. 150 °C; ^{1}H NMR (400 MHz, Chloroform-d) δ 8.37 (d, *J* = 8.7 Hz, 2H), 7.63 (d, *J* = 7.9 Hz, 1H), 7.58 (d, *J* = 8.7 Hz, 2H), 7.40 – 7.28 (m, 2H), 7.19 (t, *J* = 6.9 Hz, 1H), 3.66 (s, 3H), 2.33 (s, 3H); ^{13}C NMR (101 MHz, Chloroform-d) δ 147.1, 139.1, 138.2, 135.3, 131.2, 128.5, 123.8, 123.1, 119.9, 119.5, 111.1, 109.7, 31.5, 9.6; IR (ATR, cm^{-1}): 3072, 2932, 1593, 1506, 1468, 1362, 1337, 1236, 1109, 1070; HRMS (ESI) calculated for $C_{16}H_{15}N_2O_2$ [M+H]$^+$: 267.1128; found: 267.1124.

2-(2-chlorophenyl)-1-methyl-1H-indole (3v)

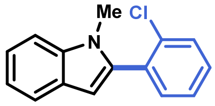

3v, 92% (2h)

Reaction conditions: 1.0 mmol scale, method B, 2 hours reaction time; Purification by flash chromatography on silica (1% ethyl acetate in petroleum ether Rf = 0.45) afforded 2-(2-chlorophenyl)-1-methyl-1H-indole (235 mg, 92%) as a orange solid; Mp. 75 °C;^{1}H NMR (400 MHz, Chloroform-*d*) δ 7.64 (d, *J* = 7.8 Hz, 1H), 7.56 (d, *J* = 6.9 Hz, 1H), 7.40 (ddd, *J* = 13.9, 6.7, 3.7 Hz, 4H), 7.29 (d, *J* = 7.8 Hz, 1H), 7.17 (t, *J* = 7.4 Hz, 1H), 3.53 (s, 3H), 2.20 (s, 3H); ^{13}C NMR (101 MHz, Chloroform-d) δ 137.1, 135.8, 134.9, 133.3, 131.6, 130.0, 129.9, 128.2, 126.8, 121.9, 119.1, 109.6, 109.3, 30.6, 9.3; IR (ART, cm-1): 3051, 2913, 1466, 1429, 1381, 1360, 1329, 1244, 1229, 1055; HRMS (ESI) calculated for $C_{16}H_{15}ClN$ [M+H]$^+$: 256.0888; found: 256.0883.

2-(3-iodophenyl)-1-methyl-1H-indole (3w)

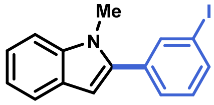

3w, 40% (1h)

Reaction conditions: 1.0 mmol scale, method B modified (2 mol% $Pd(OAc)_2$), 1 hour reaction time; Purification by flash chromatography on silica (2% ethyl acetate in petroleum ether Rf = 0.40) afforded 2-(3-iodophenyl)-1-methyl-1H-indole (132 mg, 40%) as a orange solid; Mp. 116 °C; ^{1}H NMR (400 MHz, Chloroform-*d*) δ 7.90 (t, *J* = 1.58 Hz, 1H), 7.74 (d, *J* = 7.94 Hz, 1H), 7.65 (d, *J* = 7.85 Hz, 1H), 7.48 (d, *J* = 7.73 Hz, 1H), 7.37 (d, *J* = 8.21 Hz, 1H), 7.29 (d, *J* = 7.13 Hz, 1H), 7.19 (dt, *J* = 17.41, 7.52 Hz, 2H), 6.58 (s, 1H), 3.75 (s, 3H); ^{13}C NMR (101 MHz, Chloroform-*d*) δ 139.8, 138.6, 138.2, 136.9, 135.1, 130.2, 128.6, 127.9,

122.2, 120.8, 120.2, 109.8, 102.5, 94.4, 31.3; IR (ATR, cm-1): 3048, 2928, 1722, 1591, 1557, 1533, 1466, 1429, 1381, 1339; HRMS (ESI) calculated for $C_{15}H_{13}IN$ $[M+H]^+$: 334.0087; found: 334.0083.

2-(4-bromophenyl)-1,3-dimethyl-1H-indole (3x)[16]

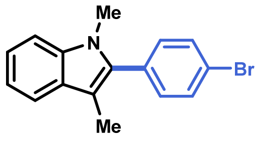

3x, 84% (16h)

Reaction conditions: 1.0 mmol scale, method B modified (2 mol% $Pd(OAc)_2$, 1.2 equiv. aryldiazonium salt), 16 hours reaction time; Purification by flash chromatography on silica (petroleum ether Rf = 0.35) afforded 2-(4-bromophenyl)-1,3-dimethyl-1H-indole (251 mg, 84%) as a orange solid; Mp. 128 °C; ^{1}H NMR (400 MHz, Chloroform-d) δ 7.65 – 7.55 (m, 3H), 7.31 (d, *J* = 8.1 Hz, 1H), 7.29 – 7.21 (m, 3H), 7.14 (t, *J* = 7.4 Hz, 1H), 3.58 (s, 3H), 2.25 (s, 3H); ^{13}C NMR (101 MHz, Chloroform-d) δ 137.5, 136.5, 132.3, 131.7, 131.2, 128.5, 122.2, 122.2, 119.4, 119.0, 109.4, 109.2, 31.1, 9.5; IR (ATR, cm-1): 3050, 2916, 2858, 1466, 1354, 1329, 1234, 1072, 1003; HRMS (ESI) calculated for $C_{16}H_{15}BrN$ $[M+H]^+$: 300.0383; found: 300.0379.

1-(4-(1-methyl-1H-indol-2-yl)phenyl)ethan-1-one (3ya)[17]

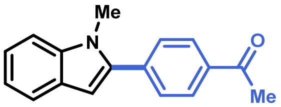

3ya, 58% (1h)

Reaction conditions: 1.0 mmol scale, method B, 1 hour reaction time; Purification by flash chromatography on silica (10% ethyl acetate in petroleum ether Rf = 0.20) afforded 1-(4-(1-methyl-1H-indol-2-yl)phenyl)ethan-1-one (144 mg, 58%) as a white solid; Mp. 129 °C; ^{1}H NMR (400 MHz, Chloroform-*d*) δ 8.07 (d, *J* = 8.3 Hz, 2H), 7.65 (dd, *J* = 11.9, 8.1 Hz, 3H), 7.39 (d, *J* = 8.2 Hz, 1H), 7.29 (t, *J* = 7.6 Hz, 1H), 7.17 (t, *J* = 7.4 Hz, 1H), 6.67 (s, 1H), 3.79 (s, 3H), 2.66 (s, 3H); ^{13}C NMR (101 MHz, Chloroform-*d*) δ 197.7, 140.4, 139.1, 137.6, 136.2, 129.3, 128.7, 128.0, 122.5, 120.9, 120.3, 109.9, 103.2, 31.6, 26.8; IR (ATR, cm^{-1}): 3051, 2945, 1676, 1600, 1465, 1408, 1354, 1340, 1263, 1184; HRMS (ESI) calculated for $C_{17}H_{16}NO$ $[M+H]^+$: 250.1227; found: 250.1221.

1-(4-(1,3-dimethyl-1H-indol-2-yl)phenyl)ethan-1-one (3y)

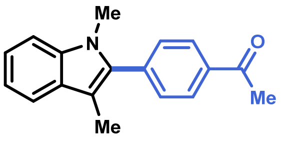

3y, 80% (3h)

Reaction conditions: 1.0 mmol scale, method B, 3 hours reaction time; Purification by flash chromatography on silica (10% ethyl acetate in petroleum ether Rf= 0.20) afforded 1-(4-(1,3-dimethyl-1H-indol-2-yl)phenyl)ethan-1-one (210 mg, 80%) as a

orange solid; Mp. 132 °C; ^{1}H NMR (400 MHz, Chloroform-*d*) δ 8.09 (d, *J* = 8.3 Hz, 2H), 7.62 (d, *J* = 7.8 Hz, 1H), 7.52 (d, *J* = 8.3 Hz, 2H), 7.35 (d, *J* = 8.1 Hz, 1H), 7.29 (t, *J* = 7.5 Hz, 1H), 7.18 (t, *J* = 7.4 Hz, 1H), 3.65 (s, 3H), 2.68 (s, 3H), 2.32 (s, 3H); ^{13}C NMR (101 MHz, Chloroform-*d*) δ 197.8, 137.9, 137.2, 136.5, 136.2, 130.8, 128.5, 128.5, 122.5, 119.6, 119.2, 110.0, 109.5, 31.3, 26.8, 9.6; IR (ATR, cm-1): 2980, 2916, 1676, 1606, 1467, 1355, 1329, 1258, 1247, 1238; HRMS (ESI) calculated for $C_{18}H_{18}NO$ $[M+H]^+$: 264.1383; found: 2641379.

2-(o-tolyl)-*1H*-indole (4a)[18]

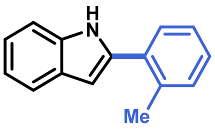

4a, 86% (1h)

Reaction conditions: 1.0 mmol scale, method B, 1 h reaction time; Purification by flash chromatography on silica (5% ethyl acetate in petroleum ether) afforded 2-(o-tolyl)-*1H*-indole (186 mg, 86%) as white solids; Mp. 84 °C[19]; ^{1}H NMR (400 MHz, Chloroform-d) δ 8.15 (s, 1H), 7.67 (d, J = 7.8 Hz, 1H), 7.49 (dd, J = 5.1, 3.7 Hz, 1H), 7.42 (d, J = 8.0 Hz, 1H), 7.36 – 7.28 (m, 3H), 7.22 (t, J = 7.5 Hz, 1H), 7.15 (t, J = 7.5 Hz, 1H), 6.63 (s, 1H), 2.52 (s, 3H); ^{13}C NMR (101 MHz, Chloroform-d) δ 137.5, 136.3, 136.2, 132.7, 131.2, 129.1, 129.0, 128.1, 126.2, 122.2, 120.6, 120.2, 110.9, 103.1, 21.2; IR (ATR, cm-1): 3399, 3049, 1485, 1454, 1344, 1302, 1119, 1057, 799, 745, 719; HRMS (ESI) calculated for $C_{15}H_{14}N$ $[M+H]^+$: 208.1121; found: 208.1119.

2-(2,4-dimethylphenyl)-*1H*-indole (4b)

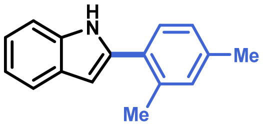

4b, 74% (30 min)

Reaction conditions: 1.0 mmol scale, method B, 30 min reaction time; Purification by flash chromatography on silica (5% ethyl acetate in petroleum ether) afforded 2-(2,4-dimethylphenyl)-*1H*-indole (163 mg, 74%) as white solids; Mp. 130 °C; ^{1}H NMR (400 MHz, Chloroform-d) δ 8.11 (s, 1H), 7.65 (d, J = 8.3 Hz, 1H), 7.43 – 7.36 (m, 2H), 7.23 – 7.08 (m, 4H), 6.60 (s, 1H), 2.48 (s, 3H), 2.38 (s, 3H); ^{13}C NMR (101 MHz, Chloroform-d) δ 137.9, 137.7, 136.1, 136.0, 132.0, 129.8, 129.0, 129.0, 126.9, 122.0, 120.5, 120.1, 110.8, 102.8, 21.3, 21.1; IR (ATR, cm-1): 3393, 2916, 1485, 1456, 1346, 1302, 1231, 907, 795, 743, 723, 675; HRMS (ESI) calculated for $C_{16}H_{16}N$ $[M+H]^+$: 222.1278; found: 222.1273.

2-mesityl-*1H*-indole (4c –C-2)[20]

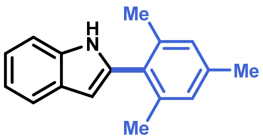

4c, 19% (5h)
[C2]

Reaction conditions: 1.0 mmol scale, slightly modified method B (1.2 equiv. aryldiazonium salt at 40 °C), 5 h reaction time; Purification by flash chromatography on silica (5% ethyl acetate in petroleum ether) afforded 2-mesityl-*1H*-indole (45 mg, 19%) as white solids; Mp. 159 °C; ^{1}H NMR (400 MHz, Chloroform-d) δ 7.86 (s, 1H), 7.67 (d, J = 7.7 Hz, 1H), 7.40 (d, J = 8.0 Hz, 1H), 7.21 (t, J = 7.5 Hz, 1H), 7.16 (t, J = 7.5 Hz, 1H), 6.99 (s, 2H), 6.40 (s, 1H), 2.37 (s, 3H), 2.17 (s, 6H); ^{13}C NMR (101 MHz, Chloroform-d) δ 138.4, 138.3, 136.4, 136.0, 130.2, 129.0, 128.3, 121.5, 120.4, 119.9, 110.8, 102.7, 21.3, 20.6; IR (ATR, cm-1): 3385, 2914, 1456, 1342, 1290, 1034, 851, 783, 756, 702; HRMS (ESI) calculated for $C_{17}H_{18}N$ [M+H]$^+$: 236.1434; found: 236.1431.

3-mesityl-*1H*-indole (4c – C-3)[20]

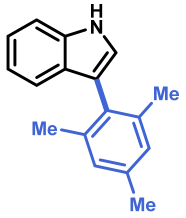

4c, 63% (5h)
[C3]

Reaction conditions: 1.0 mmol scale, slightly modified method B (1.2 equiv. aryldiazonium salt at 40 °C), 5 h reaction time; Purification by flash chromatography on silica (5% ethyl acetate in petroleum ether) afforded 3-mesityl-*1H*-indole (148 mg, 63%) as colorless oil; ^{1}H NMR (399 MHz, Chloroform-*d*) δ 8.18 (s, 1H), 7.44 (d, *J* = 8.1 Hz, 1H), 7.29 – 7.20 (m, 2H), 7.09 (t, *J* = 7.0 Hz, 1H), 7.04 (d, *J* = 2.3 Hz, 1H), 7.00 (s, 2H), 2.37 (s, 3H), 2.07 (s, 6H); ^{13}C NMR (101 MHz, Chloroform-d) δ 138.6, 136.7, 136.2, 131.0, 128.1, 127.5, 122.6, 122.1, 120.1, 119.7, 116.0, 111.2, 21.2, 20.9; IR (ATR, cm-1): 3410, 2916, 1558, 1454, 1341, 1250, 1236, 1092, 853, 743; HRMS (ESI) calculated for $C_{17}H_{18}N$ [M+H]$^+$: 236.1434; found: 236.1431.

2-(4-methoxy-2-methylphenyl)-*1H*-indole (4d)[21]

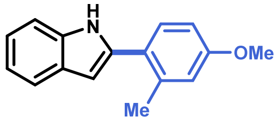

4d, 72% (3h)

Reaction conditions: 1.0 mmol scale, slightly modified method B (1.2 equiv. aryldiazonium salt at 40 °C), 3 h reaction time; Purification by flash chromatography on silica (10% ethyl acetate in petroleum ether) afforded 2-(4-methoxy-2-methylphenyl)-*1H*-indole (170 mg, 72%) as white solids; Mp. 132 °C; ^{1}H NMR (400 MHz, Chloroform-d) δ 8.08 (s, 1H), 7.64 (d, J = 8.2 Hz, 1H), 7.40 (d, J = 8.5 Hz, 2H), 7.20 (td, J = 7.5, 1.3 Hz, 1H), 7.14 (td, J = 7.3, 1.1 Hz, 1H), 6.89 – 6.81 (m, 2H), 6.55 (dd, J = 2.1, 0.9 Hz, 1H), 3.85 (s, 3H), 2.48 (s, 3H); ^{13}C NMR (101 MHz, Chloroform-d) δ 159.3, 137.9, 137.5, 136.1, 130.3, 129.0, 125.5, 121.8, 120.4, 120.1, 116.5, 111.5, 110.8, 102.4, 55.4, 21.4; IR (ATR, cm-1): 3397, 1605, 1570, 1493, 1454, 1346, 1296, 1246, 1229, 1161; HRMS (ESI) calculated for $C_{16}H_{16}NO$ [M+H]$^+$: 238.1227; found: 238.1226.

2-(4-methoxyphenyl)-3-methyl-1*H*-indole (4e)[22]

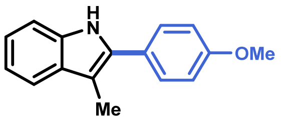

4e, 76% (4h)

Reaction conditions: 1.0 mmol scale, method B, 4 h reaction time; Purification by flash chromatography on silica (5% ethyl acetate in petroleum ether) afforded 2-(4-methoxyphenyl)-3-methyl-*1H*-indole (180 mg, 76%) as off-white solids; Mp. 126 °C; ^{1}H NMR (399 MHz, Chloroform-d) δ 7.95 (s, 1H), 7.60 (d, *J* = 7.7 Hz, 1H), 7.55 – 7.48 (m, 2H), 7.35 (d, *J* = 7.7 Hz, 1H), 7.24 – 7.12 (m, 2H), 7.06 – 6.98 (m, 2H), 3.88 (s, 3H), 2.45 (s, 3H); ^{13}C NMR (100 MHz, Chloroform-d) δ 159.1, 135.8, 134.1, 130.2, 129.1, 126.0, 122.1, 119.6, 118.9, 114.4, 110.7, 107.9, 55.5, 9.7; IR (ATR, cm-1): 3412, 3051, 2914, 2860, 2835, 1609, 1508, 1244, 1036, 744; HRMS (ESI) calculated for $C_{16}H_{16}NO$ $[M+H]^+$: 238.1227; found: 238.1229.

6-fluoro-1-methyl-2-phenyl-indole (5a)[23]

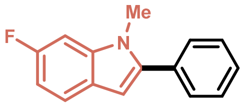

5a, 89% (2h)

Reaction conditions: 1.0 mmol scale, method A, 2h reaction time; Purification by flash chromatography on silica (2% ethyl acetate in petroleum ether) afforded 6-fluoro-1-methyl-2-phenyl-indole (200 mg, 89%) as white solids; Mp. 159 °C; ^{1}H NMR (400 MHz, Chloroform-d) δ 7.57 (dd, J = 8.6, 5.4 Hz, 1H), 7.55 – 7.47 (m, 3H), 7.45 (d, J = 6.8 Hz, 1H), 7.07 (dd, J = 9.9, 2.2 Hz, 1H), 6.95 (ddd, J = 9.7, 8.6, 2.3 Hz, 1H), 6.57 (s, 1H), 3.72 (s, 3H); ^{13}C NMR (101 MHz, Chloroform-d) δ 159.9 (d, J = 237.6 Hz), 142.2 (d, J = 4.0 Hz), 138.56 (d, J = 12.1 Hz), 132.7, 129.4, 128.7, 128.1, 124.5, 121.3 (d, J = 10.1 Hz), 108.6 (d, J = 24.4 Hz), 101.7, 96.2 (d, J = 26.1 Hz), 31.5; ^{19}F NMR (376 MHz, Chloroform-d) δ -120.6 (td, J = 9.5, 5.4 Hz); IR (ATR, cm-1): 1624, 1489, 1464, 1354, 1186, 1159, 1082, 957, 827, 766, 702; HRMS (ESI) calculated for $C_{15}H_{13}FN$ $[M+H]^+$: 226.1027; found: 226.1029.

6-fluoro-2-(4-fluorophenyl)-1-methyl-1H-indole (5b)

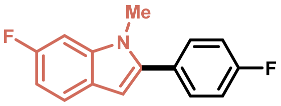

5b, 74% (1h)

Reaction conditions: 1.0 mmol scale, method A, 1h reaction time; Purification by flash chromatography on silica (petroleum ether Rf = 0.35) afforded 6-fluoro-2-(4-fluorophenyl)-1-methyl-1H-indole (180 mg, 74%) as white solids; Mp. 124 °C; ^{1}H NMR (399 MHz, Chloroform-d) δ 7.53 (dd, *J* = 8.6, 5.4 Hz, 1H), 7.48 – 7.42 (m, 2H), 7.22 – 7.12 (m, 2H), 7.03 (dd, *J* = 9.9, 2.2 Hz, 1H), 6.91 (ddd, *J* = 9.6, 8.6, 2.3 Hz, 1H), 6.50 (s, 1H), 3.67 (s, 3H); ^{13}C NMR (101 MHz, Chloroform-d) δ 164.0, 161.6, 161.2, 158.8, 141.1 (d, *J* = 3.7 Hz), 138.5 (d, *J* = 11.9 Hz), 131.1 (d, *J* = 8.1 Hz), 128.8 (d, *J* = 3.5 Hz), 124.4, 121.3 (d, *J* = 10.0 Hz), 115.8 (d, *J* = 21.6 Hz), 108.7 (d, *J* = 24.4 Hz), 101.8, 96.2 (d, *J* = 26.2 Hz), 31.4; ^{19}F NMR (376 MHz, Chloroform-d) δ -113.65 (ddd, *J* = 14.0,

8.5, 5.3 Hz), -120.39 (td, J = 10.0, 5.4 Hz); IR (ATR, cm-1): 3065, 2955, 2920, 1620, 1583, 1549, 1495, 1466, 1427, 1358; HRMS (ESI) calculated for $C_{15}H_{12}F_2N$ $[M+H]^+$: 244.0933; found: 244.0928.

5-methoxy-1-methyl-2-phenyl-indole (5c)[24]

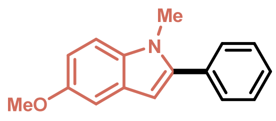

5c, 62% (2h)

Reaction conditions: 1.0 mmol scale, method A, 2h reaction time; Purification by flash chromatography on silica (2% ethyl acetate in petroleum ether) afforded 5-methoxy-1-methyl-2-phenyl-indole (146 mg, 62%) as white solids; Mp. 127 °C; ^{1}H NMR (400 MHz, Chloroform-d) δ 7.55 – 7.46 (m, 4H), 7.45 – 7.39 (m, 1H), 7.28 (d, J = 8.8 Hz, 1H), 7.14 (d, J = 2.4 Hz, 1H), 6.95 (dd, J = 8.8, 2.5 Hz, 1H), 6.53 (s, 1H), 3.90 (s, 3H), 3.74 (s, 3H); ^{13}C NMR (101 MHz, Chloroform-d) δ 154.5, 142.3, 133.9, 133.0, 129.4, 128.6, 128.4, 127.9, 112.0, 110.5, 102.3, 101.4, 56.1, 31.4; IR (ATR, cm-1): 1734, 1474, 1348, 1219, 1180, 1136, 1030, 843, 800, 766, 704; HRMS (ESI) calculated for $C_{17}H_{18}FN$ $[M+H]^+$: 236.1434; found: 236.1432.

methyl 1-methyl-2-phenyl-1H-indole-6-carboxylate (5d)[25]

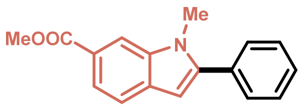

5d, 22% (16h)

Reaction conditions: 1.0 mmol scale, method B modified (1.2 equiv. aryldiazonium salt at 40 °C), 16h reaction time; Purification by flash chromatography on silica (10% ethyl acetate in petroleum ether Rf = 0.30) afforded methyl 1-methyl-2-phenyl-1H-indole-6-carboxylate (59 mg, 22%) as orange solids; Mp. 100 °C; ^{1}H NMR (400 MHz, Chloroform-*d*) δ 8.15 (s, 1H), 7.85 (d, J = 8.3 Hz, 1H), 7.64 (d, J = 8.3 Hz, 1H), 7.56 – 7.41 (m, 5H), 6.60 (s, 1H), 3.97 (s, 3H), 3.82 (s, 3H); ^{13}C NMR (101 MHz, Chloroform-*d*) δ 168.4, 145.0, 137.8, 132.3, 131.8, 129.5, 128.8, 128.6, 123.2, 121.1, 120.1, 112.2, 102.2, 52.1, 31.5; IR (ATR, cm-1): 3073, 2949, 1705, 1609, 1464, 1435, 1337, 1302, 1250, 1227; HRMS (ESI) calculated for $C_{17}H_{16}NO_2$ $[M+H]^+$: 266.1176; found: 266.1173.

1,2-dimethyl-3-(phenyldiazenyl)-indole (1bb)

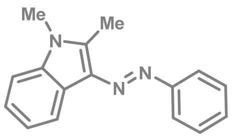

1bb, 93% (5h)

Reaction conditions: 1.0 mmol scale, method A, 5h reaction time; Purification by flash chromatography on silica (15% ethyl acetate in petroleum ether) afforded methyl 1,2-dimethyl-3-(phenyldiazenyl)-indole (231 mg, 93%) as a brown solid;

Mp. 125 ^{1}H NMR (399 MHz, DMSO-d6) δ 8.45 – 8.38 (m, 1H), 7.85 – 7.78 (m, 2H), 7.59 – 7.46 (m, 3H), 7.36 (t, J = 7.2 Hz, 1H), 7.26 (p, J = 7.2 Hz, 2H), 3.79 (s, 3H), 2.80 (s, 3H); ^{13}C NMR (100 MHz, DMSO-d6) δ 153.5, 146.6, 136.8, 131.4, 129.1, 128.3, 123.2, 122.7, 121.6, 121.1, 118.2, 110.0, 30.0, 9.9; IR (ATR, cm-1): 3055, 2924, 1534, 1446, 1387, 1059; HRMS (ESI) calculated for $C_{16}H_{16}N_3$ $[M+H]^+$: 250.1339; found: 250.1340.

2-phenylbenzofuran (6a)[26]

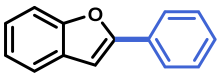

6a, 70% (6h)

Reaction conditions: 1.0 mmol scale, method C, 6 h reaction time; Purification by flash chromatography on silica (petroleum ether) afforded ethyl 2-phenylbenzofuran (136 mg, 70%) as white solids; Mp. 120 °C; ^{1}H NMR (400 MHz, Chloroform-d) δ 7.88 (d, *J* = 7.5 Hz, 2H), 7.60 (d, *J* = 7.5 Hz, 1H), 7.54 (d, *J* = 7.5 Hz, 1H), 7.46 (t, *J* = 7.5 Hz, 2H), 7.36 (t, *J* = 7.5 Hz, 1H), 7.33 – 7.21 (m, 2H), 7.04 (s, 1H); ^{13}C NMR (101 MHz, Chloroform-d) δ 156.1, 155.1, 130.7, 129.4, 129.0, 128.7, 125.1, 124.4, 123.1, 121.1, 111.4, 101.5; IR (ATR, cm-1): 3051, 3034, 2922, 2853, 1562, 1483, 1470, 1454, 1441, 1350, 1306; HRMS (ESI) calculated for $C_{14}H_{11}O$ $[M+H]^+$: 195.0805; found: 195.0803.

2-(3-chloro-4-methoxyphenyl)benzofuran (6b)

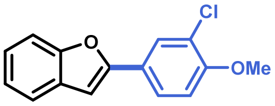

6b, 66% (16h)

Reaction conditions: 1.0 mmol scale, method C, 16 h reaction time; Purification by flash chromatography on silica (5% ethyl acetate in petroleum ether) afforded ethyl 2-(3-chloro-4-methoxyphenyl)benzofuran (171 mg, 66%) as white solids; Mp. 150 °C; ^{1}H NMR (399 MHz, Chloroform-d) δ 7.88 (d, *J* = 2.1 Hz, 1H), 7.72 (dd, *J* = 8.6, 2.1 Hz, 1H), 7.58 – 7.55 (m, 1H), 7.51 (d, *J* = 8.1 Hz, 1H), 7.32 – 7.18 (m, 2H), 6.99 (d, *J* = 8.6 Hz, 1H), 6.91 (s, 1H), 3.96 (s, 3H); ^{13}C NMR (100 MHz, Chloroform-d) δ 155.37 , 154.95 , 154.74 , 129.40 , 127.06 , 124.63 , 124.38 , 124.36 , 123.21 , 123.18 , 120.97 , 112.37 , 111.26 , 100.81 , 56.46; IR (ATR, cm-1): 3005, 2941, 2845, 1605, 1495, 1450, 1439, 1263, 1061, 804; HRMS (ESI) calculated for $C_{15}H_{12}ClO_2$ $[M+H]^+$: 259.0520; found: 259.0514.

2-(4-fluorophenyl)benzofuran (6c)[26]

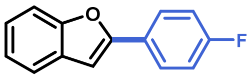

6c, 71% (4h)

Reaction conditions: 1.0 mmol scale, method C, 4 h reaction time; Purification by flash chromatography on silica (petroleum ether) afforded 2-(4-fluorophenyl)benzofuran (151 mg, 71%) as white solids; Mp. 120 °C; ^{1}H NMR (400 MHz, Chloroform-d) δ 7.88 – 7.81 (m, 2H), 7.62 – 7.56 (m, 1H), 7.56 – 7.49 (m, 1H), 7.32 – 7.21 (m, 2H), 7.19 – 7.10 (m, 2H), 6.97 – 6.95 (m, 1H); ^{13}C NMR (100 MHz, Chloroform-d) δ 163.1 (d, *J* = 247.7 Hz), 155.2, 155.0, 129.4, 127.0 , 126.9, 124.5, 123.2, 121.1, 116.1 (d, *J* = 21.8 Hz), 111.3, 101.2 (d, *J* = 1.4 Hz); ^{19}F NMR (376 MHz, Chloroform-d) δ -112.4 (m); IR (ATR, cm-1): 3046, 1599, 1499, 1450, 1223, 1206, 1157, 1098, 839, 800; HRMS (ESI) calculated for $C_{14}H_{10}FO$ [M+H]$^+$: 213.0710; found: 213.0717.

2-(4'-(trifluoromethyl)phenyl)benzofuran (6d)[27]

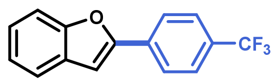

6d, 82% (16h)

Reaction conditions: 1.0 mmol scale, method C, 16 h reaction time; Purification by flash chromatography on silica (petroleum ether) afforded 2-(4'-(trifluoromethyl)phenyl)benzofuran (215 mg, 82%) as white solids; Mp. 164 °C; ^{1}H NMR (500 MHz, Chloroform-d) δ 7.95 (d, *J* = 7.8 Hz, 2H), 7.69 (d, *J* = 7.8 Hz, 2H), 7.61 (ddd, *J* = 7.8, 1.4, 0.7 Hz, 1H), 7.54 (ddd, *J* = 7.8, 1.4, 0.7 Hz, 1H), 7.33 (ddd, *J* = 8.0, 7.5, 1.3 Hz, 1H), 7.30 – 7.21 (m, 1H), 7.12 (d, *J* = 0.7 Hz, 1H); ^{13}C NMR (101 MHz, Chloroform-d) δ 155.3, 154.4, 133.9, 130.3 (q, *J* = 31.3 Hz), 129.0, 126.0 (q, *J* = 3.8 Hz), 125.3 , 125.2, 124.2 (q, *J* = 271.9 Hz), 123.5, 121.5, 111.6, 103.4; ^{19}F NMR (282 MHz, Chloroform-d) δ -62.6; IR (ATR, cm-1): 1738, 1373, 1323, 1229, 1163, 1107, 1069, 1013, 841, 810, 748.

2-(3-(trifluoromethyl)phenyl)benzofuran (6e)[28]

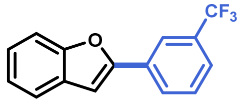

6e, 81% (30 min)

Reaction conditions: 1.0 mmol scale, method C, 30 min reaction time; Purification by flash chromatography on silica (petroleum ether) afforded 2-(3-(trifluoromethyl)phenyl)benzofuran (212 mg, 81%) as off-white solids; Mp. 67 °C; ^{1}H NMR (400 MHz, Chloroform-d) δ 8.12 (s, 1H), 8.03 (d, *J* = 7.5 Hz, 1H), 7.64 – 7.53 (m, 4H), 7.33 (ddd, *J* = 8.4, 7.3, 1.3 Hz, 1H), 7.26 (ddd, *J* = 7.7, 1.1 Hz, 1H), 7.13 (d, *J* = 0.8 Hz, 1H); ^{13}C NMR (100 MHz, Chloroform-d) δ 155.1, 154.3, 131.5 (q, J = 32 Hz), 131.4, 129.5, 129.0, 128.0, 125.1, 125.1 (q, J = 4 Hz), 123.4, 122.8 (q, J = 272 Hz), 121.77 (q, J = 4 Hz), 121.4, 111.5, 102.8; ^{19}F NMR (376 MHz, Chloroform-d) δ -62.88; IR (ATR, cm-1): 3109, 3036, 2928, 1726, 1616, 1601, 1450, 1329, 1273, 1107.

2-(4-(trifluoromethoxyphenyl) benzofuran (6f)[29]

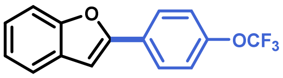

6f, 60% (2h)

Reaction conditions: 1.0 mmol scale, method C, 2 h reaction time; Purification by flash chromatography on silica (petroleum ether) afforded 2-(4-(trifluoromethoxyphenyl) benzofuran (167 mg, 60%) as white solids; Mp. 120 °C; ^{1}H NMR (500 MHz, Chloroform-d) δ 7.93 – 7.89 (m, 2H), 7.63 (ddd, *J* = 7.6, 1.4, 0.6 Hz, 1H), 7.58 – 7.55 (m, 1H), 7.39 – 7.24 (m, 4H), 7.05 (d, *J* = 0.9 Hz, 1H); ^{13}C NMR (101 MHz, Chloroform-d) δ 155.2, 154.7, 149.4, 129.4, 129.2, 126.5, 124.8, 123.3, 121.9, 121.5, 121.3, 119.4, 111.4, 102.1; ^{19}F NMR (376 MHz, Chloroform-d) δ -57.8; IR (ATR, cm-1): 3049, 2930, 1570, 1499, 1450, 1300, 1206, 1153, 804, 752.

2-(2,3-dichlorophenyl)benzofuran (6g)

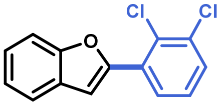

6g, 68% (3h)

Reaction conditions: 1.0 mmol scale, method C, 3 h reaction time; Purification by flash chromatography on silica (petroleum ether) afforded 2-(2,3-dichlorophenyl)benzofuran (178 mg, 68%) as white solids; Mp. 71 °C; ^{1}H NMR (399 MHz, Chloroform-d) δ 7.97 (dd, *J* = 7.9, 1.5 Hz, 1H), 7.68 – 7.63 (m, 1H), 7.57 – 7.52 (m, 2H), 7.48 (dd, *J* = 7.9, 1.5 Hz, 1H), 7.40 – 7.23 (m, 3H); ^{13}C NMR (100 MHz, Chloroform-d) δ 154.4, 151.6, 134.6, 131.4, 130.1, 129.8, 129.0, 127.5, 125.5, 123.3, 121.8, 111.3, 108.4; IR (ATR, cm-1): 3063, 3040, 1582, 1549, 1439, 1414, 1344, 1184, 1032, 781.

2-(3,5-dichlorophenyl)benzofuran (6h)[30]

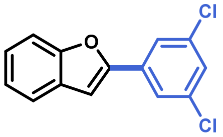

6h, 92% (1h)

Reaction conditions: 1.0 mmol scale, method C, 1 h reaction time; Purification by flash chromatography on silica (petroleum ether) afforded 2-(3,5-dichlorophenyl)benzofuran (242 mg, 92%) as bright yellow solids; Mp. 103 °C; ^{1}H NMR (400 MHz, Chloroform-d) δ 7.73 (d, *J* = 1.9 Hz, 2H), 7.63 – 7.59 (m, 1H), 7.55 – 7.50 (m, 1H), 7.37 – 7.30 (m, 2H), 7.30 – 7.23 (m, 1H), 7.07 (d, *J* = 0.8 Hz, 1H); ^{13}C NMR (100 MHz, Chloroform-d) δ 155.2, 153.1, 135.7, 133.4, 128.8, 128.4, 125.5, 123.5, 123.3, 121.6, 111.6, 103.6; IR (ATR, cm-1): 3069, 2922, 1599, 1578, 1552, 1454, 1412, 1260, 1121, 795.

3-(3-(trifluoromethyl)phenyl)benzo[*b*]thiophene (7a)[31]

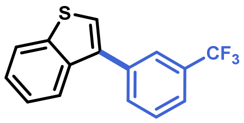

7a, 80%[b] (16h)
[C2:C3 4:96]

Reaction conditions: 1.0 mmol scale, method D, 16 h reaction time; Purification by flash chromatography on silica (petroleum ether) afforded 3-(3-(trifluoromethyl)phenyl)benzo[*b*]thiophene (222 mg, 80% [C-2:C-3 4:96]) as a colorless oil; ^{1}H NMR (399 MHz, Chloroform-d) δ 7.97 – 7.93 (m, 1H), 7.90 – 7.85 (m, 2H), 7.78 (d, *J* = 7.7 Hz, 1H), 7.69 (d, *J* = 7.7 Hz, 1H), 7.62 (t, *J* = 7.7 Hz, 1H), 7.48 (s, 1H), 7.46 – 7.42 (m, 2H); ^{13}C NMR (100 MHz, Chloroform-d) δ 140.9, 137.6, 136.9, 136.7, 132.1, 131.4 (q, *J* = 32.0 Hz), 129.4, 125.6 (q, *J* = 4.0 Hz), 124.9, 124.8, 124.7, 124.4 (q, *J* = 4.0 Hz), 124.3 (q, *J* = 273.0 Hz), 123.2, 122.6; ^{19}F NMR (376 MHz, Chloroform-d) δ -62.6 (s); IR (ATR, cm-1): 3061, 2930, 1431, 1319, 1312, 1163, 1119, 1094, 1072, 758.

3-(4-(trifluoromethoxy)phenyl)benzo[*b*]thiophene (7b)

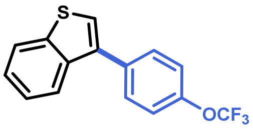

7b, 73%[b]
(16h)
[C2:C3 2:98]

Reaction conditions: 1.0 mmol scale, method D, 16 h reaction time; Purification by flash chromatography on silica (petroleum ether) afforded 3-(4-(trifluoromethoxy)phenyl)benzo[*b*]thiophene (215 mg, 73%, [C-2:C-3 2:98]) as a yellow oil; ^{1}H NMR (399 MHz, Chloroform-d) δ 7.98 – 7.92 (m, 1H), 7.91 – 7.86 (m, 1H), 7.62 (d, *J* = 8.6 Hz, 2H), 7.49 – 7.40 (m, 3H), 7.36 (d, *J* = 8.6 Hz, 2H); ^{13}C NMR (100 MHz, Chloroform-d) δ 148.8, 140.9, 137.8, 136.8, 134.9, 130.2, 124.8, 124.7, 124.2, 123.2, 122.8, 121.4, 120.7 (q, *J* = 257.2 Hz); ^{19}F NMR (376 MHz, Chloroform-d) δ -57.8; IR (ATR, cm-1): 3061, 2930, 1431, 11319, 1312, 1163, 1119, 1094, 1072, 758.

methyl 2-(5-methylbenzofuran-2-yl)benzoate (8a)[32]

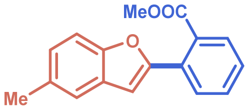

8a, 70% (2h)

Reaction conditions: 1.0 mmol scale, slightly modified method C (1.0 mol% Pd at 40 °C, no reflux work up), 2h reaction time; Purification by flash chromatography on silica (5% ethyl acetate in petroleum ether) afforded methyl 2-(5-methylbenzofuran-2-yl)benzoate (186 mg, 70%) as a yellow oil; ^{1}H NMR (399 MHz, Chloroform-d) δ 7.78 – 7.69 (m, 2H), 7.55 (td, J = 7.6, 1.3 Hz, 1H), 7.44 (td, J = 7.6, 1.3 Hz, 1H), 7.41 – 7.36 (m, 2H), 7.12 (dd, J = 8.4, 1.4 Hz, 1H), 6.87 (s, 1H), 3.82 (s, 3H), 2.46 (s, 3H); ^{13}C NMR (100 MHz, Chloroform-d) δ 169.6, 154.8, 153.7, 132.5, 131.1, 131.0, 129.8, 129.4, 129.1, 129.0, 128.6,

126.0, 121.1, 110.7, 104.3, 52.6, 21.5; IR (ATR, cm-1): 2949, 2920, 1726, 1449, 1289, 1265, 1123, 1090, 918, 754; HRMS (ESI) calculated for $C_{17}H_{15}O_3$ $[M+H]^+$: 267.1016; found: 267.1016.

8. References

(1) Kalyani, D.; McMurtrey, K. B.; Neufeldt, S. R.; Sanford, M. S. *J. Am. Chem. Soc.* **2011**, *133*, 18566.
(2) Baxter, R. D.; Sale, D.; Engle, K. M.; Yu, J. Q.; Blackmond, D. G. *J. Am. Chem. Soc.* **2012**, *134*, 4600.
(3) Frisch, M. J.*et al.*; Gaussian 09, Revision D.01, Gaussian, Inc.: Wallingford CT, 2009.
(4) Erb, W.; Hellal, A.; Albini, M.; Rouden, J.; Blanchet, J. *Chem. - Eur. J.* **2014**, *20*, 6608.
(5) Sun, N.; Huang, P.; Wang, Y.; Mo, W.; Hu, B.; Shen, Z.; Hu, X. *Tetrahedron* **2015**, *71*, 4835.
(6) Cody, W. L.; Holsworth, D. D.; Powell, N. A.; Jalaie, M.; Zhang, E.; Wang, W.; Samas, B.; Bryant, J.; Ostroski, R.; Ryan, M. J.; Edmunds, J. J. *Bioorg. Med. Chem.* **2005**, *13*, 59.
(7) Deprez, N. R.; Kalyani, D.; Krause, A.; Sanford, M. S. *J. Am. Chem. Soc.* **2006**, *128*, 4972.
(8) Chen, Y.-y.; Chen, J.-h.; Zhang, N.-n.; Ye, L.-m.; Zhang, X.-J.; Yan, M. *Tetrahedron Lett.* **2015**, *56*, 478.
(9) So, C. M.; Chow, W. K.; Choy, P. Y.; Lau, C. P.; Kwong, F. Y. *Chem. - Eur. J.* **2010**, *16*, 7996.
(10) Zou, Y.; Yue, G.; Xu, J.; Zhou, J. *Eur. J. Org. Chem.* **2014**, *2014*, 5901.
(11) Zhang, L.; Li, P.; Liu, C.; Yang, J.; Wang, M.; Wang, L. *Cat. Sci. Technol.* **2014**, *4*, 1979.
(12) Shi, Z.; Ding, S.; Cui, Y.; Jiao, N. *Angew. Chem. Int. Ed.* **2009**, *48*, 7895.
(13) Huang, Y.; Lin, Z.; Cao, R. *Chem. - Eur. J.* **2011**, *17*, 12706.
(14) Nandi, D.; Jhou, Y.-M.; Lee, J.-Y.; Kuo, B.-C.; Liu, C.-Y.; Huang, P.-W.; Lee, H. M. *J. Org. Chem.* **2012**, *77*, 9384.
(15) Lebrasseur, N.; Larrosa, I. *J. Am. Chem. Soc.* **2008**, *130*, 2926.
(16) Wei, W.-t.; Dong, X.-j.; Nie, S.-z.; Chen, Y.-y.; Zhang, X.-j.; Yan, M. *Org. Lett.* **2013**, *15*, 6018.
(17) Yanagisawa, S.; Sudo, T.; Noyori, R.; Itami, K. *J. Am. Chem. Soc.* **2006**, *128*, 11748.
(18) Fang, Y.-Q.; Lautens, M. *J. Org. Chem.* **2008**, *73*, 538.
(19) Jadhav, J.; Gaikwad, V.; Kurane, R.; Salunkhe, R.; Rashinkar, G. *Synlett* **2012**, DOI: 10.1055/s-0032-1317320, 2511.
(20) Alsabeh, P. G.; Lundgren, R. J.; Longobardi, L. E.; Stradiotto, M. *Chem. Commun.* **2011**, *47*, 6936.
(21) Fang, Y.-Q.; Lautens, M. *The Journal of Organic Chemistry* **2008**, *73*, 538.
(22) Nguyen, Q.; Nguyen, T.; Driver, T. G. *J. Am. Chem. Soc.* **2013**, *135*, 620.
(23) Lavery, C. B.; McDonald, R.; Stradiotto, M. *Chem. Commun.* **2012**, *48*, 7277.
(24) Liang, Z.; Yao, B.; Zhang, Y. *Org. Lett.* **2010**, *12*, 3185.
(25) Lane, B. S.; Brown, M. A.; Sames, D. *J. Am. Chem. Soc.* **2005**, *127*, 8050.
(26) Gao, H.; Xu, Q. L.; Keene, C.; Kurti, L. *Chem. - Eur. J.* **2014**, *20*, 8883.
(27) Denmark, S. E.; Smith, R. C.; Chang, W.-T. T.; Muhuhi, J. M. *J. Am. Chem. Soc.* **2009**, *131*, 3104.
(28) Fürstner, A.; Davies, P. W. *J. Am. Chem. Soc.* **2005**, *127*, 15024.
(29) Moure, M. J.; SanMartin, R.; Dominguez, E. *Adv. Synth. Catal.* **2014**, *356*, 2070.
(30) Loukotova, L.; Yuan, K. D.; Doucet, H. *ChemCatChem* **2014**, *6*, 1303.
(31) Tang, D. T.; Collins, K. D.; Glorius, F. *J. Am. Chem. Soc.* **2013**, *135*, 7450.
(32) Judd, D. B.; Dowle, M. D.; Middlemiss, D.; Scopes, D. I. C.; Ross, B. C.; Jack, T. I.; Pass, M.; Tranquillini, E.; Hobson, J. E. *J. Med. Chem.* **1994**, *37*, 3108.

9. DFT calculations: XYZ and thermal Data

Int1

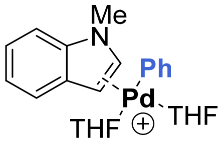

Pd	0.401845000000	-0.081752000000	-0.049681000000
C	-2.123926000000	-1.541797000000	-0.795201000000
C	-2.772787000000	-1.392679000000	0.443846000000
C	-4.104267000000	-0.994782000000	0.548681000000
C	-4.789415000000	-0.773771000000	-0.638393000000
C	-4.166985000000	-0.941578000000	-1.886867000000
C	-2.835989000000	-1.324123000000	-1.976954000000
C	-0.738307000000	-1.888858000000	-0.509997000000
C	-0.661362000000	-1.984974000000	0.898164000000
H	-4.593227000000	-0.874374000000	1.510169000000
H	-5.833290000000	-0.478913000000	-0.600910000000
H	-4.740189000000	-0.774509000000	-2.792808000000
H	-2.357184000000	-1.451088000000	-2.943301000000
H	0.128938000000	-2.416673000000	1.498773000000
N	-1.862427000000	-1.684302000000	1.455929000000
C	-2.145914000000	-1.612773000000	2.875268000000
H	-2.306117000000	-0.577043000000	3.191376000000
H	-3.038100000000	-2.201043000000	3.104931000000
H	-1.301056000000	-2.025880000000	3.428151000000
H	-0.084240000000	-2.432503000000	-1.181633000000
O	-1.349534000000	1.273550000000	0.025559000000
C	-2.968898000000	2.779973000000	-0.735440000000
C	-2.595566000000	3.160214000000	0.701599000000
C	-1.754608000000	1.973567000000	-1.165841000000
C	-1.896267000000	1.898079000000	1.199717000000
H	-3.143872000000	3.646518000000	-1.376467000000
H	-1.967216000000	1.222863000000	-1.928995000000
H	-3.459275000000	3.422269000000	1.316420000000
H	-2.604978000000	1.193761000000	1.649209000000
H	-1.903025000000	4.008276000000	0.703501000000
H	-1.081285000000	2.093737000000	1.902509000000
H	-0.927014000000	2.614865000000	-1.493463000000
H	-3.860801000000	2.144835000000	-0.749814000000
O	1.612549000000	1.728165000000	0.005945000000
C	3.586750000000	2.765574000000	0.816409000000
C	3.860628000000	2.142165000000	-0.555666000000
C	2.303190000000	2.059893000000	1.240011000000
C	2.459525000000	2.043958000000	-1.131073000000
H	3.425917000000	3.844180000000	0.722074000000
H	2.518386000000	1.126857000000	1.767438000000
H	4.293819000000	1.141307000000	-0.450118000000
H	2.339649000000	1.254487000000	-1.873439000000
H	4.521543000000	2.749534000000	-1.177806000000
H	2.112128000000	2.996400000000	-1.546120000000
H	1.629317000000	2.676832000000	1.838922000000
H	4.393949000000	2.599682000000	1.532879000000
C	2.061701000000	-1.129860000000	-0.158011000000
C	2.764478000000	-1.454919000000	1.003654000000
C	2.610614000000	-1.429233000000	-1.406305000000
C	4.025157000000	-2.047311000000	0.914143000000
C	3.873051000000	-2.017673000000	-1.489581000000
C	4.583086000000	-2.322981000000	-0.331016000000
H	2.345130000000	-1.243724000000	1.985541000000
H	2.067316000000	-1.202755000000	-2.321328000000
H	4.568181000000	-2.293071000000	1.822068000000
H	4.298352000000	-2.240269000000	-2.463650000000
H	5.562945000000	-2.784278000000	-0.399211000000

Zero-point correction= 0.496034 (Hartree/Particle)
Thermal correction to Energy= 0.523177
Thermal correction to Enthalpy= 0.524121
Thermal correction to Gibbs Free Energy= 0.435883

Sum of electronic and zero-point Energies= -1226.605378
Sum of electronic and thermal Energies= -1226.578235
Sum of electronic and thermal Enthalpies= -1226.577291
Sum of electronic and thermal Free Energies= -1226.665530
E(RM06L) = -1227.81949251

Int1-C2-Pd

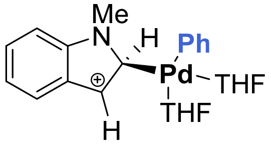

Pd	0.517202000000	-0.037791000000	-0.051690000000
C	-2.731718000000	-1.480706000000	-0.868203000000
C	-2.813778000000	-1.300302000000	0.542623000000
C	-4.006833000000	-0.878097000000	1.164253000000
C	-5.094272000000	-0.662801000000	0.350090000000
C	-5.033409000000	-0.840768000000	-1.060510000000
C	-3.872863000000	-1.241861000000	-1.672593000000
C	-1.417214000000	-1.888430000000	-1.146304000000
C	-0.687554000000	-1.858039000000	0.061068000000
H	-4.071114000000	-0.742888000000	2.238997000000
H	-6.033884000000	-0.350332000000	0.795073000000
H	-5.925590000000	-0.659416000000	-1.650125000000
H	-3.824126000000	-1.383016000000	-2.747788000000
H	0.105276000000	-2.566276000000	0.292890000000
N	-1.611247000000	-1.583362000000	1.093207000000
C	-1.299901000000	-1.574875000000	2.508646000000
H	-1.301943000000	-0.557271000000	2.912710000000
H	-2.027274000000	-2.181270000000	3.055266000000
H	-0.307915000000	-2.006888000000	2.650013000000
H	-1.002197000000	-2.171893000000	-2.104590000000
O	-1.269380000000	1.272893000000	-0.039332000000
C	-3.052184000000	2.544377000000	-0.824630000000
C	-2.664504000000	3.097979000000	0.552091000000
C	-1.766468000000	1.859304000000	-1.253760000000
C	-1.718415000000	2.025997000000	1.102254000000
H	-3.367066000000	3.322263000000	-1.523408000000
H	-1.905132000000	1.048281000000	-1.970940000000
H	-3.525172000000	3.256292000000	1.205097000000
H	-2.230454000000	1.325224000000	1.768867000000
H	-2.141439000000	4.053018000000	0.443515000000
H	-0.846105000000	2.443045000000	1.614608000000
H	-1.025638000000	2.571251000000	-1.639891000000
H	-3.852840000000	1.803096000000	-0.737769000000
O	1.784609000000	1.717592000000	-0.025927000000
C	3.776479000000	2.739862000000	0.749817000000
C	4.031613000000	2.104821000000	-0.620399000000
C	2.504741000000	2.030211000000	1.196678000000
C	2.623570000000	2.000724000000	-1.178840000000
H	3.608206000000	3.816782000000	0.648791000000
H	2.732714000000	1.089870000000	1.705597000000
H	4.466162000000	1.105098000000	-0.510634000000
H	2.493268000000	1.194227000000	-1.900652000000
H	4.684875000000	2.705950000000	-1.256507000000
H	2.275289000000	2.944364000000	-1.611977000000
H	1.844329000000	2.638678000000	1.818776000000
H	4.595194000000	2.583664000000	1.455328000000
C	2.126325000000	-1.154168000000	-0.124193000000
C	2.775333000000	-1.516779000000	1.056938000000
C	2.682444000000	-1.495851000000	-1.357592000000
C	4.001156000000	-2.182335000000	1.001814000000
C	3.909175000000	-2.159563000000	-1.405933000000
C	4.571751000000	-2.497426000000	-0.228318000000
H	2.338765000000	-1.281644000000	2.025945000000
H	2.173525000000	-1.244728000000	-2.285844000000
H	4.506033000000	-2.455832000000	1.923681000000
H	4.344153000000	-2.414554000000	-2.367780000000
H	5.524849000000	-3.014690000000	-0.269854000000

Zero-point correction= 0.495804 (Hartree/Particle)
Thermal correction to Energy= 0.523099
Thermal correction to Enthalpy= 0.524044
Thermal correction to Gibbs Free Energy= 0.434790
Sum of electronic and zero-point Energies= -1226.588998
Sum of electronic and thermal Energies= -1226.561703
Sum of electronic and thermal Enthalpies= -1226.560758
Sum of electronic and thermal Free Energies= -1226.650012
E(RM06L) = -1227.80388747

TS-Heck-C2-Indole

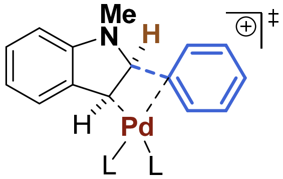

Pd	0.327144000	0.253948000	-0.399260000
C	-2.535492000	0.317255000	-0.328346000
C	-3.069938000	-0.935740000	0.021232000
C	-4.064298000	-1.069085000	0.988363000
C	-4.541719000	0.097587000	1.574226000
C	-4.043901000	1.357970000	1.213554000
C	-3.037518000	1.475908000	0.264271000
C	-1.463479000	0.082644000	-1.303887000
C	-1.403762000	-1.375075000	-1.440210000
H	-4.461785000	-2.038270000	1.270792000
H	-5.322701000	0.030025000	2.324665000
H	-4.450296000	2.246536000	1.684948000
H	-2.643225000	2.450010000	-0.010794000
H	-1.105941000	-1.862777000	-2.361297000
N	-2.463421000	-1.921976000	-0.752403000
C	-2.680584000	-3.341133000	-0.592564000
H	-2.204704000	-3.718302000	0.321527000
H	-3.753186000	-3.544577000	-0.556420000
H	-2.254382000	-3.865877000	-1.449638000
H	-1.366569000	0.693079000	-2.203835000
C	0.240502000	-1.810399000	-0.424619000
C	-0.035299000	-2.334751000	0.851773000
C	1.242169000	-2.414297000	-1.204240000
C	0.692957000	-3.413458000	1.344688000
C	1.975241000	-3.485929000	-0.704430000
C	1.699980000	-3.987199000	0.568253000
H	-0.830347000	-1.897782000	1.452244000
H	1.442902000	-2.045176000	-2.207578000
H	0.475702000	-3.807369000	2.332797000
H	2.750466000	-3.942064000	-1.312530000
H	2.262115000	-4.833674000	0.949824000
O	2.382530000	0.432162000	0.493089000
C	4.423411000	-0.675487000	0.928396000
C	4.713295000	0.665219000	0.247032000
C	2.998607000	-0.480046000	1.430537000
C	3.373211000	0.974027000	-0.405446000
H	5.119356000	-0.907832000	1.737368000
H	2.412376000	-1.398461000	1.450627000
H	5.525204000	0.612974000	-0.481664000
H	3.275432000	0.474185000	-1.377313000
H	4.965203000	1.429034000	0.990224000
H	3.160218000	2.038285000	-0.527034000
H	2.969648000	-0.009142000	2.419136000
H	4.457267000	-1.491444000	0.198244000
O	0.385729000	2.451830000	-0.307455000
C	0.461094000	4.549410000	0.725821000
C	-0.409987000	4.670358000	-0.529671000
C	0.400358000	3.056063000	1.002913000
C	-0.073654000	3.393819000	-1.299655000
H	0.087663000	5.143354000	1.562677000
H	1.265058000	2.650834000	1.531011000

H	-0.200186000	5.568459000	-1.114165000
H	0.739958000	3.536870000	-2.017248000
H	-1.470145000	4.686535000	-0.254635000
H	-0.935618000	2.966903000	-1.819688000
H	-0.517904000	2.772643000	1.532414000
H	1.490516000	4.857341000	0.515090000

Zero-point correction= 0.494986 (Hartree/Particle)
Thermal correction to Energy= 0.521502
Thermal correction to Enthalpy= 0.522446
Thermal correction to Gibbs Free Energy= 0.435148
Sum of electronic and zero-point Energies= -1226.584587
Sum of electronic and thermal Energies= -1226.558071
Sum of electronic and thermal Enthalpies= -1226.557127
Sum of electronic and thermal Free Energies= -1226.644425
E(RM06L) = -1227.79090820

TS-Heck-C3-Indole

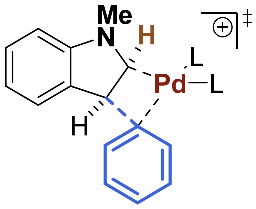

Pd	0.390619000	-0.240639000	-0.436032000
C	-2.998520000	-0.203442000	-0.451407000
C	-2.876433000	-1.459045000	0.168369000
C	-3.771917000	-1.883508000	1.152161000
C	-4.808788000	-1.021475000	1.477455000
C	-4.952109000	0.231350000	0.856607000
C	-4.045394000	0.652417000	-0.103437000
C	-1.878222000	-0.061863000	-1.373488000
C	-1.154302000	-1.314250000	-1.291062000
H	-3.673205000	-2.850276000	1.634600000
H	-5.530962000	-1.324855000	2.228684000
H	-5.780367000	0.873356000	1.136477000
H	-4.138851000	1.627581000	-0.571328000
H	-0.676948000	-1.796626000	-2.142789000
N	-1.786616000	-2.127399000	-0.357375000
C	-1.378324000	-3.467495000	0.001305000
H	-1.234504000	-3.548819000	1.084133000
H	-2.130280000	-4.199490000	-0.310988000
H	-0.429521000	-3.686023000	-0.490894000
H	-1.963432000	0.460637000	-2.316860000
C	-0.701583000	1.474289000	-0.642092000
C	-0.232327000	2.318321000	-1.658409000
C	-1.190050000	2.027559000	0.547203000
C	-0.186642000	3.695372000	-1.454592000
C	-1.141330000	3.404717000	0.743608000
C	-0.637282000	4.239740000	-0.252907000
H	0.092161000	1.902828000	-2.609705000
H	-1.621911000	1.384716000	1.309683000
H	0.183696000	4.344465000	-2.242335000
H	-1.515180000	3.828192000	1.670989000
H	-0.618661000	5.314557000	-0.103082000
O	2.146192000	0.839225000	0.530201000
C	3.063243000	3.014725000	0.501799000
C	3.019851000	2.515777000	1.948761000
C	2.962550000	1.713822000	-0.280974000
C	1.999075000	1.388849000	1.858836000
H	3.971292000	3.573080000	0.263980000
H	2.477634000	1.828395000	-1.253007000
H	2.716508000	3.286186000	2.660781000
H	0.977064000	1.765324000	1.964626000
H	3.998413000	2.130339000	2.253524000
H	2.162979000	0.579798000	2.575390000
H	3.939523000	1.234424000	-0.413702000

H	2.194813000	3.646682000	0.288010000
O	1.668046000	-2.051098000	-0.257108000
C	3.137319000	-3.506450000	0.818267000
C	3.902597000	-2.873530000	-0.350920000
C	2.053625000	-2.470960000	1.064586000
C	2.804492000	-2.161534000	-1.149184000
H	3.764691000	-3.673023000	1.696416000
H	1.155642000	-2.851381000	1.555453000
H	4.438681000	-3.607342000	-0.955954000
H	2.468046000	-2.732060000	-2.018875000
H	4.631147000	-2.148271000	0.024406000
H	3.100225000	-1.158494000	-1.469734000
H	2.438099000	-1.606493000	1.618804000
H	2.689985000	-4.461541000	0.524731000

Zero-point correction= 0.495133 (Hartree/Particle)
Thermal correction to Energy= 0.521740
Thermal correction to Enthalpy= 0.522684
Thermal correction to Gibbs Free Energy= 0.435225
Sum of electronic and zero-point Energies= -1226.575570
Sum of electronic and thermal Energies= -1226.548963
Sum of electronic and thermal Enthalpies= -1226.548019
Sum of electronic and thermal Free Energies= -1226.635478
E(RM06L) = -1227.78721045

Int2

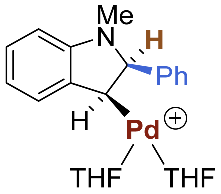

Pd	0.370736000	-0.255477000	-0.490080000
C	-2.377025000	0.589006000	-0.434681000
C	-3.071118000	-0.248751000	0.461581000
C	-3.995152000	0.280905000	1.362892000
C	-4.207710000	1.656180000	1.346282000
C	-3.537215000	2.496698000	0.449227000
C	-2.622362000	1.961238000	-0.449520000
C	-1.494169000	-0.244746000	-1.252726000
C	-1.838068000	-1.688067000	-0.871245000
H	-4.548937000	-0.353516000	2.046506000
H	-4.928598000	2.083074000	2.036721000
H	-3.747985000	3.560786000	0.445509000
H	-2.116610000	2.597264000	-1.170327000
H	-2.320949000	-2.258009000	-1.677940000
N	-2.708455000	-1.570842000	0.282727000
C	-3.572495000	-2.673745000	0.648941000
H	-3.848561000	-2.600411000	1.704235000
H	-4.489312000	-2.703007000	0.043547000
H	-3.030584000	-3.613433000	0.512910000
H	-1.386823000	-0.017625000	-2.316455000
C	-0.464025000	-2.308898000	-0.523835000
C	-0.157400000	-2.778855000	0.775217000
C	0.480926000	-2.512080000	-1.559110000
C	1.035344000	-3.440073000	1.015427000
C	1.682145000	-3.184454000	-1.300197000
C	1.953086000	-3.647276000	-0.021638000
H	-0.882457000	-2.627311000	1.567880000
H	0.228251000	-2.240392000	-2.581294000
H	1.250998000	-3.813378000	2.011505000
H	2.387612000	-3.351533000	-2.107334000
H	2.877462000	-4.180884000	0.175401000
O	2.430995000	-0.448735000	0.463910000
C	4.000854000	1.265198000	0.609997000
C	3.721963000	0.788120000	2.046734000
C	3.581820000	0.059545000	-0.217466000
C	2.634662000	-0.293249000	1.877484000
H	5.045157000	1.543365000	0.452261000

H	3.282564000	0.291304000	-1.241647000
H	3.394171000	1.602774000	2.697114000
H	1.670907000	-0.019103000	2.314444000
H	4.622374000	0.356502000	2.491464000
H	2.941940000	-1.257851000	2.294856000
H	4.359509000	-0.715209000	-0.236213000
H	3.370820000	2.121702000	0.350155000
O	0.753139000	1.899840000	-0.462991000
C	0.485216000	4.163719000	-1.102807000
C	0.939320000	4.084705000	0.359448000
C	0.770921000	2.756987000	-1.625334000
C	0.542016000	2.667133000	0.740543000
H	1.015603000	4.928166000	-1.674186000
H	0.022810000	2.393901000	-2.335296000
H	0.459389000	4.832574000	0.994080000
H	-0.513125000	2.592499000	1.025517000
H	2.024271000	4.213900000	0.435951000
H	1.165277000	2.223089000	1.519884000
H	1.763568000	2.670188000	-2.078715000
H	-0.585676000	4.384127000	-1.154588000

Zero-point correction= 0.497205 (Hartree/Particle)
Thermal correction to Energy= 0.523717
Thermal correction to Enthalpy= 0.524661
Thermal correction to Gibbs Free Energy= 0.438725
Sum of electronic and zero-point Energies= -1226.602377
Sum of electronic and thermal Energies= -1226.575865
Sum of electronic and thermal Enthalpies= -1226.574921
Sum of electronic and thermal Free Energies= -1226.660857
E(RM06L) = -1227.81264787

TS-Heck-C2-Benzothiophene

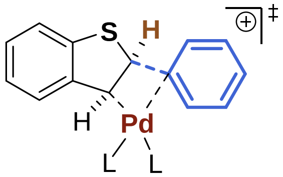

Pd	0.143896000	0.149672000	-0.500582000
C	-2.432907000	-1.094279000	-0.462392000
C	-2.389153000	-2.142320000	0.470657000
C	-3.366504000	-2.284229000	1.453088000
C	-4.420597000	-1.378760000	1.464248000
C	-4.500119000	-0.352226000	0.514648000
C	-3.510620000	-0.202344000	-0.445961000
C	-1.268980000	-1.088397000	-1.353212000
C	-0.386167000	-2.189950000	-1.071117000
H	-3.321651000	-3.089865000	2.178416000
H	-5.200704000	-1.480338000	2.211661000
H	-5.344369000	0.329387000	0.530308000
H	-3.557614000	0.599135000	-1.176127000
H	0.132791000	-2.703370000	-1.869286000
S	-1.020726000	-3.219792000	0.205990000
H	-1.356780000	-0.723449000	-2.377076000
C	1.466989000	-1.416693000	-0.488015000
C	1.885360000	-1.841734000	0.775951000
C	2.370458000	-1.408979000	-1.558653000
C	3.219558000	-2.175771000	0.986931000
C	3.702643000	-1.755539000	-1.342998000
C	4.129213000	-2.128675000	-0.069902000
H	1.168010000	-1.922935000	1.588685000
H	2.038922000	-1.134608000	-2.557451000
H	3.545297000	-2.495444000	1.972215000
H	4.403810000	-1.744373000	-2.171677000
H	5.164702000	-2.409058000	0.094040000
O	1.642053000	1.513084000	0.462360000
C	3.419014000	1.520635000	2.024194000
C	3.803801000	2.379712000	0.815915000
C	1.905384000	1.428818000	1.882842000
C	2.855072000	1.852070000	-0.249378000

H	3.718947000	1.957911000	2.978777000
H	1.487564000	0.491515000	2.257209000
H	4.850481000	2.265328000	0.526396000
H	3.245683000	0.946181000	-0.722698000
H	3.613665000	3.439199000	1.016196000
H	2.589615000	2.580149000	-1.019505000
H	1.395060000	2.269691000	2.365177000
H	3.867134000	0.525146000	1.938026000
O	-1.180381000	1.882877000	-0.516562000
C	-2.571973000	3.508659000	0.366504000
C	-1.814971000	4.153476000	-0.813985000
C	-1.672412000	2.346112000	0.752631000
C	-0.855351000	3.049310000	-1.298498000
H	-3.545248000	3.126948000	0.044295000
H	-0.824308000	2.668539000	1.371257000
H	-1.251644000	5.031633000	-0.489240000
H	0.193190000	3.309647000	-1.117221000
H	-2.498567000	4.471900000	-1.603213000
H	-0.984662000	2.771299000	-2.345806000
H	-2.183286000	1.506960000	1.227048000
H	-2.733412000	4.203586000	1.193140000

Zero-point correction= 0.451369 (Hartree/Particle)
Thermal correction to Energy= 0.476466
Thermal correction to Enthalpy= 0.477410
Thermal correction to Gibbs Free Energy= 0.393463
Sum of electronic and zero-point Energies= -1530.145197
Sum of electronic and thermal Energies= -1530.120100
Sum of electronic and thermal Enthalpies= -1530.119156
Sum of electronic and thermal Free Energies= -1530.203103
E(RM06L) = -1531.29891797

TS-Heck-C3-Benzothiophene

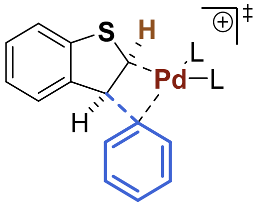

Pd	0.606964000	-0.219749000	-0.454179000
C	-2.874121000	-0.842943000	-0.509316000
C	-2.714013000	-1.860845000	0.441200000
C	-3.706615000	-2.127301000	1.386384000
C	-4.870759000	-1.374529000	1.340812000
C	-5.048761000	-0.364450000	0.382414000
C	-4.052487000	-0.088387000	-0.536620000
C	-1.696956000	-0.674497000	-1.348865000
C	-0.690247000	-1.662237000	-1.076556000
H	-3.580347000	-2.909340000	2.127638000
H	-5.659603000	-1.575098000	2.058658000
H	-5.972666000	0.203740000	0.367933000
H	-4.167672000	0.710497000	-1.263160000
H	-0.163703000	-2.154162000	-1.894196000
S	-1.183186000	-2.706526000	0.278542000
H	-1.789282000	-0.273633000	-2.351827000
C	-0.921234000	1.135891000	-0.600523000
C	-0.838202000	2.070822000	-1.637819000
C	-1.456758000	1.513360000	0.637936000
C	-1.228751000	3.390975000	-1.415875000
C	-1.834651000	2.832474000	0.856224000
C	-1.720649000	3.772646000	-0.169972000
H	-0.469633000	1.774254000	-2.617046000
H	-1.596282000	0.771244000	1.420692000
H	-1.161404000	4.115907000	-2.221209000
H	-2.238617000	3.125023000	1.820534000
H	-2.037431000	4.797334000	-0.003423000
O	1.980189000	1.365445000	0.316278000

C	2.171438000	3.698363000	0.583656000
C	2.542147000	3.018721000	1.905236000
C	2.339555000	2.561833000	-0.412898000
C	1.916725000	1.640413000	1.734639000
H	2.805708000	4.555058000	0.345888000
H	1.686092000	2.639159000	-1.282672000
H	2.150196000	3.538934000	2.781750000
H	0.864245000	1.637238000	2.037398000
H	3.629448000	2.941477000	2.008416000
H	2.446274000	0.835533000	2.250397000
H	3.378261000	2.451445000	-0.745059000
H	1.127445000	4.029224000	0.600260000
O	2.329743000	-1.588274000	-0.353992000
C	4.550179000	-2.030767000	0.276403000
C	3.775686000	-3.351997000	0.267901000
C	3.683921000	-1.151418000	-0.611846000
C	2.323807000	-2.884562000	0.286006000
H	5.567721000	-2.130747000	-0.107404000
H	3.734040000	-0.088505000	-0.375815000
H	4.009551000	-3.993842000	1.119794000
H	1.935195000	-2.759712000	1.301721000
H	3.986647000	-3.910334000	-0.649672000
H	1.654854000	-3.540756000	-0.277162000
H	3.894705000	-1.305921000	-1.675866000
H	4.601879000	-1.617895000	1.289605000

Zero-point correction= 0.450787 (Hartree/Particle)
Thermal correction to Energy= 0.476062
Thermal correction to Enthalpy= 0.477006
Thermal correction to Gibbs Free Energy= 0.392933
Sum of electronic and zero-point Energies= -1530.146644
Sum of electronic and thermal Energies= -1530.121368
Sum of electronic and thermal Enthalpies= -1530.120424
Sum of electronic and thermal Free Energies= -1530.204497
E(RM06L) = -1531.29908460

TS-Heck-C2-Benzofuran

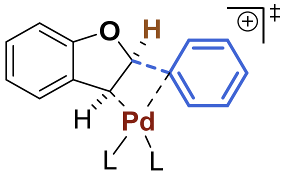

Pd	0.280159000000	0.922344000000	-0.198332000000
C	-2.183552000000	-0.461690000000	-0.485018000000
C	-1.897079000000	-1.805120000000	-0.239188000000
C	-2.639915000000	-2.611458000000	0.605284000000
C	-3.738774000000	-2.009775000000	1.211603000000
C	-4.061256000000	-0.664832000000	0.977552000000
C	-3.289337000000	0.123781000000	0.134074000000
C	-1.125967000000	0.022930000000	-1.382831000000
C	-0.289035000000	-1.131542000000	-1.580064000000
H	-2.387390000000	-3.653014000000	0.766708000000
H	-4.364029000000	-2.598961000000	1.874080000000
H	-4.929997000000	-0.235439000000	1.464838000000
H	-3.538799000000	1.166323000000	-0.032995000000
H	0.299813000000	-1.353942000000	-2.458299000000
O	-0.795744000000	-2.214346000000	-0.958931000000
H	-1.292630000000	0.726864000000	-2.197369000000
C	1.449049000000	-0.727225000000	-0.448603000000
C	1.519767000000	-1.591962000000	0.649265000000
C	2.581225000000	-0.507688000000	-1.245971000000
C	2.734349000000	-2.183906000000	0.984112000000
C	3.794145000000	-1.100144000000	-0.900671000000
C	3.870546000000	-1.932648000000	0.215202000000
H	0.628906000000	-1.808970000000	1.233182000000
H	2.516482000000	0.115289000000	-2.135221000000
H	2.791258000000	-2.852259000000	1.837705000000

H	4.673324000000	-0.927446000000	-1.513618000000
H	4.812592000000	-2.406571000000	0.471494000000
C	1.917375000000	1.476882000000	2.483088000000
H	0.935150000000	1.626790000000	2.931236000000
H	2.184622000000	0.417581000000	2.524699000000
H	2.657251000000	2.081326000000	3.013268000000
O	1.814654000000	1.920445000000	1.116813000000
H	2.678088000000	1.811634000000	0.693352000000
C	-1.511392000000	3.546553000000	-0.840823000000
H	-2.272943000000	2.865026000000	-1.219195000000
H	-1.999463000000	4.413886000000	-0.390149000000
H	-0.858964000000	3.860175000000	-1.661269000000
O	-0.777536000000	2.825990000000	0.157621000000
H	-0.132470000000	3.414242000000	0.574740000000

Zero-point correction= 0.321644 (Hartree/Particle)
Thermal correction to Energy= 0.342407
Thermal correction to Enthalpy= 0.343351
Thermal correction to Gibbs Free Energy= 0.270061
Sum of electronic and zero-point Energies= -973.883109
Sum of electronic and thermal Energies= -973.862345
Sum of electronic and thermal Enthalpies= -973.861401
Sum of electronic and thermal Free Energies= -973.934691
E(RM06L) = -974.825379782

TS-Heck-C3-Benzofuran

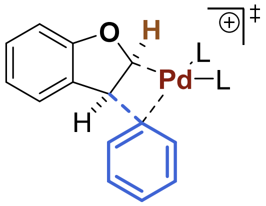

Pd	1.005532000000	-0.549383000000	-0.217237000000
C	-2.295179000000	-0.094717000000	-0.587742000000
C	-2.355265000000	-1.371630000000	-0.026960000000
C	-3.359531000000	-1.769106000000	0.843470000000
C	-4.340200000000	-0.823565000000	1.120521000000
C	-4.309823000000	0.464425000000	0.559626000000
C	-3.284814000000	0.845601000000	-0.292742000000
C	-1.087132000000	-0.057759000000	-1.400105000000
C	-0.524772000000	-1.373448000000	-1.273835000000
H	-3.382331000000	-2.765520000000	1.269118000000
H	-5.153961000000	-1.091028000000	1.786739000000
H	-5.098595000000	1.168088000000	0.802208000000
H	-3.243682000000	1.844973000000	-0.713622000000
H	-0.079615000000	-1.932693000000	-2.094273000000
O	-1.320454000000	-2.160038000000	-0.450988000000
H	-0.992441000000	0.515547000000	-2.312341000000
C	0.218739000000	1.322281000000	-0.473644000000
C	0.907218000000	2.127214000000	-1.389560000000
C	-0.326219000000	1.881070000000	0.686715000000
C	1.104858000000	3.477926000000	-1.106445000000
C	-0.123007000000	3.231242000000	0.960603000000
C	0.594397000000	4.027952000000	0.068626000000
H	1.291283000000	1.706007000000	-2.315332000000
H	-0.914106000000	1.269766000000	1.366500000000
H	1.646617000000	4.101472000000	-1.810776000000
H	-0.538047000000	3.664324000000	1.865471000000
H	0.738649000000	5.082738000000	0.278837000000
C	2.730257000000	0.294387000000	2.302367000000
H	2.692118000000	-0.767447000000	2.544530000000
H	3.648983000000	0.728752000000	2.704055000000
H	1.855554000000	0.802005000000	2.720754000000
O	2.733260000000	0.380119000000	0.868890000000
H	2.780957000000	1.311274000000	0.607181000000
C	3.161412000000	-2.878027000000	-0.260361000000
H	3.838503000000	-2.037031000000	-0.114914000000

H	3.544025000000	-3.753580000000	0.268970000000
H	3.058087000000	-3.092765000000	-1.327923000000
O	1.900584000000	-2.480615000000	0.306112000000
H	1.280780000000	-3.222090000000	0.292387000000

Zero-point correction= 0.321681 (Hartree/Particle)
Thermal correction to Energy= 0.342468
Thermal correction to Enthalpy= 0.343412
Thermal correction to Gibbs Free Energy= 0.269381
Sum of electronic and zero-point Energies= -973.881054
Sum of electronic and thermal Energies= -973.860267
Sum of electronic and thermal Enthalpies= -973.859323
Sum of electronic and thermal Free Energies= -973.933354
E(RM06L) = -974.823750776

Int2 (C3-Ar)

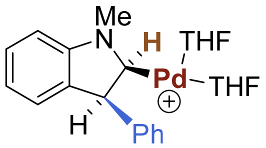

Pd	-0.316205000000	-0.094490000000	-0.635938000000
C	3.141634000000	-0.436239000000	-0.050176000000
C	2.987707000000	0.930545000000	0.200741000000
C	3.736560000000	1.590956000000	1.165050000000
C	4.665388000000	0.837110000000	1.884584000000
C	4.836703000000	-0.524516000000	1.641269000000
C	4.074580000000	-1.170226000000	0.663740000000
C	2.201929000000	-0.835595000000	-1.160469000000
C	1.450583000000	0.474878000000	-1.440287000000
H	3.618688000000	2.653928000000	1.349088000000
H	5.269595000000	1.326071000000	2.642020000000
H	5.572018000000	-1.085303000000	2.208132000000
H	4.218653000000	-2.228556000000	0.464532000000
H	1.227882000000	0.759753000000	-2.472133000000
N	2.023937000000	1.470767000000	-0.659073000000
C	1.857994000000	2.890892000000	-0.873641000000
H	1.552060000000	3.393476000000	0.050148000000
H	2.796111000000	3.339364000000	-1.221120000000
H	1.079405000000	3.048675000000	-1.619899000000
H	2.744064000000	-1.206372000000	-2.038877000000
C	1.079411000000	-1.830234000000	-0.793755000000
C	0.142035000000	-2.175220000000	-1.799953000000
C	1.000367000000	-2.489545000000	0.454952000000
C	-0.825216000000	-3.164896000000	-1.562639000000
C	0.042027000000	-3.465775000000	0.670720000000
C	-0.870678000000	-3.806996000000	-0.336274000000
H	0.256329000000	-1.774181000000	-2.804398000000
H	1.715193000000	-2.240014000000	1.231661000000
H	-1.516255000000	-3.436750000000	-2.353913000000
H	0.009731000000	-3.980820000000	1.625809000000
H	-1.603680000000	-4.587870000000	-0.158850000000
O	-2.249705000000	-0.655522000000	0.547133000000
C	-3.708806000000	-0.815962000000	2.383664000000
C	-4.520243000000	-0.895834000000	1.065709000000
C	-2.253915000000	-1.006220000000	1.933134000000
C	-3.457510000000	-1.191059000000	0.003167000000
H	-4.004075000000	-1.583571000000	3.102186000000
H	-1.532651000000	-0.359476000000	2.437166000000
H	-5.015297000000	0.056745000000	0.859716000000
H	-3.629264000000	-0.707683000000	-0.960648000000
H	-5.288529000000	-1.671372000000	1.091226000000

H	-3.330287000000	-2.269456000000	-0.157952000000
H	-1.928295000000	-2.048629000000	2.038858000000
H	-3.845099000000	0.156108000000	2.864294000000
O	-1.109605000000	1.974194000000	-0.428684000000
C	-1.729475000000	3.906495000000	0.685893000000
C	-2.758162000000	3.692824000000	-0.441574000000
C	-1.191226000000	2.501941000000	0.902502000000
C	-2.292378000000	2.396568000000	-1.134310000000
H	-2.174197000000	4.330059000000	1.588797000000
H	-0.190581000000	2.445177000000	1.335897000000
H	-2.787411000000	4.537335000000	-1.132630000000
H	-2.004380000000	2.527241000000	-2.179093000000
H	-3.762820000000	3.564309000000	-0.030512000000
H	-3.046928000000	1.605824000000	-1.065079000000
H	-1.881246000000	1.886381000000	1.495076000000
H	-0.922224000000	4.568046000000	0.357270000000

Zero-point correction= 0.497469 (Hartree/Particle)
Thermal correction to Energy= 0.524380
Thermal correction to Enthalpy= 0.525324
Thermal correction to Gibbs Free Energy= 0.435860
Sum of electronic and zero-point Energies= -1226.604699
Sum of electronic and thermal Energies= -1226.577788
Sum of electronic and thermal Enthalpies= -1226.576844
Sum of electronic and thermal Free Energies= -1226.666308
E(RM06L) = -1227.81620867

Int2 (C2-Ar/Indole)

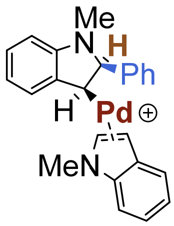

Pd	-0.242468000000	0.291420000000	-0.358682000000
C	2.347710000000	-1.039511000000	-0.828840000000
C	3.270026000000	-0.516235000000	0.103604000000
C	4.172327000000	-1.358660000000	0.757731000000
C	4.118449000000	-2.716814000000	0.467277000000
C	3.207417000000	-3.247128000000	-0.460155000000
C	2.321260000000	-2.407052000000	-1.117173000000
C	1.551583000000	0.061565000000	-1.337902000000
C	2.184820000000	1.331429000000	-0.769970000000
H	4.901223000000	-0.974757000000	1.463090000000
H	4.814480000000	-3.385501000000	0.964233000000
H	3.212968000000	-4.310637000000	-0.672059000000
H	1.621957000000	-2.798843000000	-1.851331000000
H	2.698364000000	1.918732000000	-1.546147000000
N	3.118204000000	0.847304000000	0.227316000000
C	4.130326000000	1.730680000000	0.765314000000
H	4.501591000000	1.339524000000	1.715661000000
H	4.976991000000	1.858344000000	0.077367000000
H	3.681192000000	2.708366000000	0.960396000000
H	1.234999000000	0.070770000000	-2.382062000000
C	1.025860000000	2.154562000000	-0.167937000000
C	0.714383000000	2.065701000000	1.205281000000
C	0.255049000000	3.007309000000	-0.988890000000
C	-0.336188000000	2.822882000000	1.736834000000
C	-0.778894000000	3.760304000000	-0.447127000000
C	-1.074523000000	3.668347000000	0.915982000000
H	1.341886000000	1.458944000000	1.851091000000
H	0.494409000000	3.091437000000	-2.045838000000
H	-0.553982000000	2.762032000000	2.798122000000
H	-1.354969000000	4.421498000000	-1.085602000000
H	-1.877847000000	4.265136000000	1.335300000000
C	-2.960276000000	-1.329302000000	0.545459000000
C	-2.704841000000	-1.091545000000	-0.820964000000

C	-3.712645000000	-0.547244000000	-1.627885000000
C	-1.325781000000	-1.486050000000	-1.065715000000
C	-4.179009000000	-1.006955000000	1.144306000000
N	-1.823624000000	-1.895459000000	1.113580000000
C	-5.157335000000	-0.464398000000	0.325674000000
H	-4.362001000000	-1.182335000000	2.199337000000
C	-4.930959000000	-0.242834000000	-1.045193000000
H	-6.122889000000	-0.211580000000	0.751347000000
H	-5.727458000000	0.173902000000	-1.652605000000
H	-3.539069000000	-0.370598000000	-2.684805000000
C	-0.846211000000	-1.970158000000	0.168029000000
H	-0.902608000000	-1.734242000000	-2.031640000000
H	0.085949000000	-2.479600000000	0.379148000000
C	-1.658477000000	-2.170441000000	2.526905000000
H	-1.678338000000	-1.241520000000	3.106662000000
H	-2.459073000000	-2.829056000000	2.872886000000
H	-0.700744000000	-2.668936000000	2.682689000000

Zero-point correction= 0.416551 (Hartree/Particle)
Thermal correction to Energy= 0.440350
Thermal correction to Enthalpy= 0.441294
Thermal correction to Gibbs Free Energy= 0.360790
Sum of electronic and zero-point Energies= -1164.890466
Sum of electronic and thermal Energies= -1164.866668
Sum of electronic and thermal Enthalpies= -1164.865724
Sum of electronic and thermal Free Energies= -1164.946227
E(RM06L) = -1166.01372381

Int2 (C3-Ar/Indole)

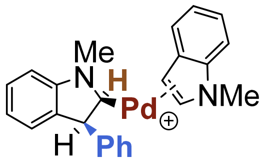

Pd	0.396938000000	0.199250000000	-0.867607000000
C	-3.030636000000	0.423809000000	0.038943000000
C	-2.986577000000	-0.947448000000	-0.230966000000
C	-3.694893000000	-1.875655000000	0.518548000000
C	-4.490214000000	-1.384743000000	1.556241000000
C	-4.569646000000	-0.018634000000	1.820776000000
C	-3.840667000000	0.897865000000	1.057431000000
C	-2.147944000000	1.141494000000	-0.954027000000
C	-1.505149000000	-0.034899000000	-1.700961000000
H	-3.652325000000	-2.938650000000	0.303415000000
H	-5.065039000000	-2.081021000000	2.158010000000
H	-5.208879000000	0.337656000000	2.621354000000
H	-3.917113000000	1.962725000000	1.257689000000
H	-1.238480000000	0.053662000000	-2.756518000000
N	-2.164717000000	-1.179607000000	-1.344344000000
C	-1.951658000000	-2.480022000000	-1.938699000000
H	-1.350684000000	-3.127199000000	-1.288429000000
H	-2.914825000000	-2.963395000000	-2.124080000000
H	-1.431782000000	-2.356698000000	-2.890485000000
H	-2.742573000000	1.767296000000	-1.633556000000
C	-0.969582000000	1.952556000000	-0.388851000000
C	-0.034739000000	2.492566000000	-1.307081000000
C	-0.780356000000	2.196589000000	0.988633000000
C	1.056265000000	3.254028000000	-0.845543000000
C	0.292259000000	2.958760000000	1.423664000000
C	1.218412000000	3.481670000000	0.510566000000
H	-0.228727000000	2.441927000000	-2.376543000000
H	-1.486720000000	1.790112000000	1.703985000000
H	1.753989000000	3.671510000000	-1.563794000000
H	0.412662000000	3.155400000000	2.484436000000
H	2.053654000000	4.075731000000	0.866017000000
C	2.658409000000	-1.088281000000	1.060318000000
C	2.678234000000	-1.385265000000	-0.319359000000

C	3.866567000000	-1.217693000000	-1.044620000000
C	1.333208000000	-1.808519000000	-0.677843000000
C	3.774539000000	-0.582893000000	1.730249000000
N	1.390820000000	-1.370466000000	1.553274000000
C	4.932663000000	-0.417031000000	0.988711000000
H	3.745126000000	-0.341434000000	2.787709000000
C	4.982014000000	-0.738207000000	-0.381203000000
H	5.824598000000	-0.036493000000	1.475616000000
H	5.912996000000	-0.604270000000	-0.921861000000
H	3.906453000000	-1.456391000000	-2.102941000000
C	0.595508000000	-1.766402000000	0.517471000000
H	1.062798000000	-2.378377000000	-1.558794000000
H	-0.410019000000	-2.115412000000	0.717300000000
C	0.910735000000	-0.975457000000	2.861052000000
H	1.605970000000	-1.319279000000	3.630009000000
H	0.809083000000	0.114851000000	2.920339000000
H	-0.062513000000	-1.436363000000	3.037825000000

Zero-point correction= 0.417184 (Hartree/Particle)
Thermal correction to Energy= 0.440648
Thermal correction to Enthalpy= 0.441592
Thermal correction to Gibbs Free Energy= 0.362480
Sum of electronic and zero-point Energies= -1164.896585
Sum of electronic and thermal Energies= -1164.873121
Sum of electronic and thermal Enthalpies= -1164.872177
Sum of electronic and thermal Free Energies= -1164.951289
E(RM06L) = -1166.02062986

10. NMR spectra

1-methyl-2-phenyl-1H-indole (3a)

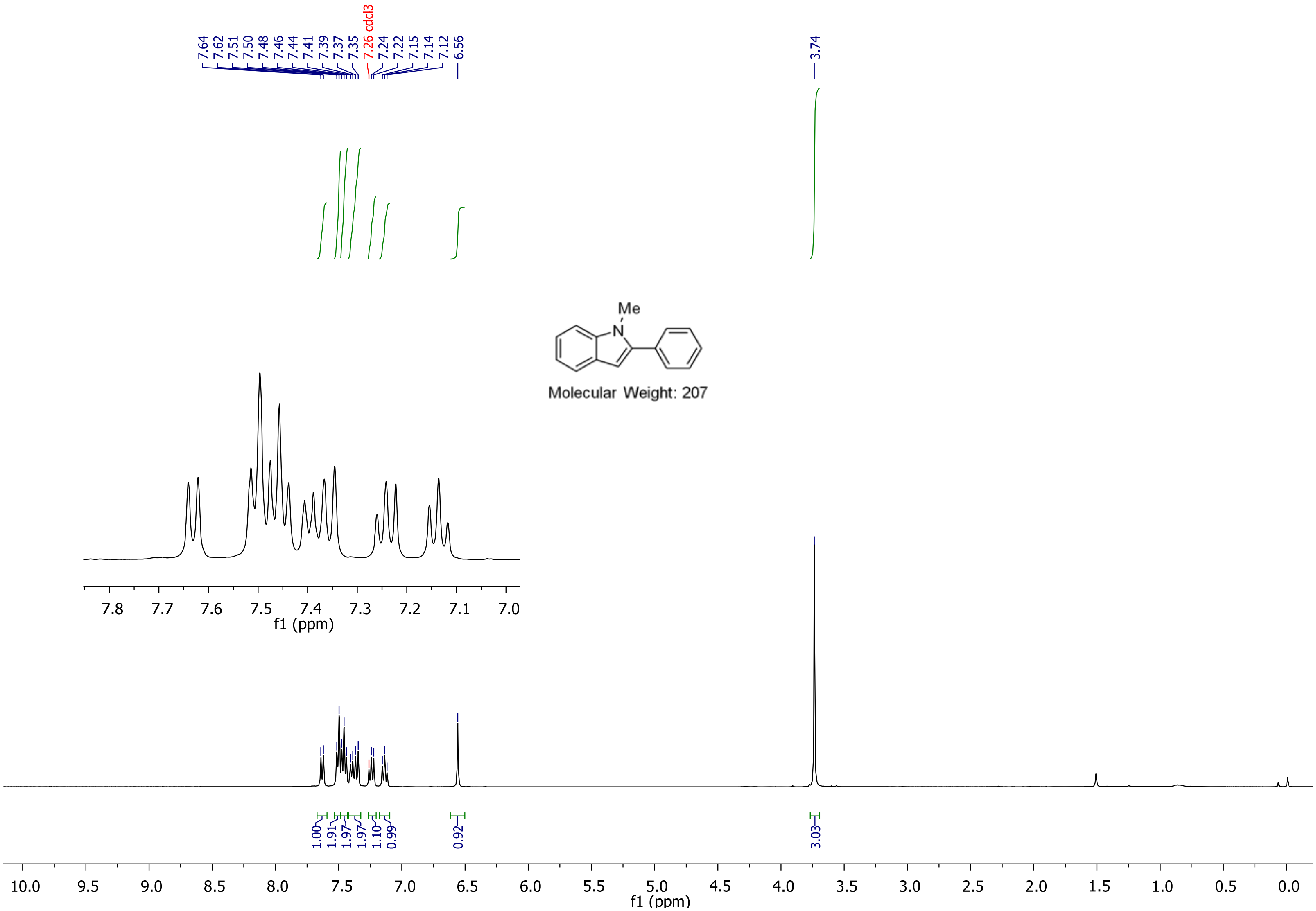

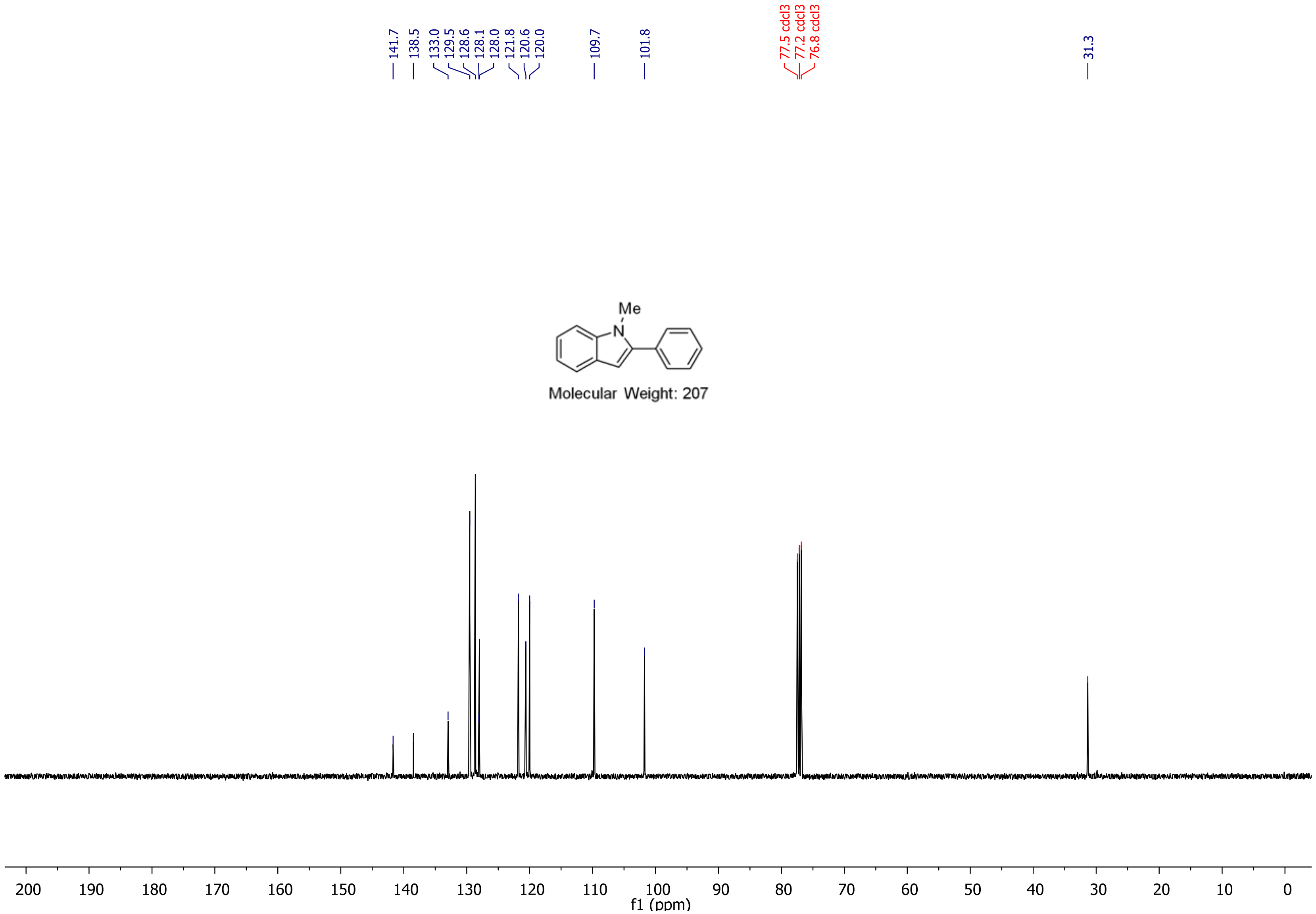

141.7
138.5
133.0
129.5
128.6
128.1
128.0
121.8
120.6
120.0
109.7
101.8
77.5 cdcl3
77.2 cdcl3
76.8 cdcl3
31.3
Me
N
Molecular Weight: 207
200
190
180
170
160
150
140
130
120
110
100
90
80
70
60
50
40
30
20
10
0
f1 (ppm)

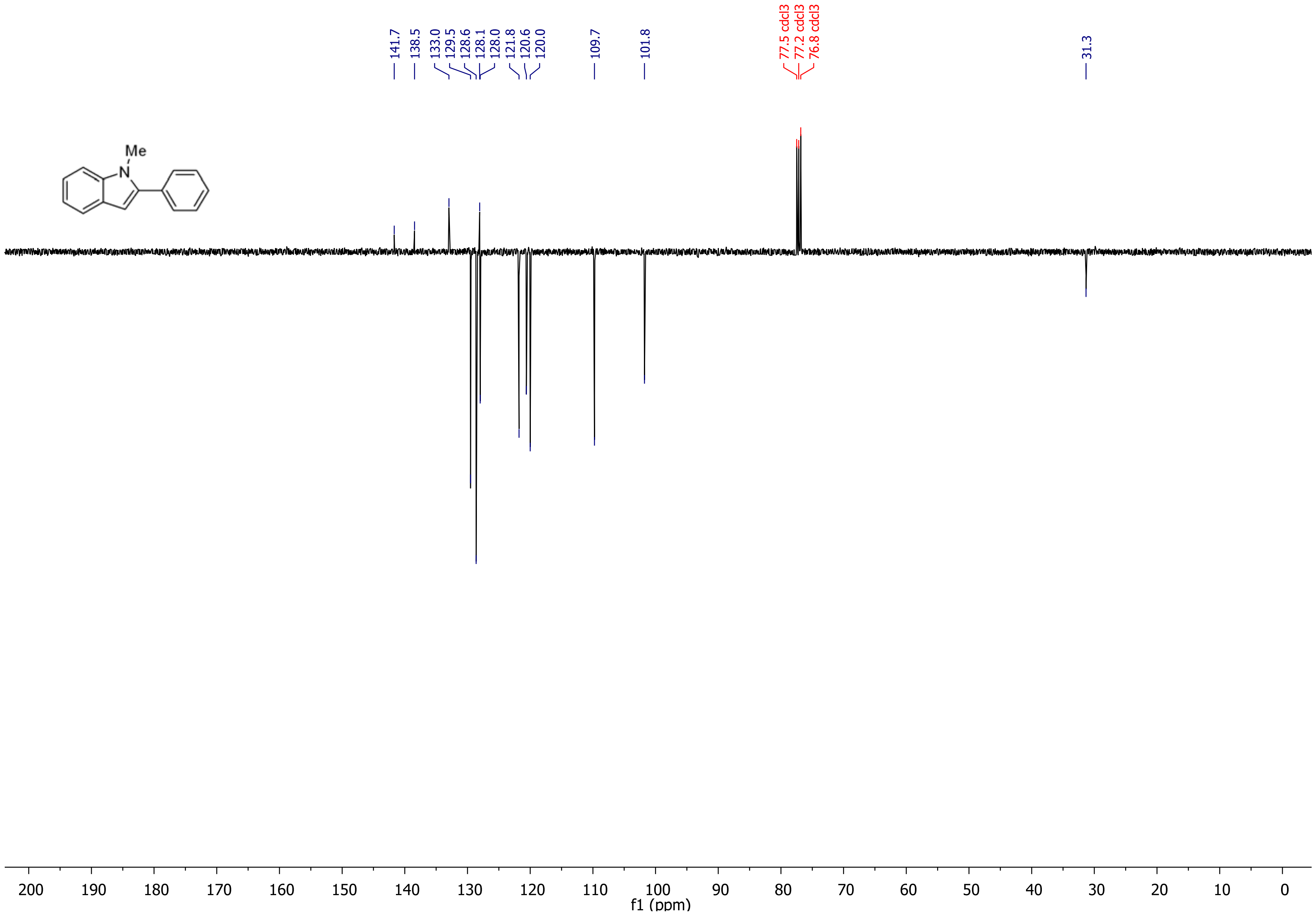

141.7
138.5
133.0
129.5
128.6
128.1
128.0
121.8
120.6
120.0
109.7
101.8
77.5 cdcl3
77.2 cdcl3
76.8 cdcl3
31.3
Me
N
200
190
180
170
160
150
140
130
120
110
100
90
80
70
60
50
40
30
20
10
0
f1 (ppm)

1,3-dimethyl-2-phenyl-*1H*-indole (3b)

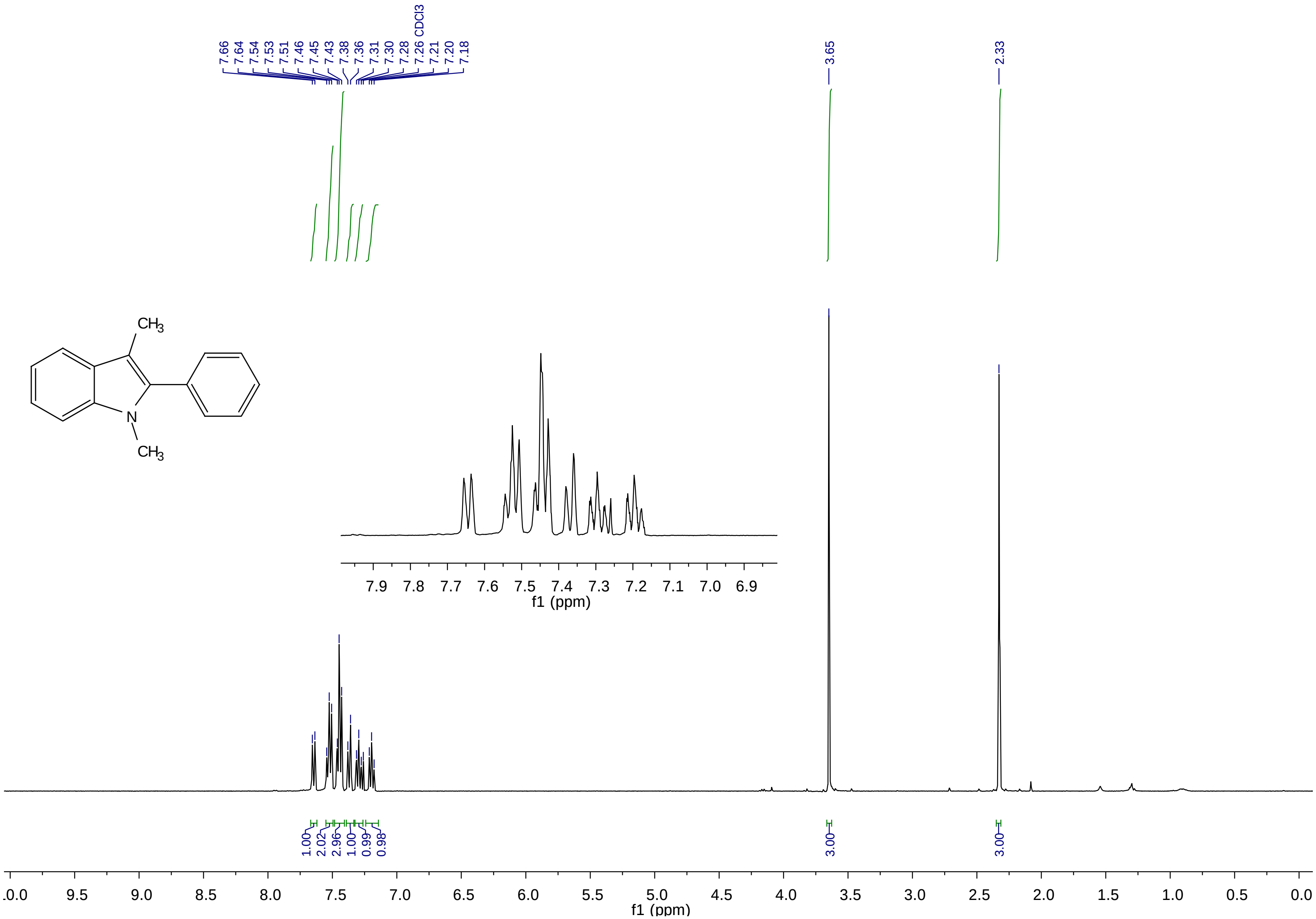

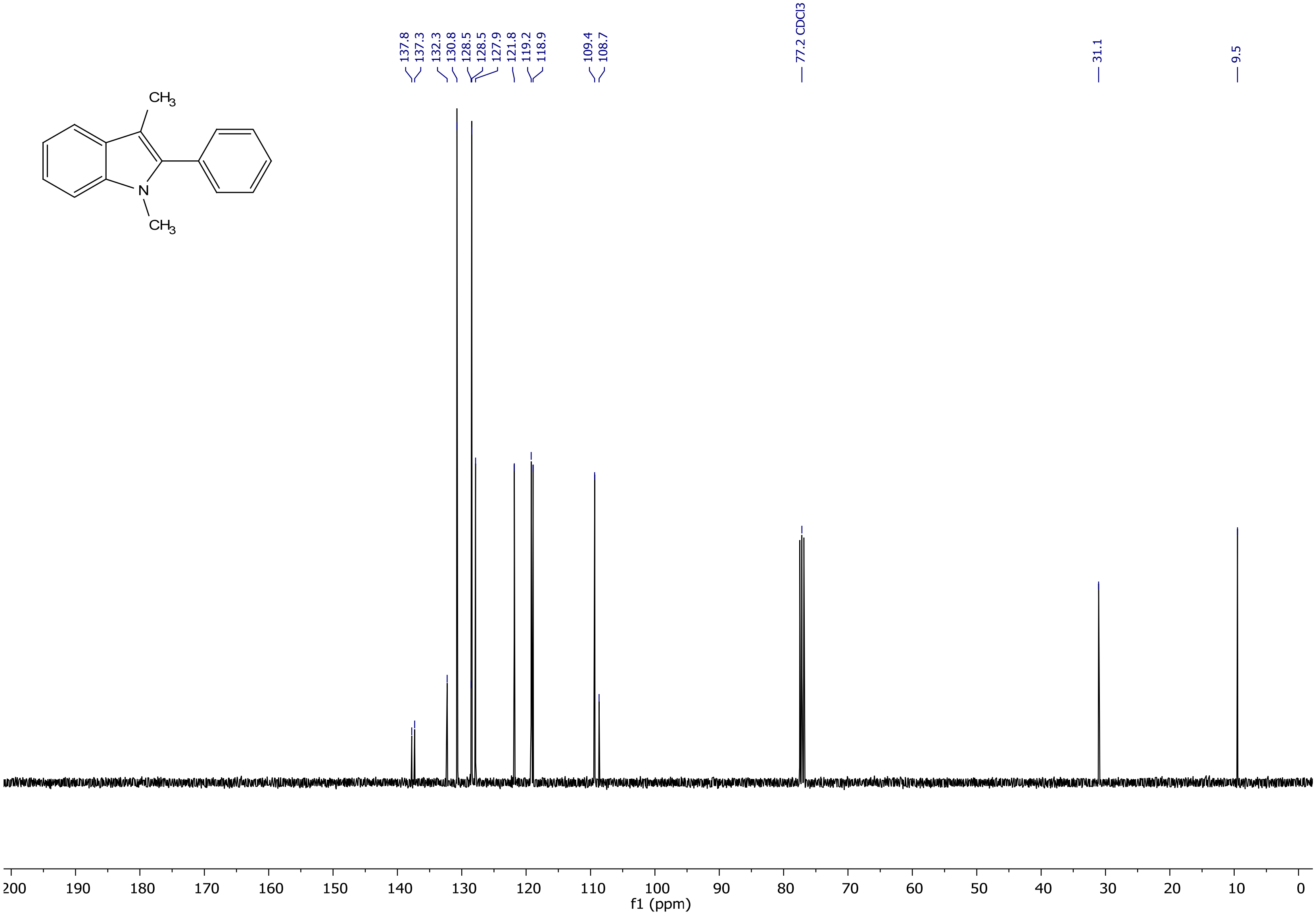
CH3
N
CH3
137.8
137.3
132.3
130.8
128.5
128.5
127.9
121.8
119.2
118.9
109.4
108.7
77.2 CDCl3
31.1
9.5
200
190
180
170
160
150
140
130
120
110
100
90
80
70
60
50
40
30
20
10
0
f1 (ppm)

1-methyl-2-(2-methylphenyl)-1*H*-indole (3c)

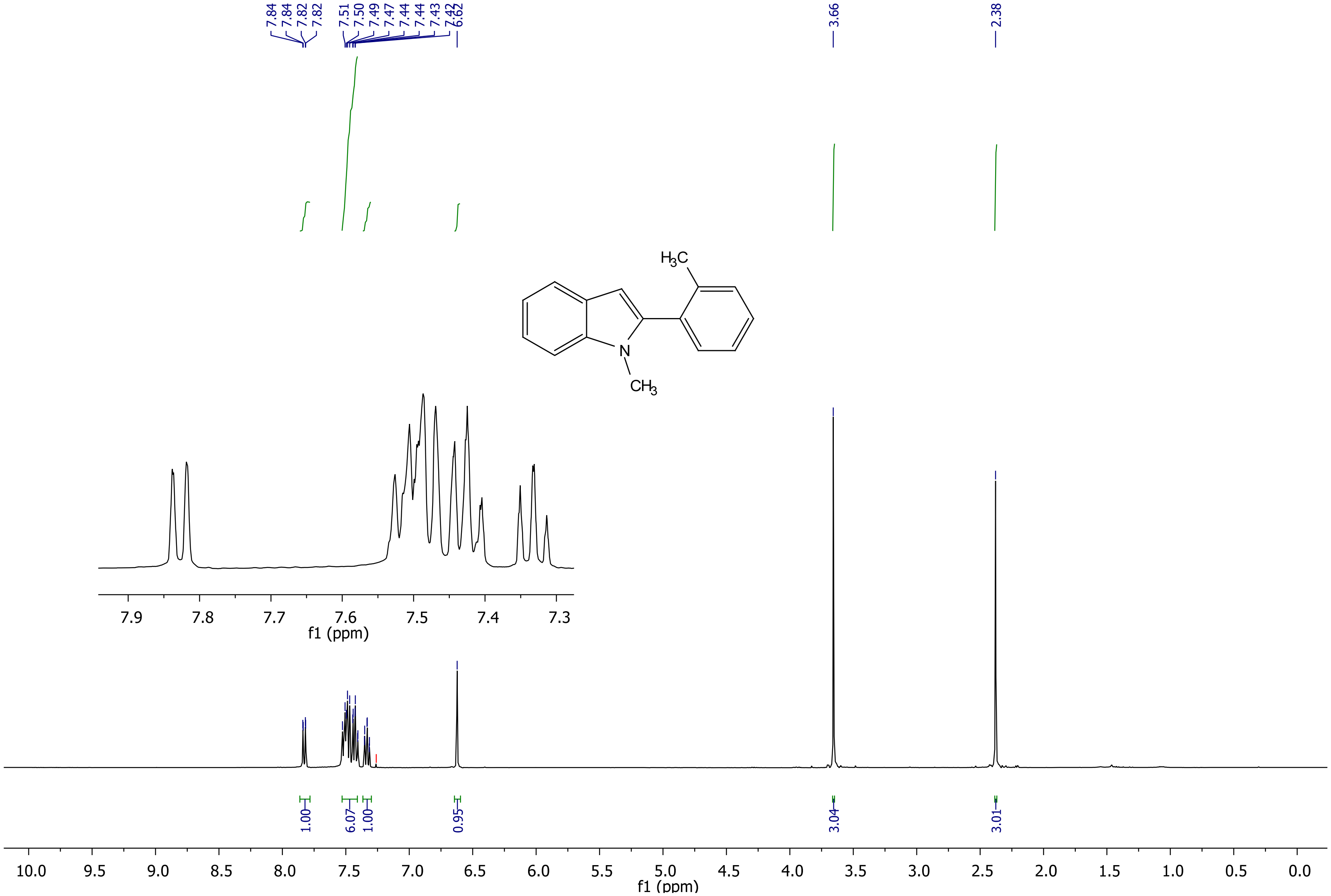

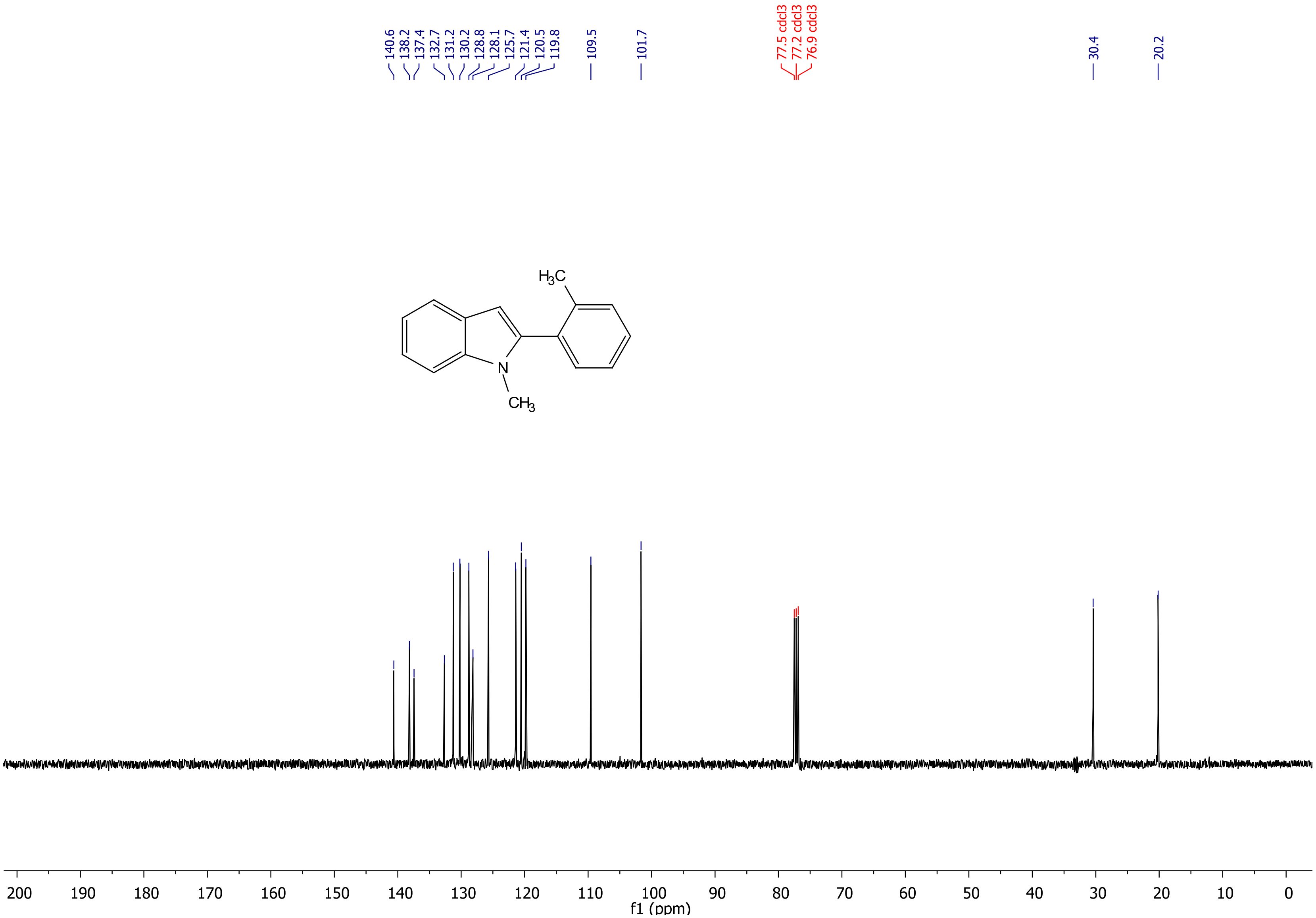

140.6
138.2
137.4
132.7
131.2
130.2
128.8
128.1
125.7
121.4
120.5
119.8
109.5
101.7
77.5 cdcl3
77.2 cdcl3
76.9 cdcl3
30.4
20.2
H_3C
N
CH_3
200
190
180
170
160
150
140
130
120
110
100
90
80
70
60
50
40
30
20
10
0
f1 (ppm)

1-methyl-2-(*p*-tolyl)-*1H*-indole (3d)

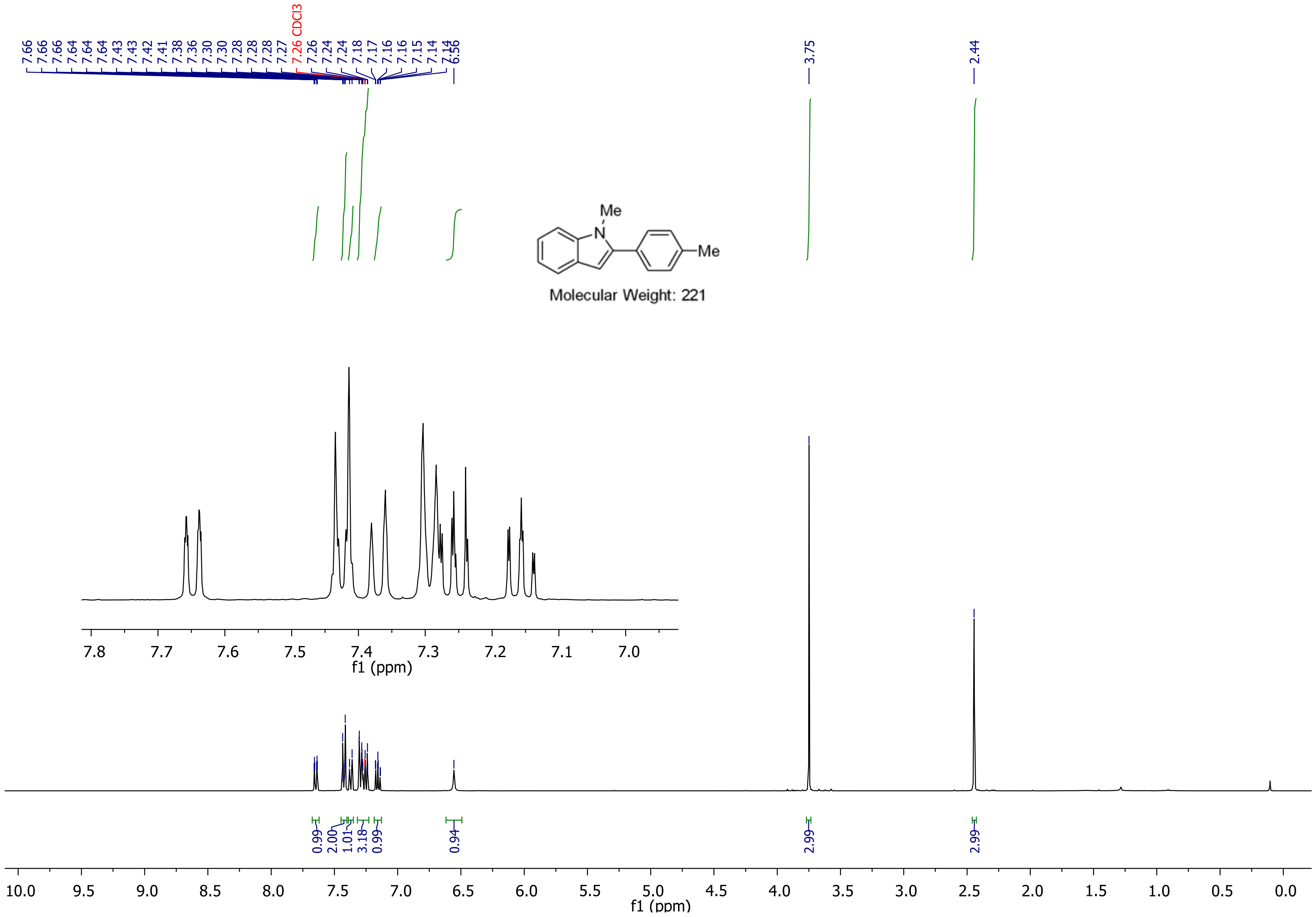

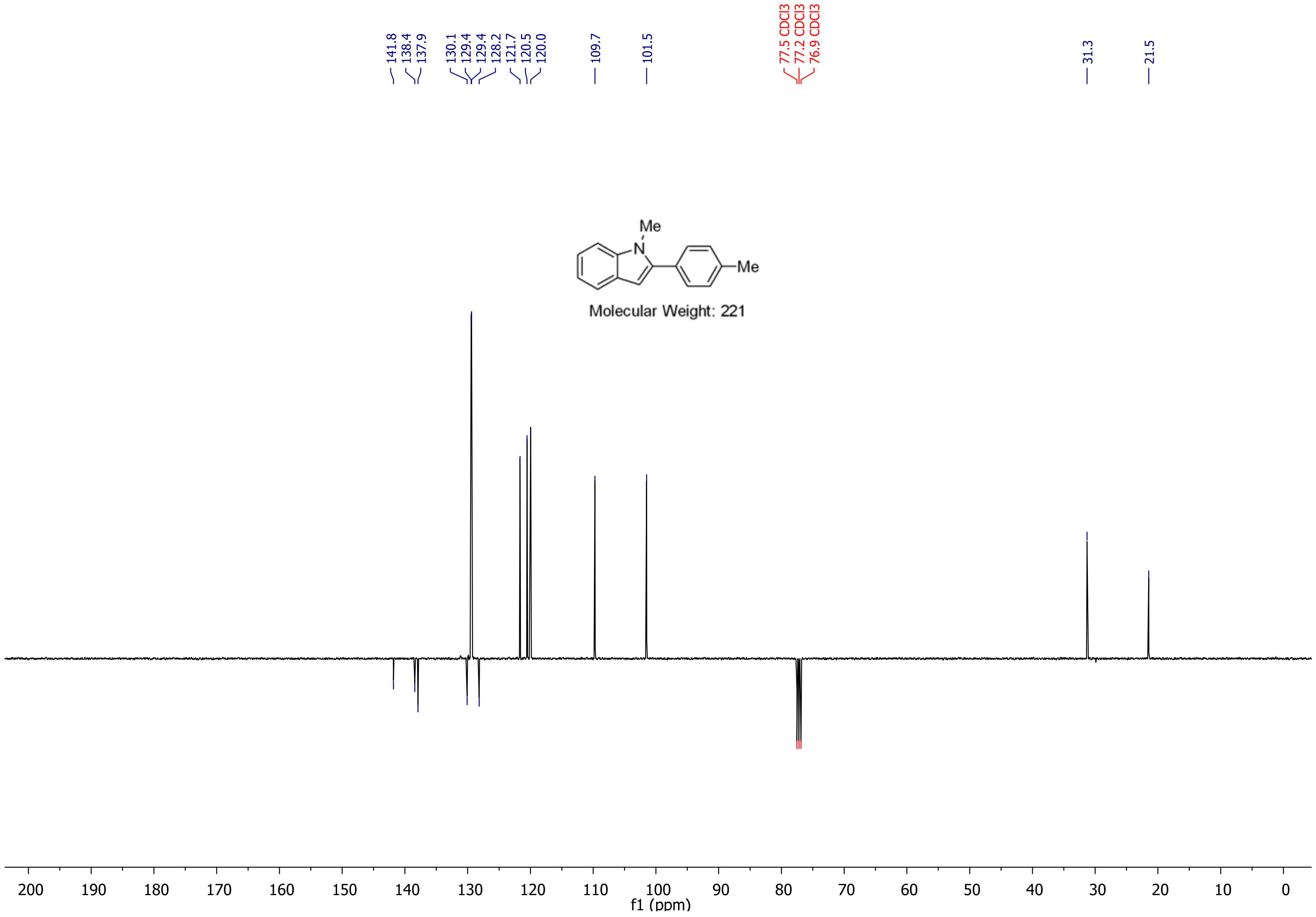
141.8
138.4
137.9
130.1
129.4
129.4
128.2
121.7
120.5
120.0
109.7
101.5
77.5 CDCl3
77.2 CDCl3
76.9 CDCl3
31.3
21.5
Me
N
Me
Molecular Weight: 221
200
190
180
170
160
150
140
130
120
110
100
90
80
70
60
50
40
30
20
10
0
f1 (ppm)

2-(2',4'-dimethylphenyl)-1-methyl-1*H*-indole (3e)

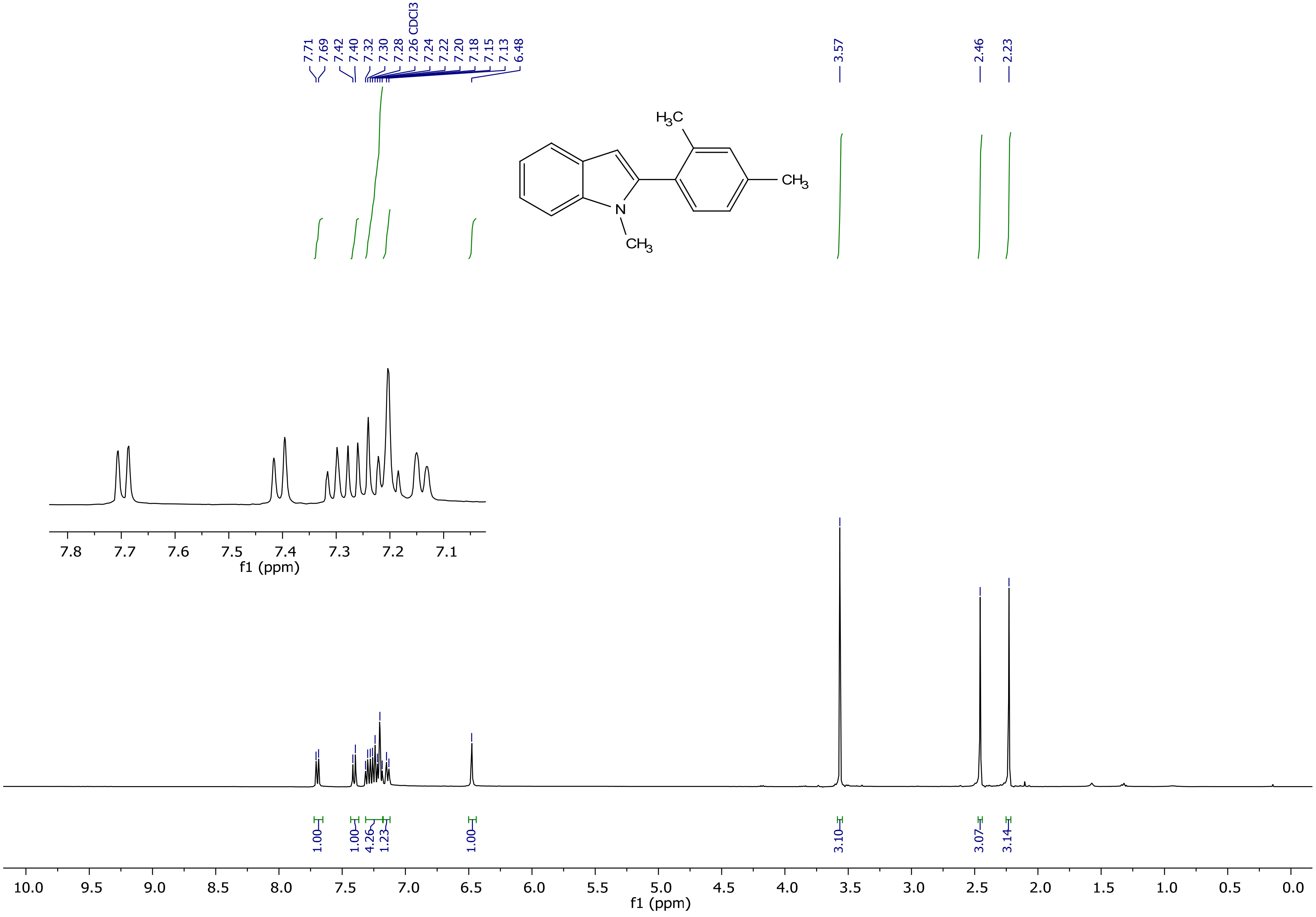

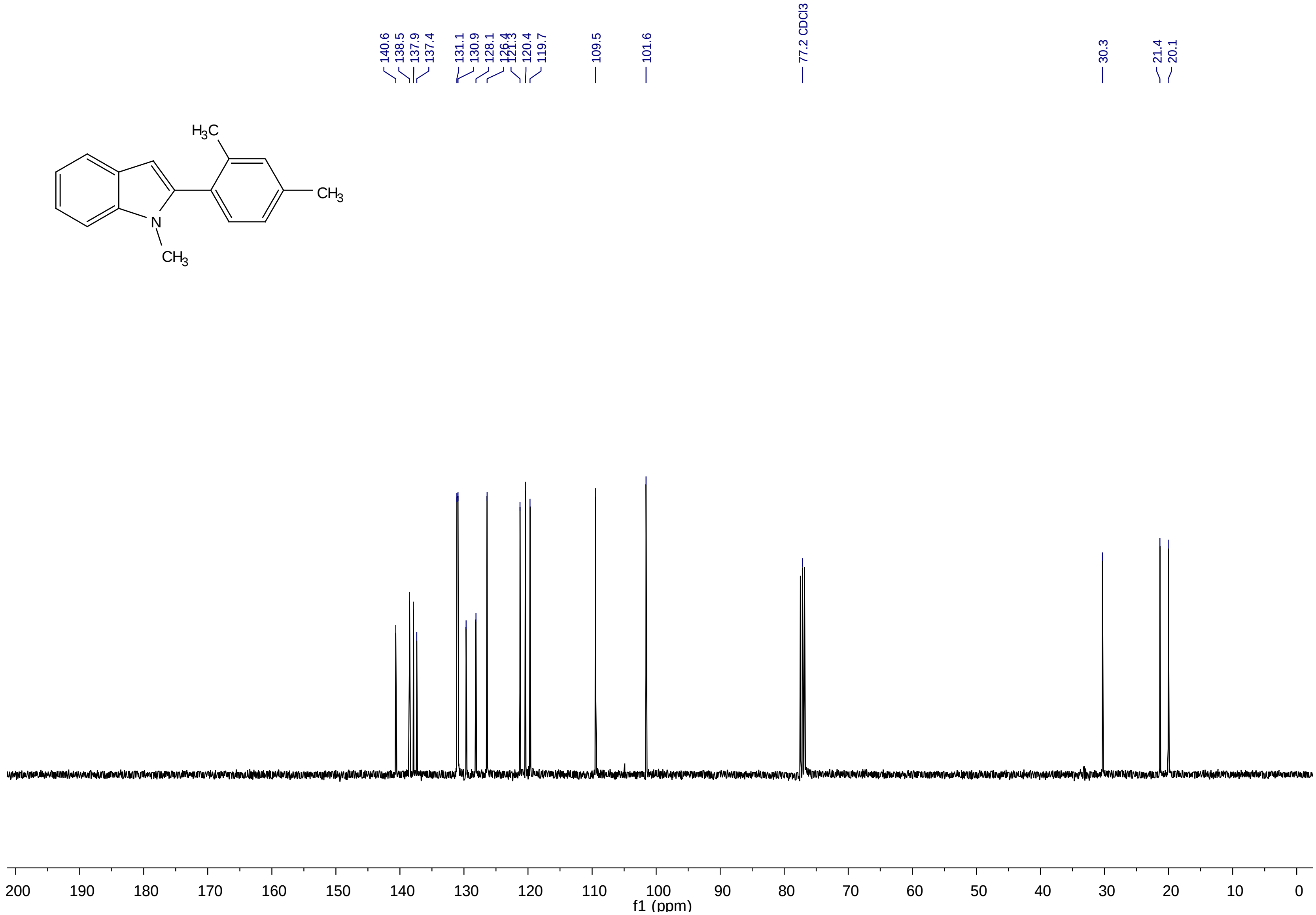
140.6
138.5
137.9
137.4
131.1
130.9
128.1
126.4
121.3
120.4
119.7
109.5
101.6
77.2 CDCl3
30.3
21.4
20.1
H3C
CH3
N
CH3
200
190
180
170
160
150
140
130
120
110
100
90
80
70
60
50
40
30
20
10
0
f1 (ppm)

2-mesityl-1-methyl-1*H*-indole (3f – C-2)

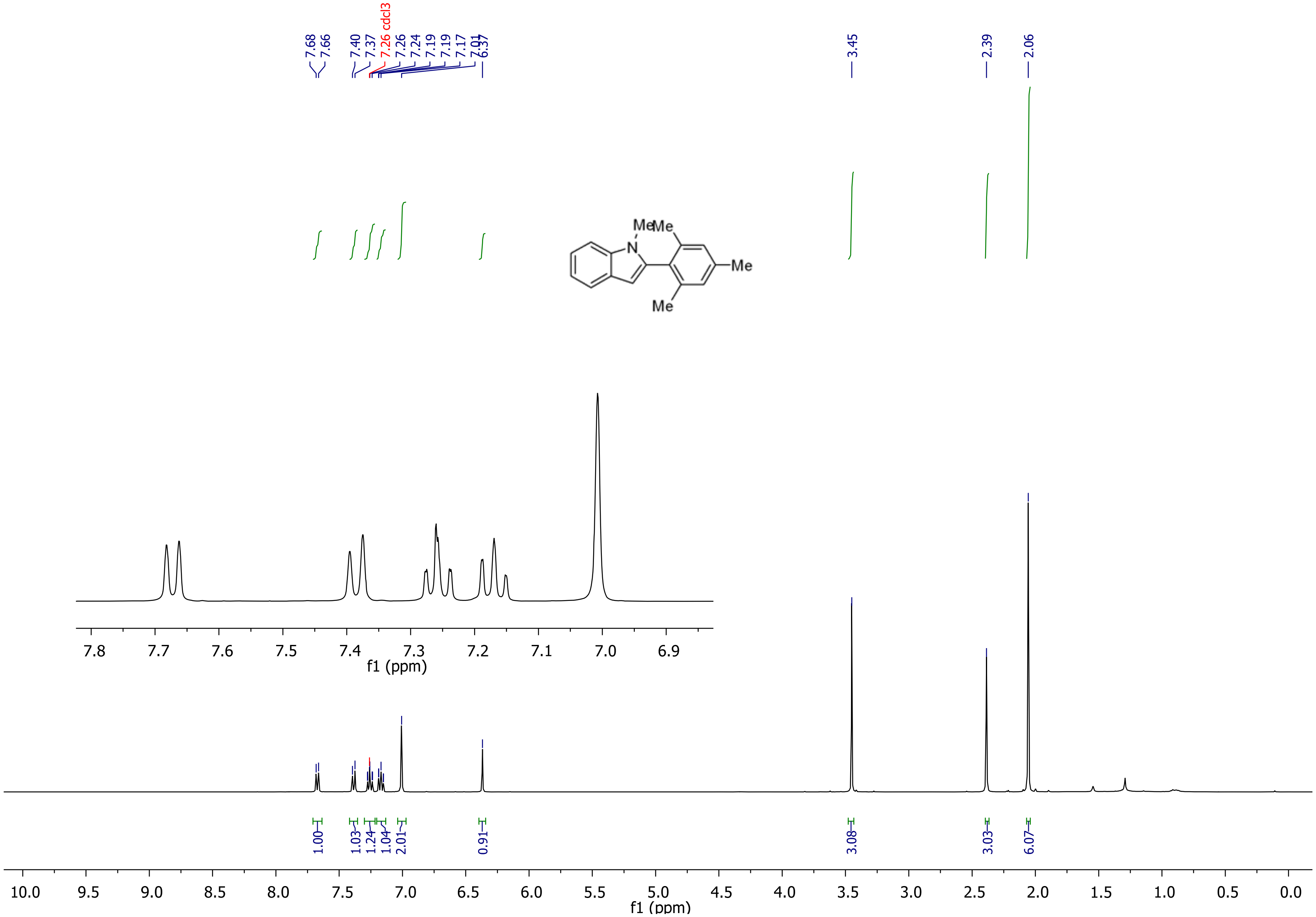

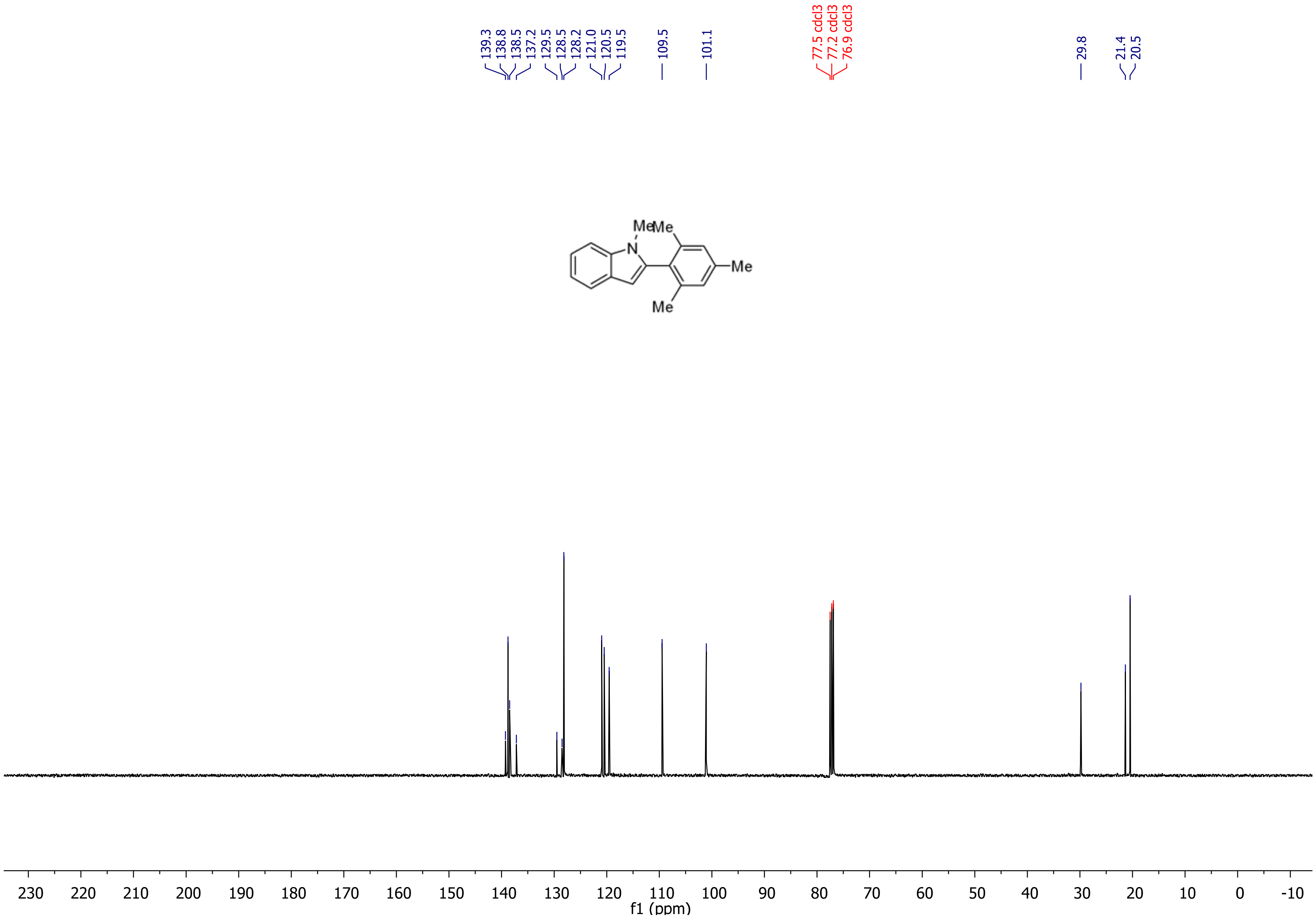
139.3
138.8
138.5
137.2
129.5
128.5
128.2
121.0
120.5
119.5
109.5
101.1
77.5 cdcl3
77.2 cdcl3
76.9 cdcl3
29.8
21.4
20.5
Me
Me
N
Me
Me
230 220 210 200 190 180 170 160 150 140 130 120 110 100 90 80 70 60 50 40 30 20 10 0 -10
f1 (ppm)

3-mesityl-1-methyl-1*H*-indole (3f – C-3)

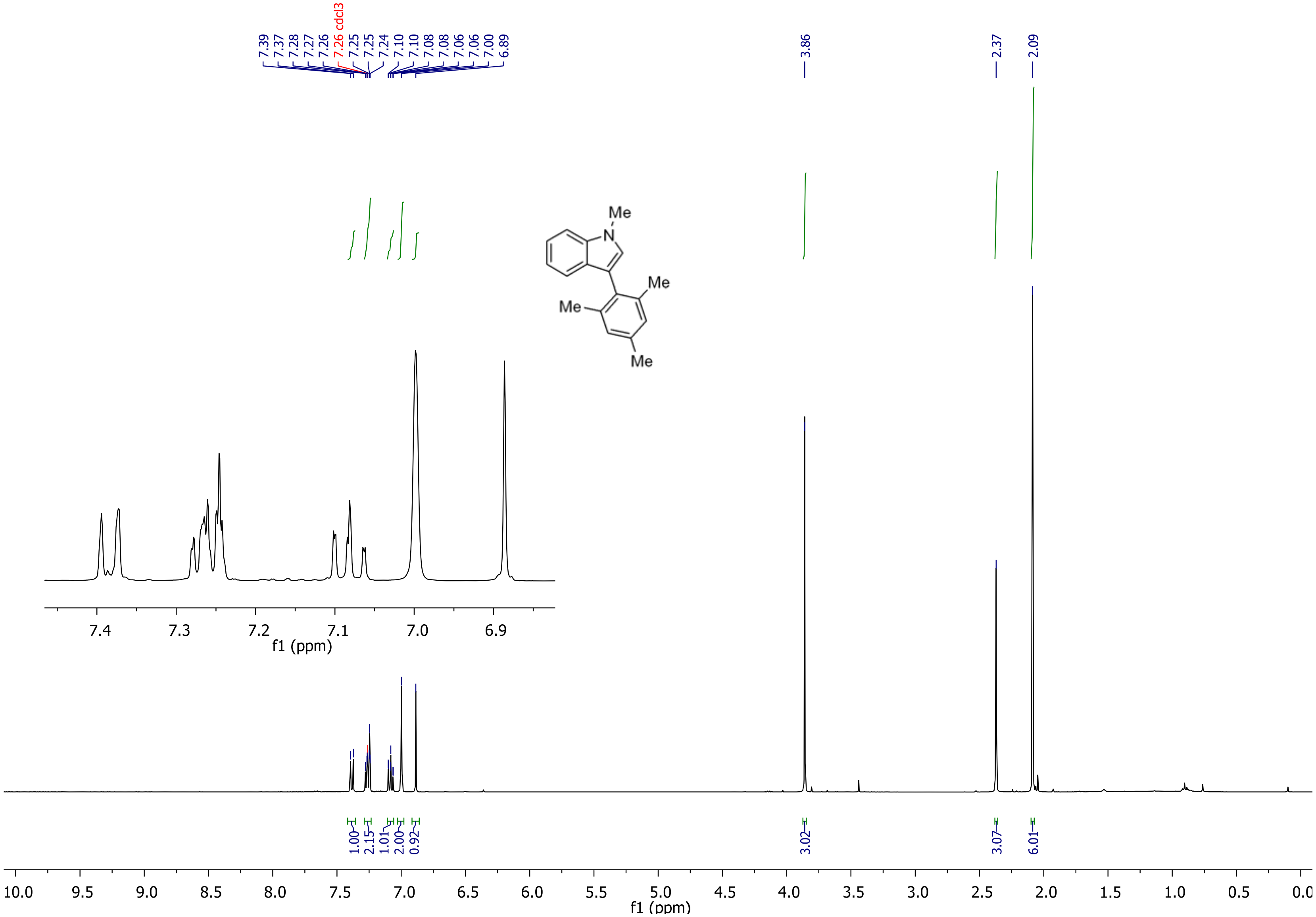

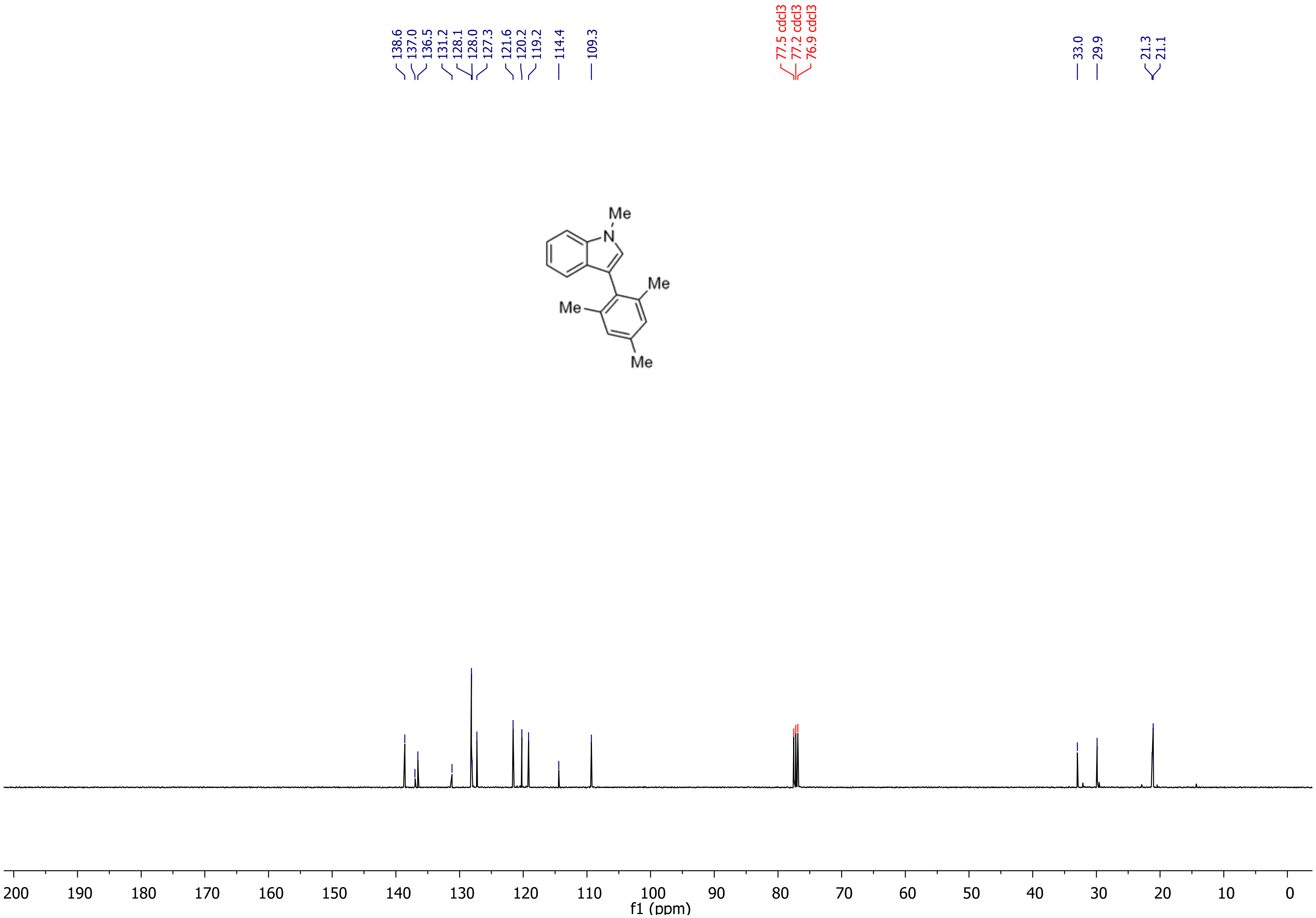
138.6
137.0
136.5
131.2
128.1
128.0
127.3
121.6
120.2
119.2
114.4
109.3
77.5 cdcl3
77.2 cdcl3
76.9 cdcl3
33.0
29.9
21.3
21.1
Me
N
Me
Me
Me
200 190 180 170 160 150 140 130 120 110 100 90 80 70 60 50 40 30 20 10 0
f1 (ppm)

2-(4-(*tert*-butyl)phenyl)-1-methyl-1*H*-indole (3g)

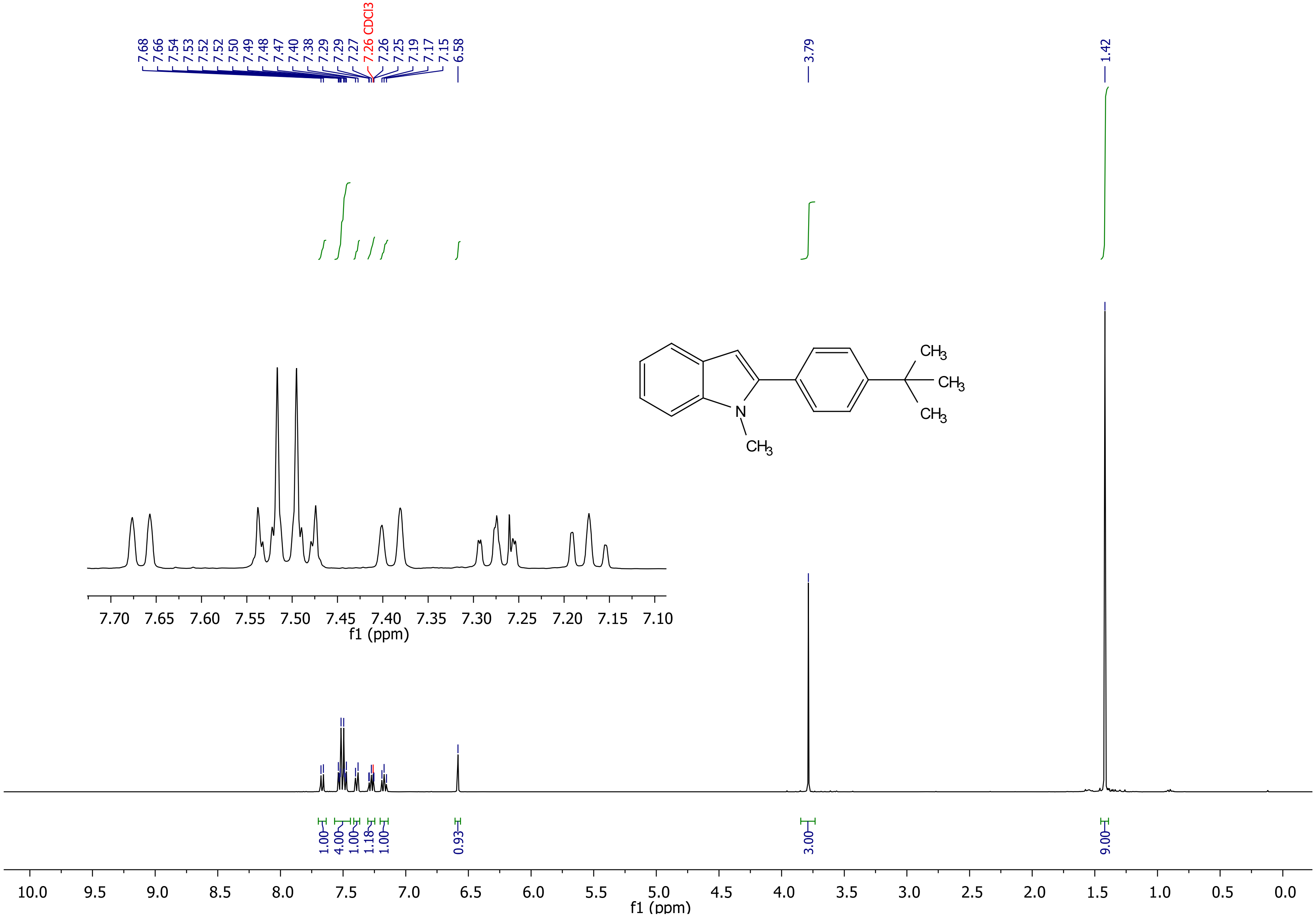

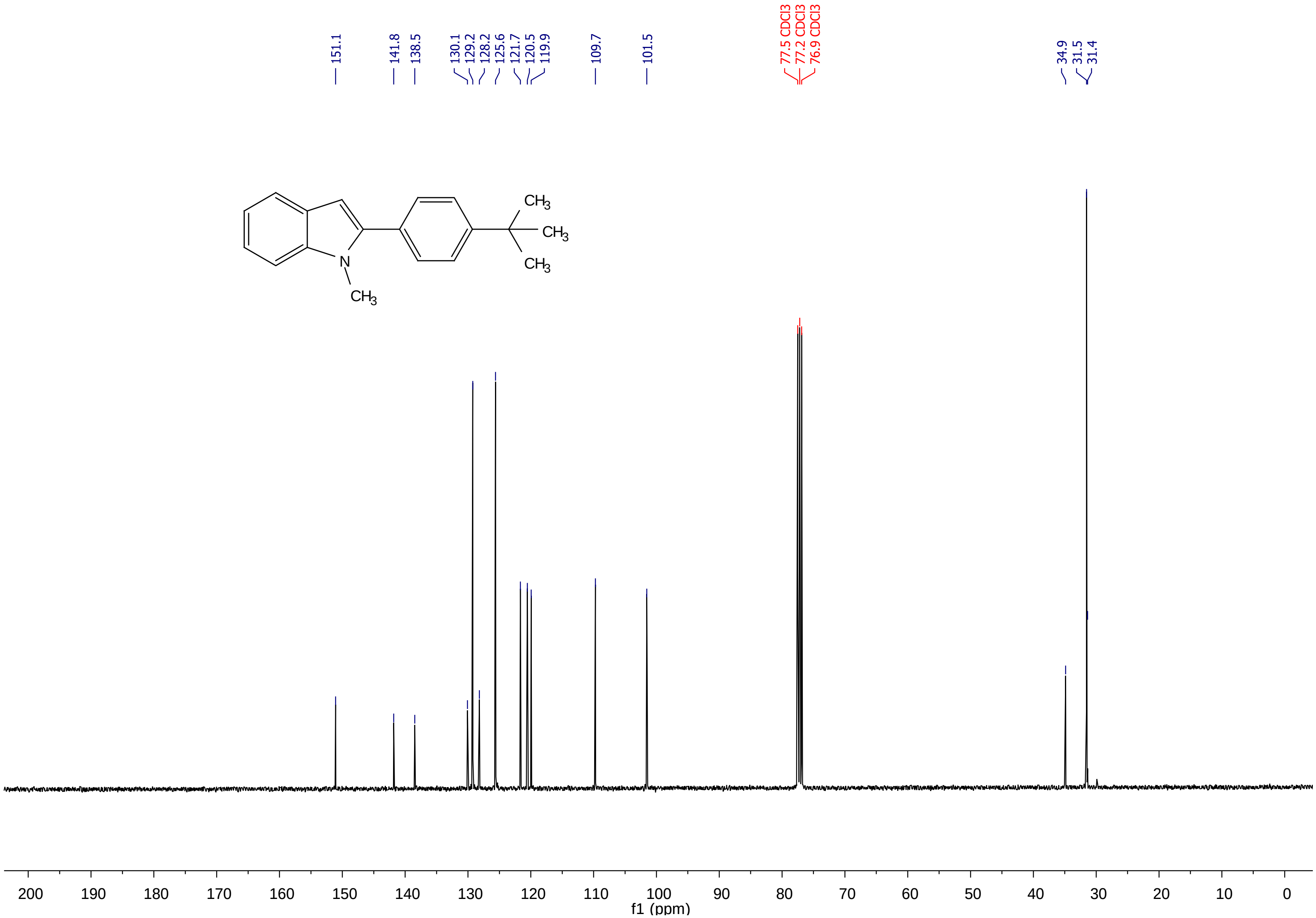
151.1
141.8
138.5
130.1
129.2
128.2
125.6
121.7
120.5
119.9
109.7
101.5
77.5 CDCl3
77.2 CDCl3
76.9 CDCl3
34.9
31.5
31.4
CH3
CH3
CH3
N
CH3
200
190
180
170
160
150
140
130
120
110
100
90
80
70
60
50
40
30
20
10
0
f1 (ppm)

2-(4-methoxyphenyl)-1-methyl-*1H*-indole (3h)

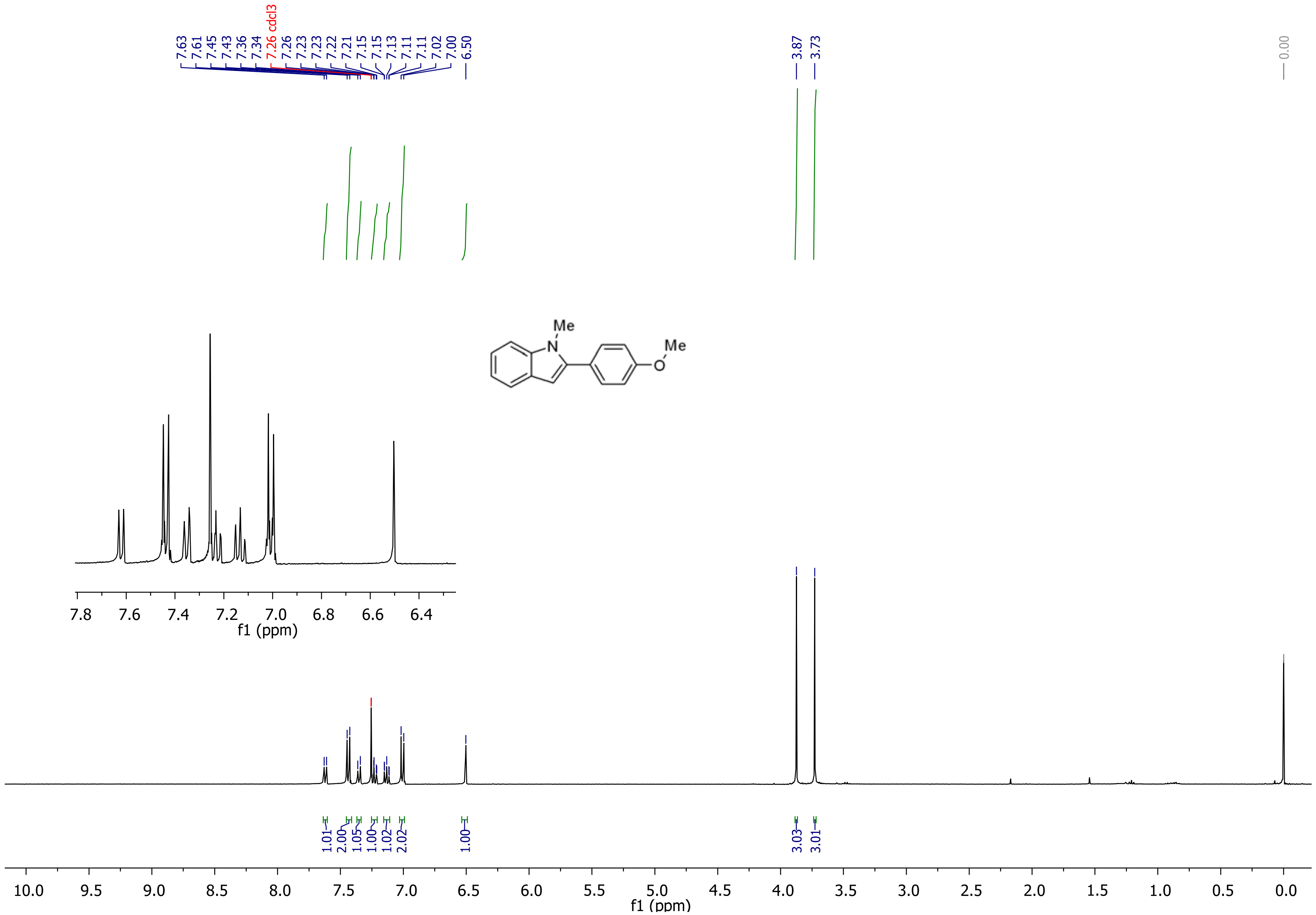

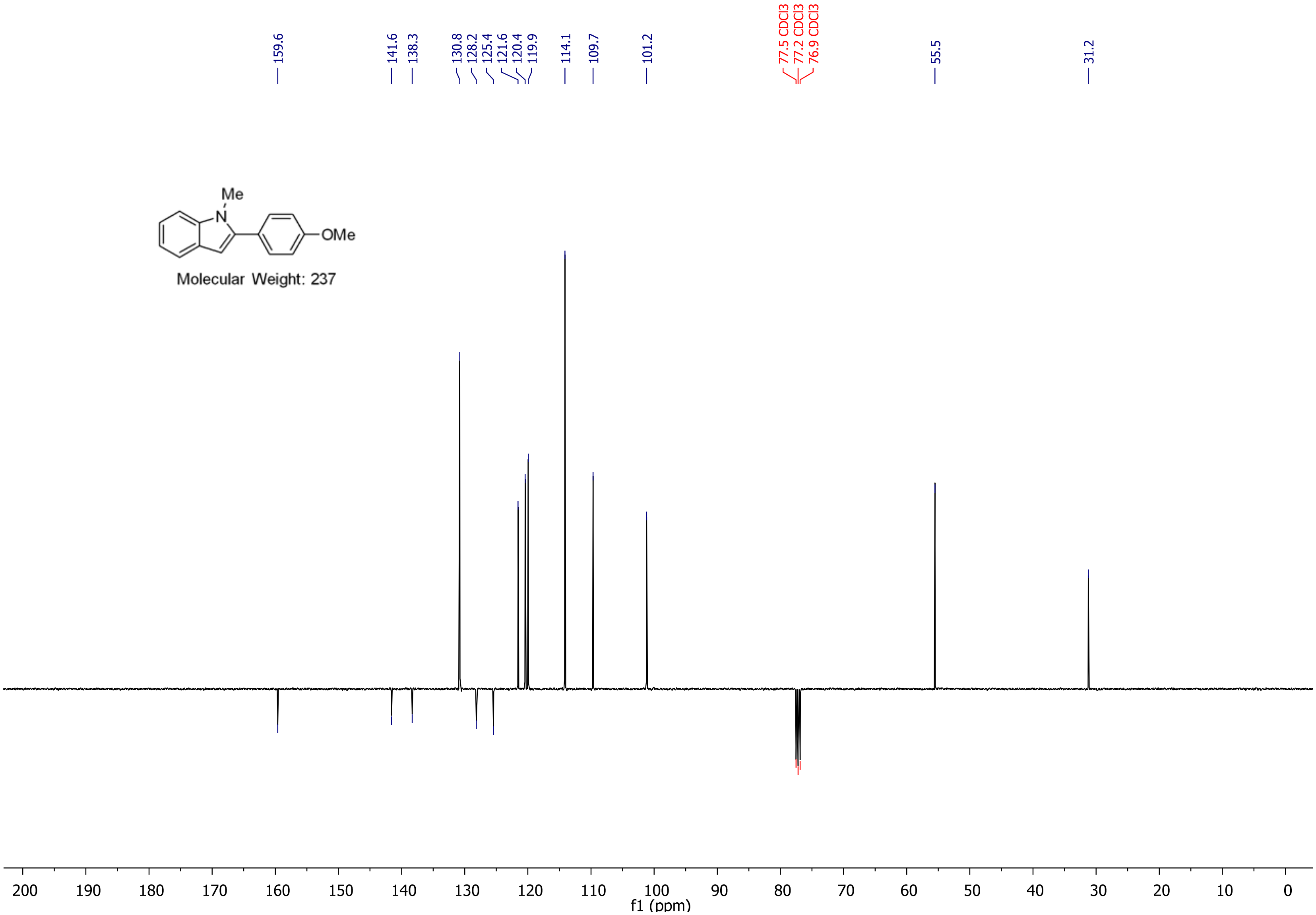
159.6
141.6
138.3
130.8
128.2
125.4
121.6
120.4
119.9
114.1
109.7
101.2
77.5 CDCl3
77.2 CDCl3
76.9 CDCl3
55.5
31.2
Me
N
OMe
Molecular Weight: 237
200
190
180
170
160
150
140
130
120
110
100
90
80
70
60
50
40
30
20
10
0
f1 (ppm)

2-(3-methoxyphenyl)-1-methyl-1*H*-indole (3i)

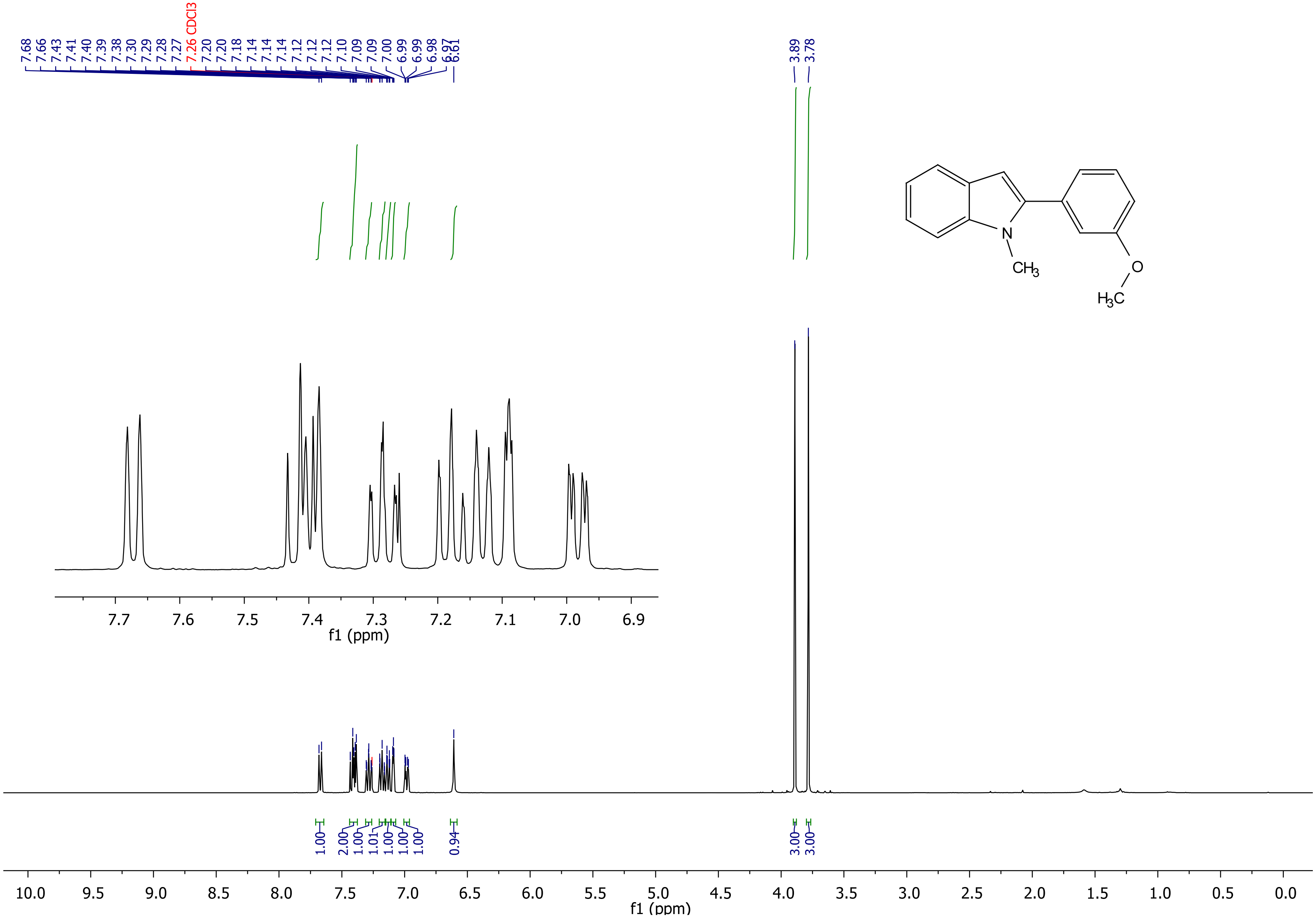

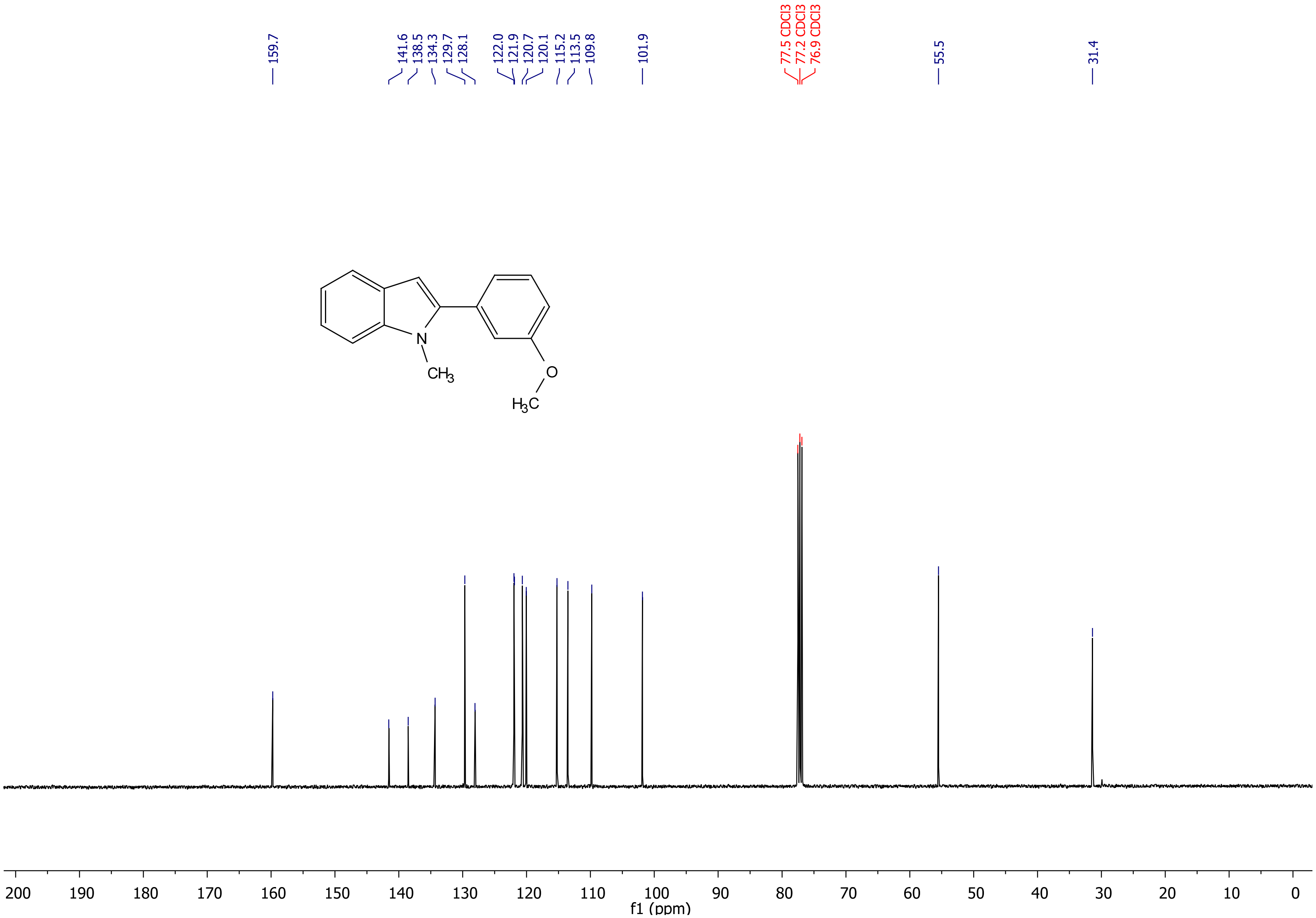
159.7
141.6
138.5
134.3
129.7
128.1
122.0
121.9
120.7
120.1
115.2
113.5
109.8
101.9
77.5 CDCl3
77.2 CDCl3
76.9 CDCl3
55.5
31.4
N
CH3
O
H3C
200
190
180
170
160
150
140
130
120
110
100
90
80
70
60
50
40
30
20
10
0
f1 (ppm)

2-(2-methoxyphenyl)-1-methyl-*1H*-indole (3j)

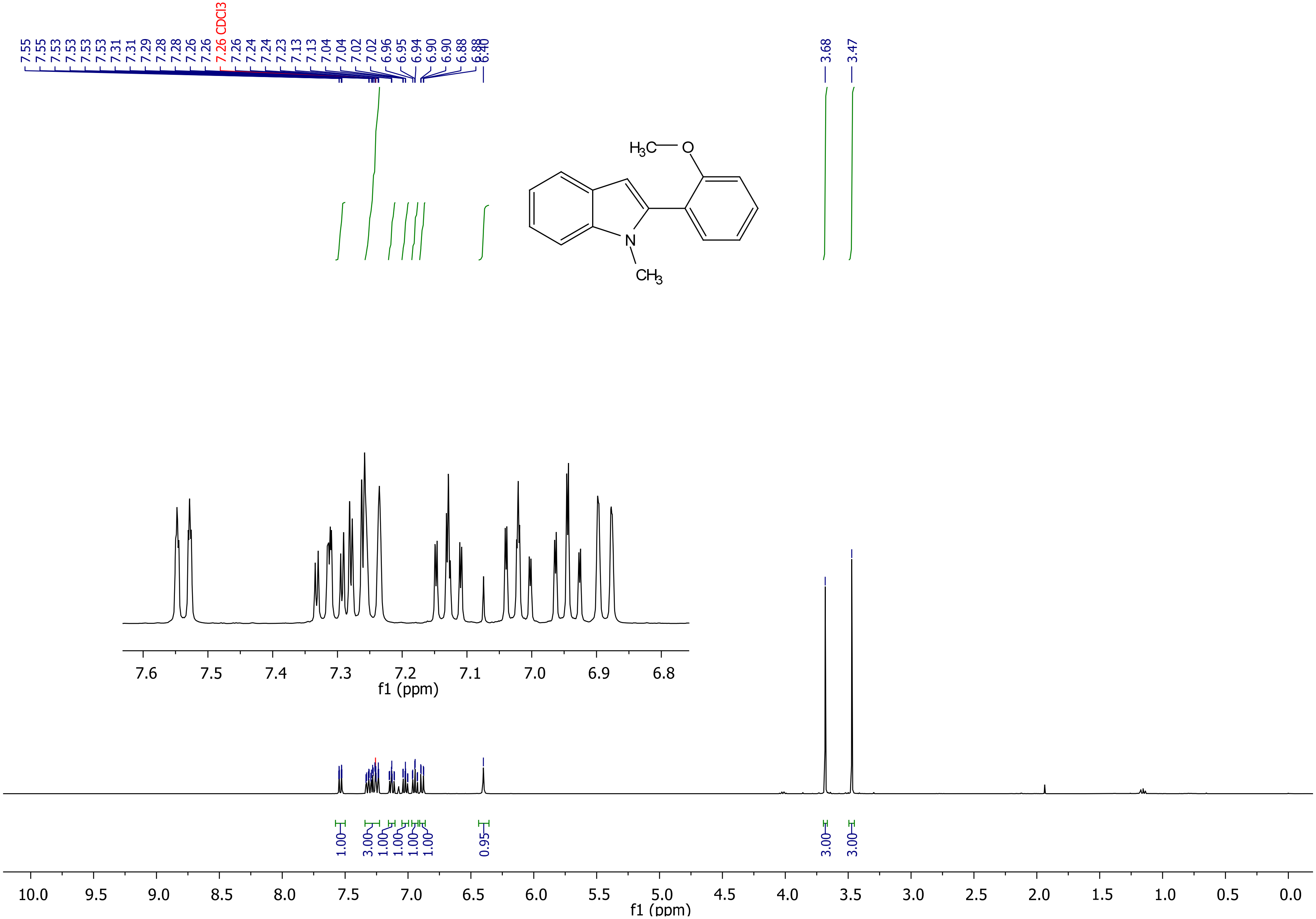

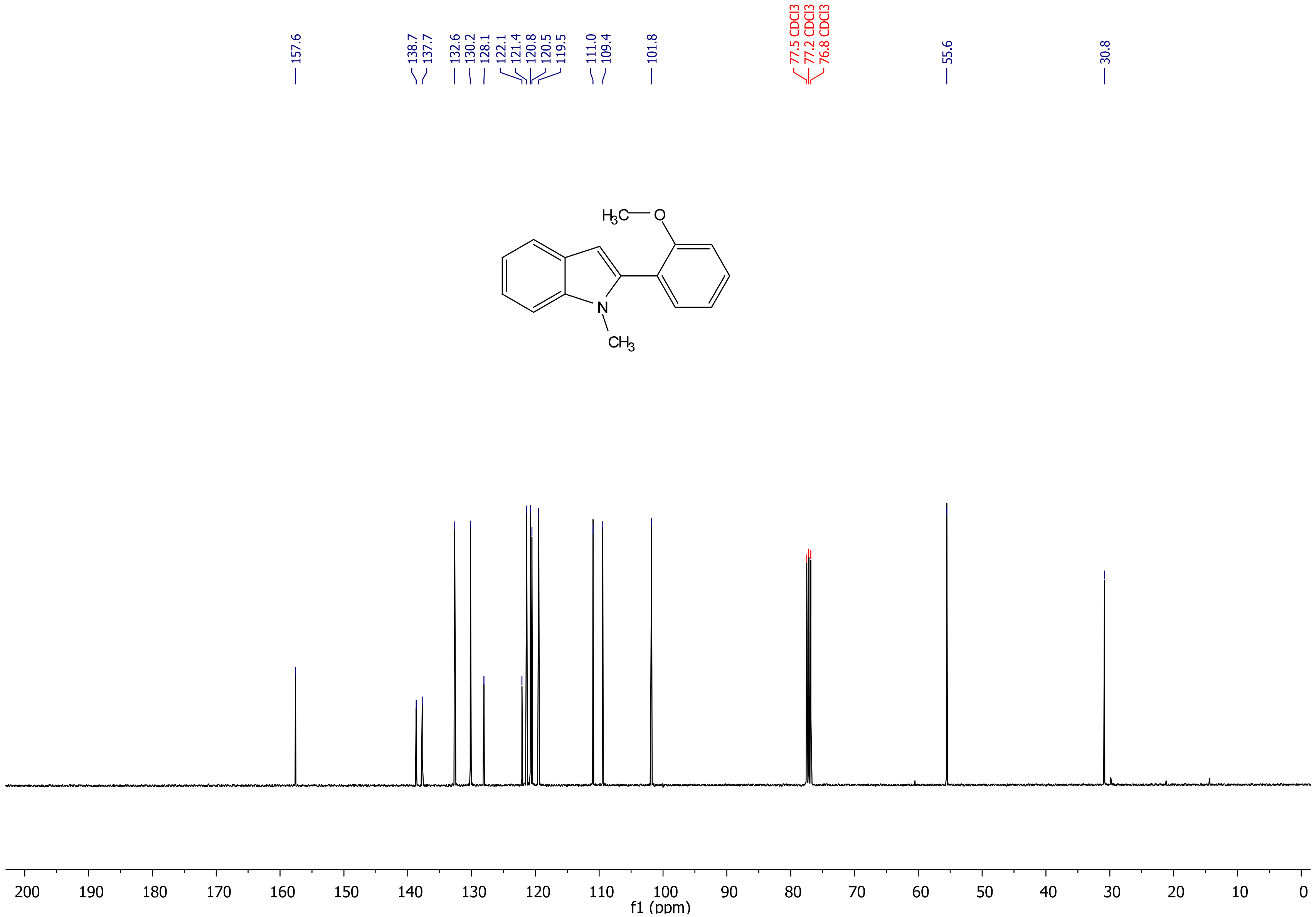
157.6
138.7
137.7
132.6
130.2
128.1
122.1
121.4
120.8
120.5
119.5
111.0
109.4
101.8
77.5 CDCl3
77.2 CDCl3
76.8 CDCl3
55.6
30.8
H3C
O
N
CH3
200
190
180
170
160
150
140
130
120
110
100
90
80
70
60
50
40
30
20
10
0
f1 (ppm)

1-methyl-2-(3,4,5-trimethoxyphenyl)-1*H*-indole (3k)

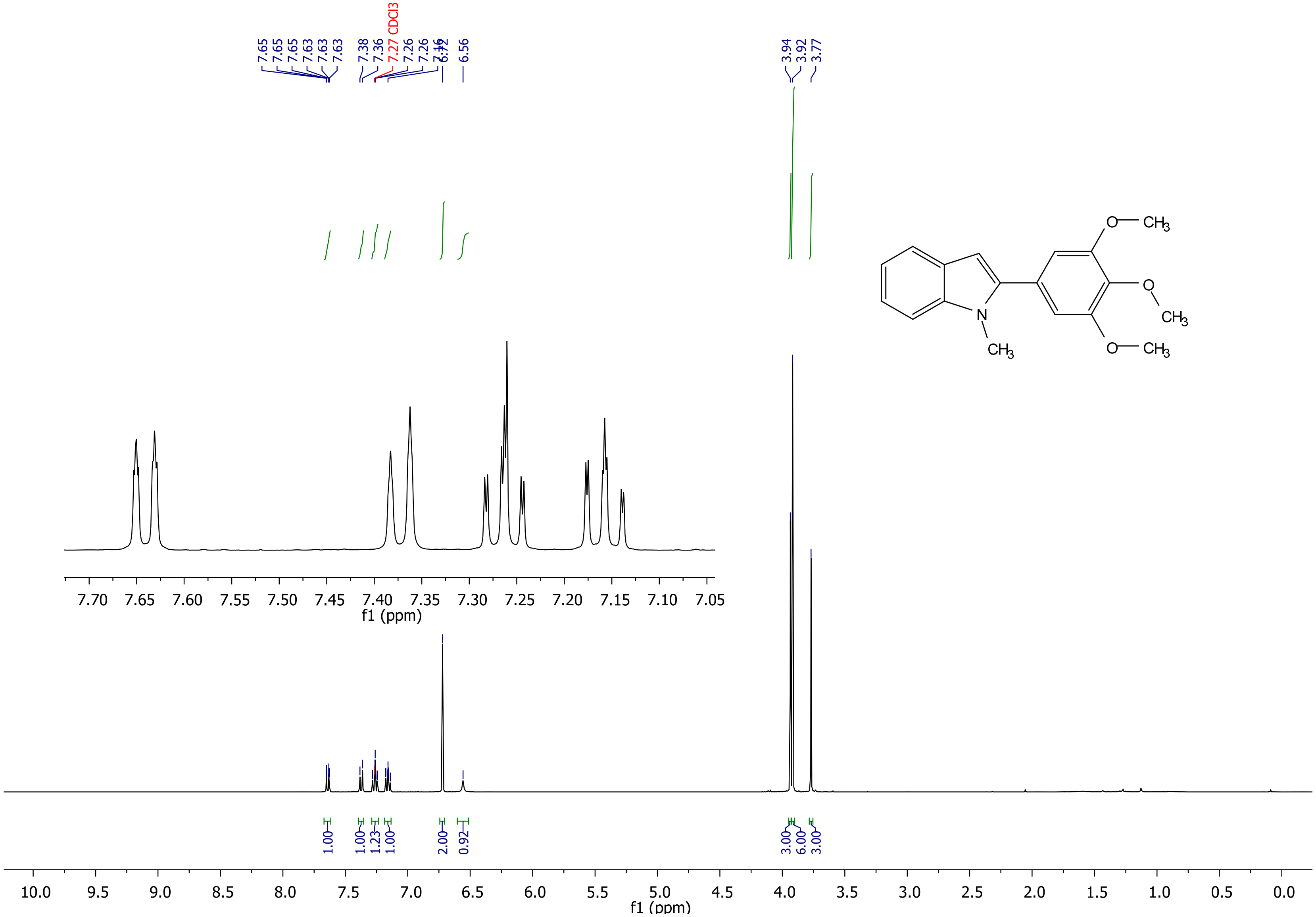

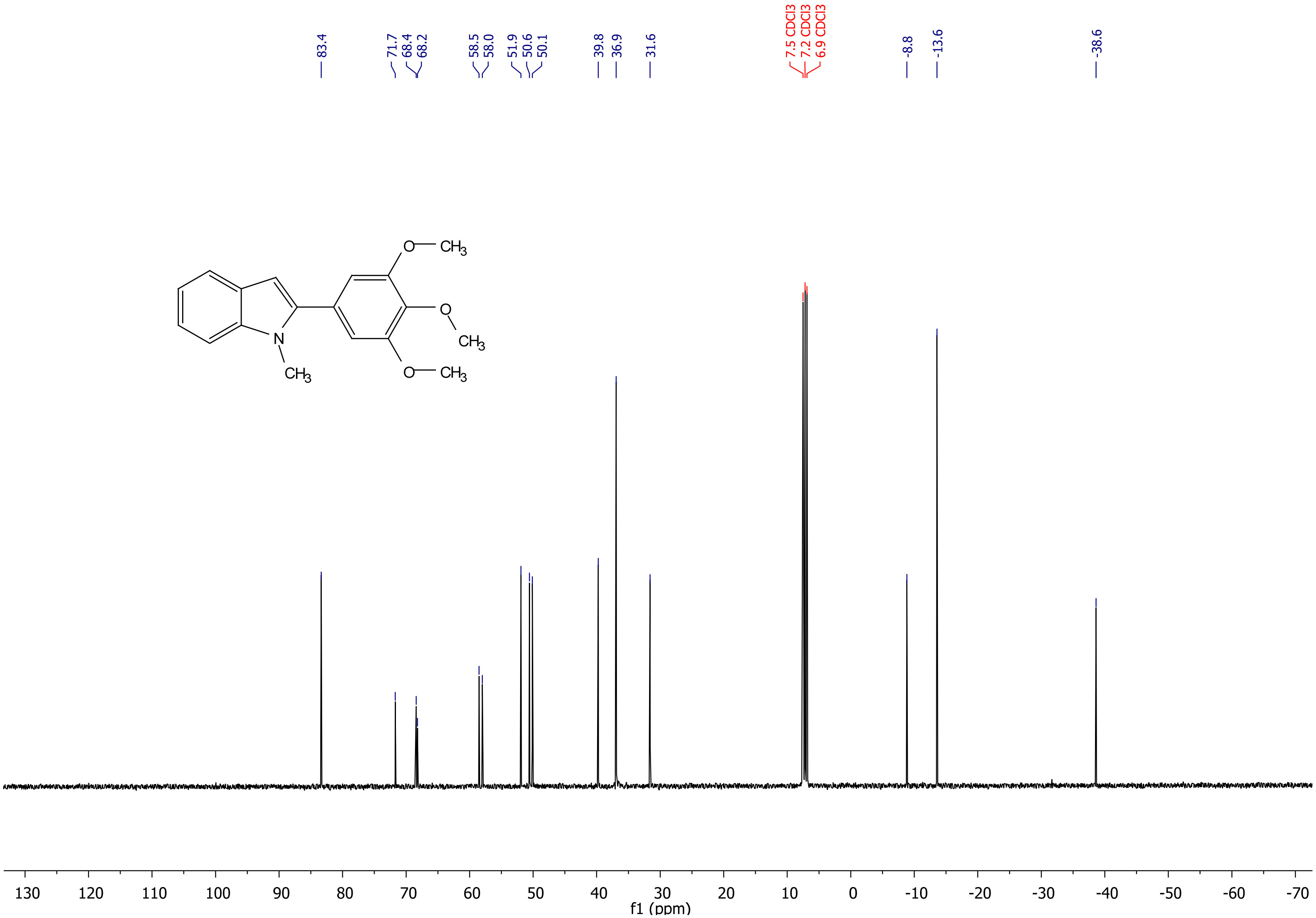
83.4
71.7
68.4
68.2
58.5
58.0
51.9
50.6
50.1
39.8
36.9
31.6
7.5 CDCl3
7.2 CDCl3
6.9 CDCl3
-8.8
-13.6
-38.6
O
CH3
N
130 120 110 100 90 80 70 60 50 40 30 20 10 0 -10 -20 -30 -40 -50 -60 -70
f1 (ppm)

2-(2,4-dimethoxyphenyl)-1-methyl-1*H*-indole (3l)

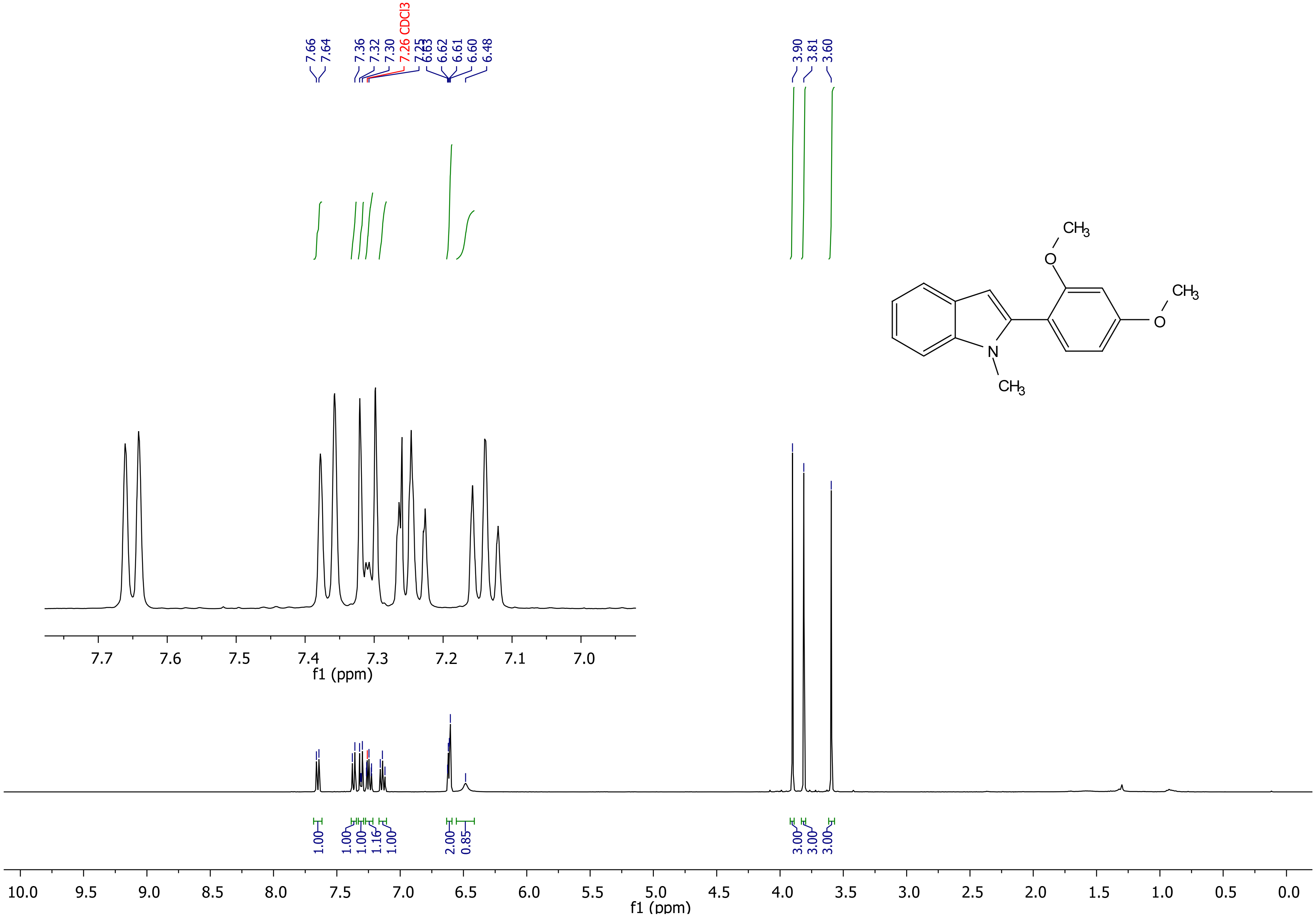

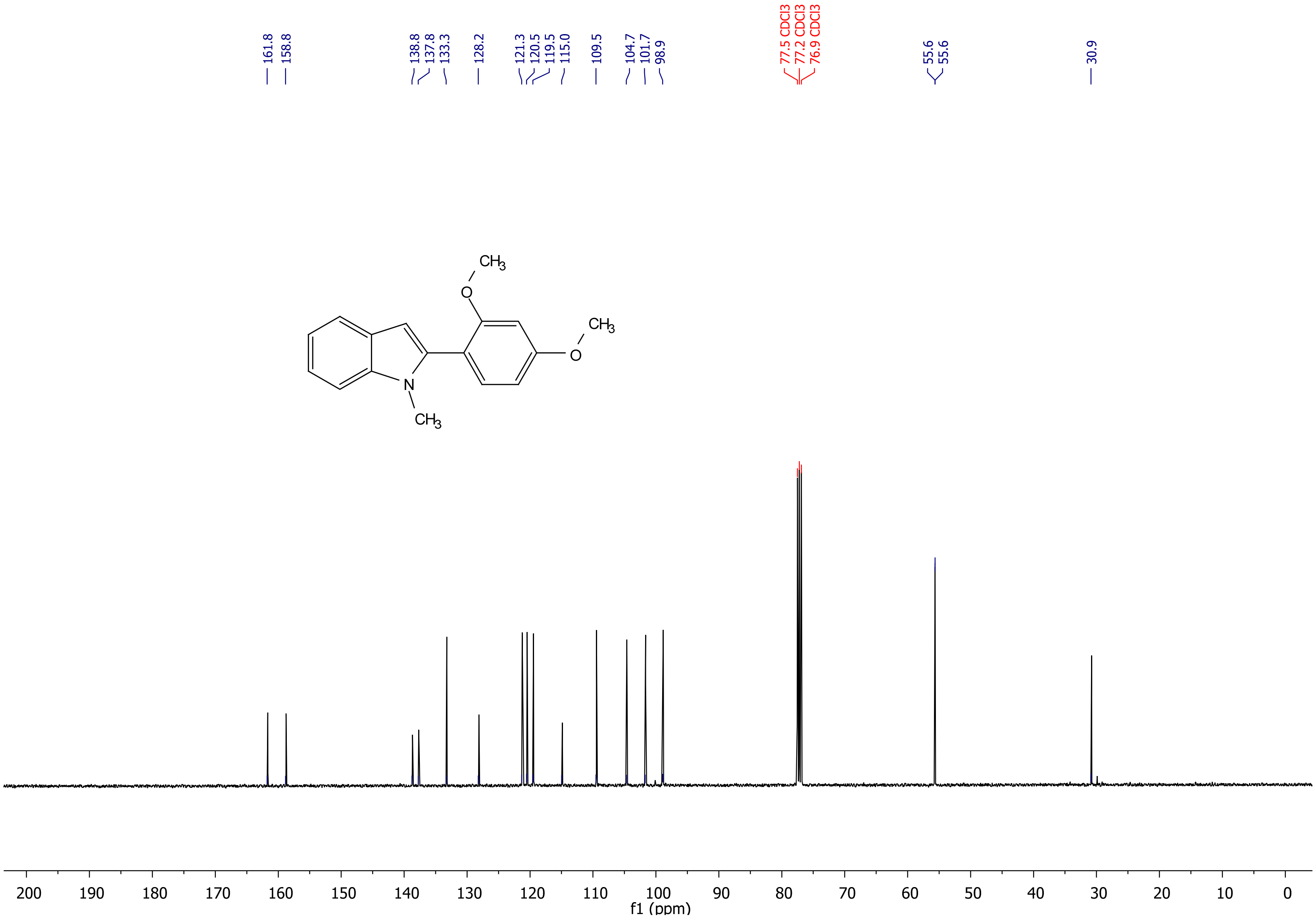

161.8
158.8
138.8
137.8
133.3
128.2
121.3
120.5
119.5
115.0
109.5
104.7
101.7
98.9
77.5 CDCl3
77.2 CDCl3
76.9 CDCl3
55.6
55.6
30.9
CH3
O
N
f1 (ppm)
200
190
180
170
160
150
140
130
120
110
100
90
80
70
60
50
40
30
20
10
0

2-(3-chloro-4-methoxyphenyl)-1-methyl-1*H*-indole (3m)

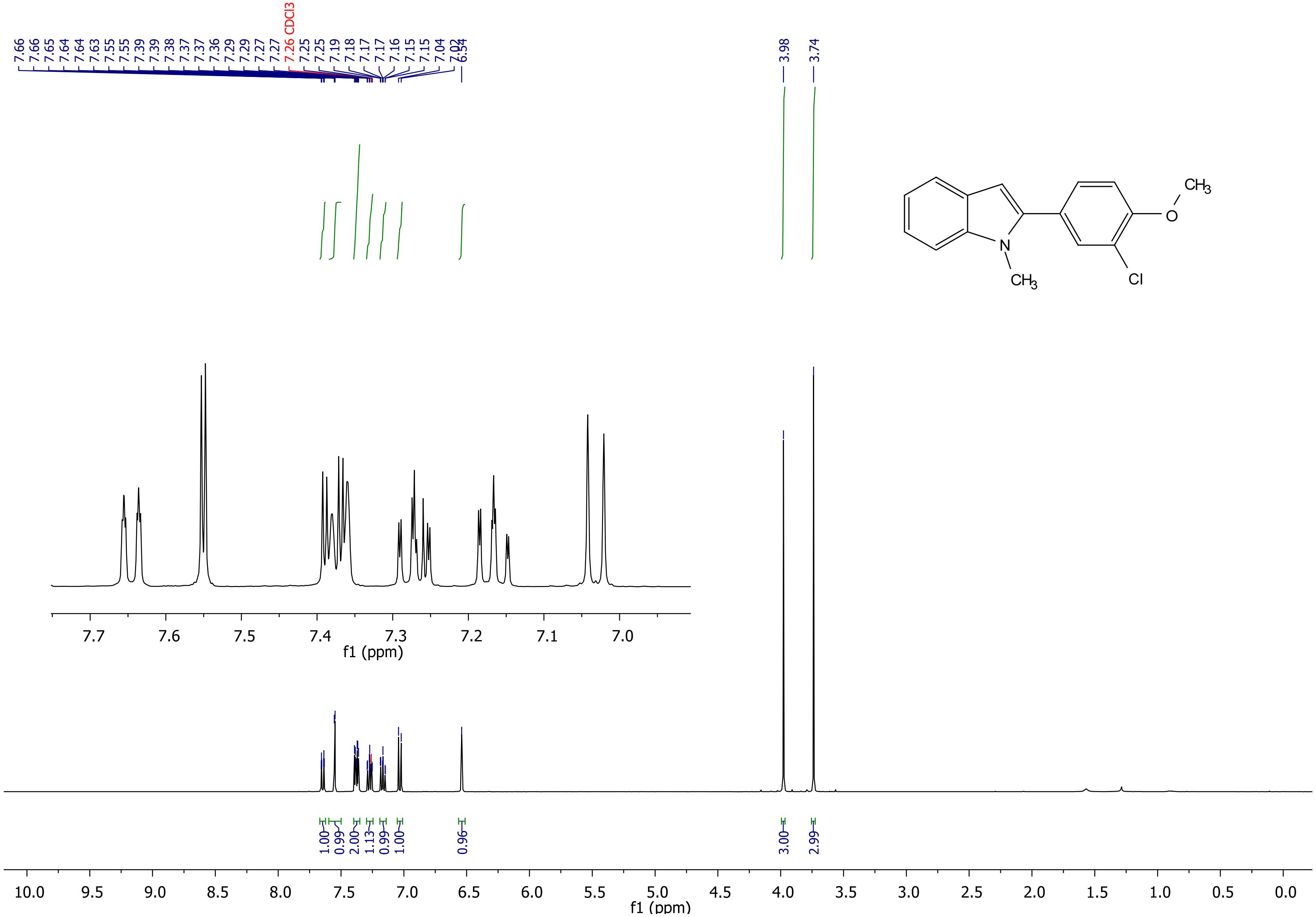

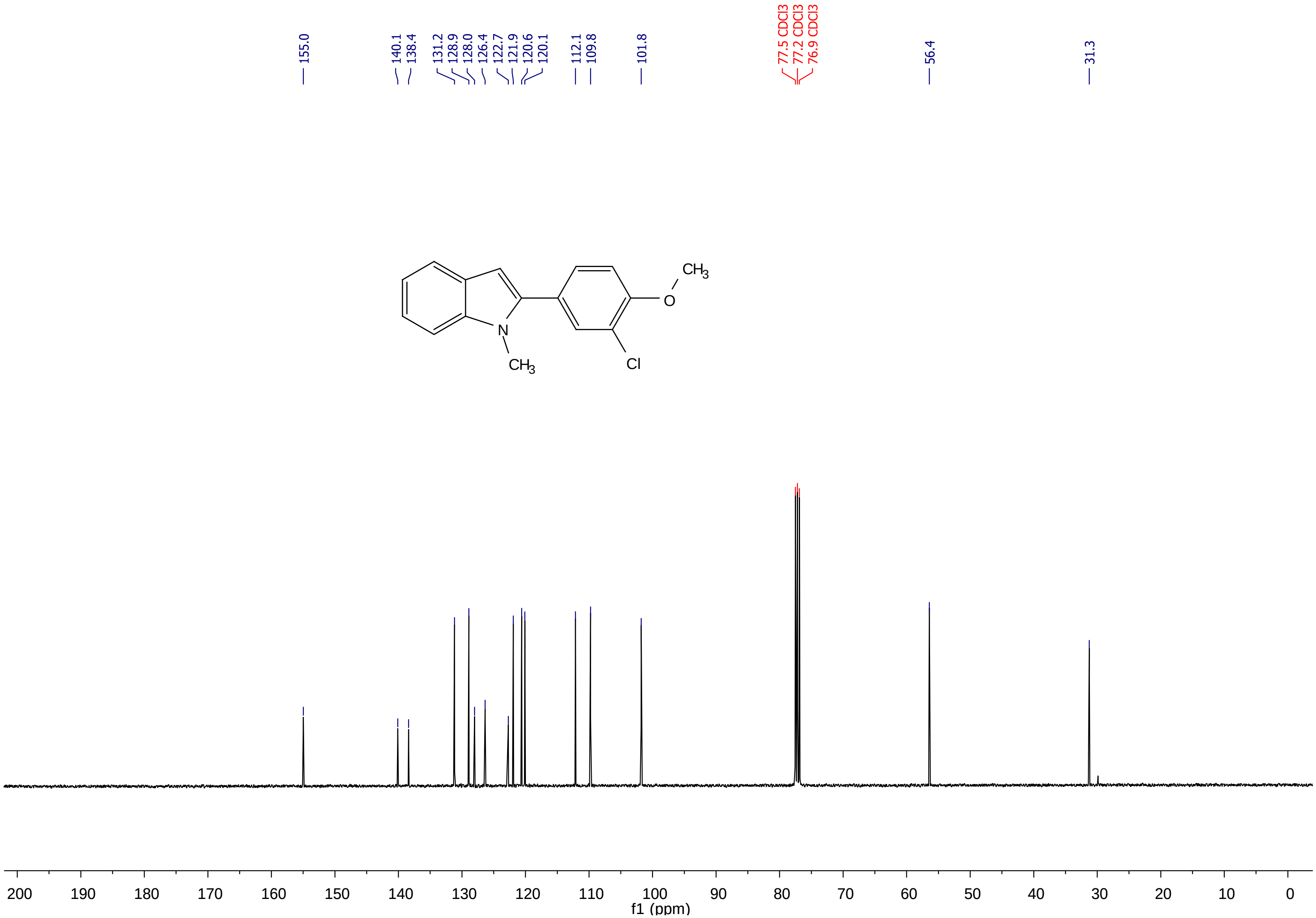
155.0
140.1
138.4
131.2
128.9
128.0
126.4
122.7
121.9
120.6
120.1
112.1
109.8
101.8
77.5 CDCl3
77.2 CDCl3
76.9 CDCl3
56.4
31.3
CH3
O
N
CH3
Cl
200 190 180 170 160 150 140 130 120 110 100 90 80 70 60 50 40 30 20 10 0
f1 (ppm)

2-(4-methoxy-2-methylphenyl)-1-methyl-1*H*-indole (3n)

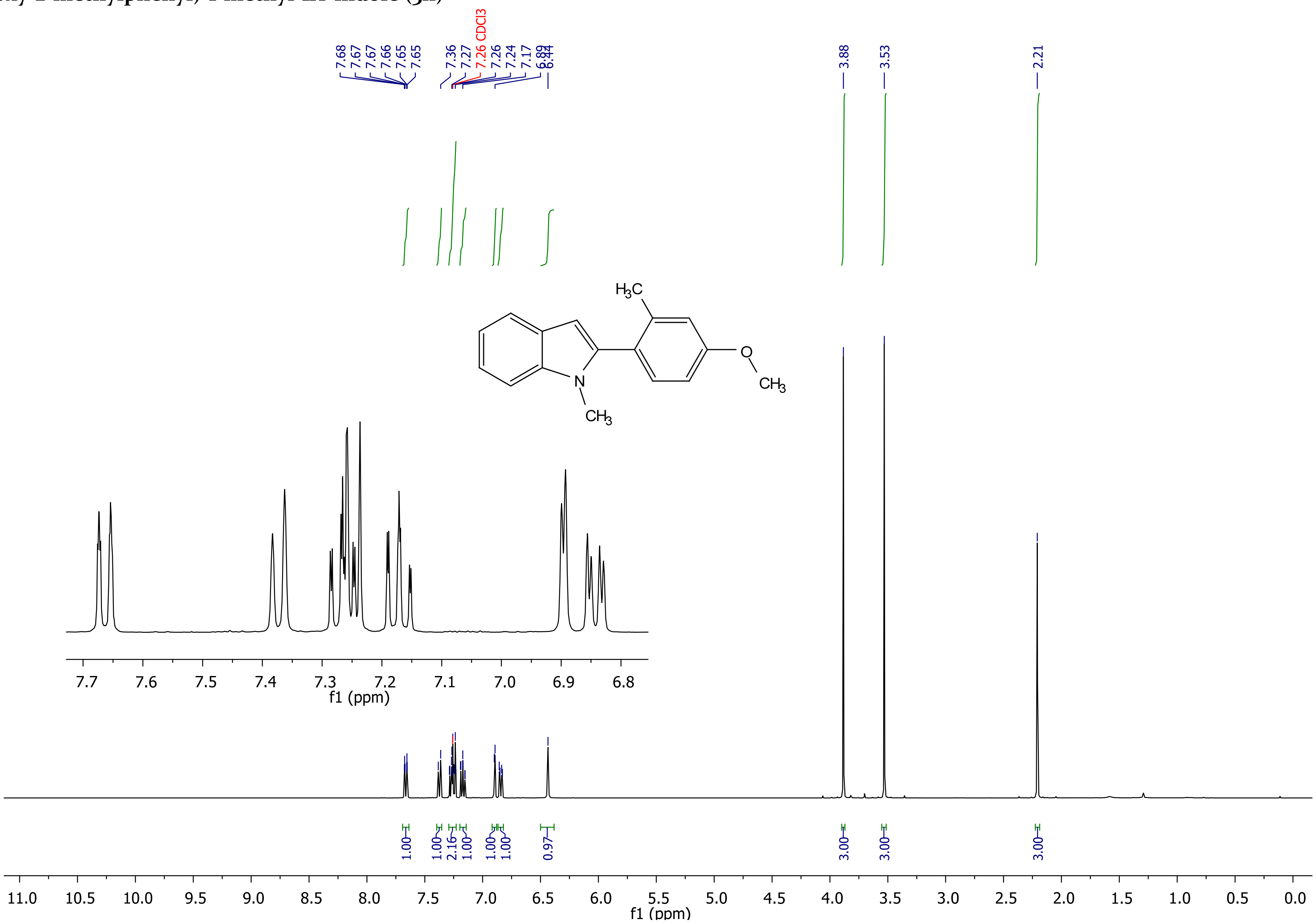

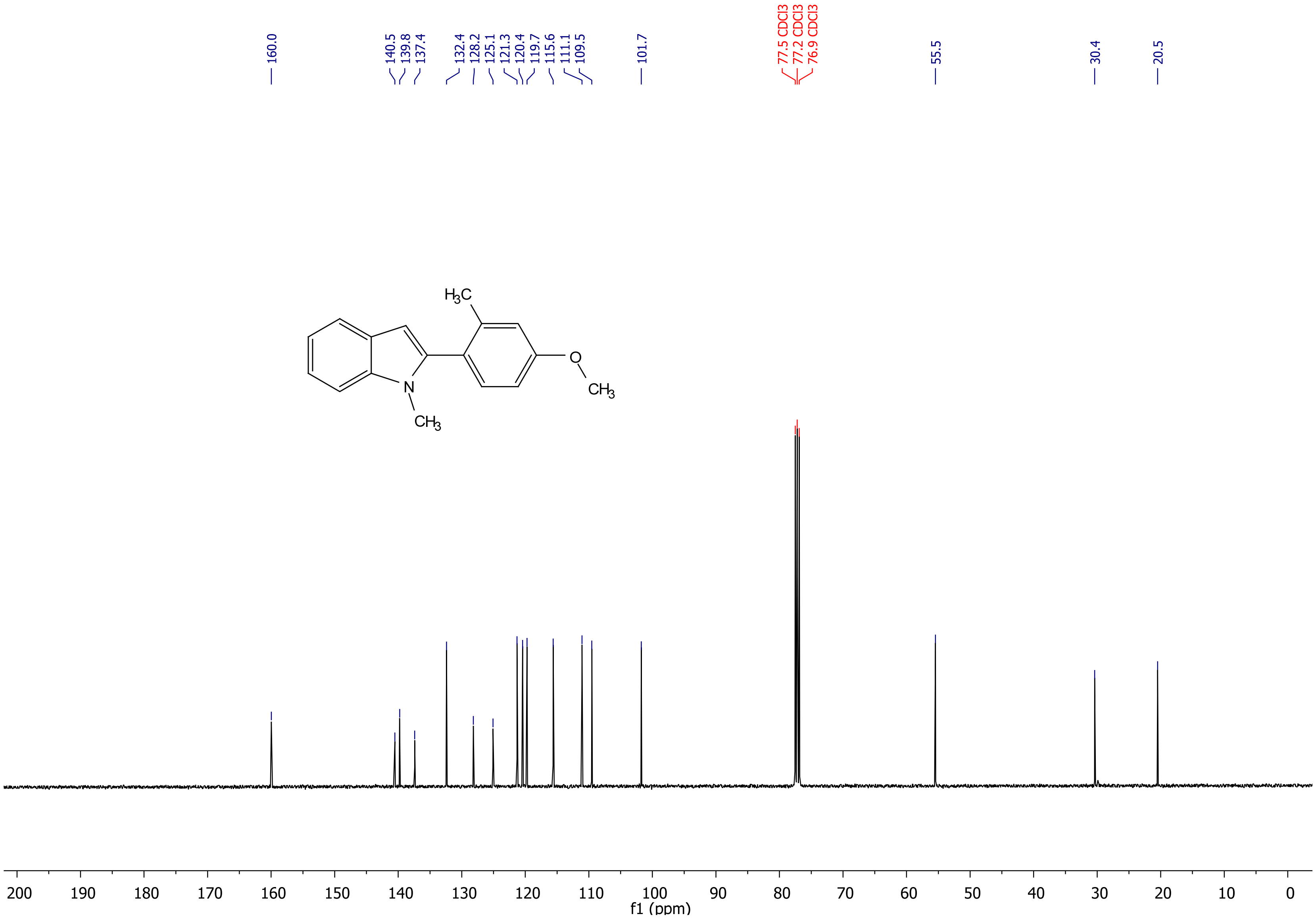

160.0
140.5
139.8
137.4
132.4
128.2
125.1
121.3
120.4
119.7
115.6
111.1
109.5
101.7
77.5 CDCl3
77.2 CDCl3
76.9 CDCl3
55.5
30.4
20.5
H3C
N
CH3
O
CH3
200
190
180
170
160
150
140
130
120
110
100
90
80
70
60
50
40
30
20
10
0
f1 (ppm)

1-methyl -2-(4-phenoxyphenyl)-1*H*-indole (30)

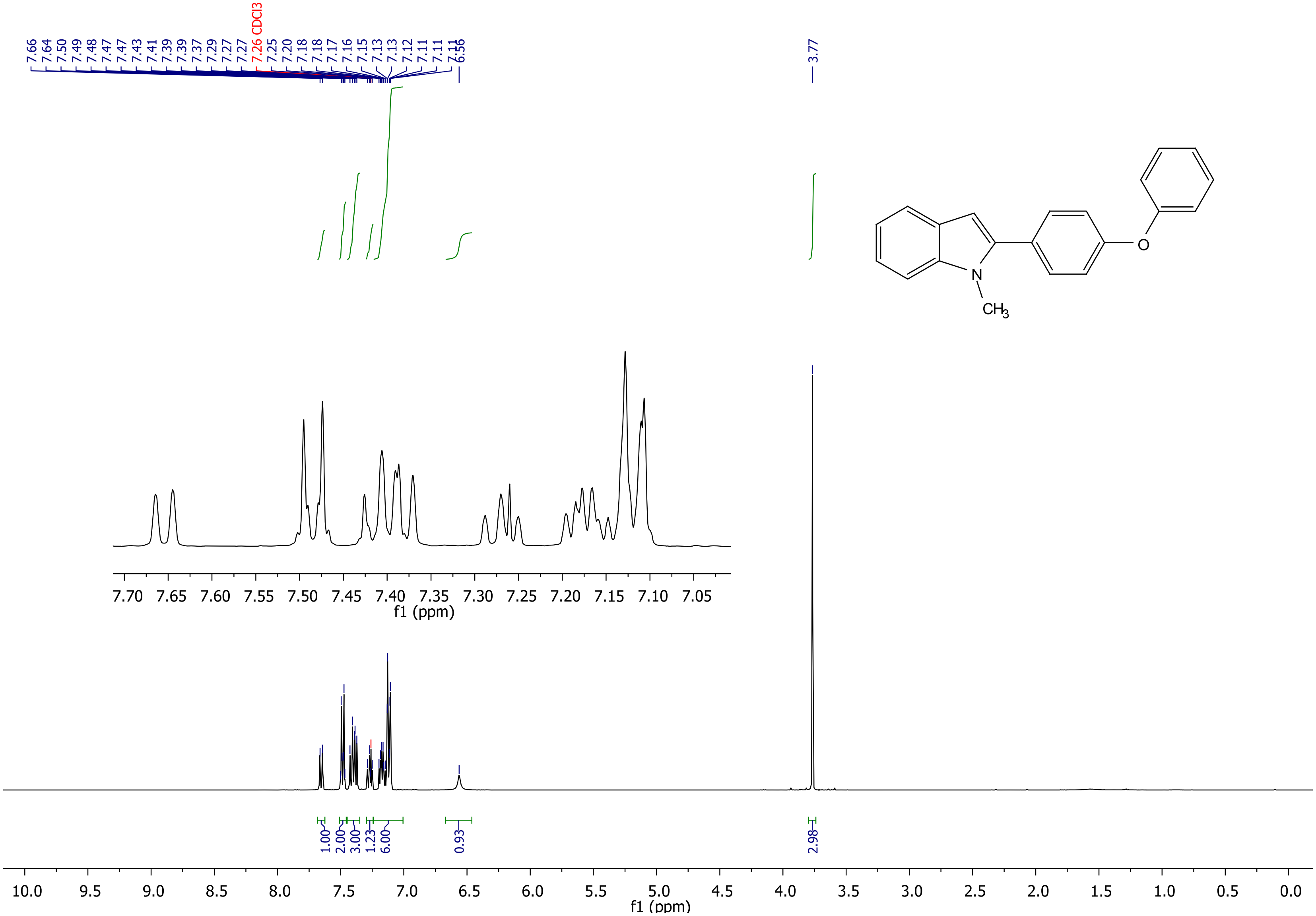

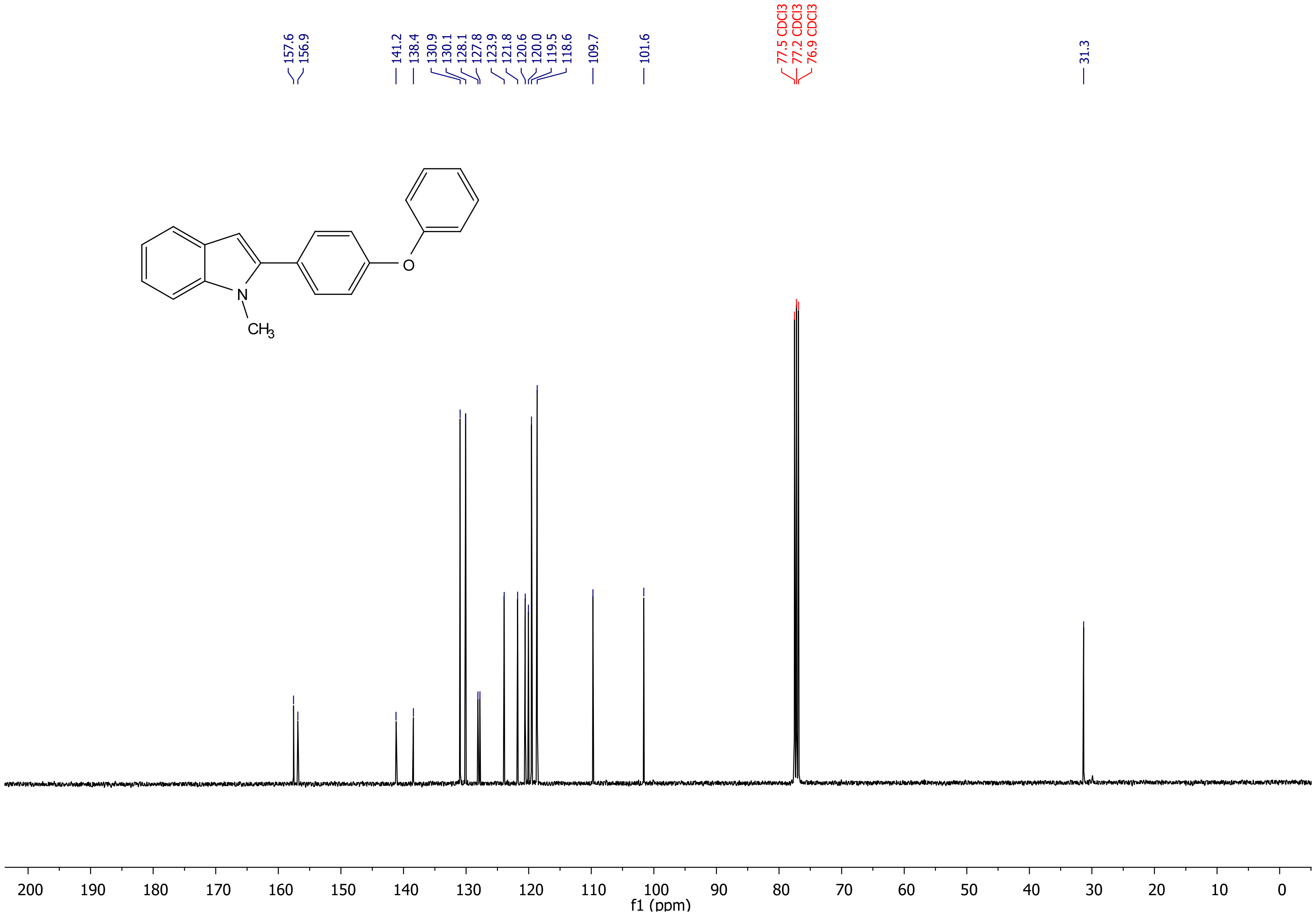

157.6
156.9
141.2
138.4
130.9
130.1
128.1
127.8
123.9
121.8
120.6
120.0
119.5
118.6
109.7
101.6
77.5 CDCl3
77.2 CDCl3
76.9 CDCl3
31.3
O
N
CH3
200
190
180
170
160
150
140
130
120
110
100
90
80
70
60
50
40
30
20
10
0
f1 (ppm)

2-(4'-hydroxyphenyl)-1-methyl-*1H*-indole (3p)

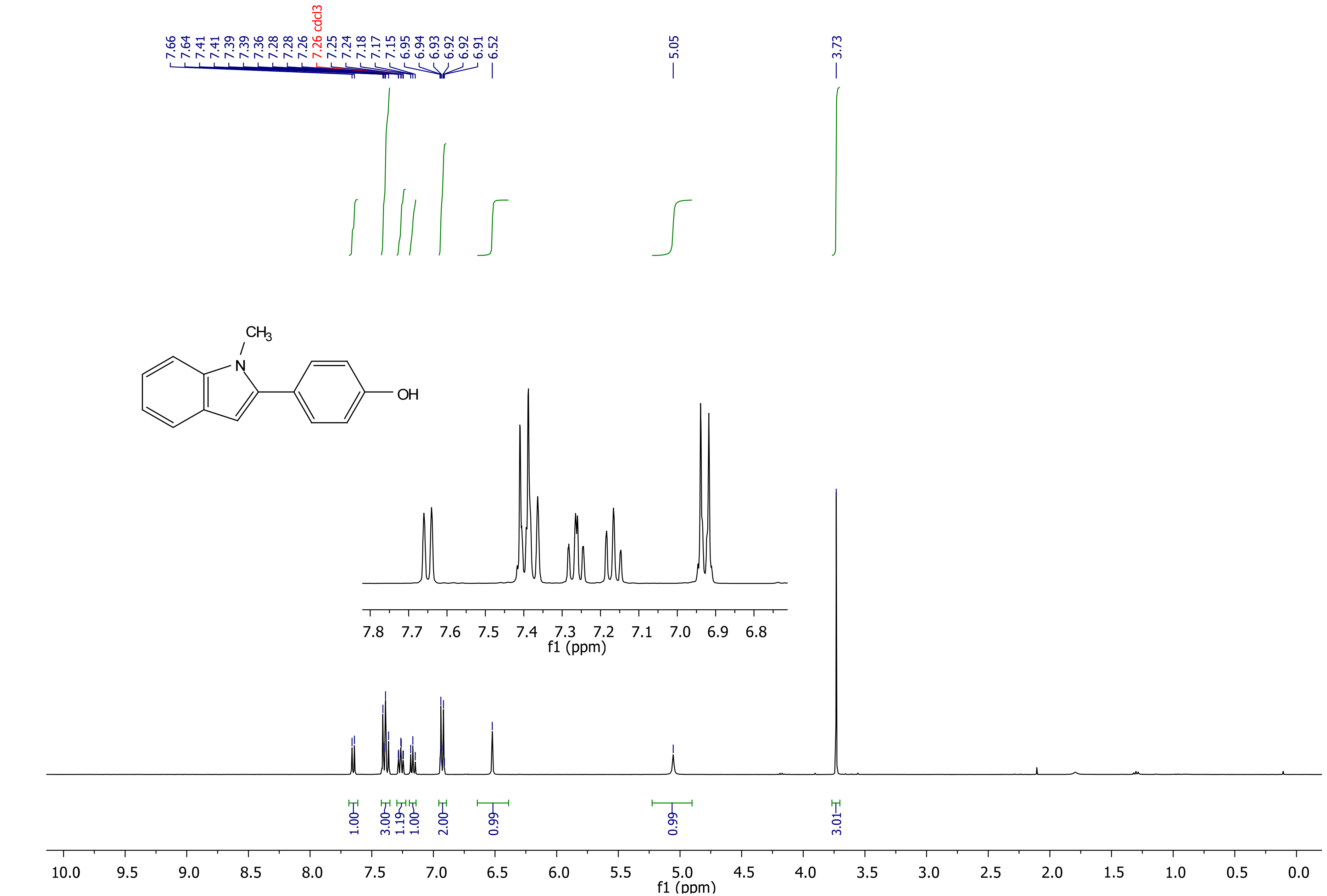

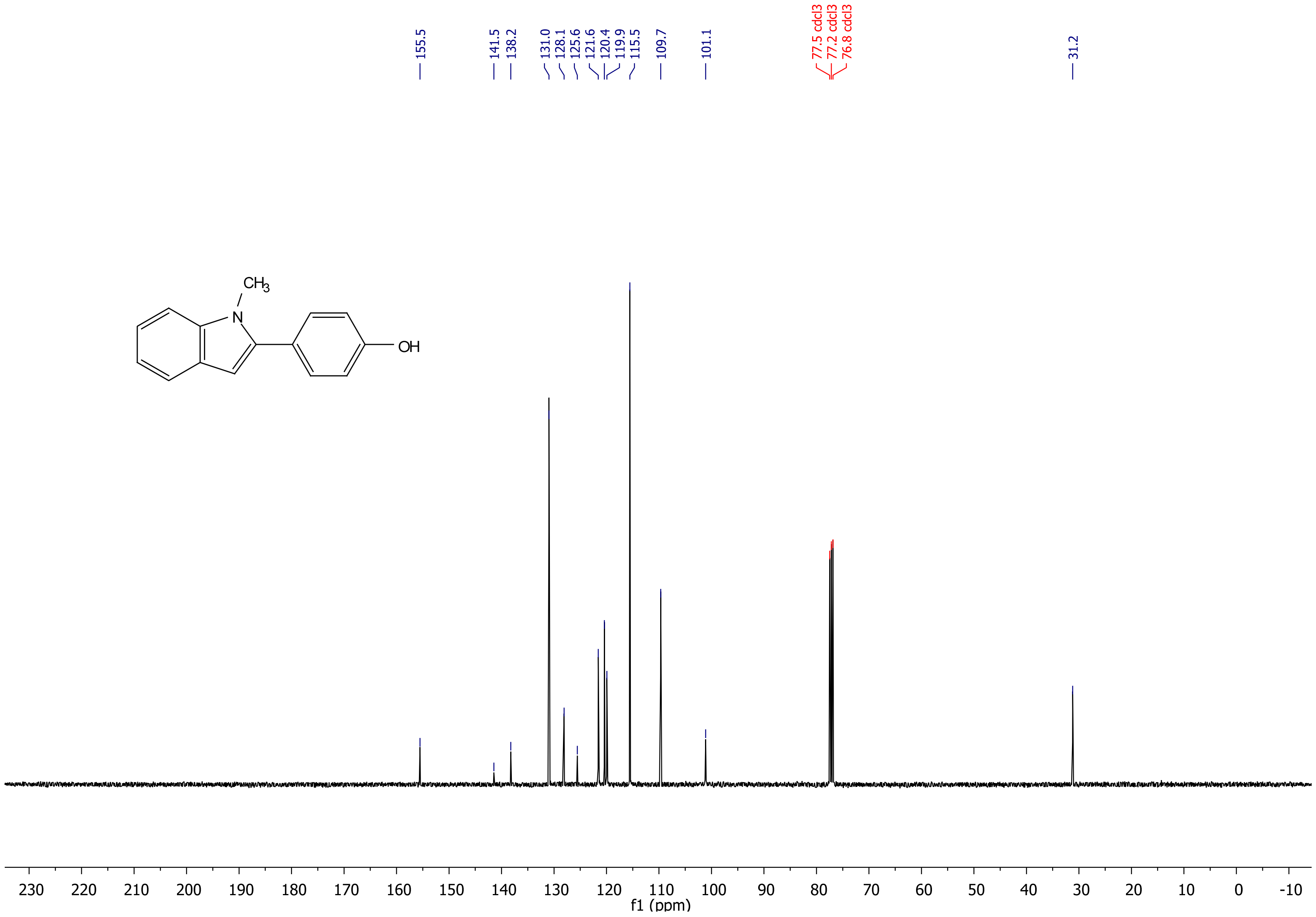
155.5
141.5
138.2
131.0
128.1
125.6
121.6
120.4
119.9
115.5
109.7
101.1
77.5 cdcl3
77.2 cdcl3
76.8 cdcl3
31.2
CH3
N
OH
230
220
210
200
190
180
170
160
150
140
130
120
110
100
90
80
70
60
50
40
30
20
10
0
-10
f1 (ppm)

9-ethyl-3-(1'-methyl-1'H-indol-2'-yl)-*9H*-carbazole (3q)

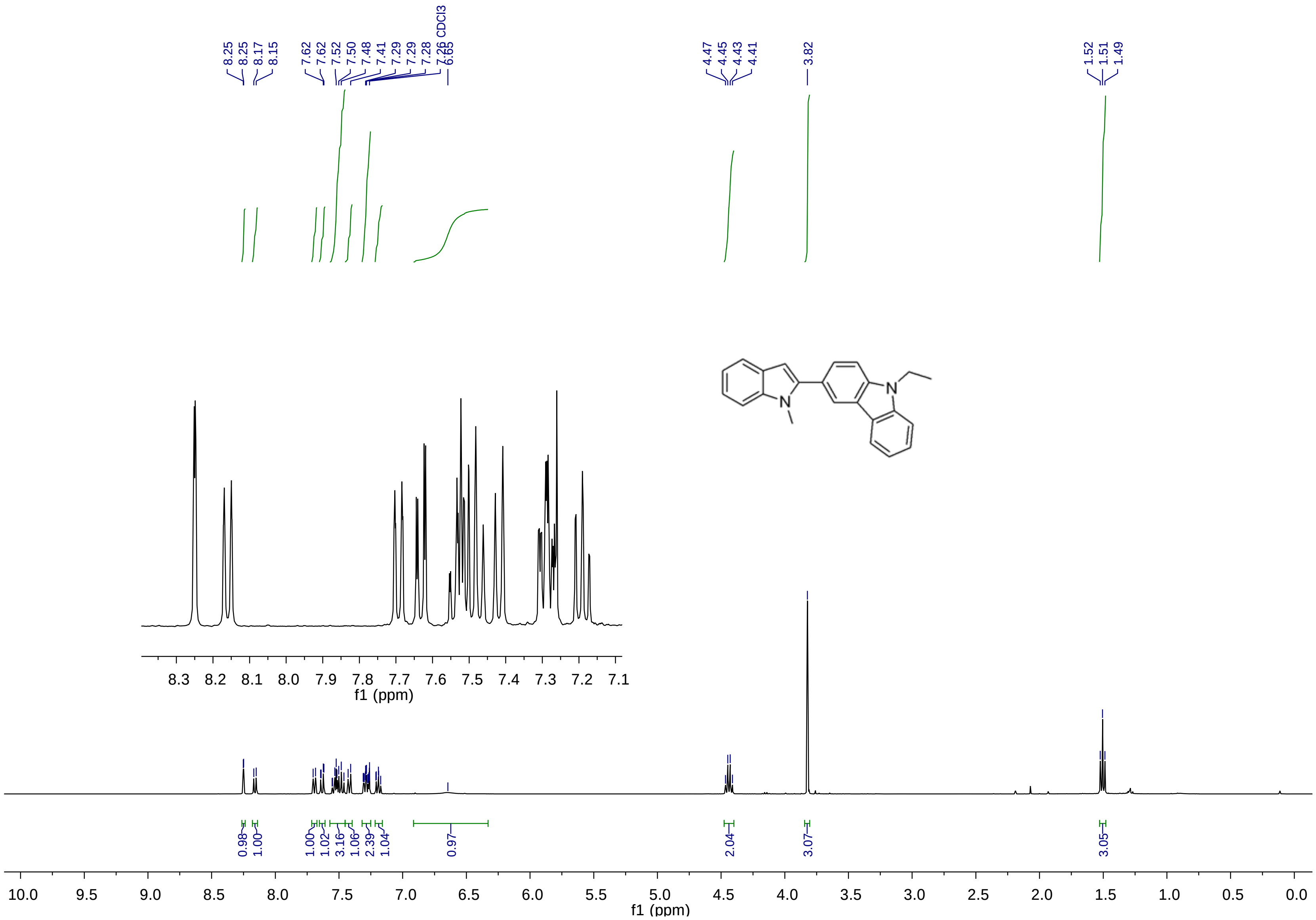

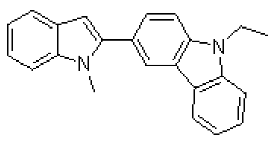

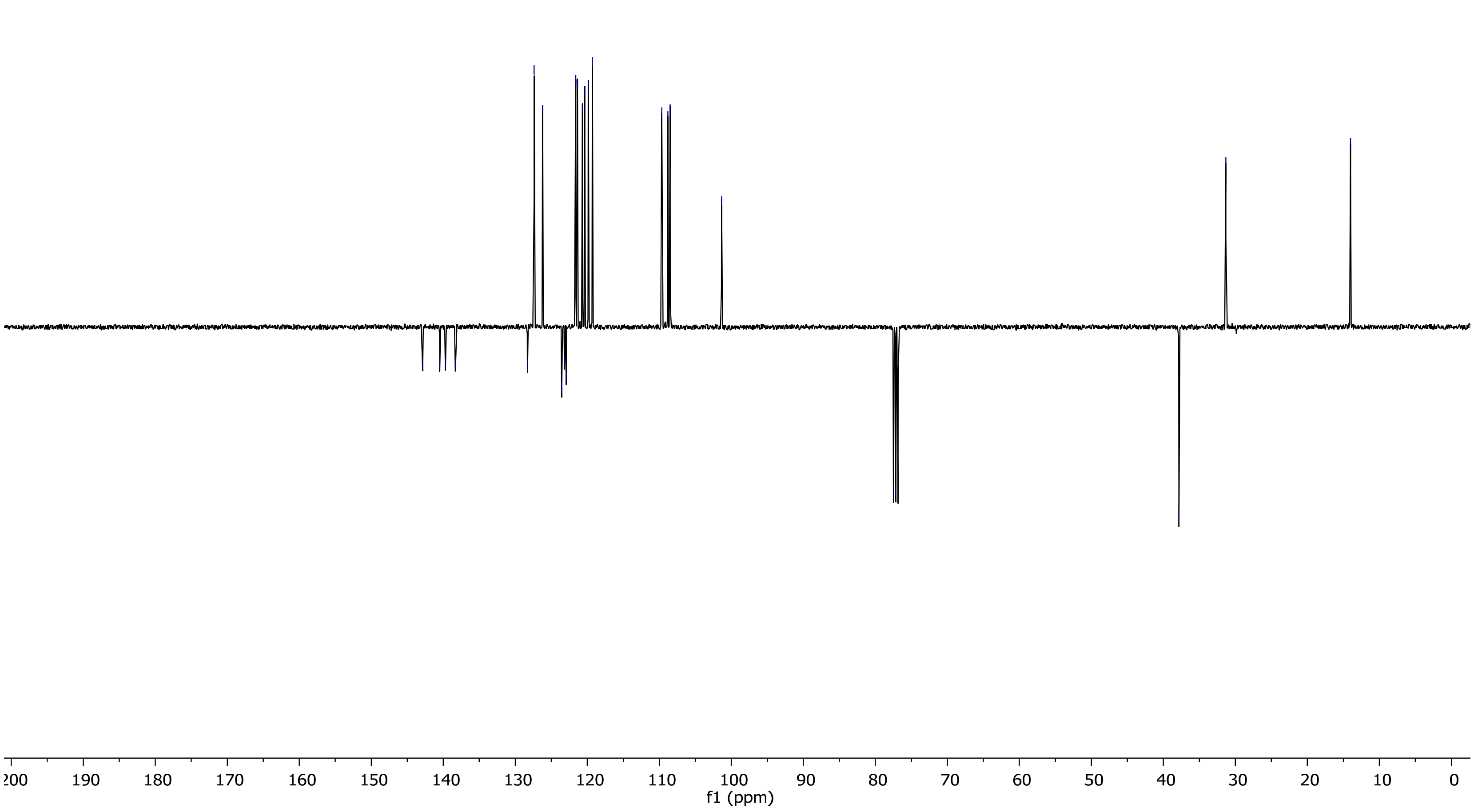
142.86
140.50
139.71
138.32
128.32
127.39
126.19
123.56
123.16
122.94
121.61
121.37
120.67
120.35
119.86
119.29
109.66
108.82
108.50
101.36
77.16 CDCl3
37.83
31.32
14.01
200 190 180 170 160 150 140 130 120 110 100 90 80 70 60 50 40 30 20 10 0
f1 (ppm)

Ethyl 5-(1-methyl-1*H*-indol-2-yl)benzofuran-2-carboxylate (3r)

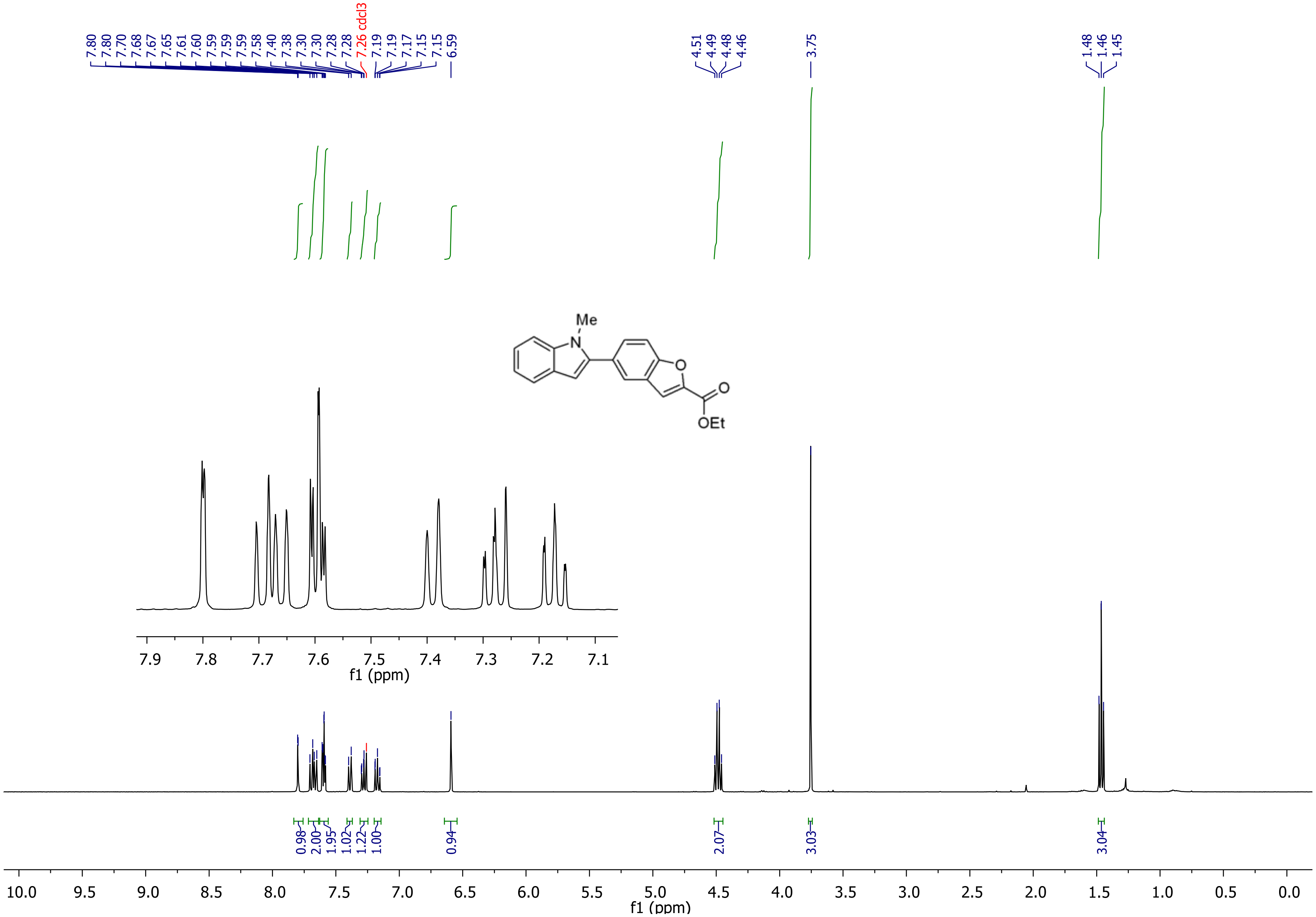

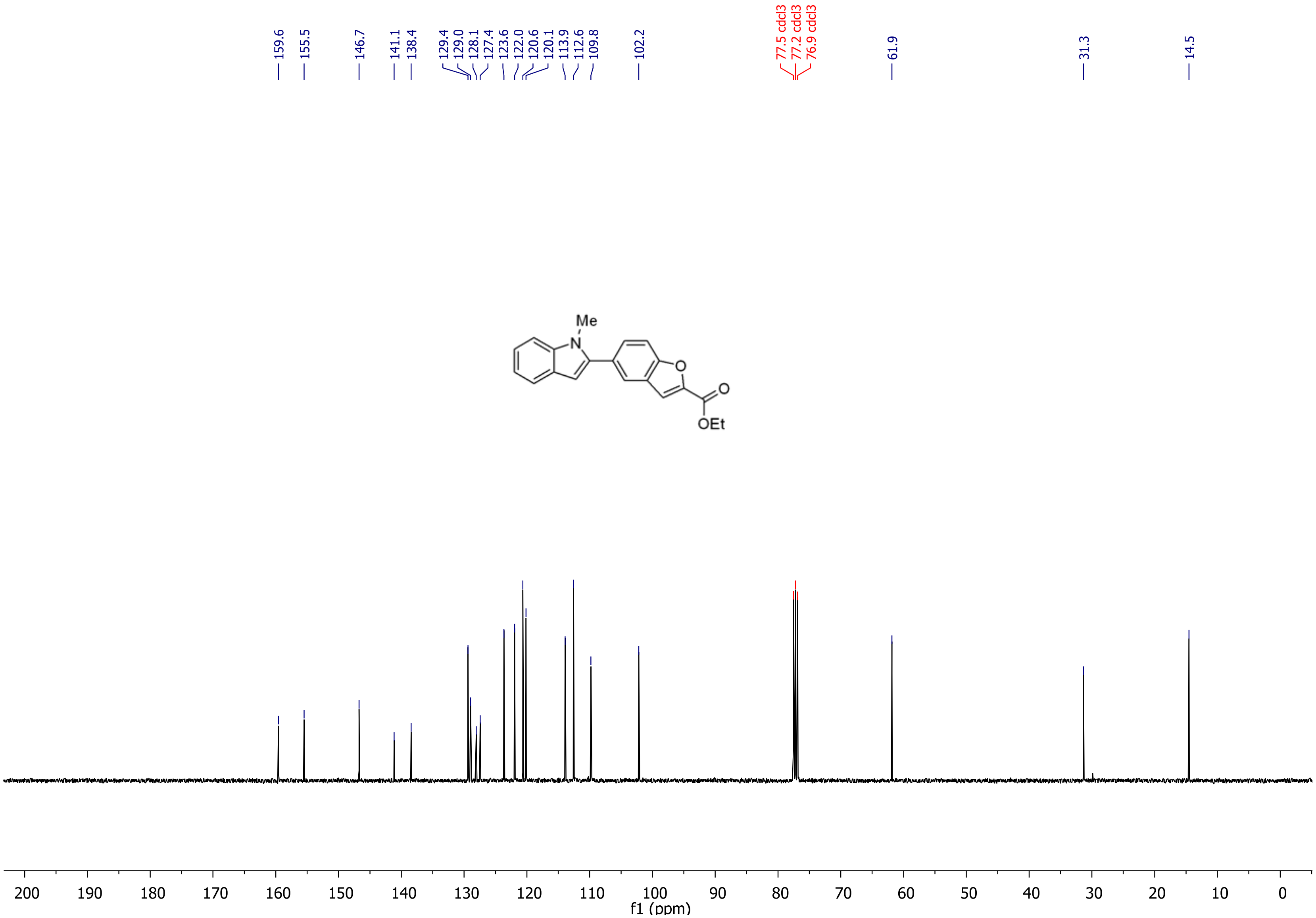
159.6
155.5
146.7
141.1
138.4
129.4
129.0
128.1
127.4
123.6
122.0
120.6
120.1
113.9
112.6
109.8
102.2
77.5 cdcl3
77.2 cdcl3
76.9 cdcl3
61.9
31.3
14.5
Me
N
O
O
OEt
200 190 180 170 160 150 140 130 120 110 100 90 80 70 60 50 40 30 20 10 0
f1 (ppm)

2-(4-fluorophenyl)-1-methyl-1*H*-indole (3s)

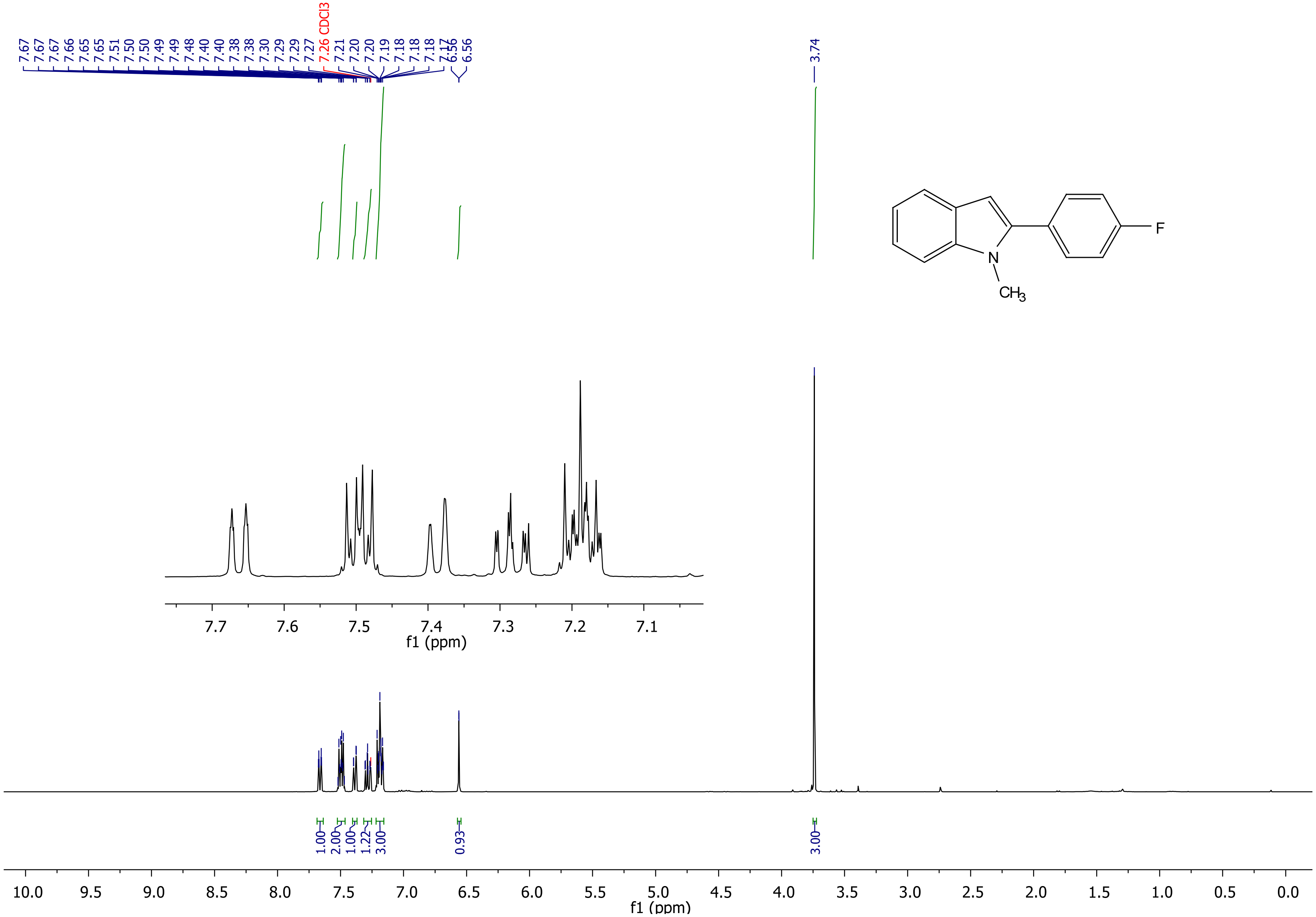

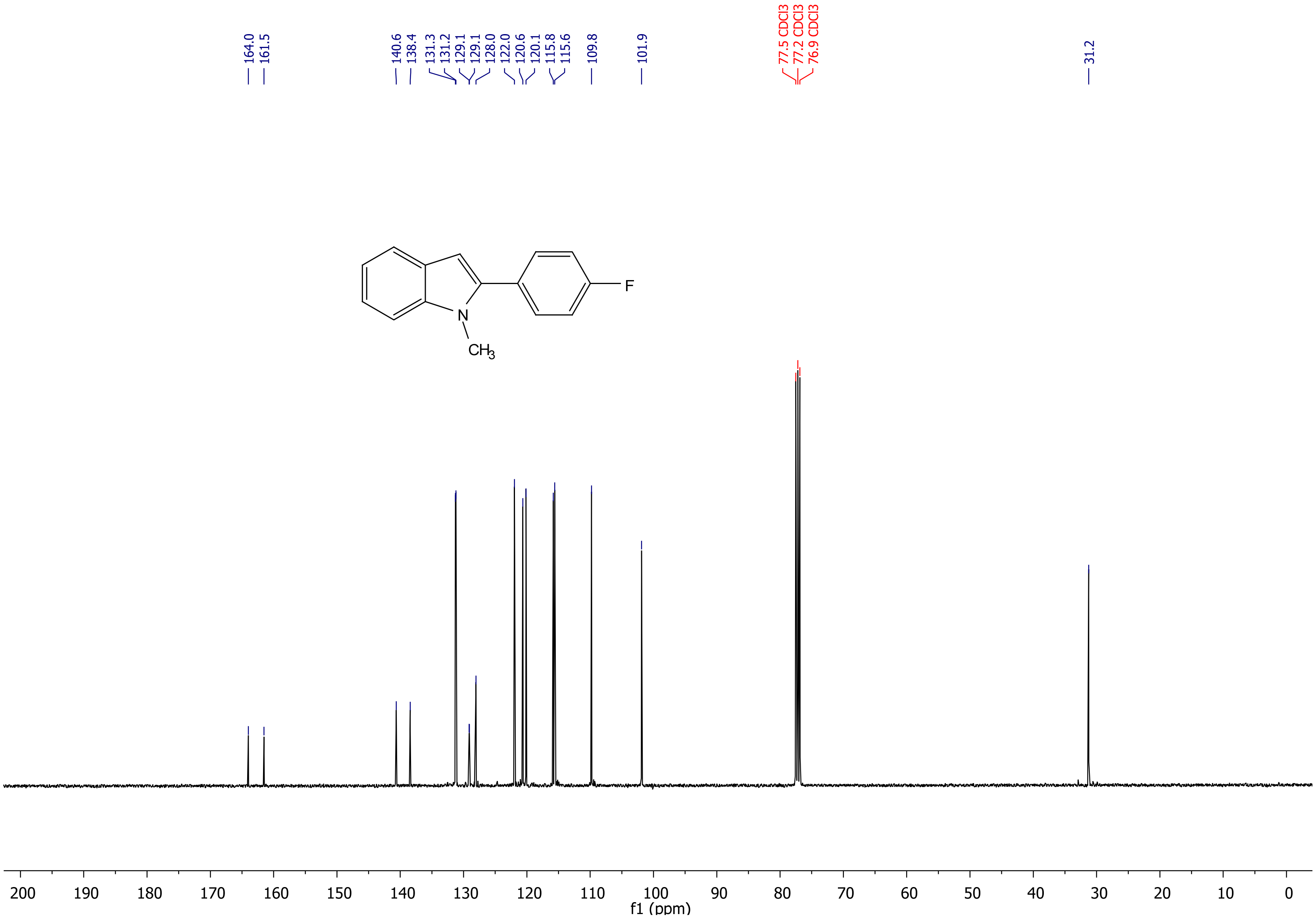

164.0
161.5
140.6
138.4
131.3
131.2
129.1
129.1
128.0
122.0
120.6
120.1
115.8
115.6
109.8
101.9
77.5 CDCl3
77.2 CDCl3
76.9 CDCl3
31.2
N
CH3
F
200 190 180 170 160 150 140 130 120 110 100 90 80 70 60 50 40 30 20 10 0
f1 (ppm)

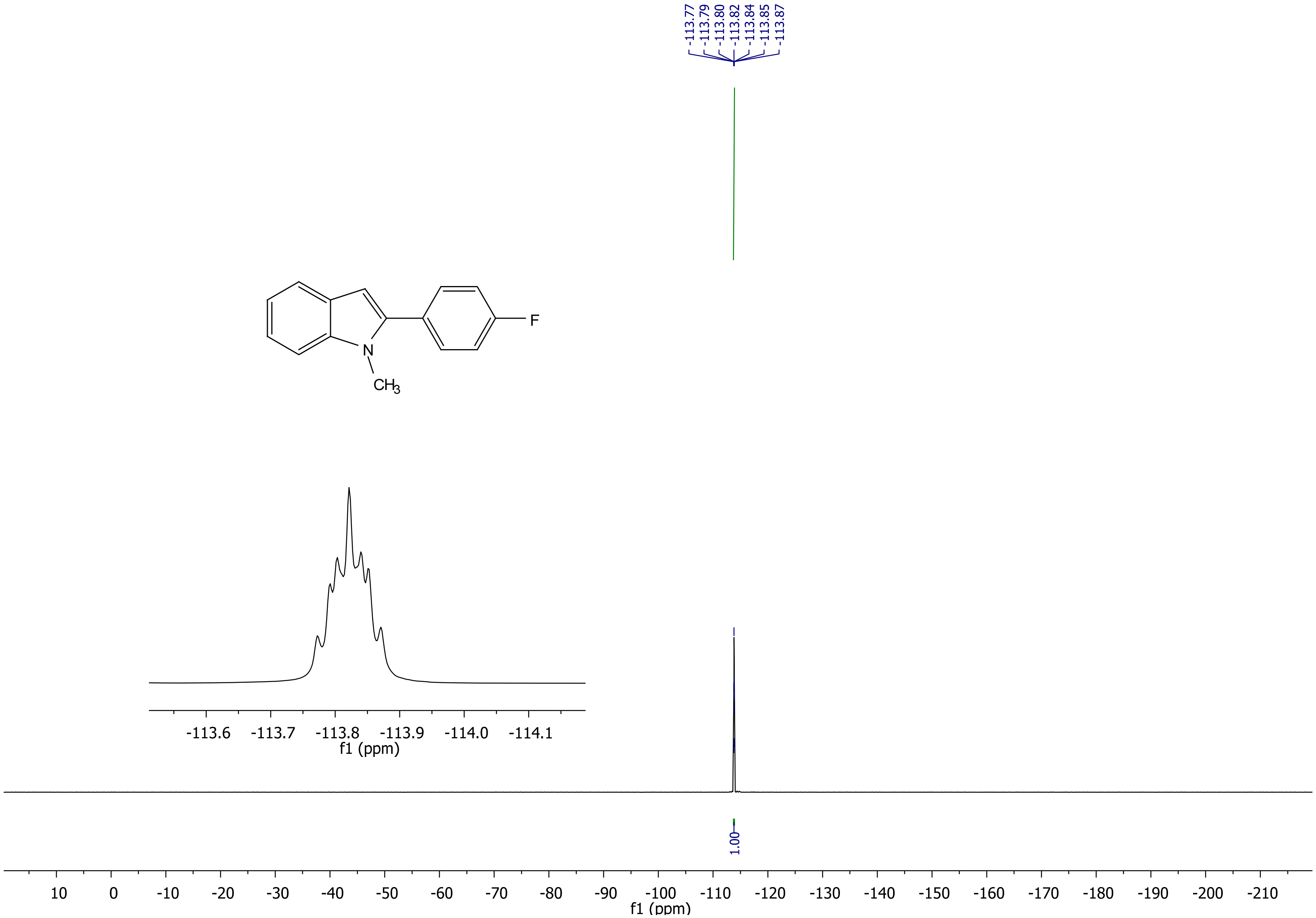
-113.77
-113.79
-113.80
-113.82
-113.84
-113.85
-113.87
F
N
CH3
-113.6
-113.7
-113.8
-113.9
-114.0
-114.1
f1 (ppm)
1.00
10
0
-10
-20
-30
-40
-50
-60
-70
-80
-90
-100
-110
-120
-130
-140
-150
-160
-170
-180
-190
-200
-210
f1 (ppm)

1,3-dimethyl-2-(3-(trifluoromethyl)phenyl)-1H-indole (3t)

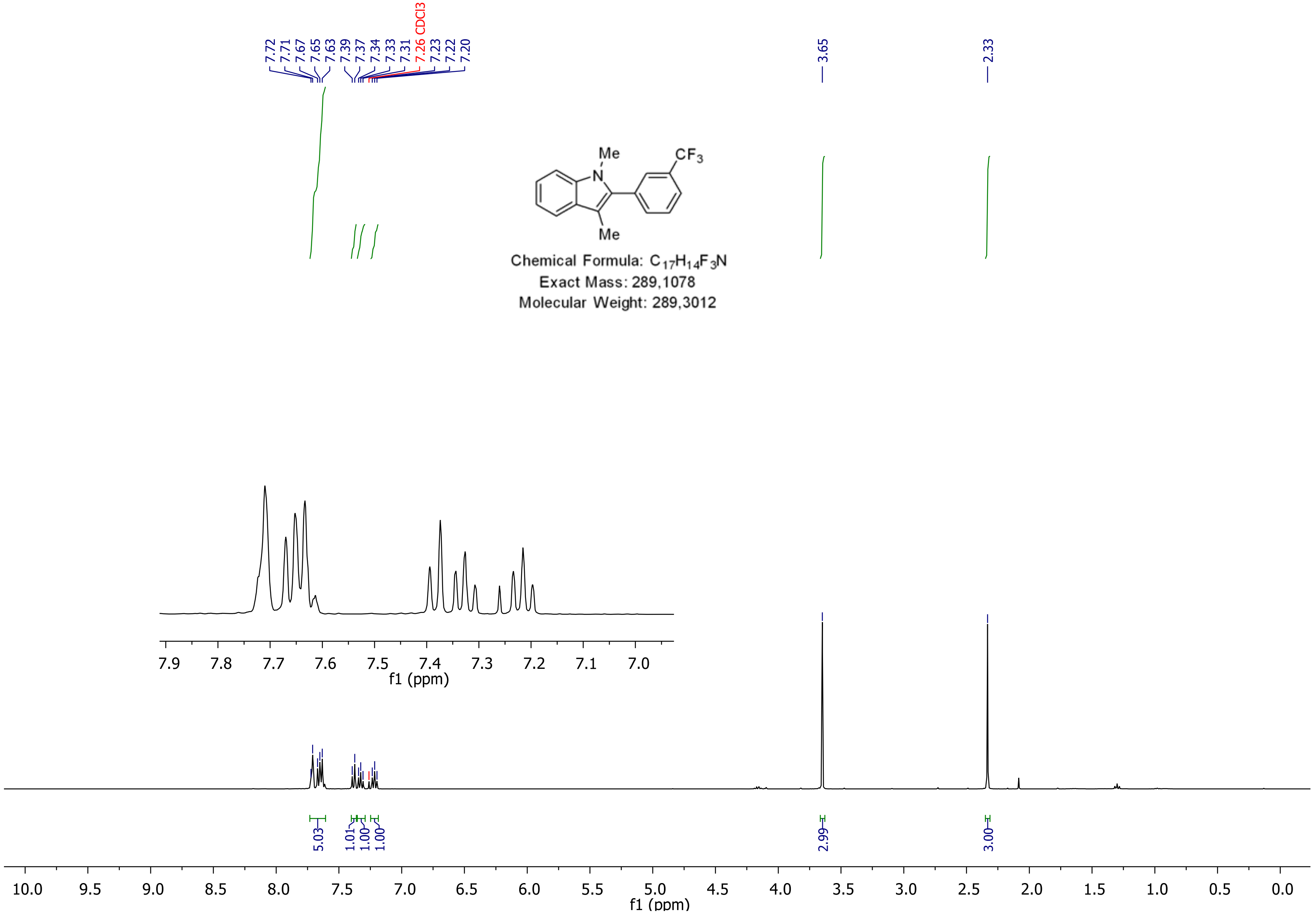

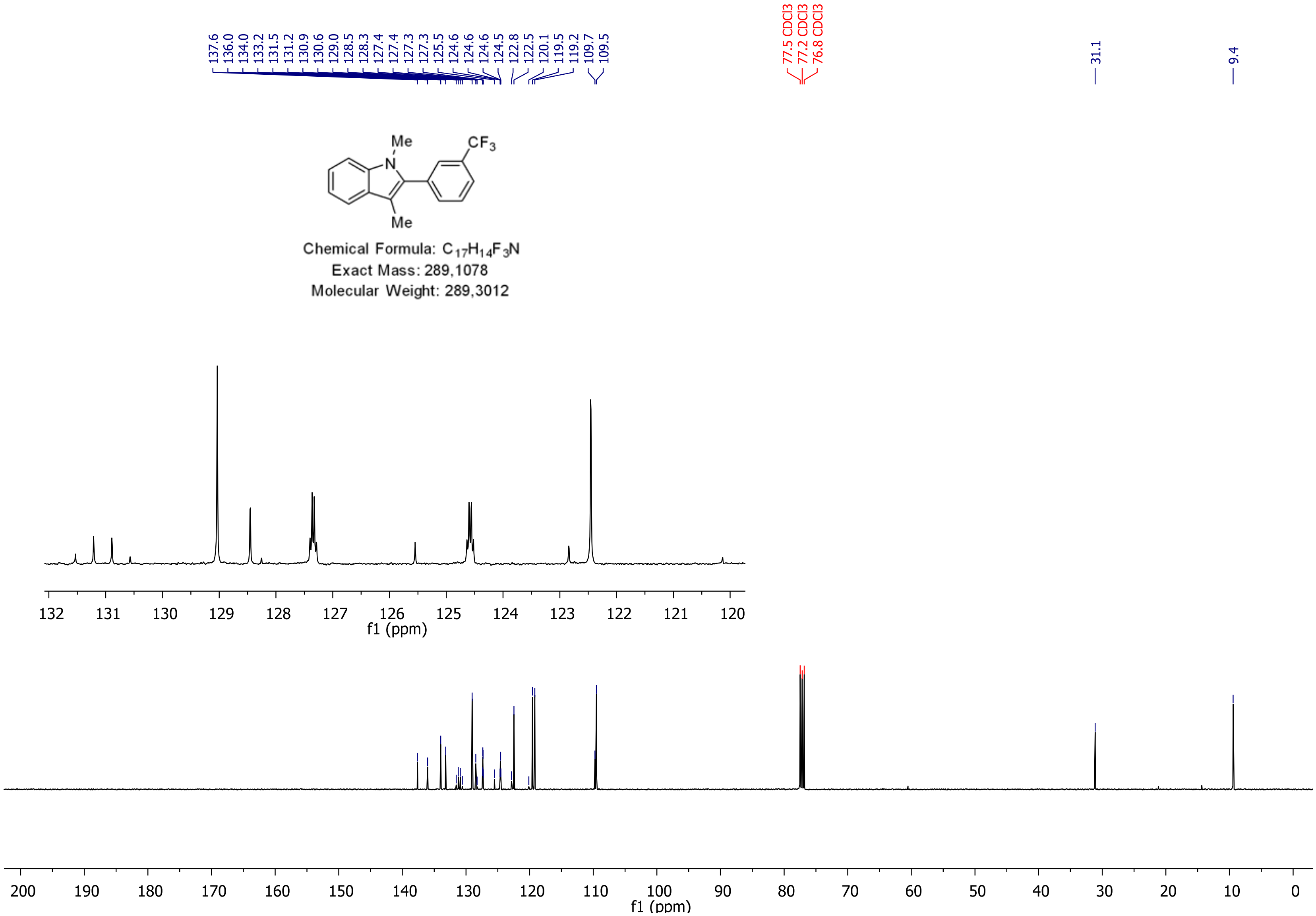
137.6
136.0
134.0
133.2
131.5
131.2
130.9
130.6
129.0
128.5
128.3
127.4
127.4
127.3
127.3
125.5
124.6
124.6
124.6
124.5
122.8
122.5
120.1
119.5
119.2
109.7
109.5
77.5 CDCl3
77.2 CDCl3
76.8 CDCl3
31.1
9.4
Me
N
CF3
Me
Chemical Formula: C17H14F3N
Exact Mass: 289,1078
Molecular Weight: 289,3012
f1 (ppm)
f1 (ppm)

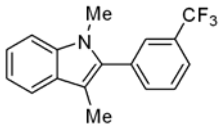

Chemical Formula: $C_{17}H_{14}F_3N$
Exact Mass: 289,1078
Molecular Weight: 289,3012

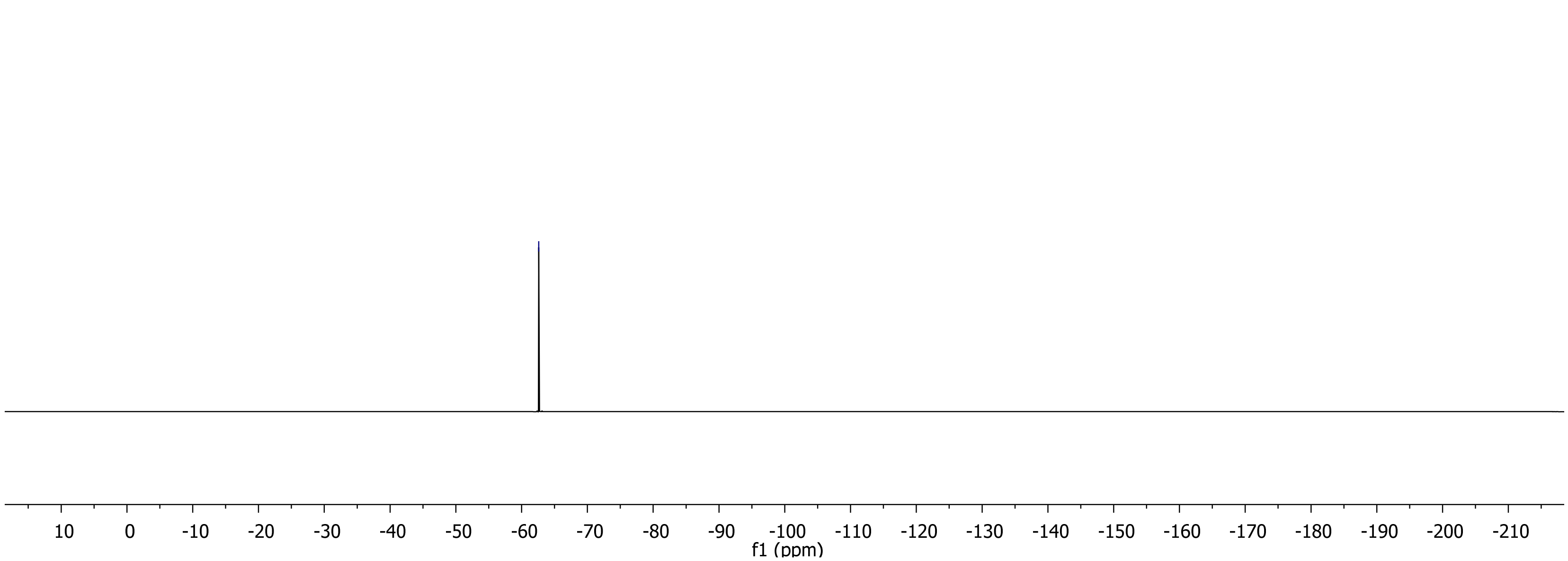

1,3-dimethyl-2-(4-nitrophenyl)-1H-indole (3u)

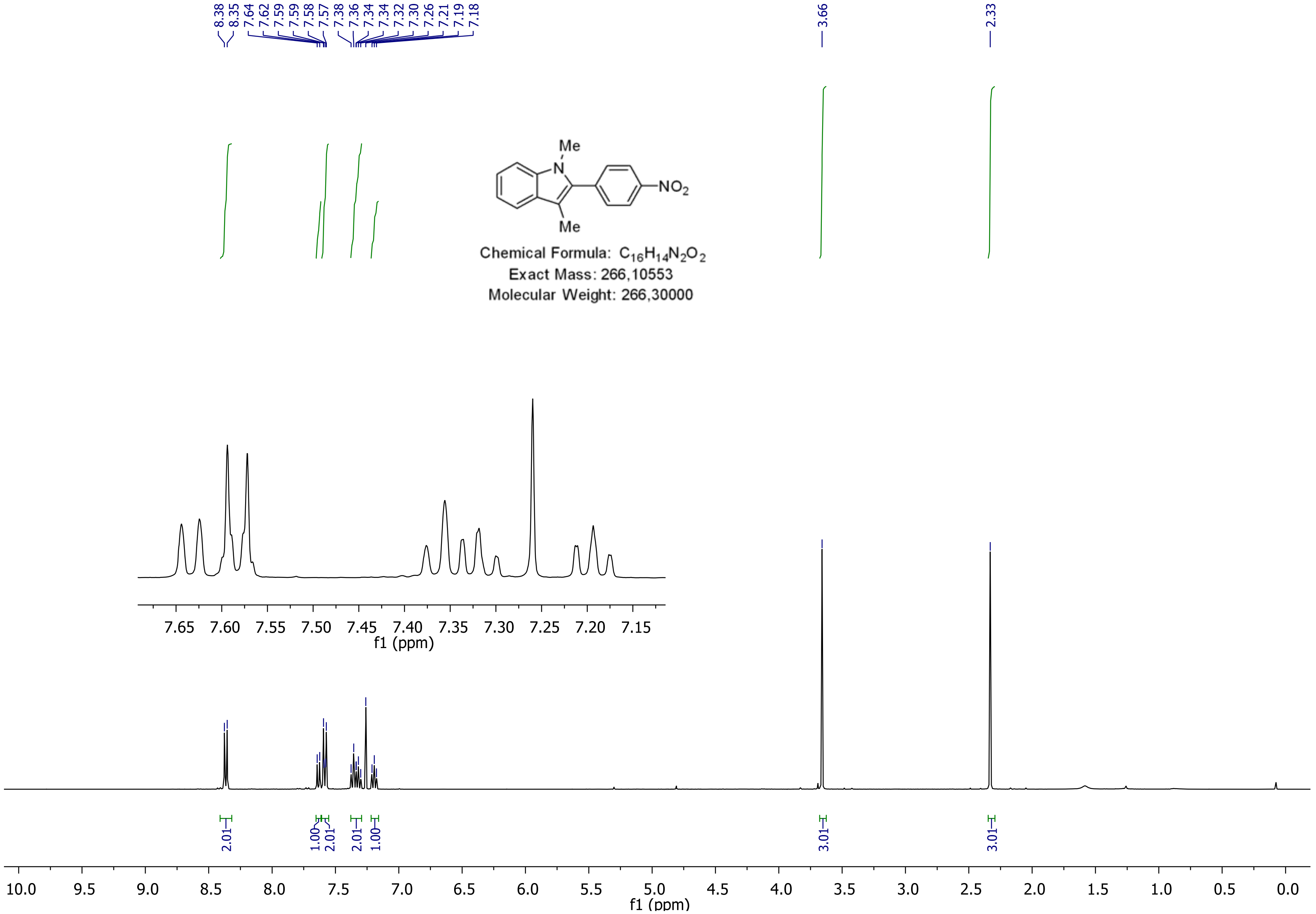

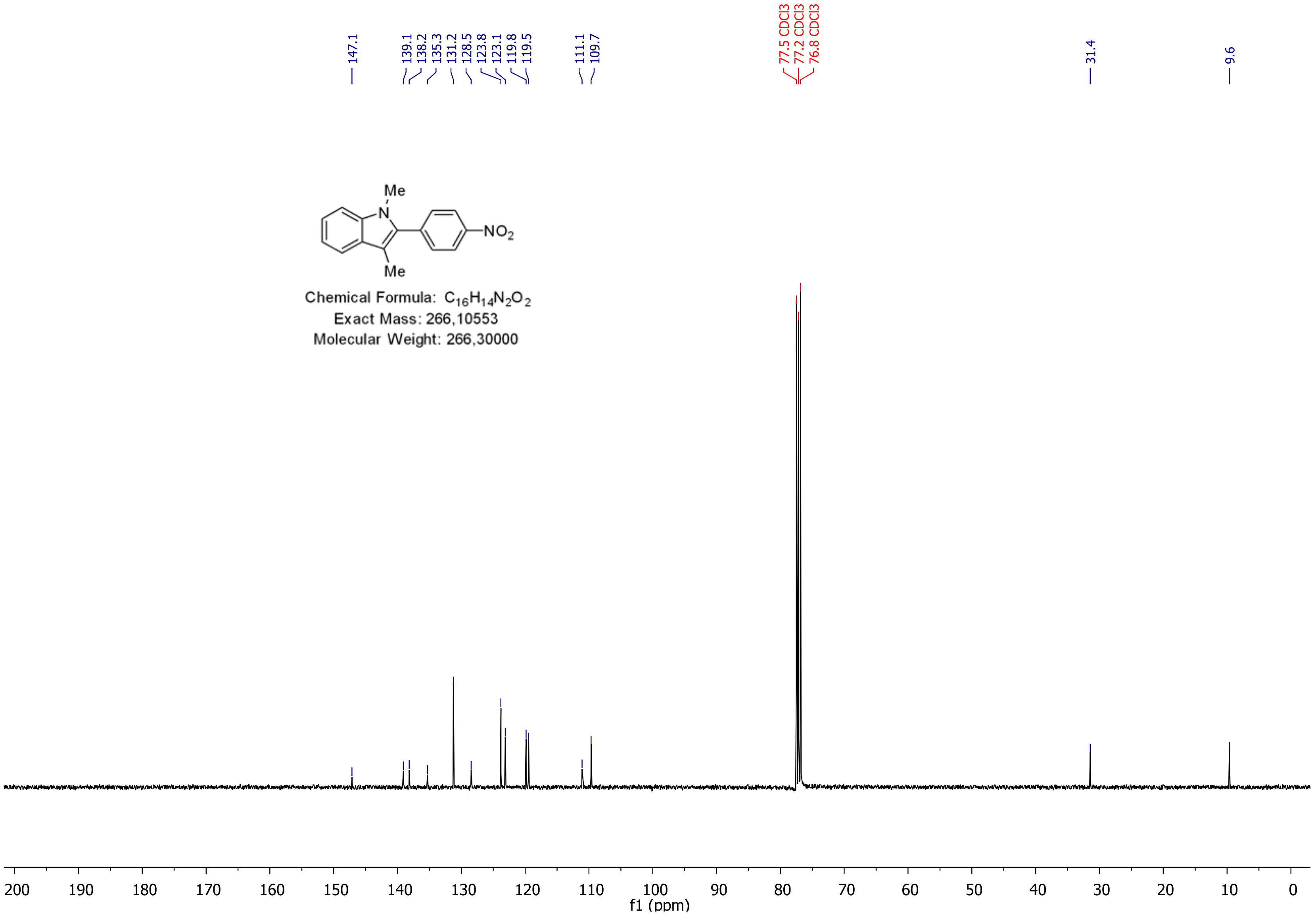
147.1
139.1
138.2
135.3
131.2
128.5
123.8
123.1
119.8
119.5
111.1
109.7
77.5 CDCl3
77.2 CDCl3
76.8 CDCl3
31.4
9.6
Me
N
NO2
Me
Chemical Formula: C16H14N2O2
Exact Mass: 266,10553
Molecular Weight: 266,30000
200
190
180
170
160
150
140
130
120
110
100
90
80
70
60
50
40
30
20
10
0
f1 (ppm)

2-(2-chlorophenyl)-1-methyl-1H-indole (3v)

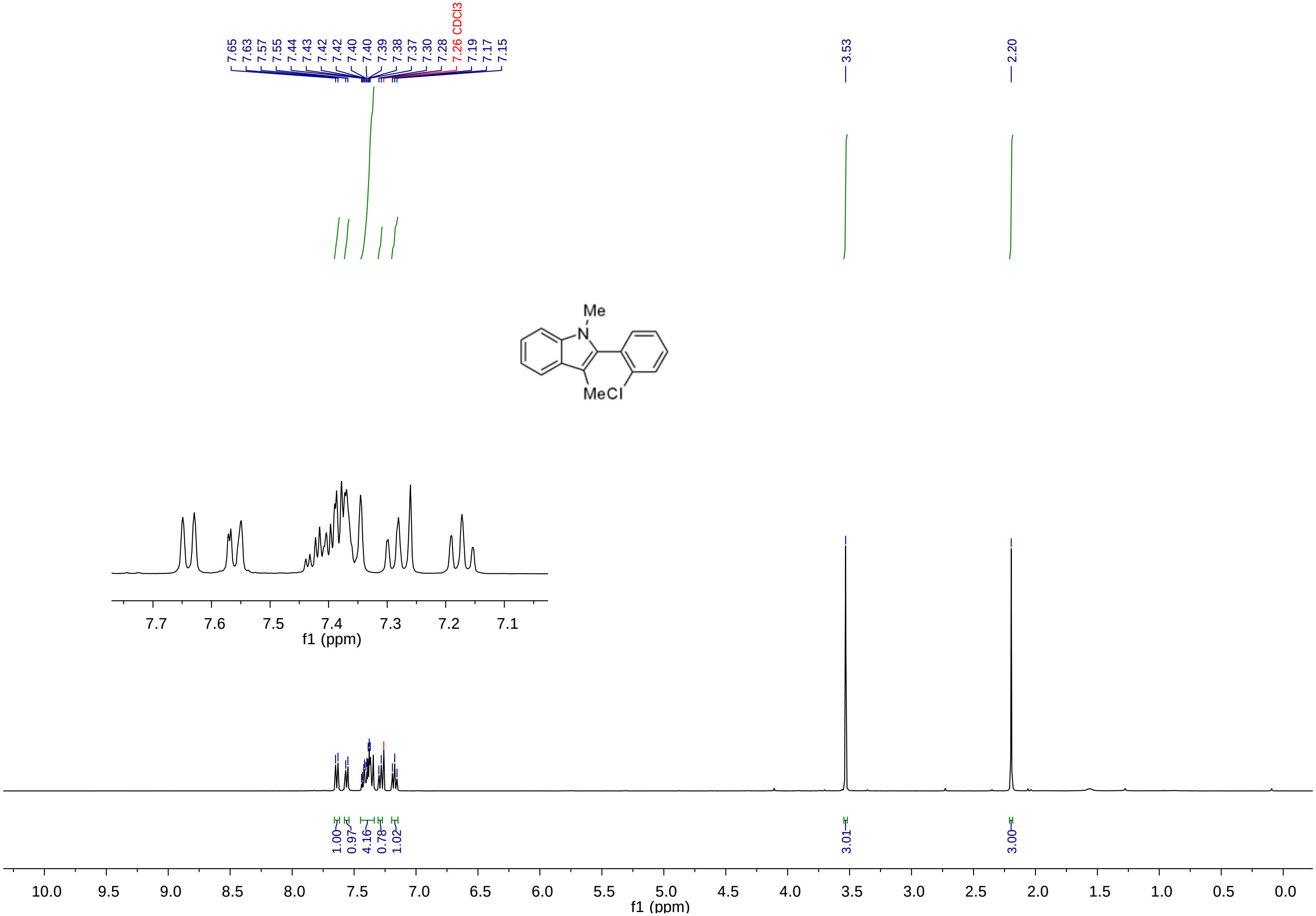

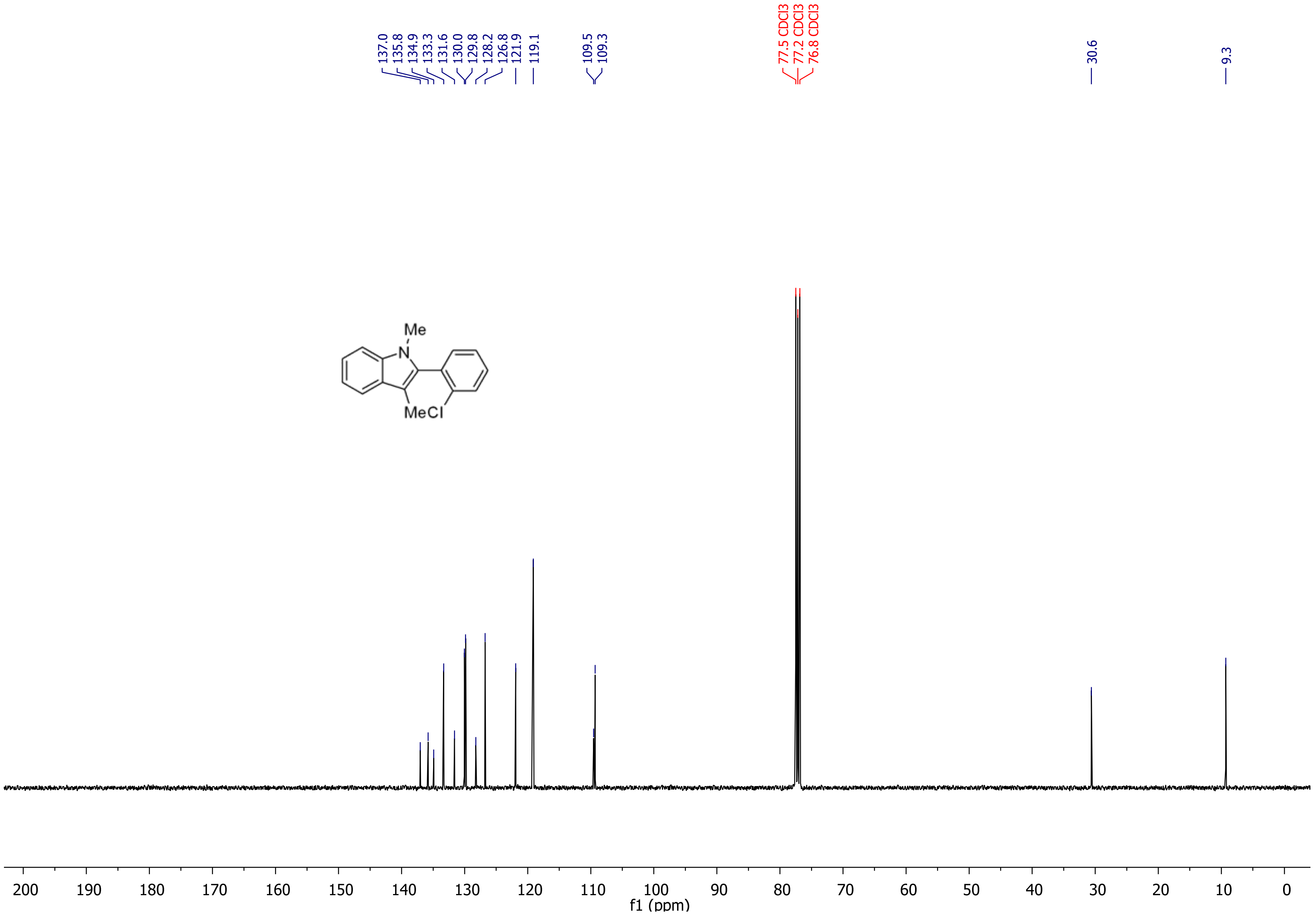
137.0
135.8
134.9
133.3
131.6
130.0
129.8
128.2
126.8
121.9
119.1
109.5
109.3
77.5 CDCl3
77.2 CDCl3
76.8 CDCl3
30.6
9.3
Me
N
Me
Cl
200
190
180
170
160
150
140
130
120
110
100
90
80
70
60
50
40
30
20
10
0
f1 (ppm)

2-(3-iodophenyl)-1-methyl-1H-indole (3w)

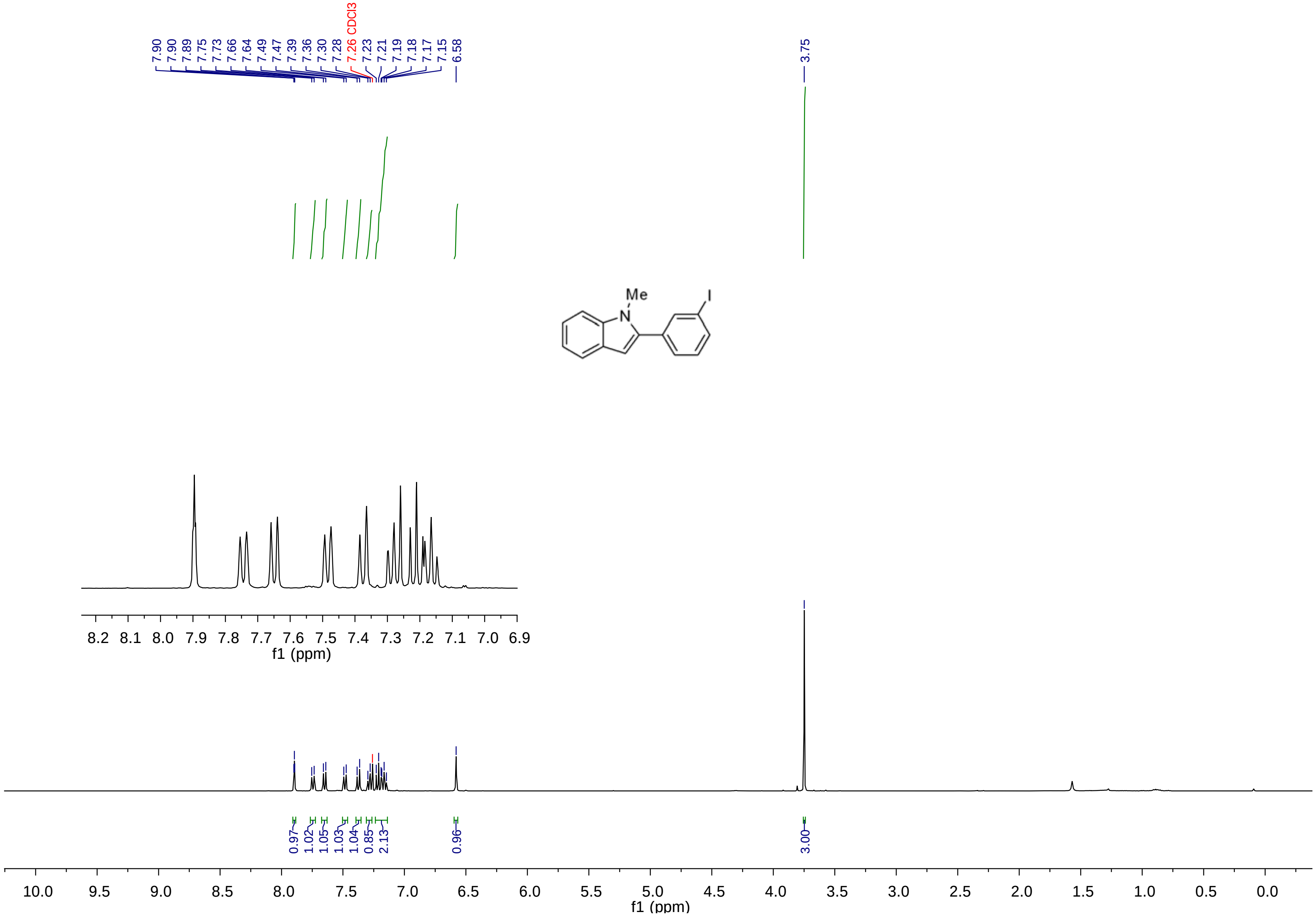

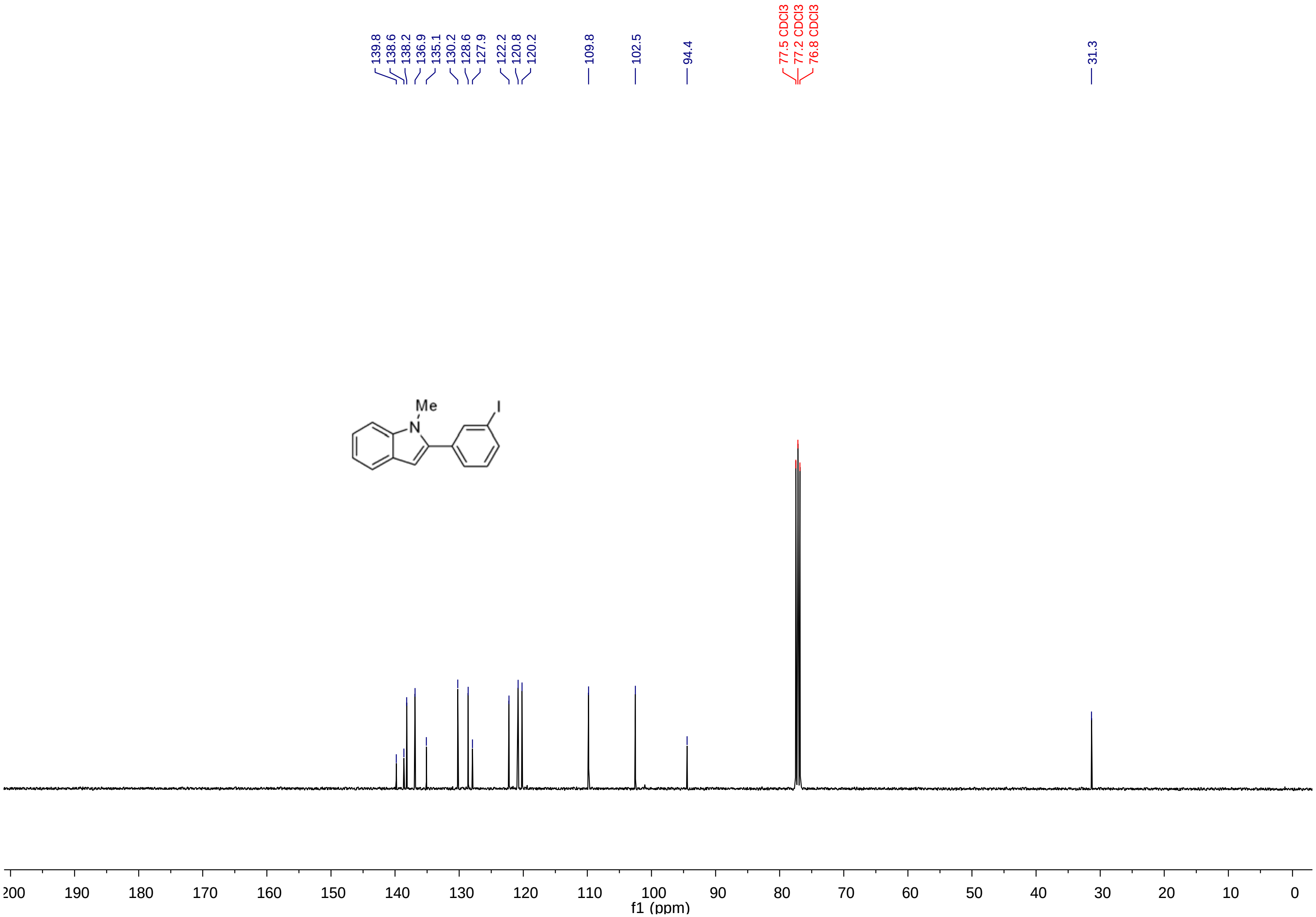

139.8
138.6
138.2
136.9
135.1
130.2
128.6
127.9
122.2
120.8
120.2
109.8
102.5
94.4
77.5 CDCl3
77.2 CDCl3
76.8 CDCl3
31.3
Me
N
I
200 190 180 170 160 150 140 130 120 110 100 90 80 70 60 50 40 30 20 10 0
f1 (ppm)

2-(4-bromophenyl)-1,3-dimethyl-1H-indole (3x)

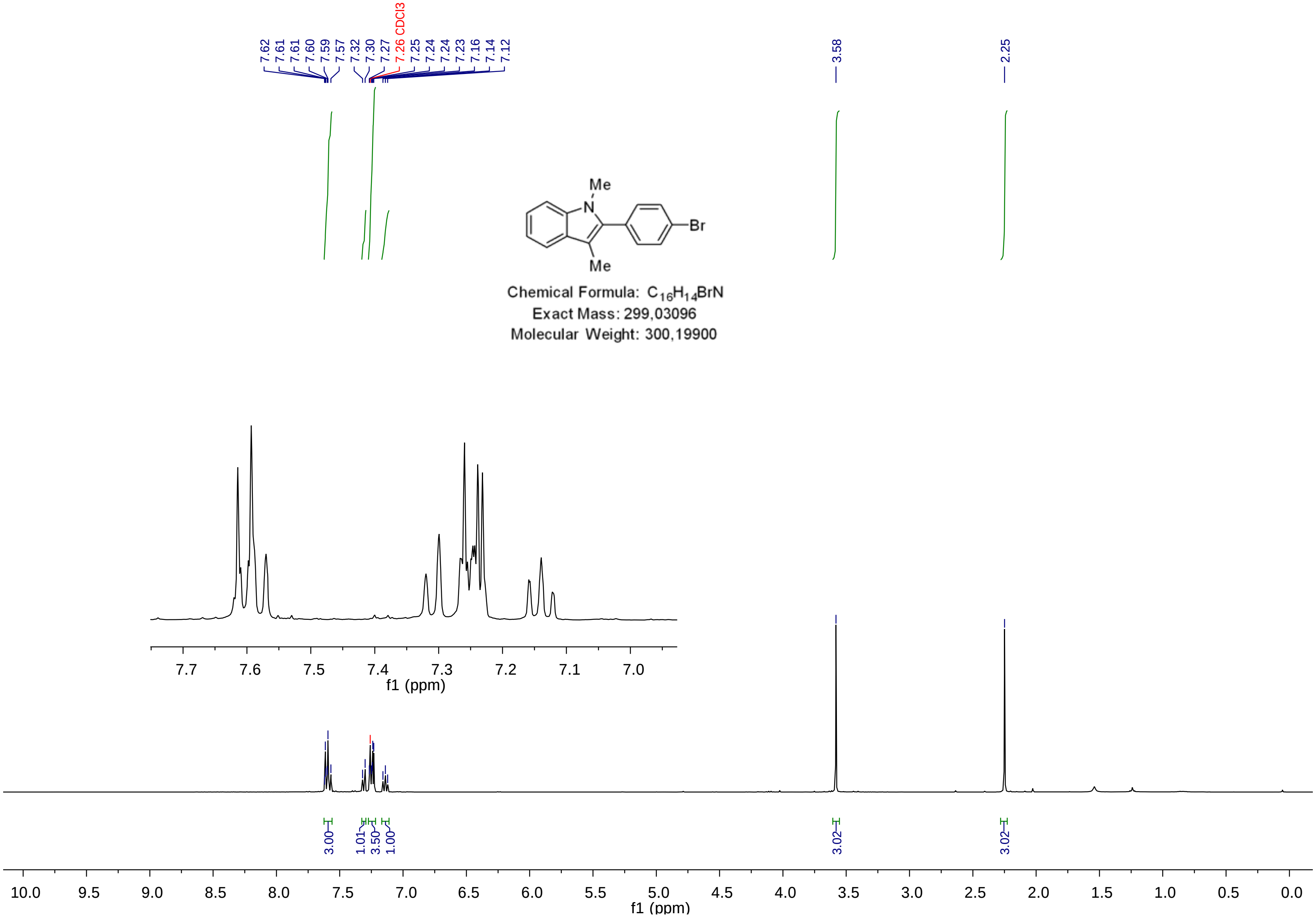

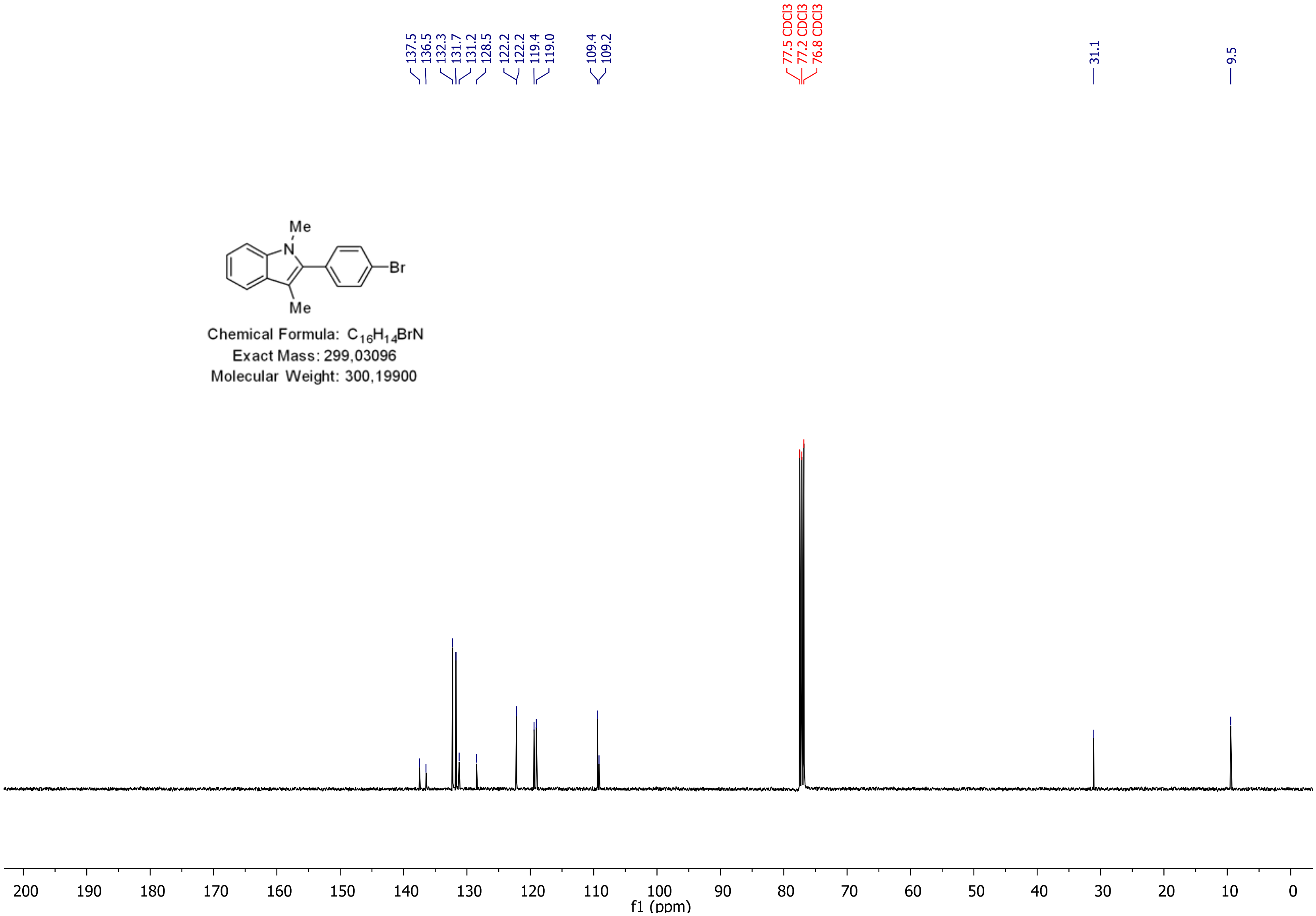
137.5
136.5
132.3
131.7
131.2
128.5
122.2
122.2
119.4
119.0
109.4
109.2
77.5 CDCl3
77.2 CDCl3
76.8 CDCl3
31.1
9.5
Me
N
Br
Me
Chemical Formula: C16H14BrN
Exact Mass: 299,03096
Molecular Weight: 300,19900
200 190 180 170 160 150 140 130 120 110 100 90 80 70 60 50 40 30 20 10 0
f1 (ppm)

1-(4-(1-methyl-1H-indol-2-yl)phenyl)ethan-1-one (3ya)

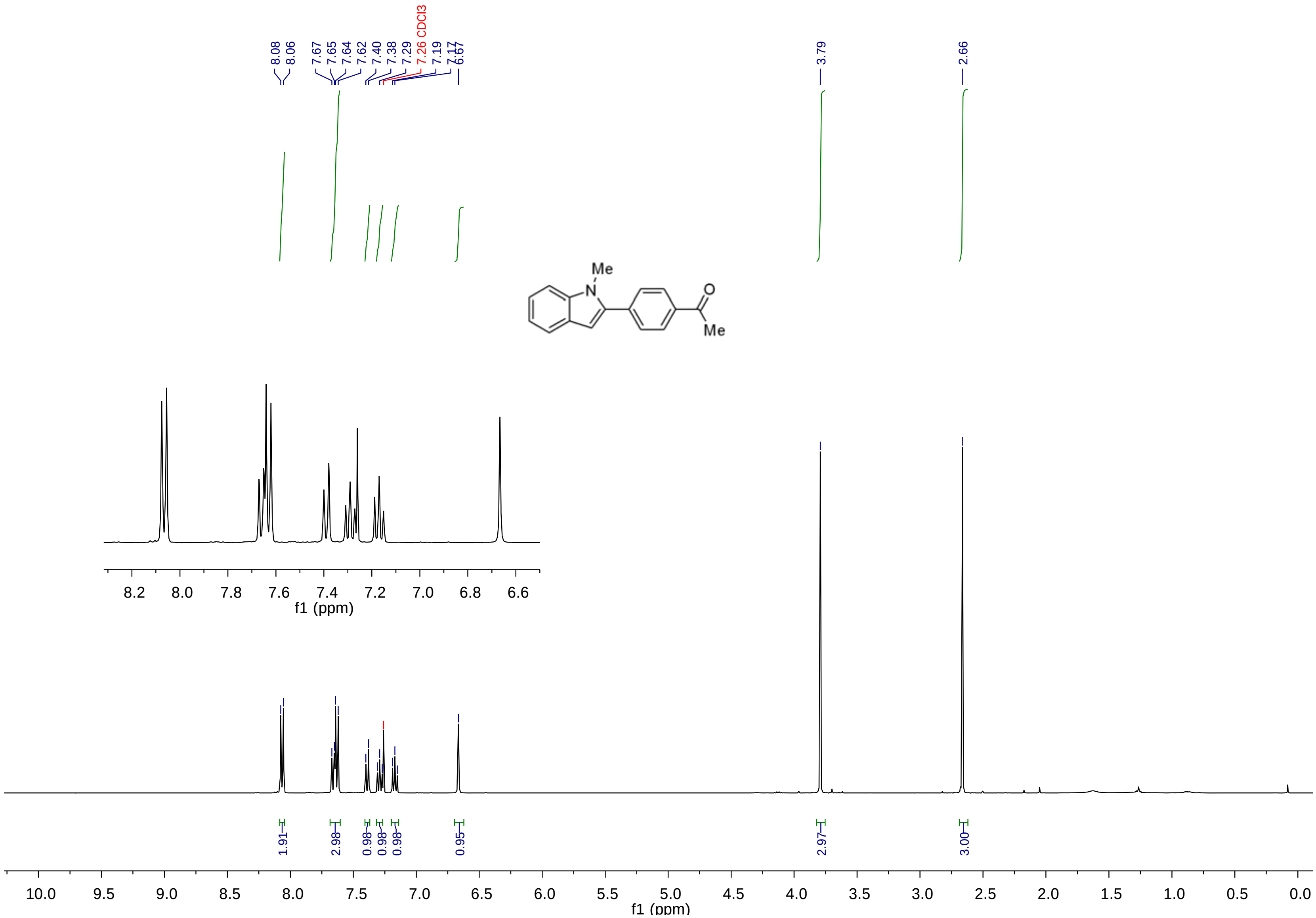

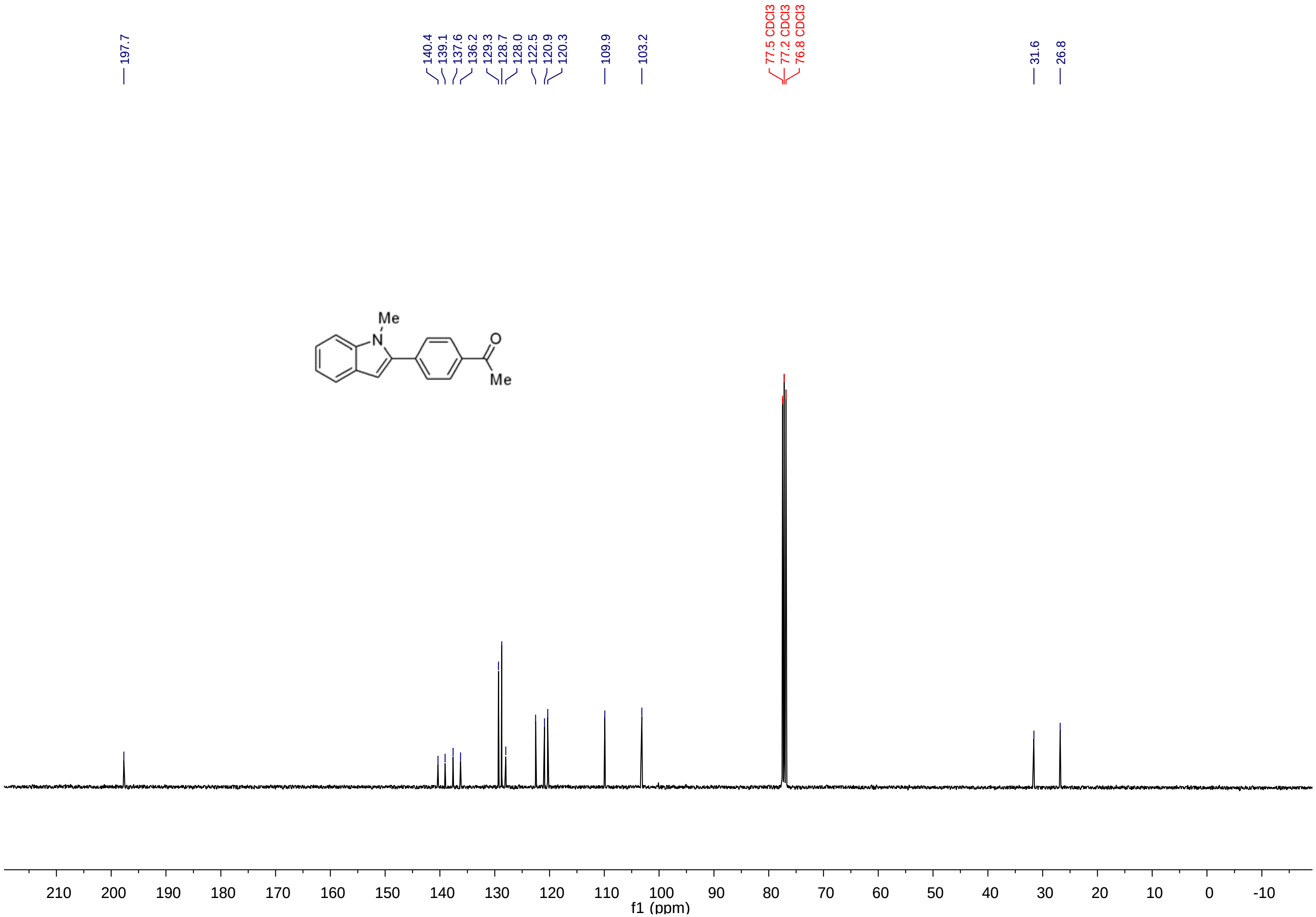

197.7
140.4
139.1
137.6
136.2
129.3
128.7
128.0
122.5
120.9
120.3
109.9
103.2
77.5 CDCl3
77.2 CDCl3
76.8 CDCl3
31.6
26.8
Me
N
O
Me
210 200 190 180 170 160 150 140 130 120 110 100 90 80 70 60 50 40 30 20 10 0 -10
f1 (ppm)

1-(4-(1,3-dimethyl-1H-indol-2-yl)phenyl)ethan-1-one (3y)

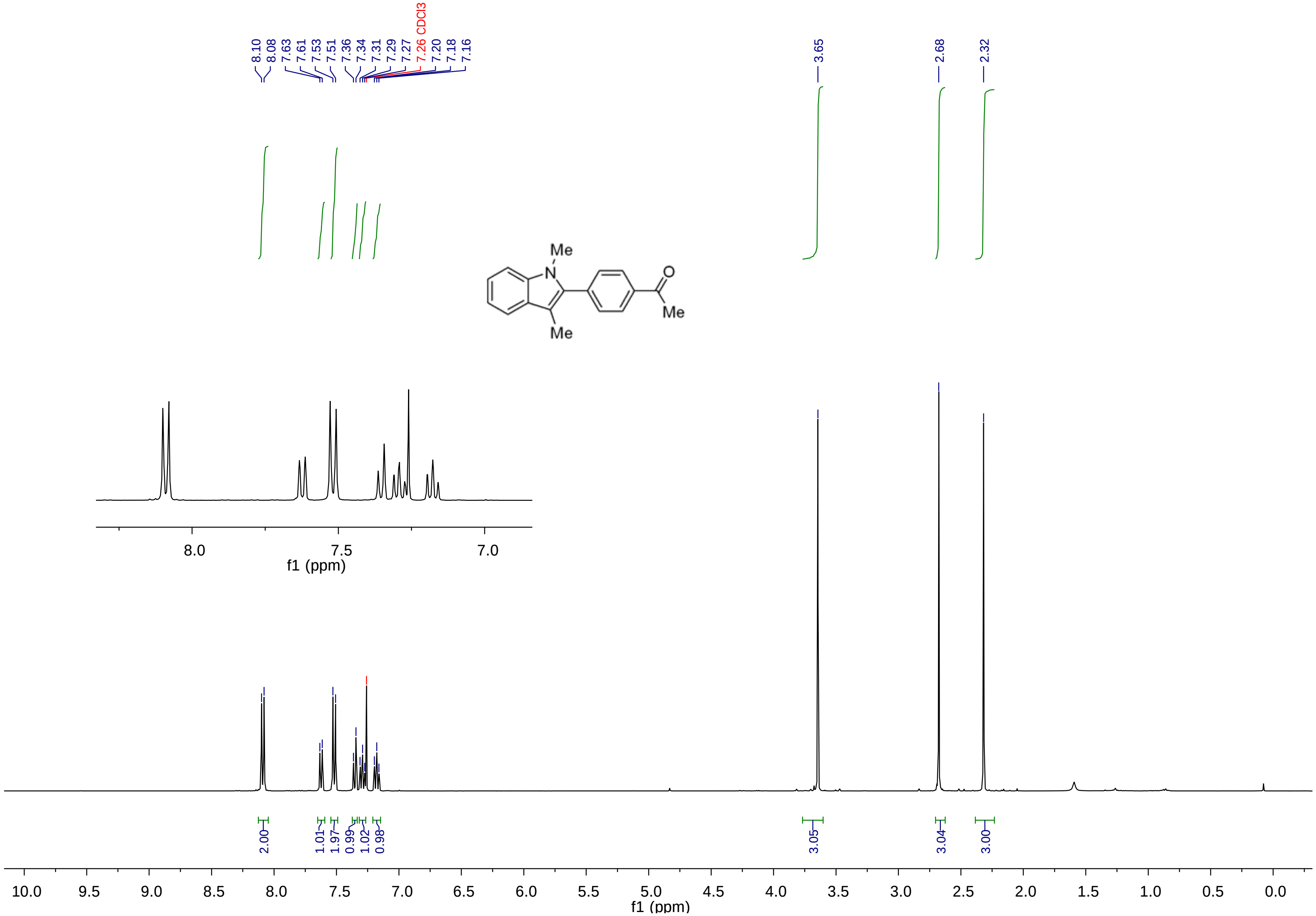

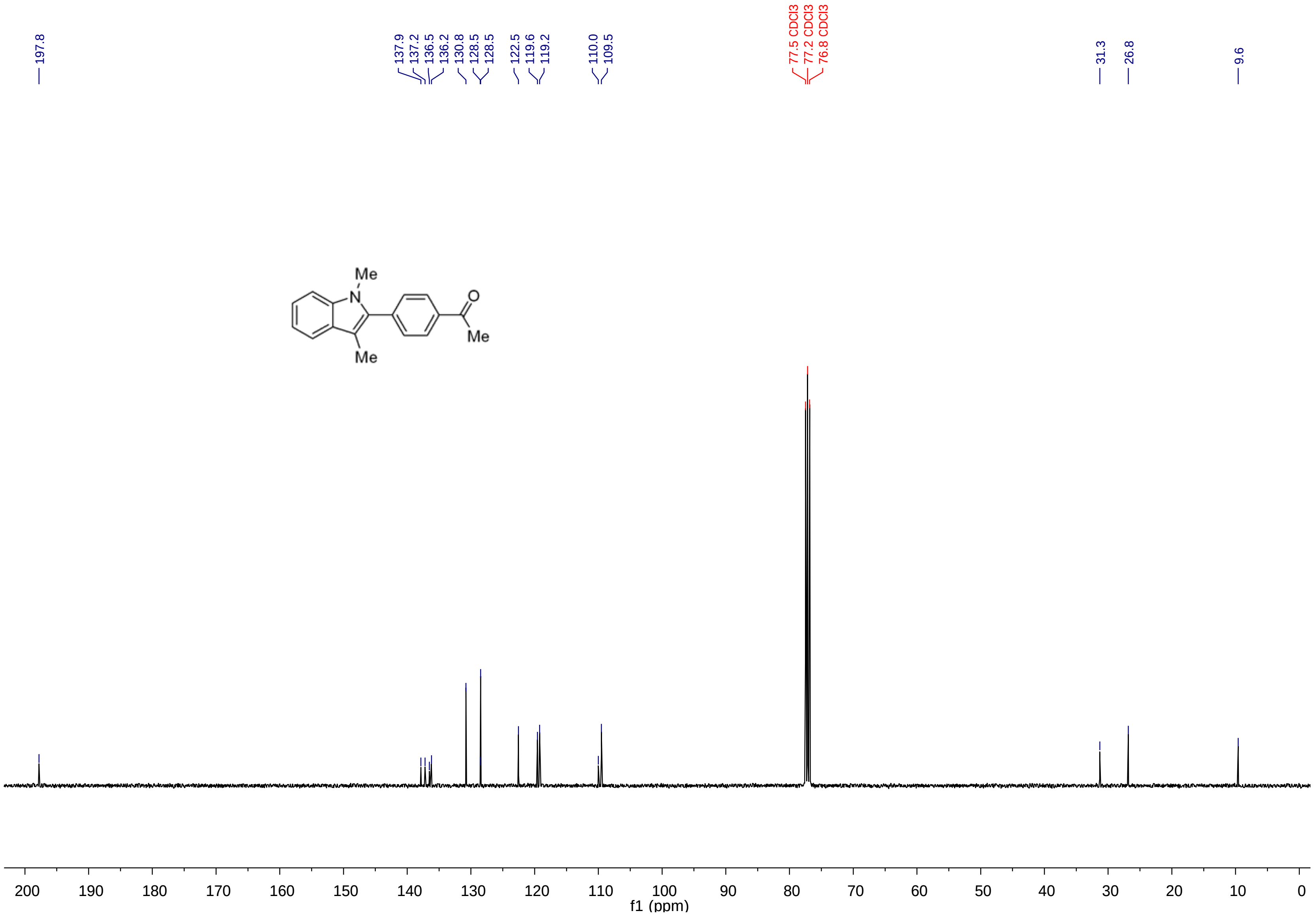
197.8
137.9
137.2
136.5
136.2
130.8
128.5
128.5
122.5
119.6
119.2
110.0
109.5
77.5 CDCl3
77.2 CDCl3
76.8 CDCl3
31.3
26.8
9.6
Me
N
O
Me
Me
200
190
180
170
160
150
140
130
120
110
100
90
80
70
60
50
40
30
20
10
0
f1 (ppm)

2-(o-tolyl)-*1H*-indole (4a)

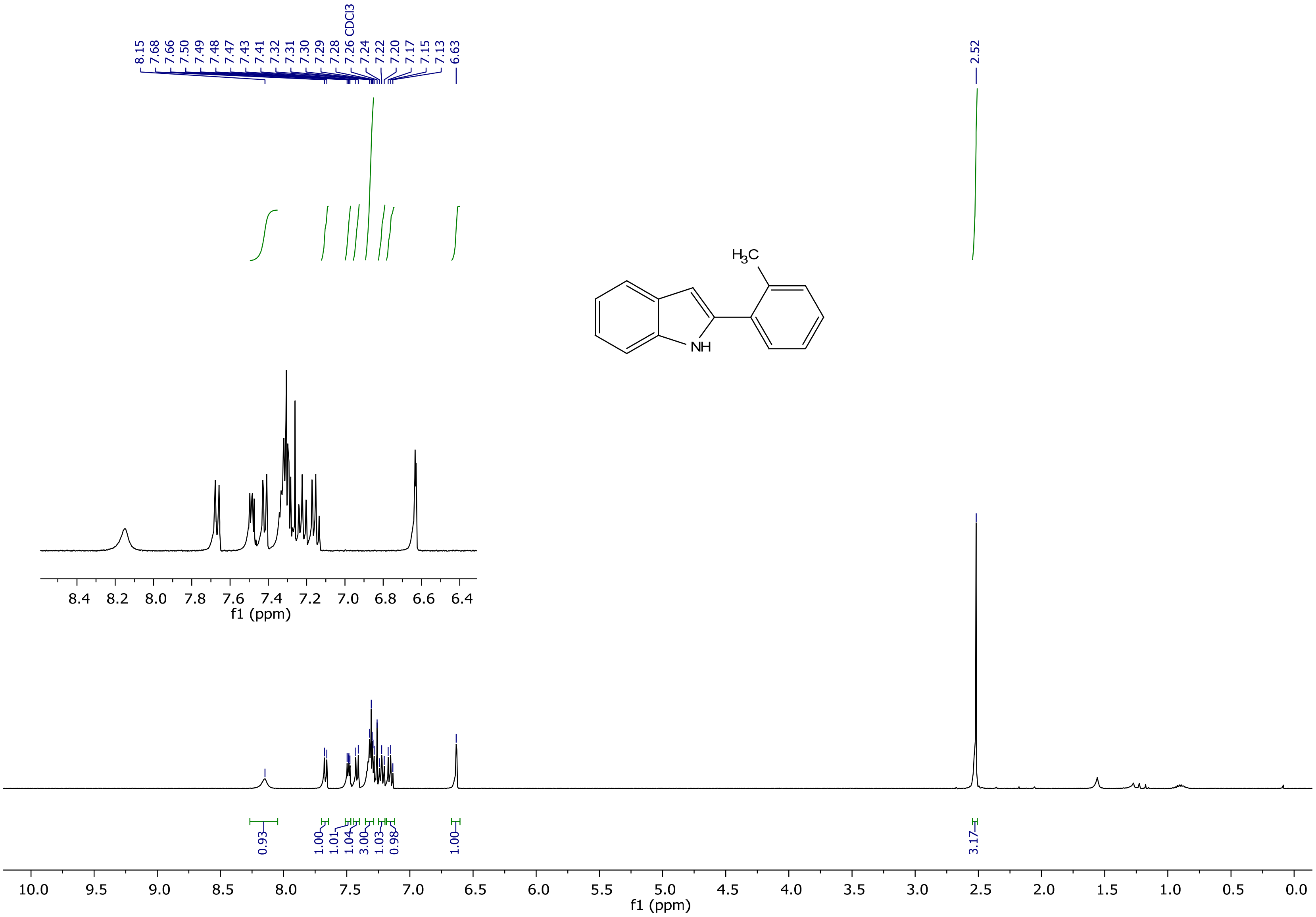

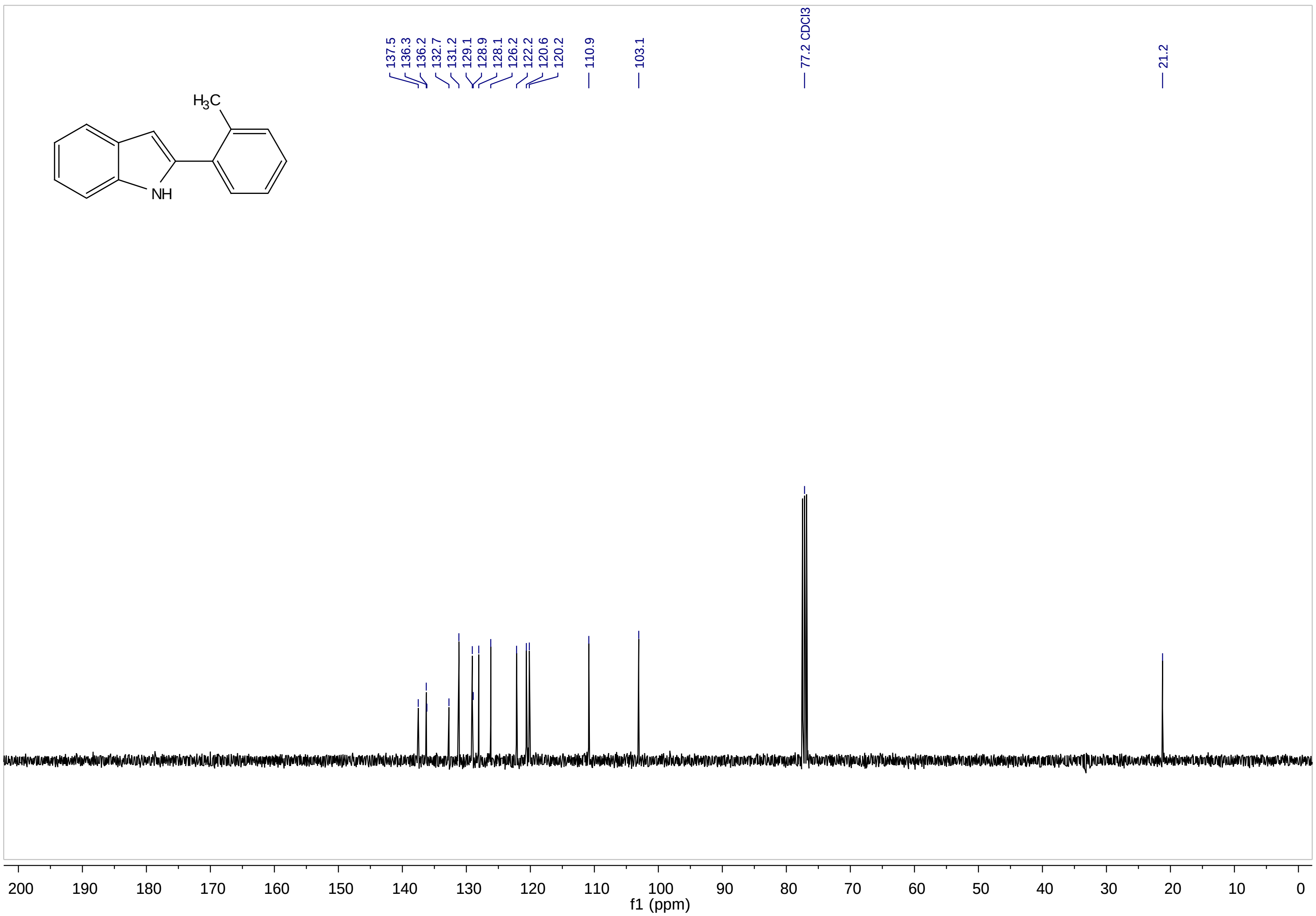
137.5
136.3
136.2
132.7
131.2
129.1
128.9
128.1
126.2
122.2
120.6
120.2
110.9
103.1
77.2 CDCl3
21.2
H3C
NH
200
190
180
170
160
150
140
130
120
110
100
90
80
70
60
50
40
30
20
10
0
f1 (ppm)

2-(2,4-dimethylphenyl)-*1H*-indole (4b)

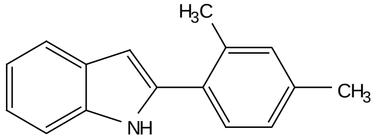

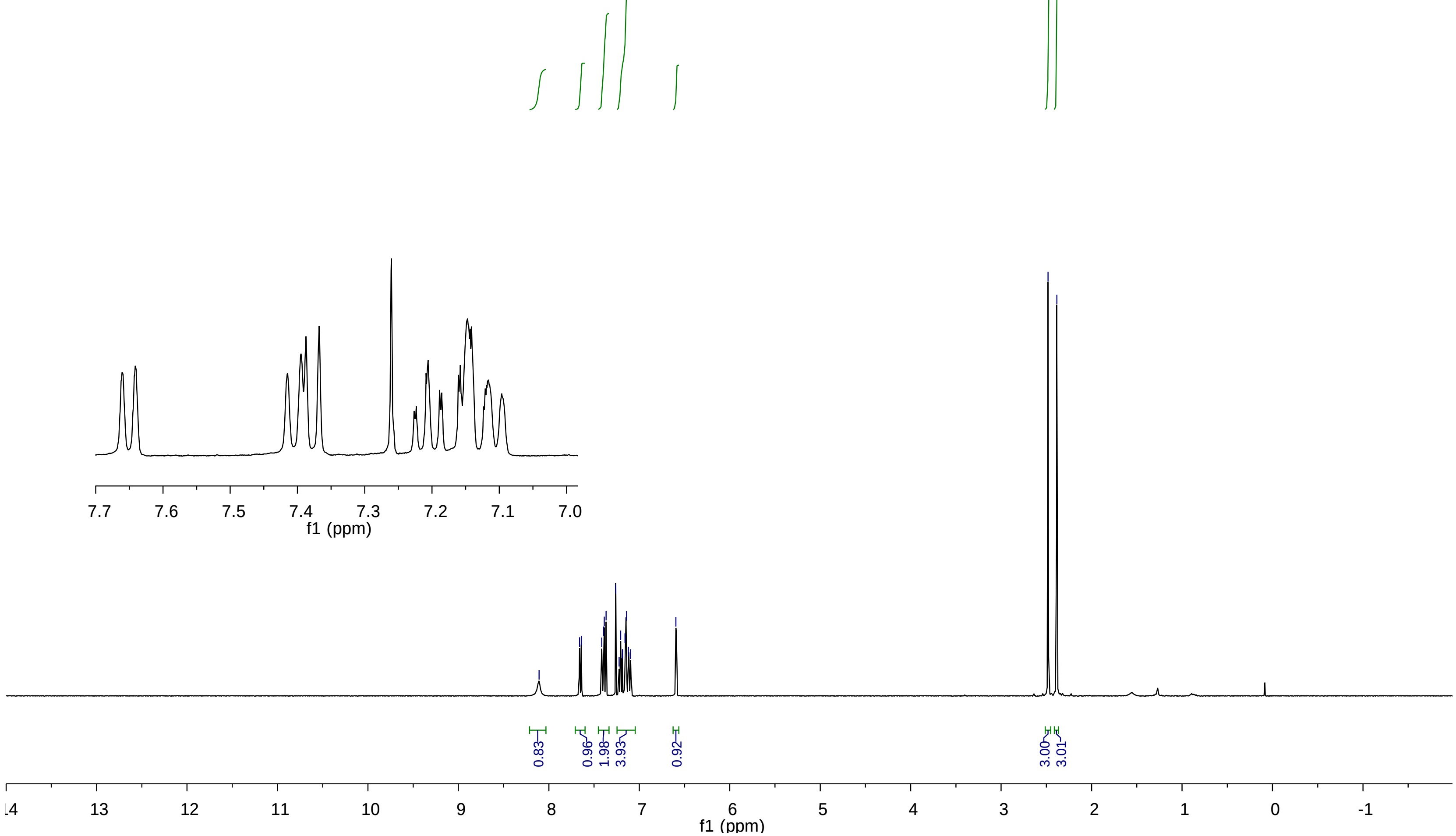

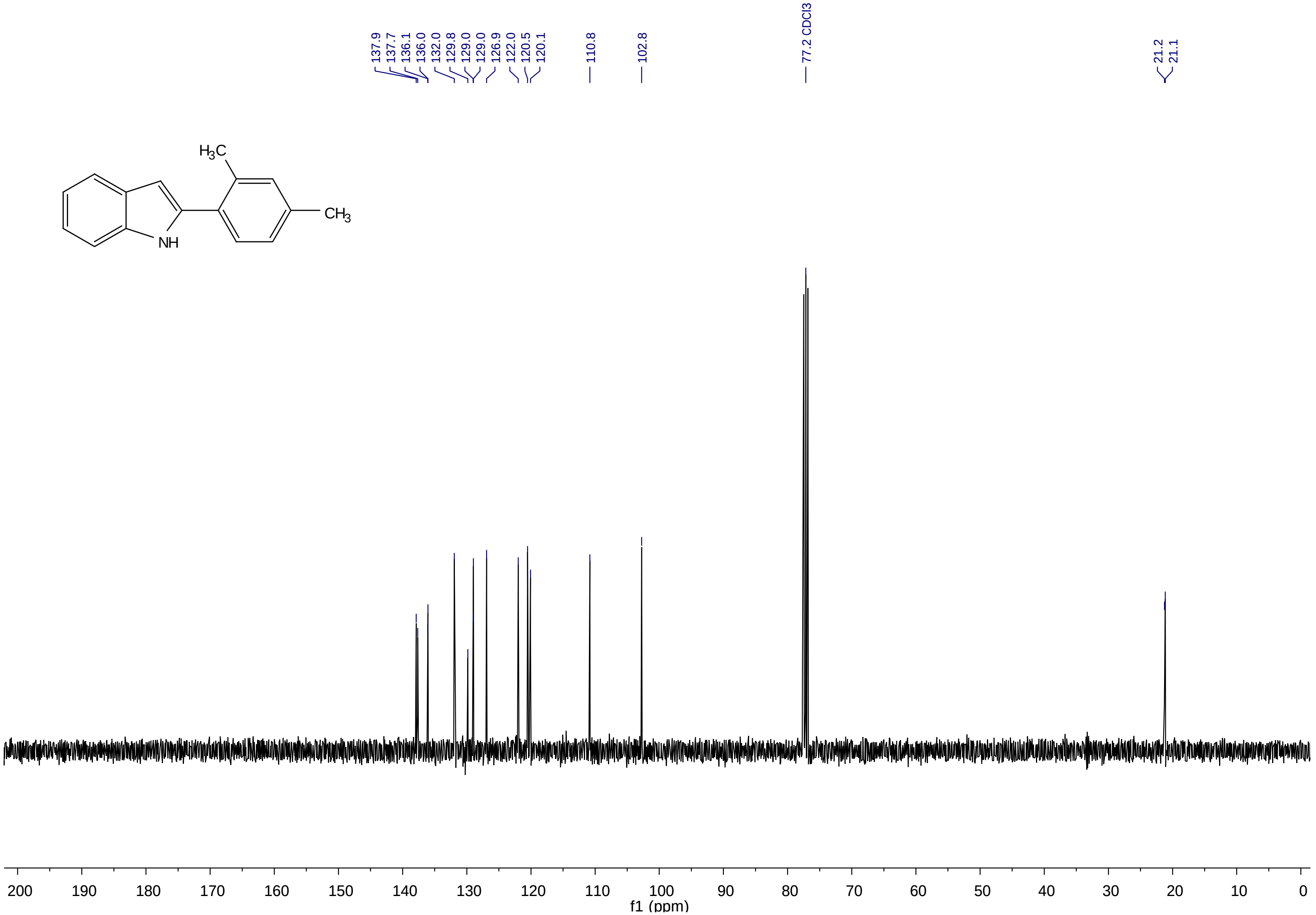
137.9
137.7
136.1
136.0
132.0
129.8
129.0
129.0
126.9
122.0
120.5
120.1
110.8
102.8
77.2 CDCl3
21.2
21.1
H3C
CH3
NH
200 190 180 170 160 150 140 130 120 110 100 90 80 70 60 50 40 30 20 10 0
f1 (ppm)

2-mesityl-*1H*-indole (4c –C-2)

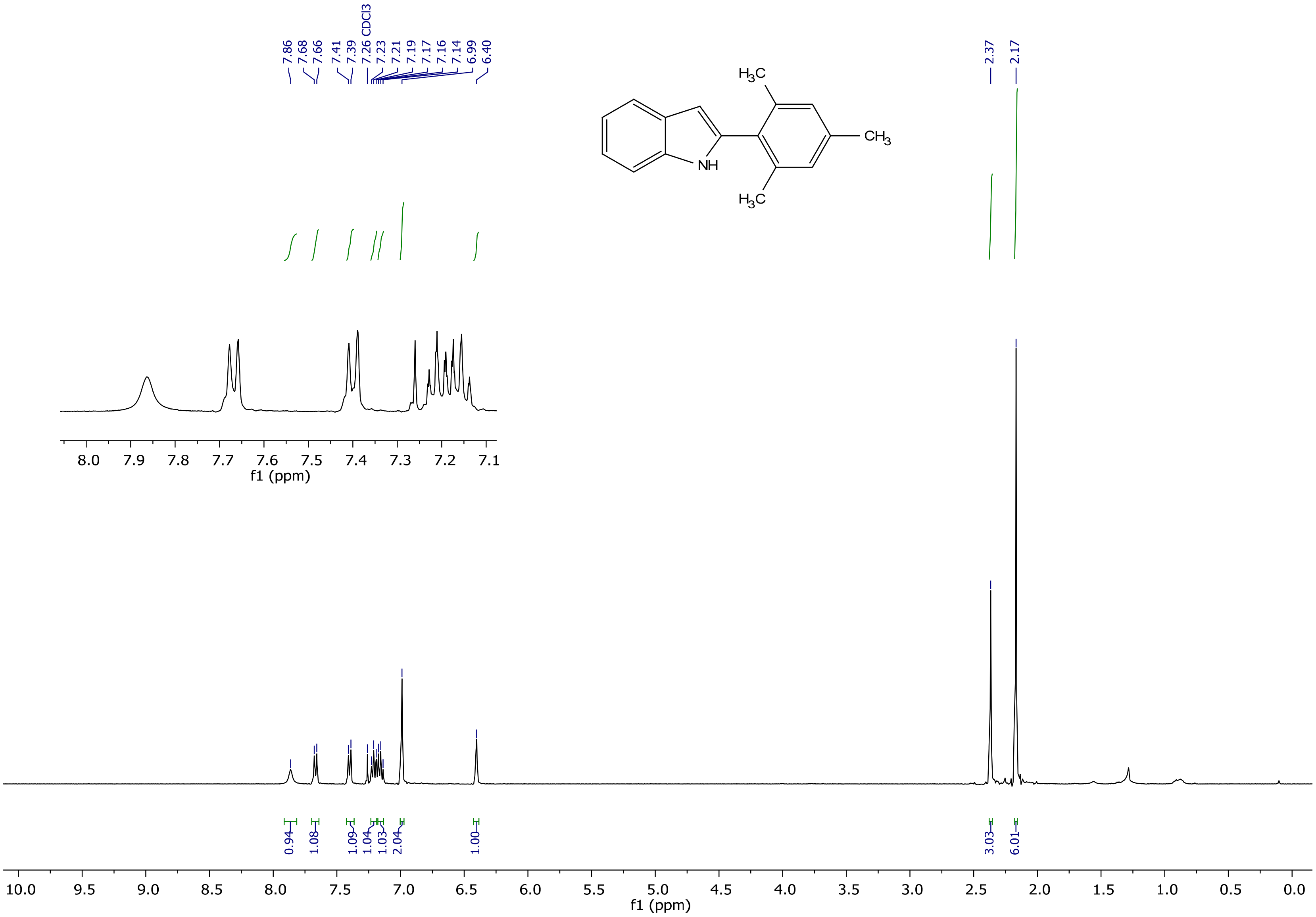

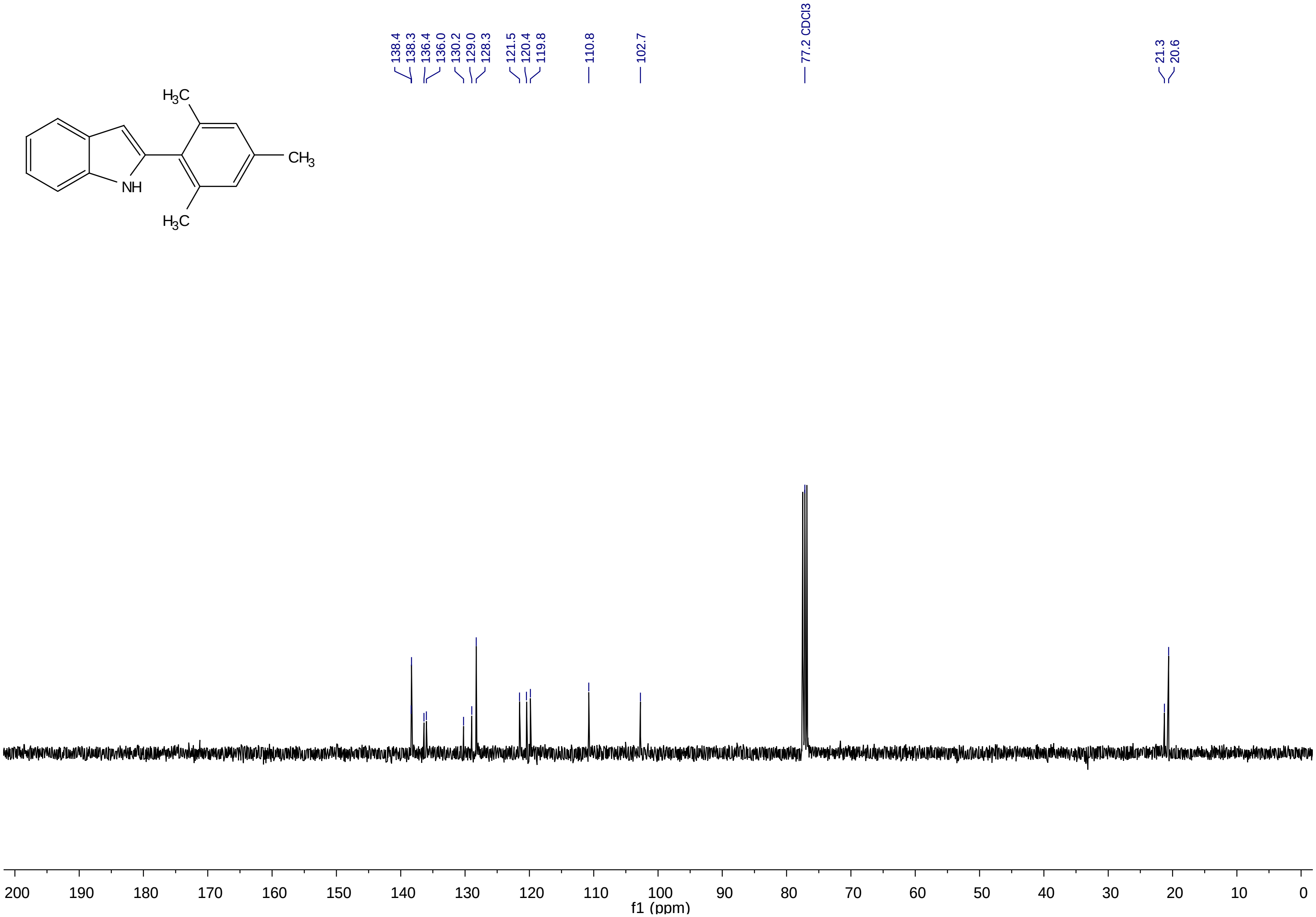
H3C
CH3
NH
H3C
138.4
138.3
136.4
136.0
130.2
129.0
128.3
121.5
120.4
119.8
110.8
102.7
77.2 CDCl3
21.3
20.6
200 190 180 170 160 150 140 130 120 110 100 90 80 70 60 50 40 30 20 10 0
f1 (ppm)

3-mesityl-*1H*-indole (4c – C-3)

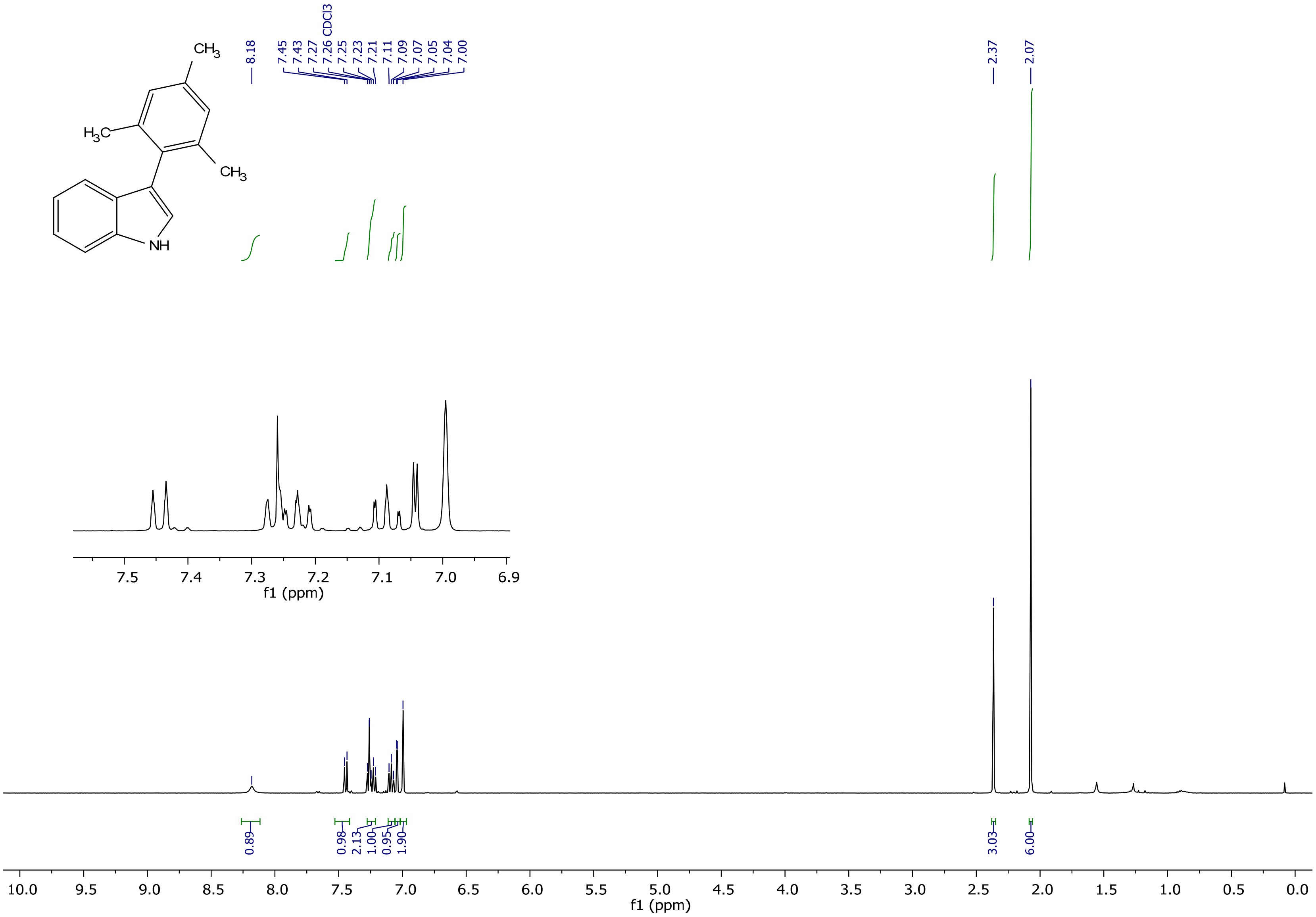

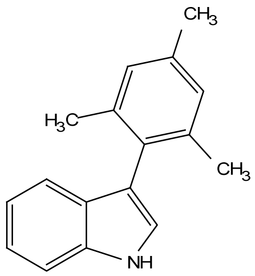
CH3
H3C
CH3
NH

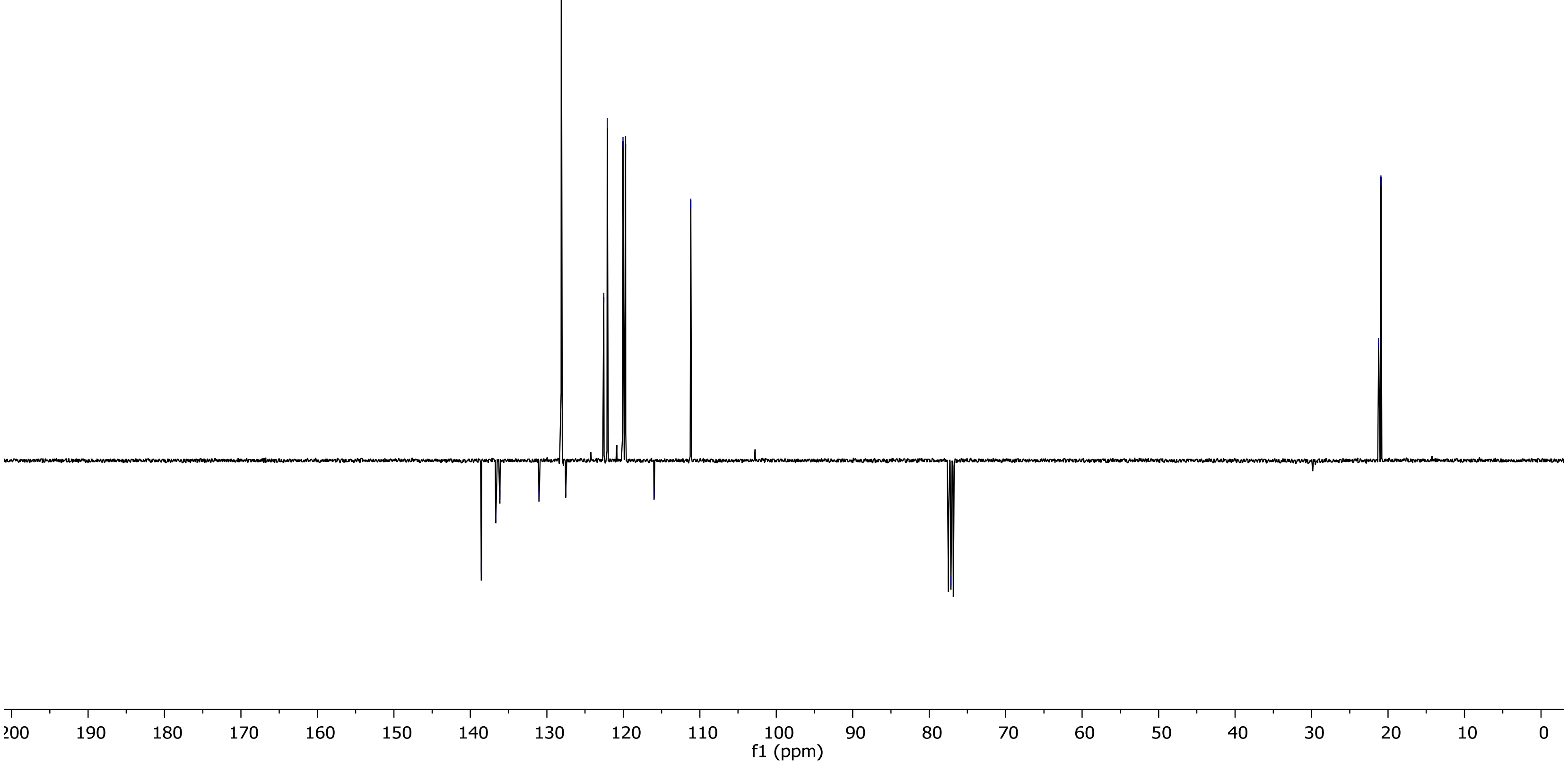
138.57
136.68
136.15
131.03
128.12
127.54
122.56
122.09
120.05
119.72
115.99
111.19
77.16 CDCl3
21.22
20.92
200
190
180
170
160
150
140
130
120
110
100
90
80
70
60
50
40
30
20
10
0
f1 (ppm)

2-(4-methoxy-2-methylphenyl)-*1H*-indole (4d)

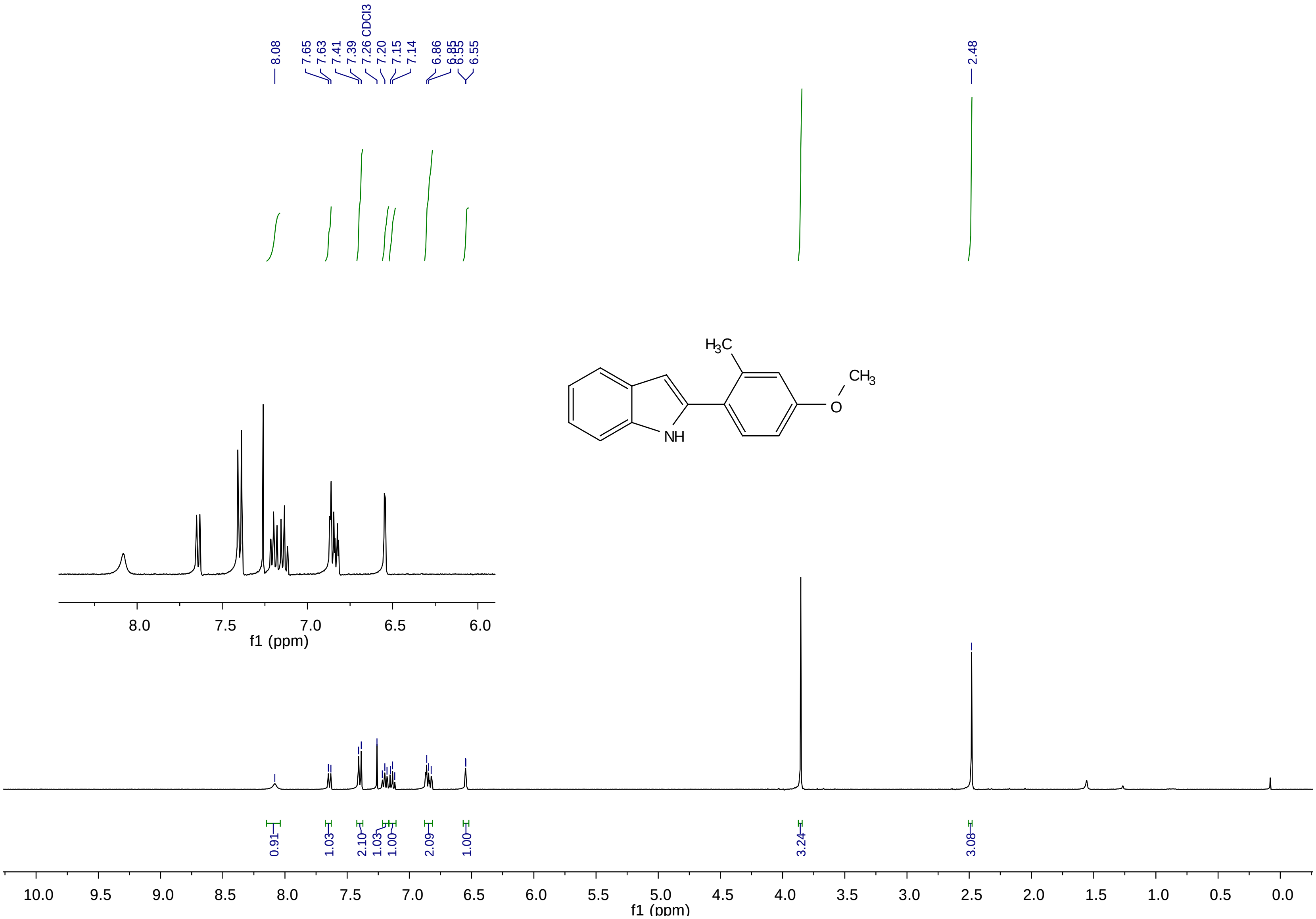

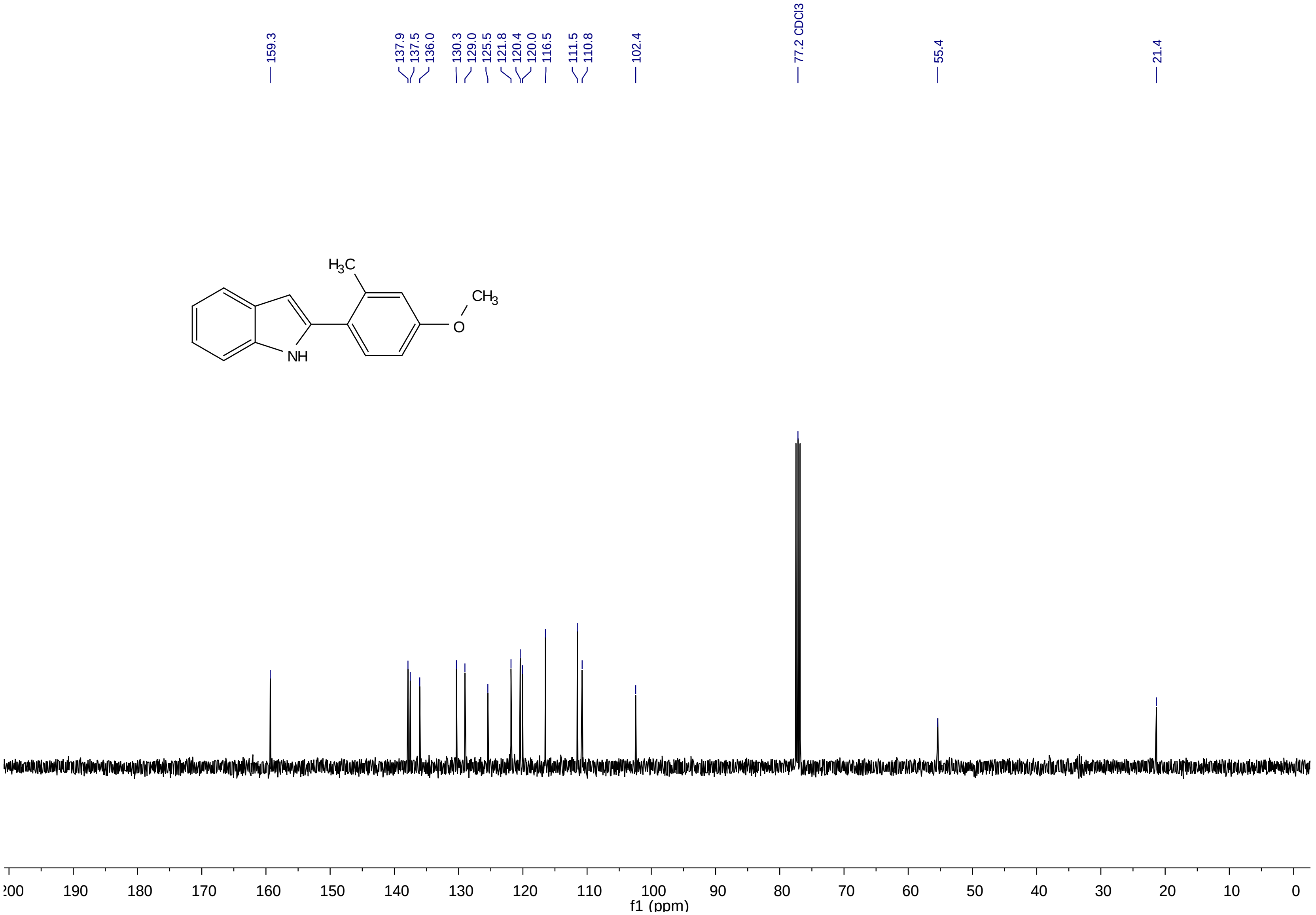
159.3
137.9
137.5
136.0
130.3
129.0
125.5
121.8
120.4
120.0
116.5
111.5
110.8
102.4
77.2 CDCl3
55.4
21.4
H3C
CH3
O
NH
200
190
180
170
160
150
140
130
120
110
100
90
80
70
60
50
40
30
20
10
0
f1 (ppm)

2-(4-methoxyphenyl)-3-methyl-1*H*-indole (4e)

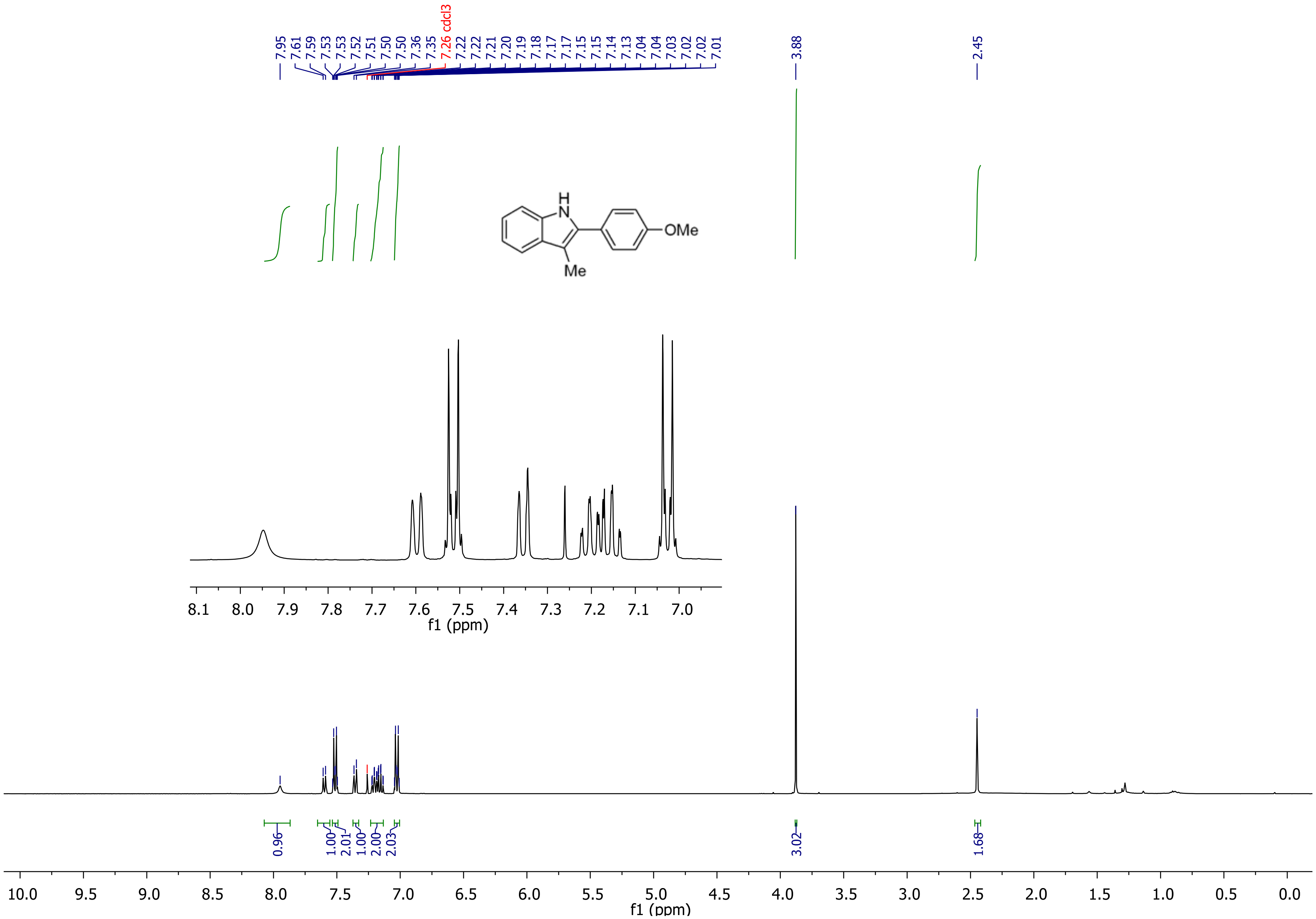

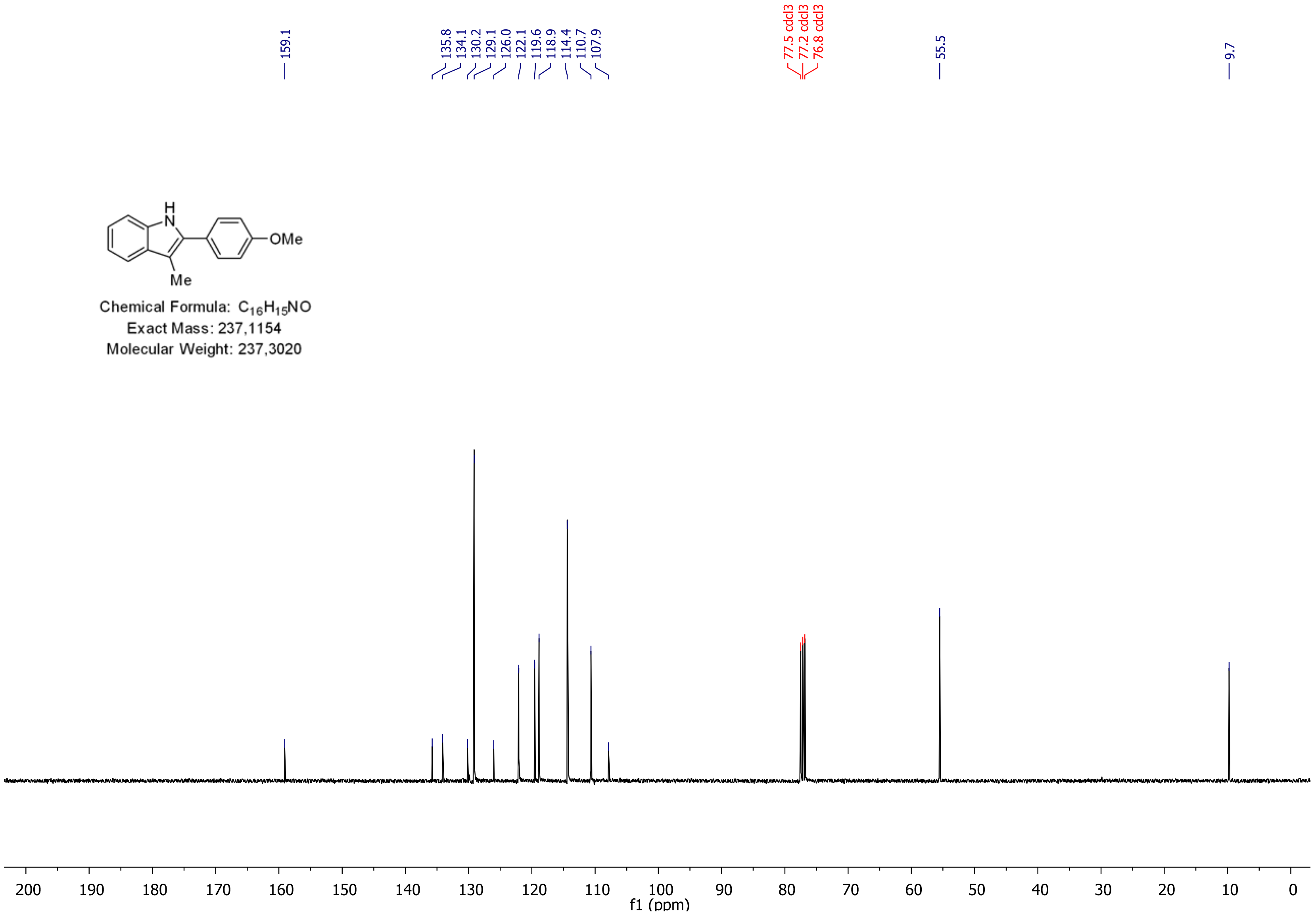
159.1
135.8
134.1
130.2
129.1
126.0
122.1
119.6
118.9
114.4
110.7
107.9
77.5 cdcl3
77.2 cdcl3
76.8 cdcl3
55.5
9.7
OMe
Me
Chemical Formula: C16H15NO
Exact Mass: 237,1154
Molecular Weight: 237,3020
200
190
180
170
160
150
140
130
120
110
100
90
80
70
60
50
40
30
20
10
0
f1 (ppm)

6-fluoro-1-methyl-2-phenyl-indole (5a)

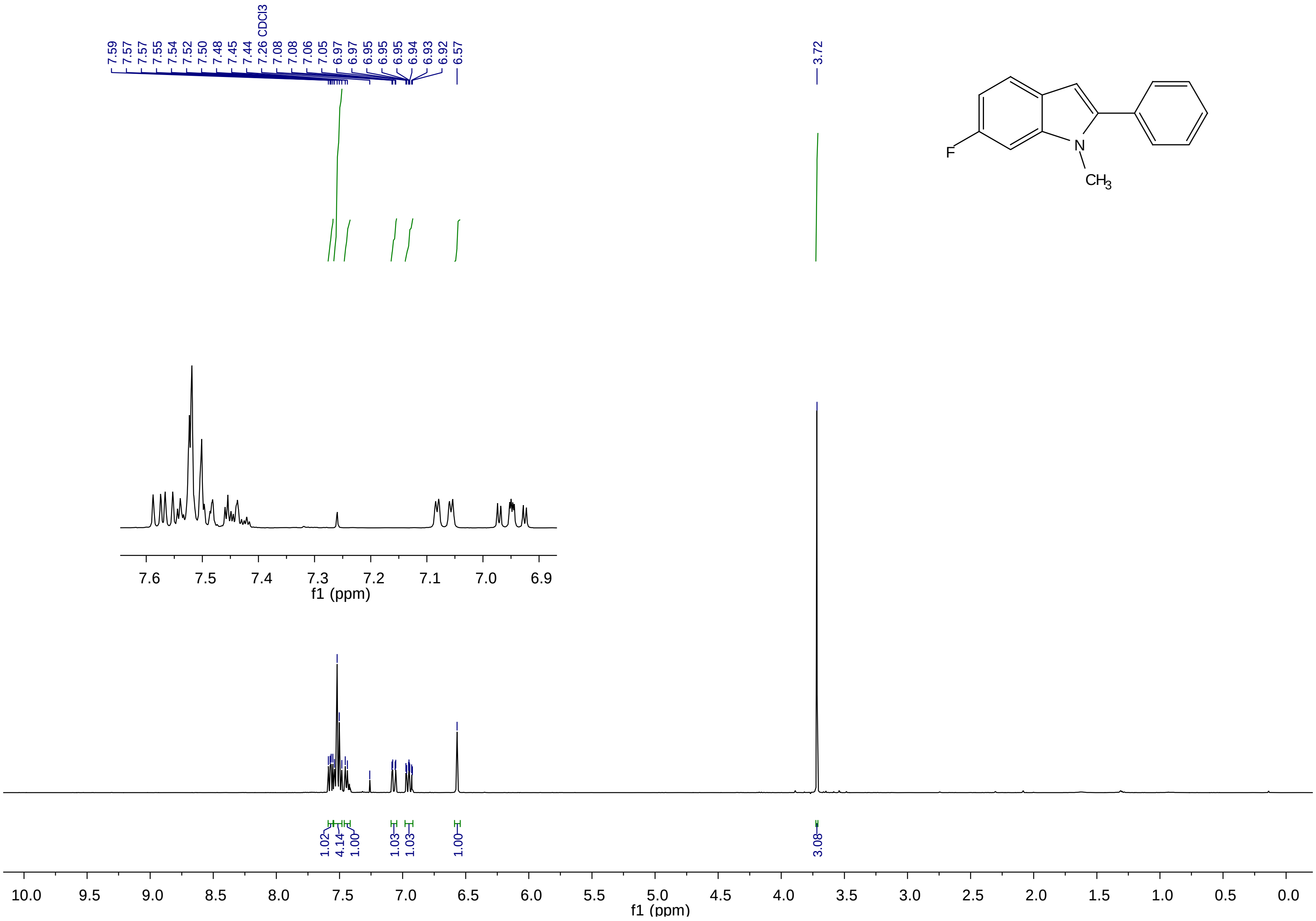

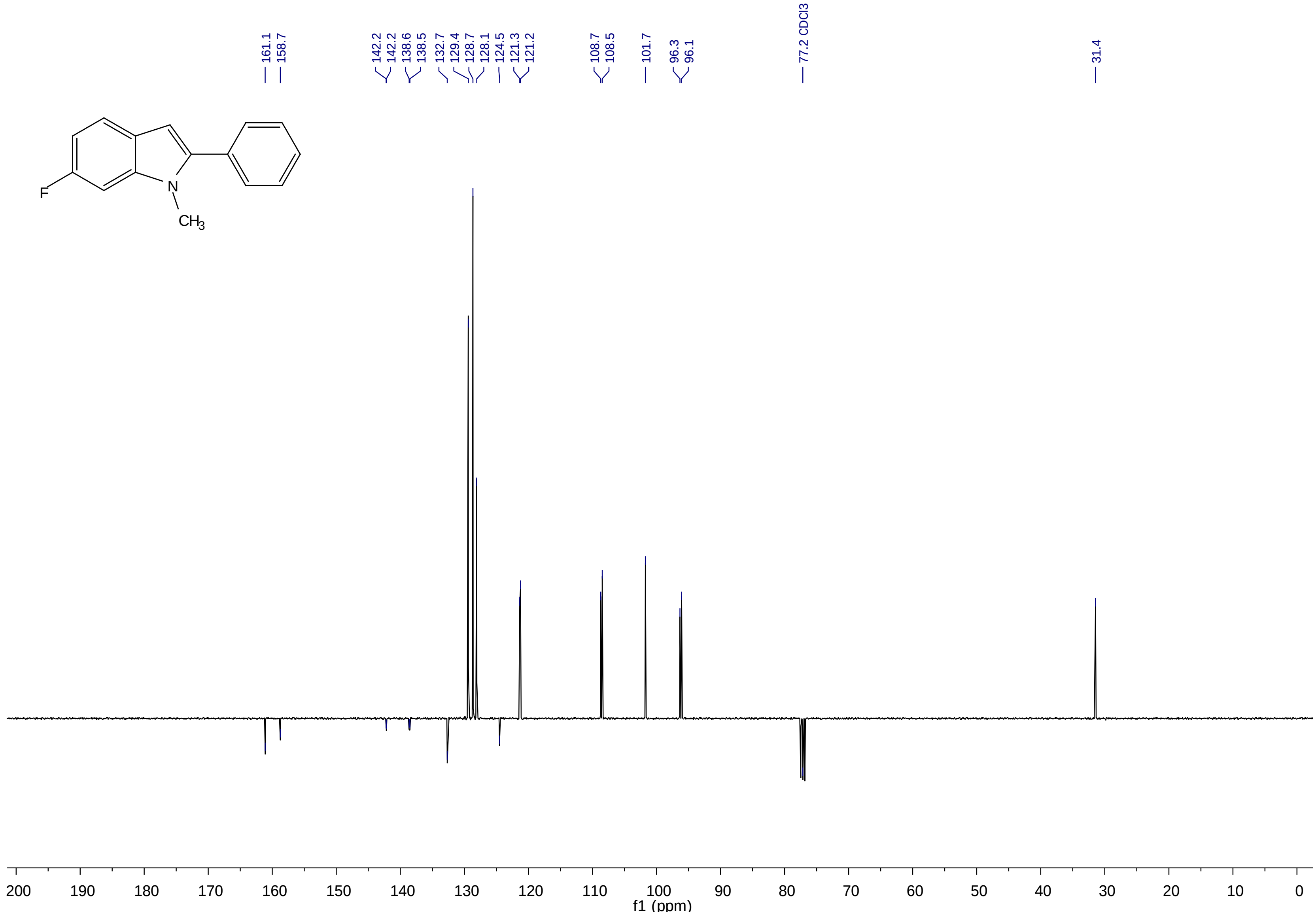
161.1
158.7
142.2
142.2
138.6
138.5
132.7
129.4
128.7
128.1
124.5
121.3
121.2
108.7
108.5
101.7
96.3
96.1
77.2 CDCl3
31.4
F
N
CH3
200
190
180
170
160
150
140
130
120
110
100
90
80
70
60
50
40
30
20
10
0
f1 (ppm)

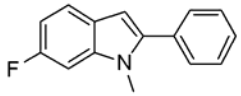

Chemical Formula: $C_{15}H_{12}FN$
Exact Mass: 225,0954

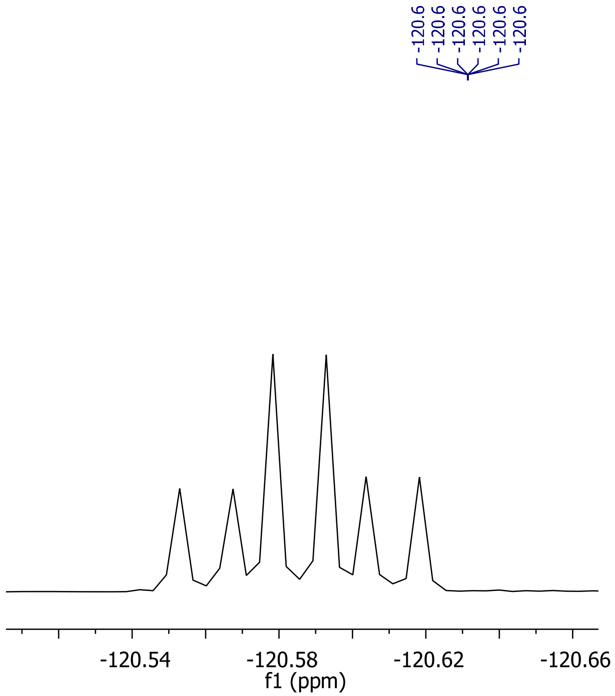

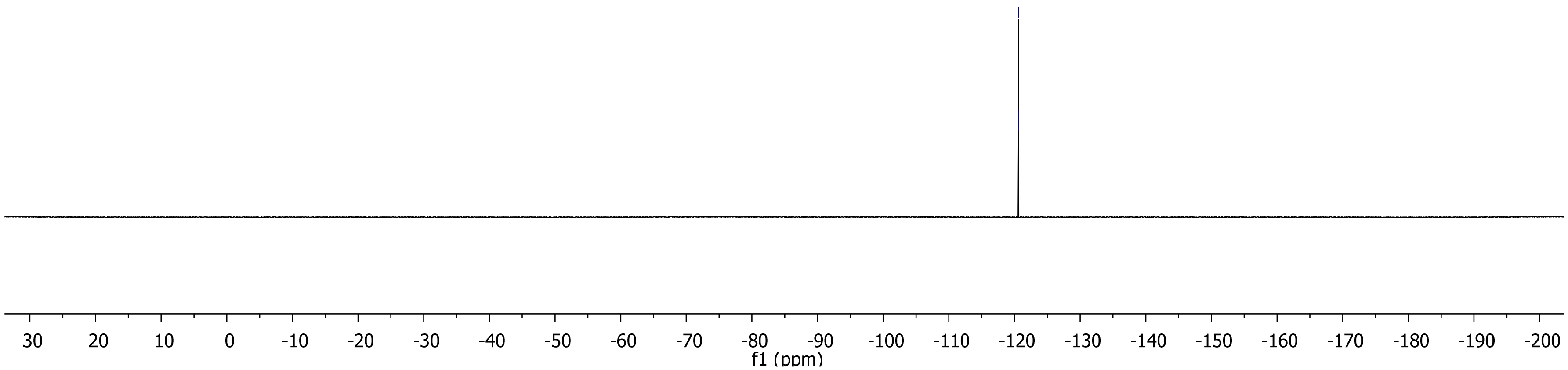

6-fluoro-2-(4-fluorophenyl)-1-methyl-1H-indole (5b)

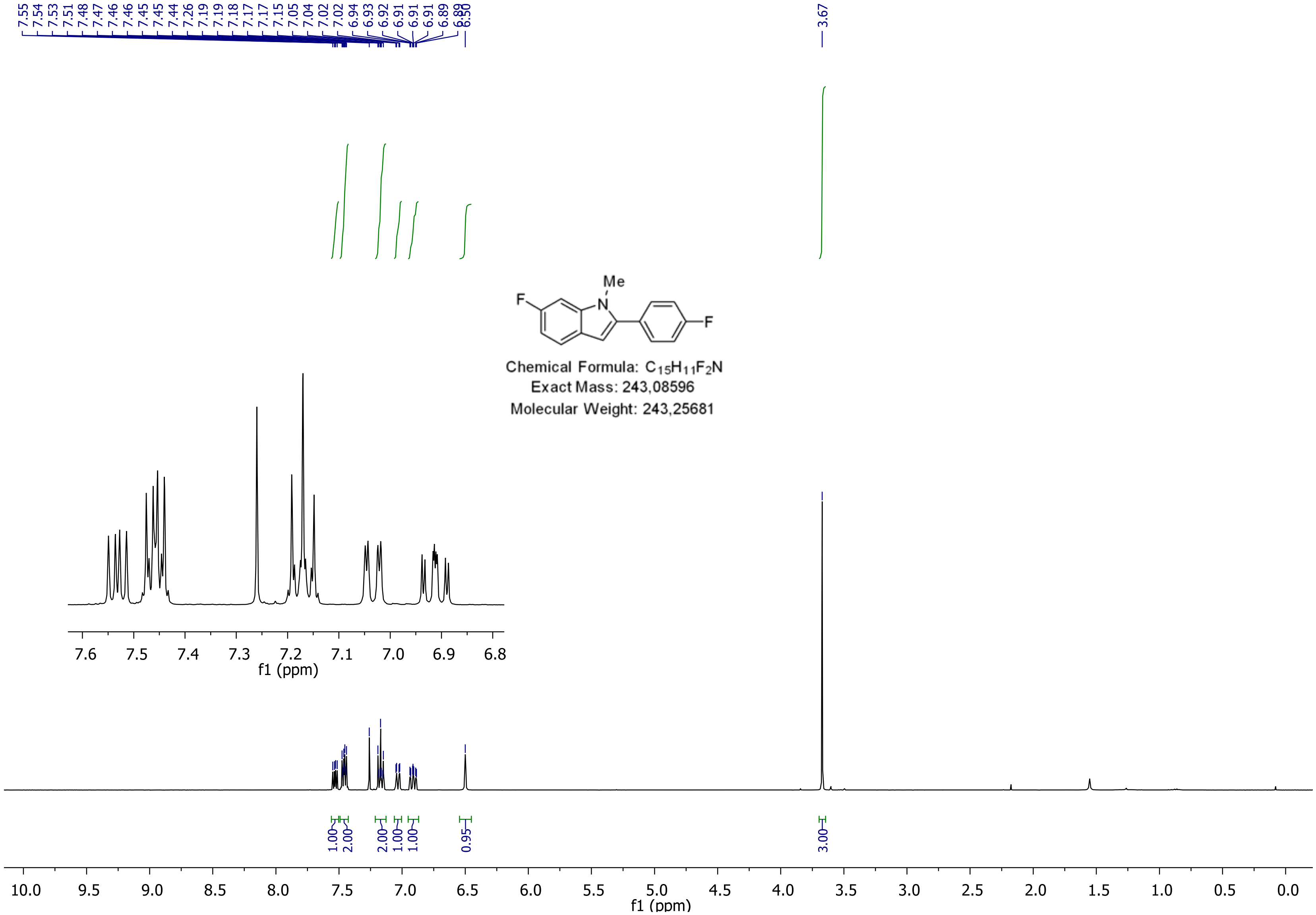

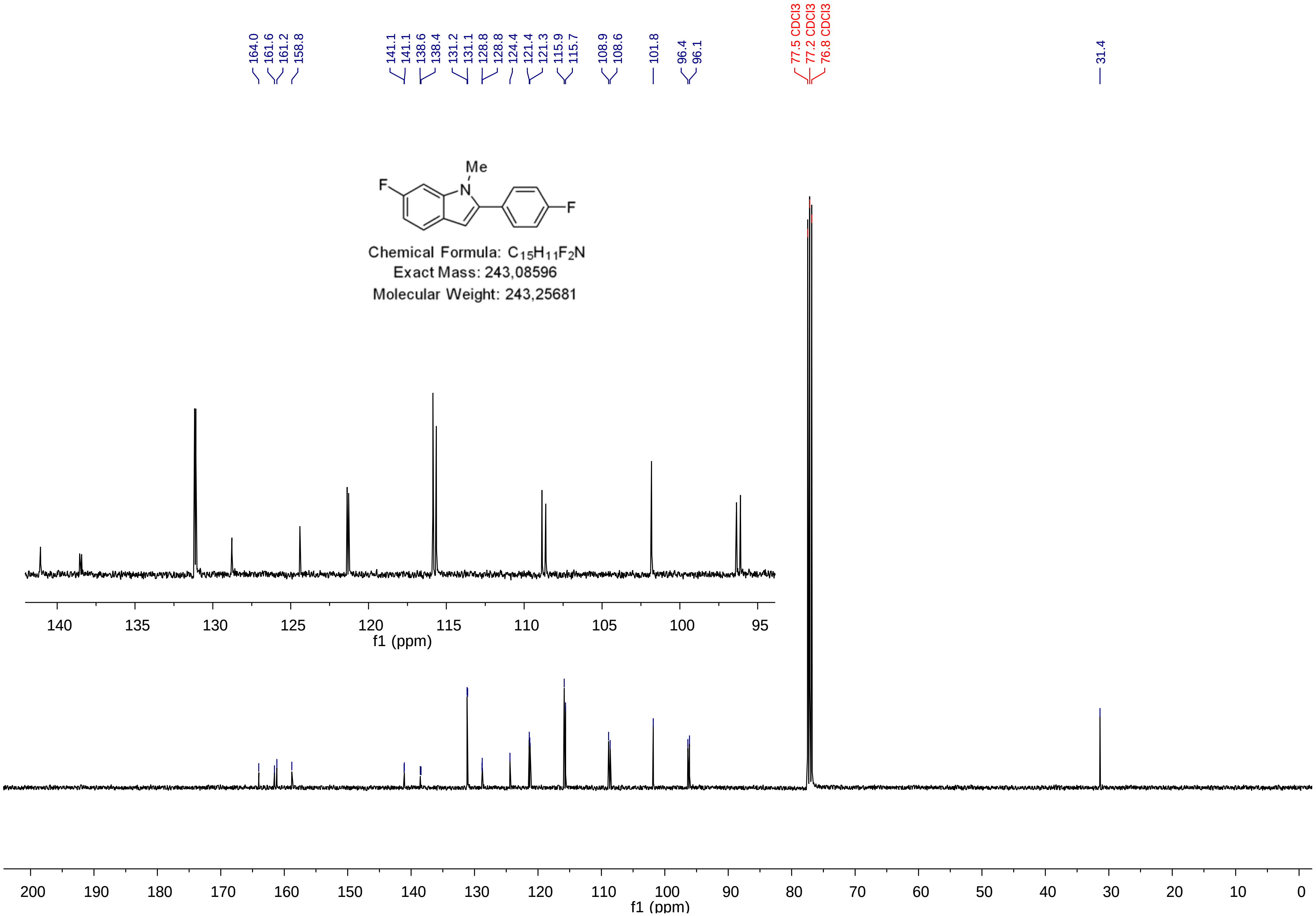
164.0
161.6
161.2
158.8
141.1
141.1
138.6
138.4
131.2
131.1
128.8
128.8
124.4
121.4
121.3
115.9
115.7
108.9
108.6
101.8
96.4
96.1
77.5 CDCl3
77.2 CDCl3
76.8 CDCl3
31.4
Me
N
F
F
Chemical Formula: C15H11F2N
Exact Mass: 243,08596
Molecular Weight: 243,25681
f1 (ppm)
f1 (ppm)

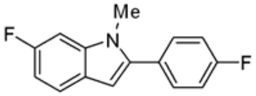

Chemical Formula: $C_{15}H_{11}F_2N$
Exact Mass: 243,08596
Molecular Weight: 243,25681

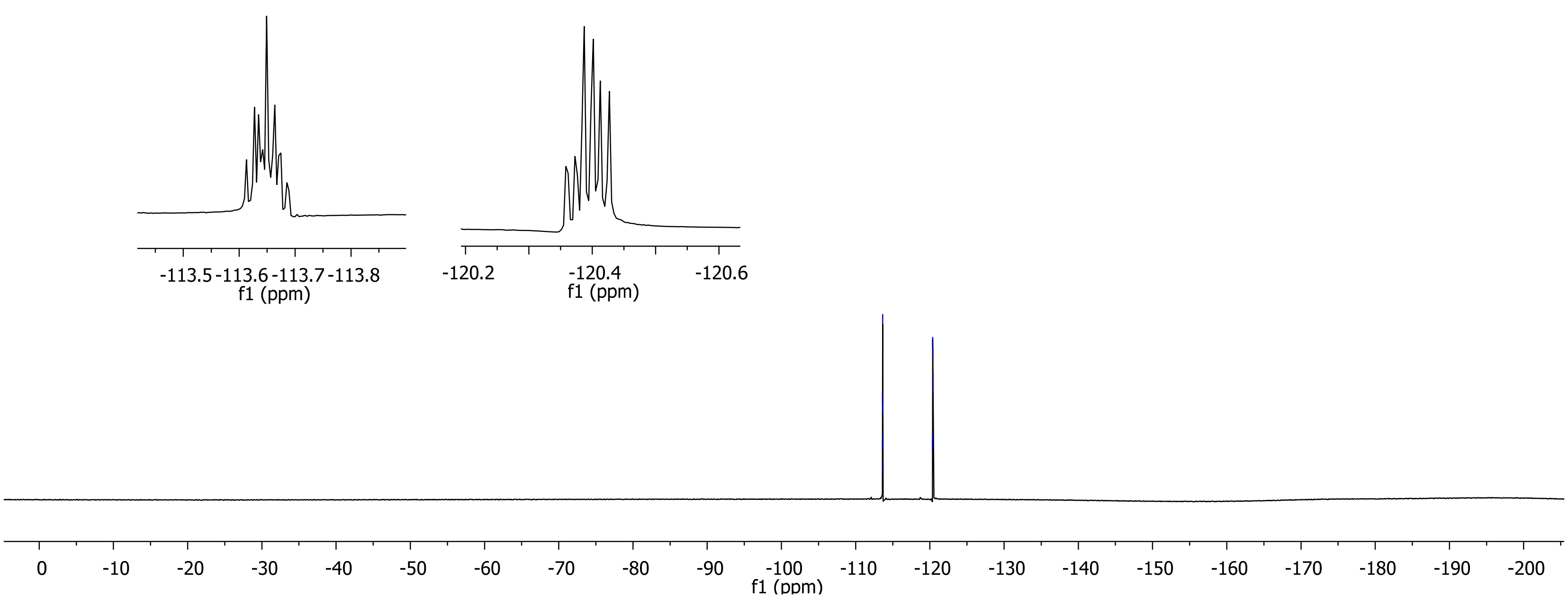

5-methoxy-1-methyl-2-phenyl-indole (5c)

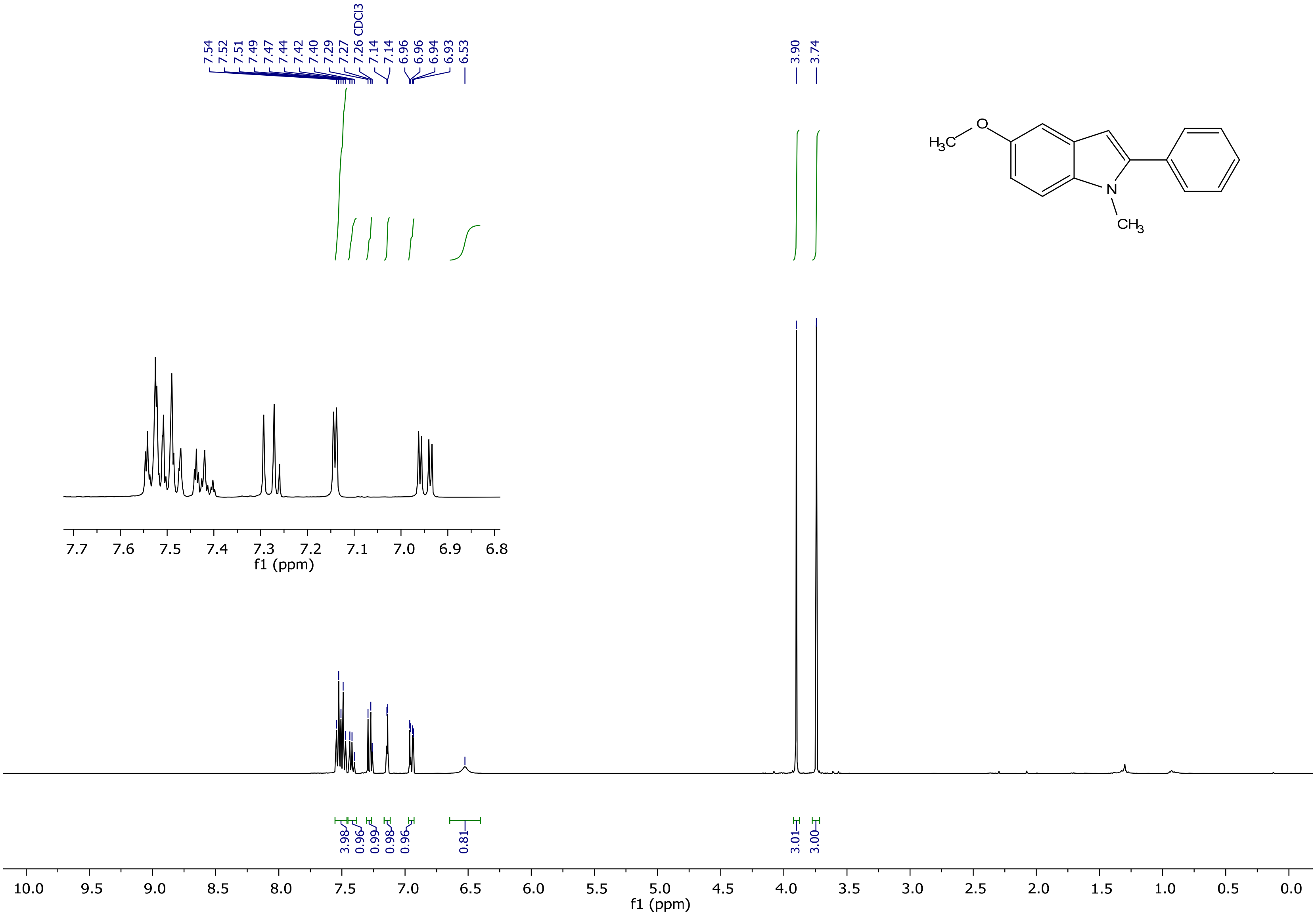

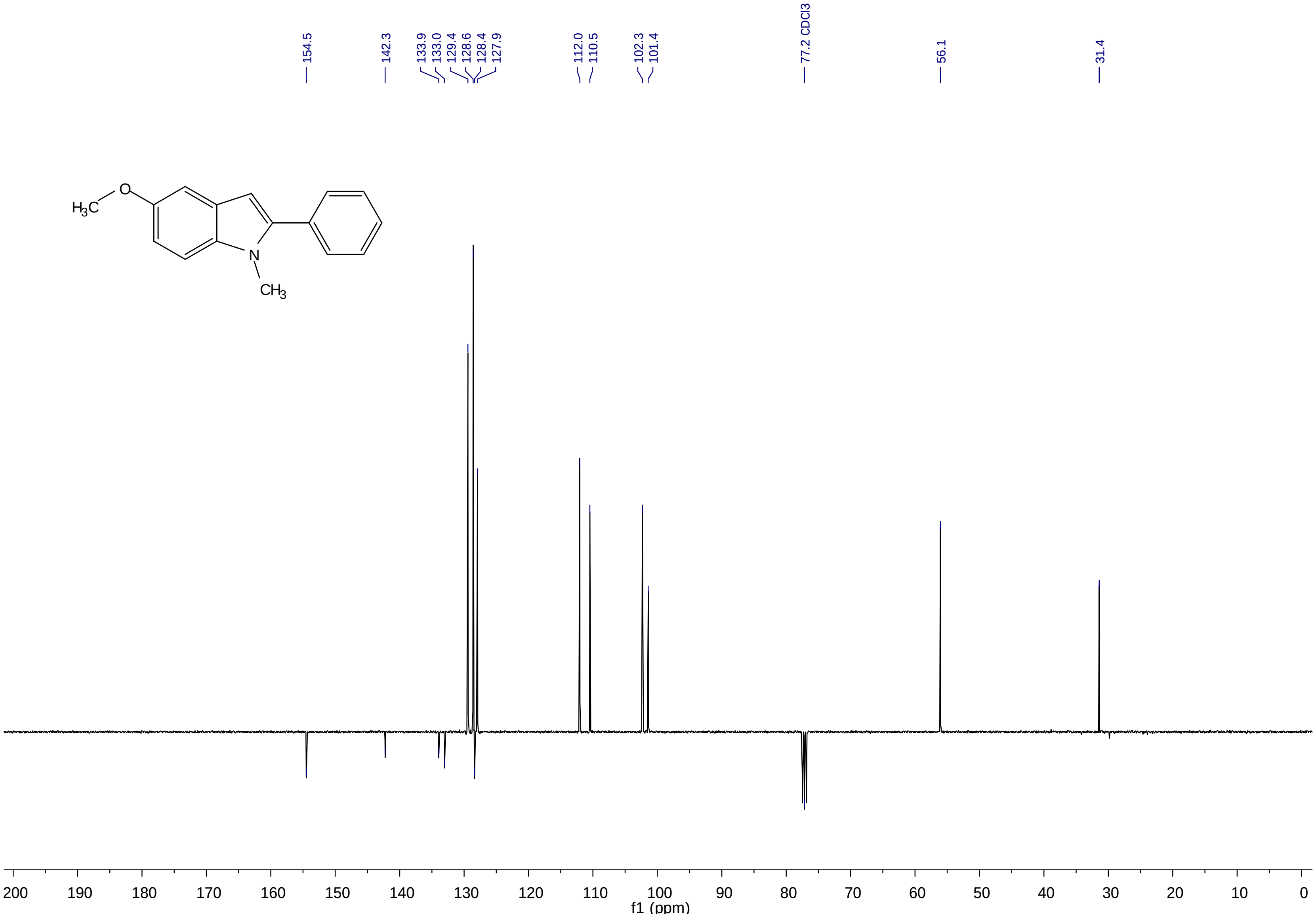
154.5
142.3
133.9
133.0
129.4
128.6
128.4
127.9
112.0
110.5
102.3
101.4
77.2 CDCl3
56.1
31.4
H3C
O
N
CH3
200 190 180 170 160 150 140 130 120 110 100 90 80 70 60 50 40 30 20 10 0
f1 (ppm)

methyl 1-methyl-2-phenyl-1H-indole-6-carboxylate (5d)

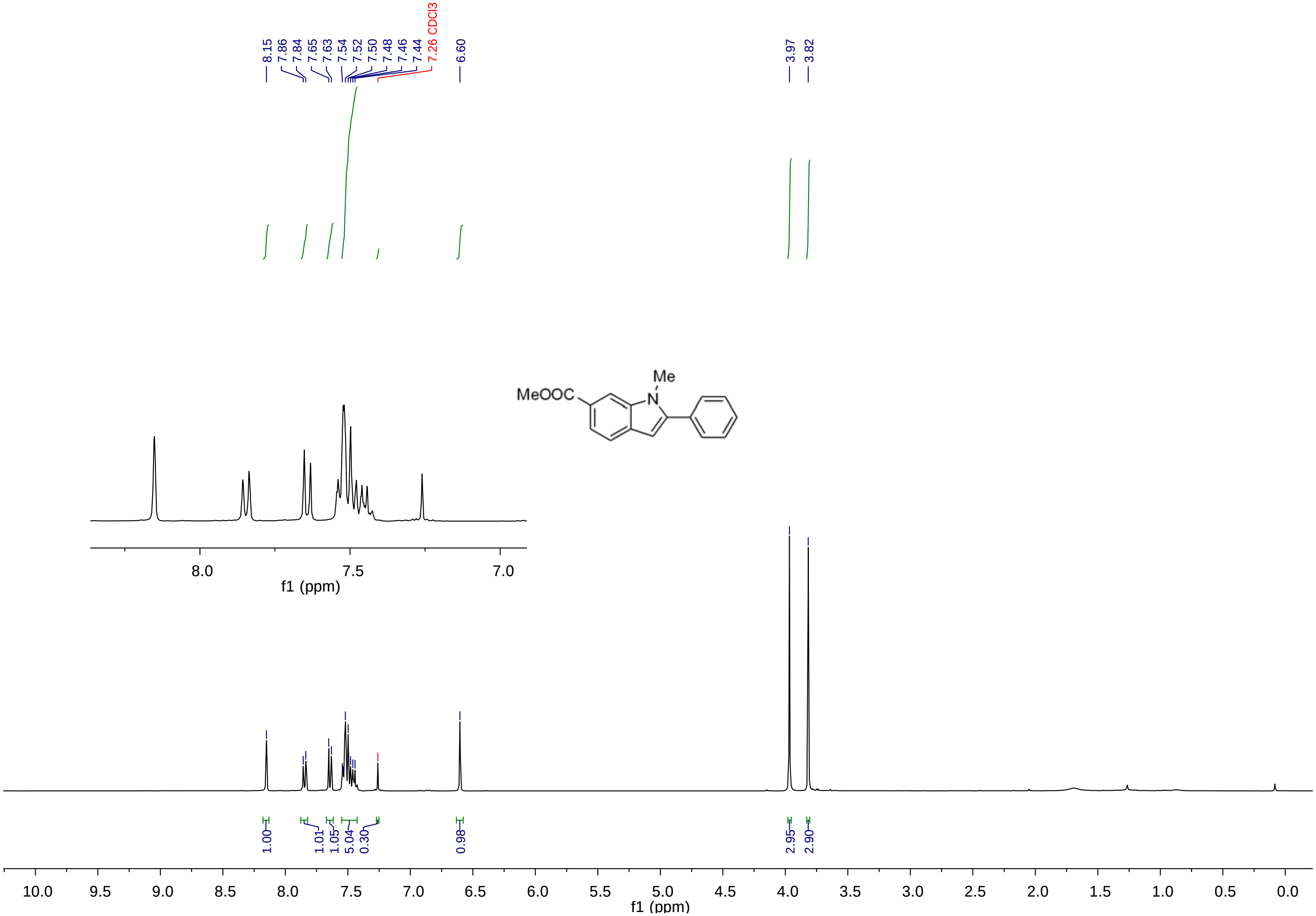

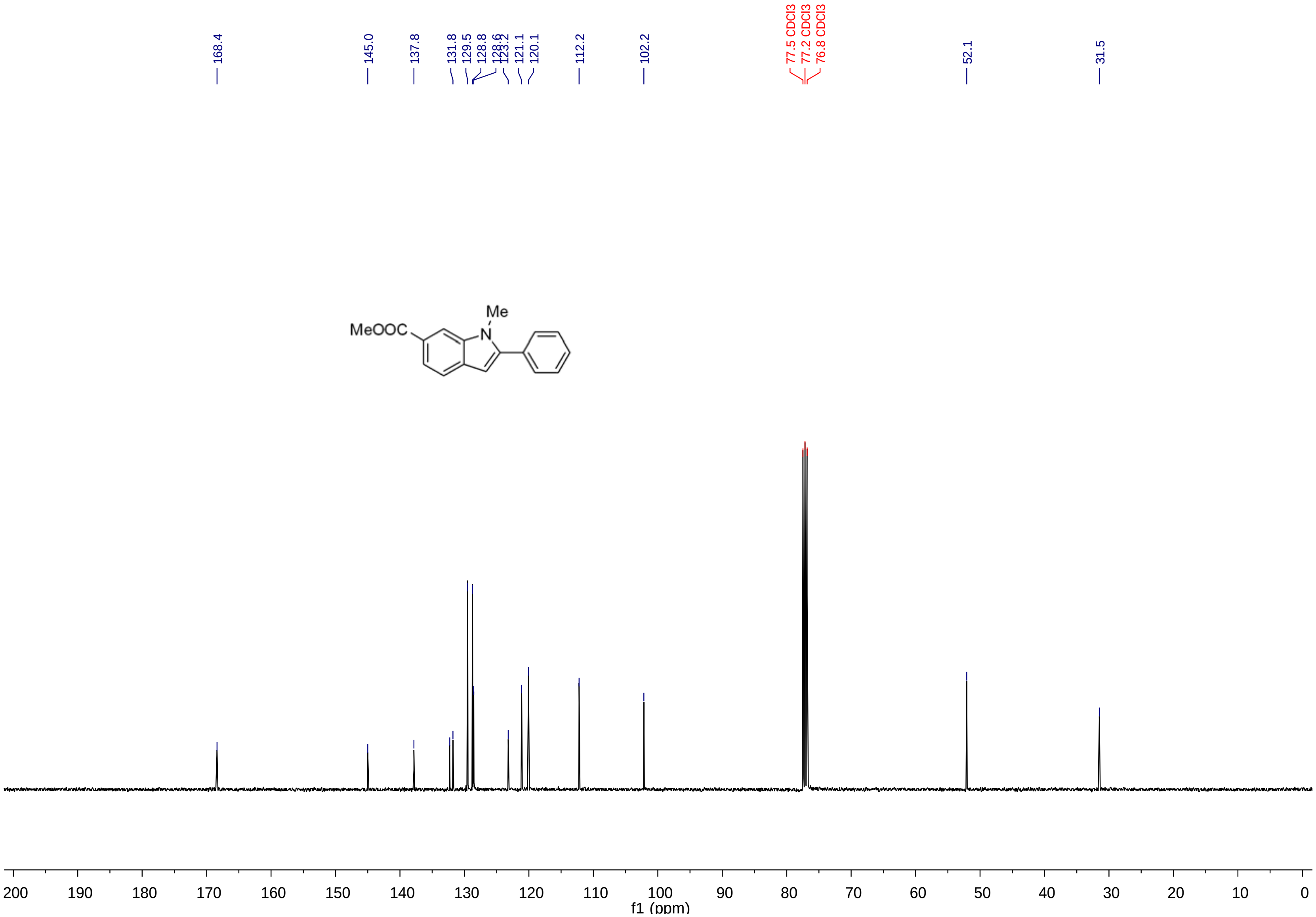
168.4
145.0
137.8
131.8
129.5
128.8
128.6
123.2
121.1
120.1
112.2
102.2
77.5 CDCl3
77.2 CDCl3
76.8 CDCl3
52.1
31.5
MeOOC
Me
N
200
190
180
170
160
150
140
130
120
110
100
90
80
70
60
50
40
30
20
10
0
f1 (ppm)

2-phenylbenzofuran (6a)

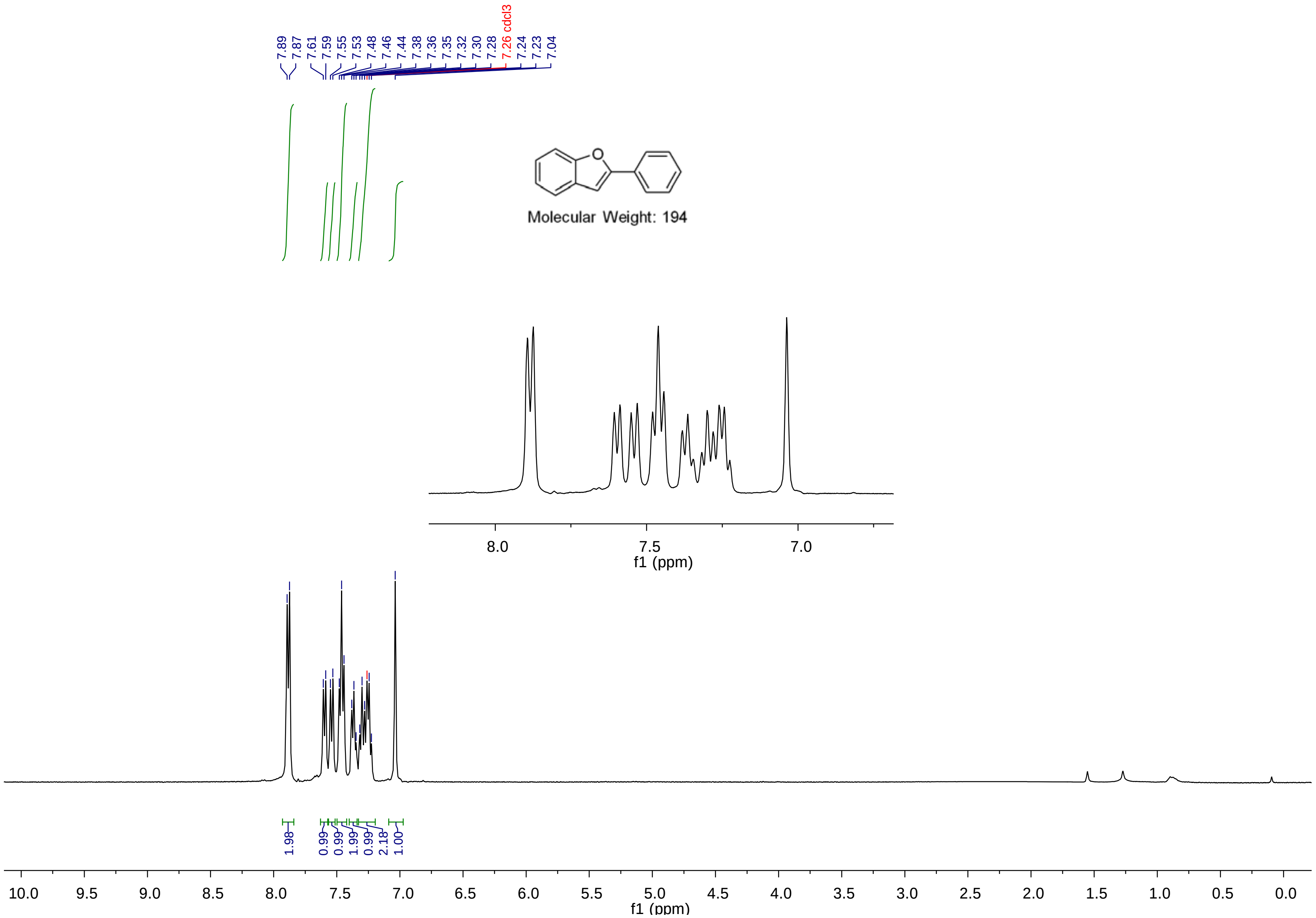

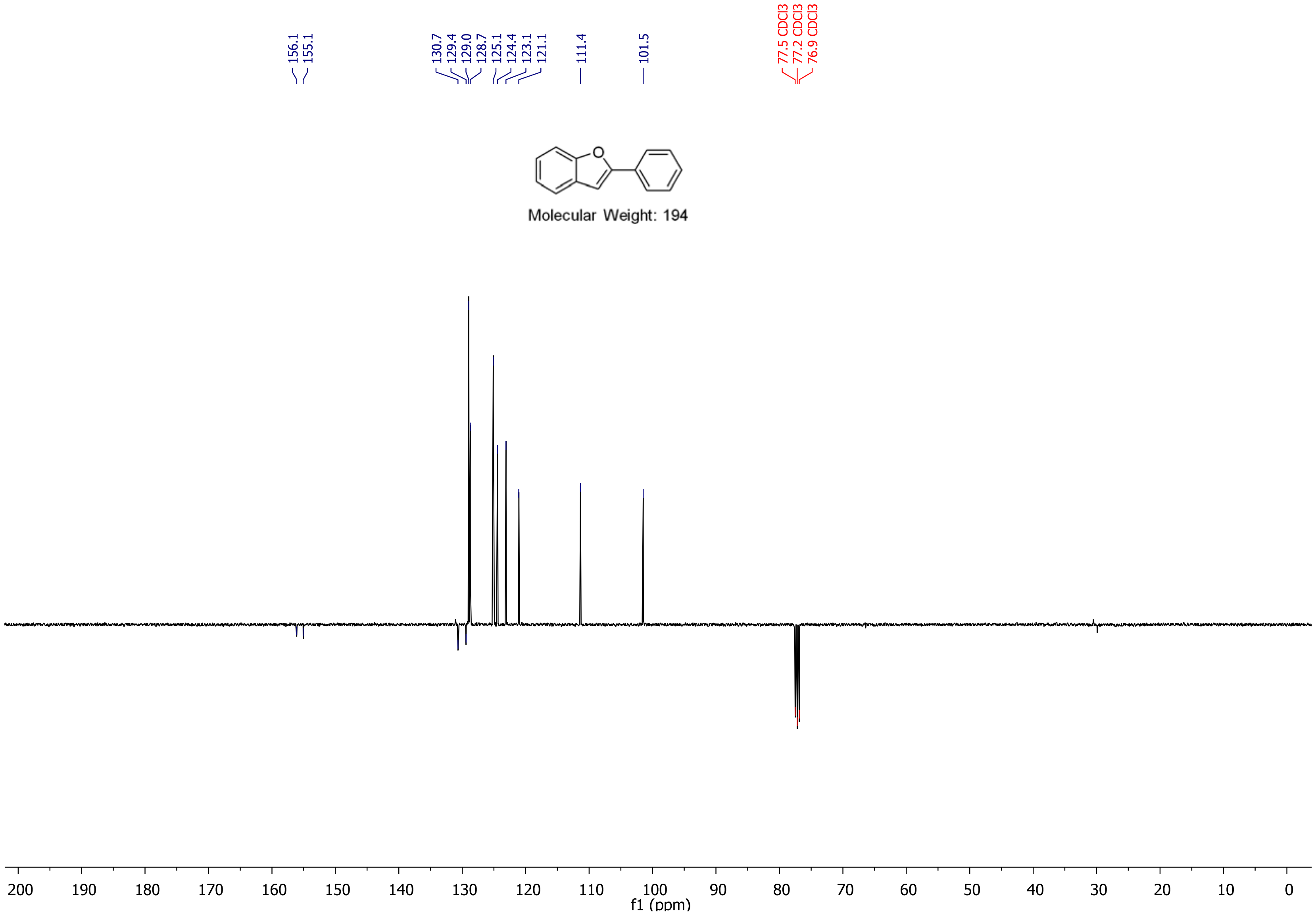
156.1
155.1
130.7
129.4
129.0
128.7
125.1
124.4
123.1
121.1
111.4
101.5
77.5 CDCl3
77.2 CDCl3
76.9 CDCl3
Molecular Weight: 194
200 190 180 170 160 150 140 130 120 110 100 90 80 70 60 50 40 30 20 10 0
f1 (ppm)

2-(3-chloro-4-methoxyphenyl)benzofuran (6b)

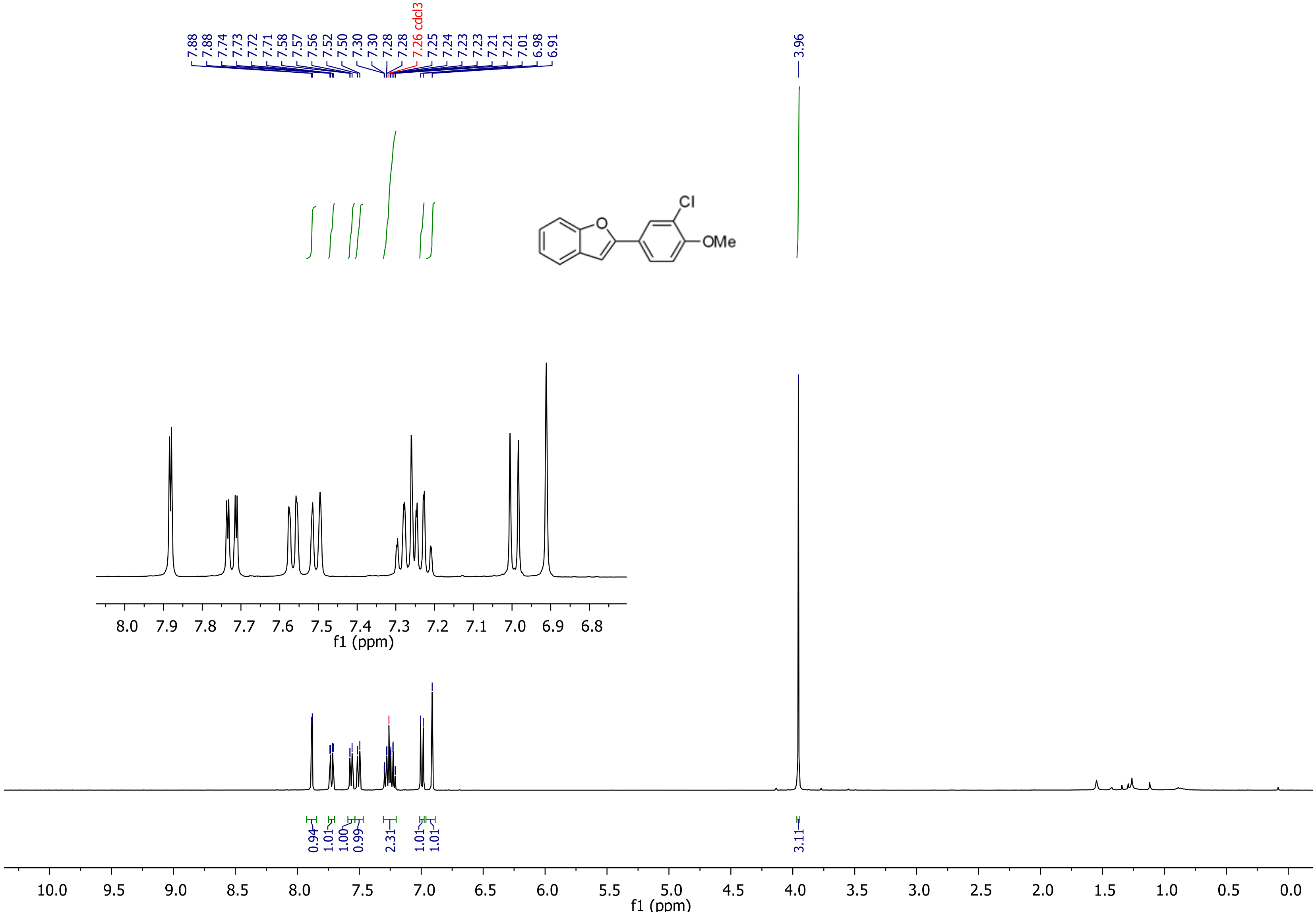

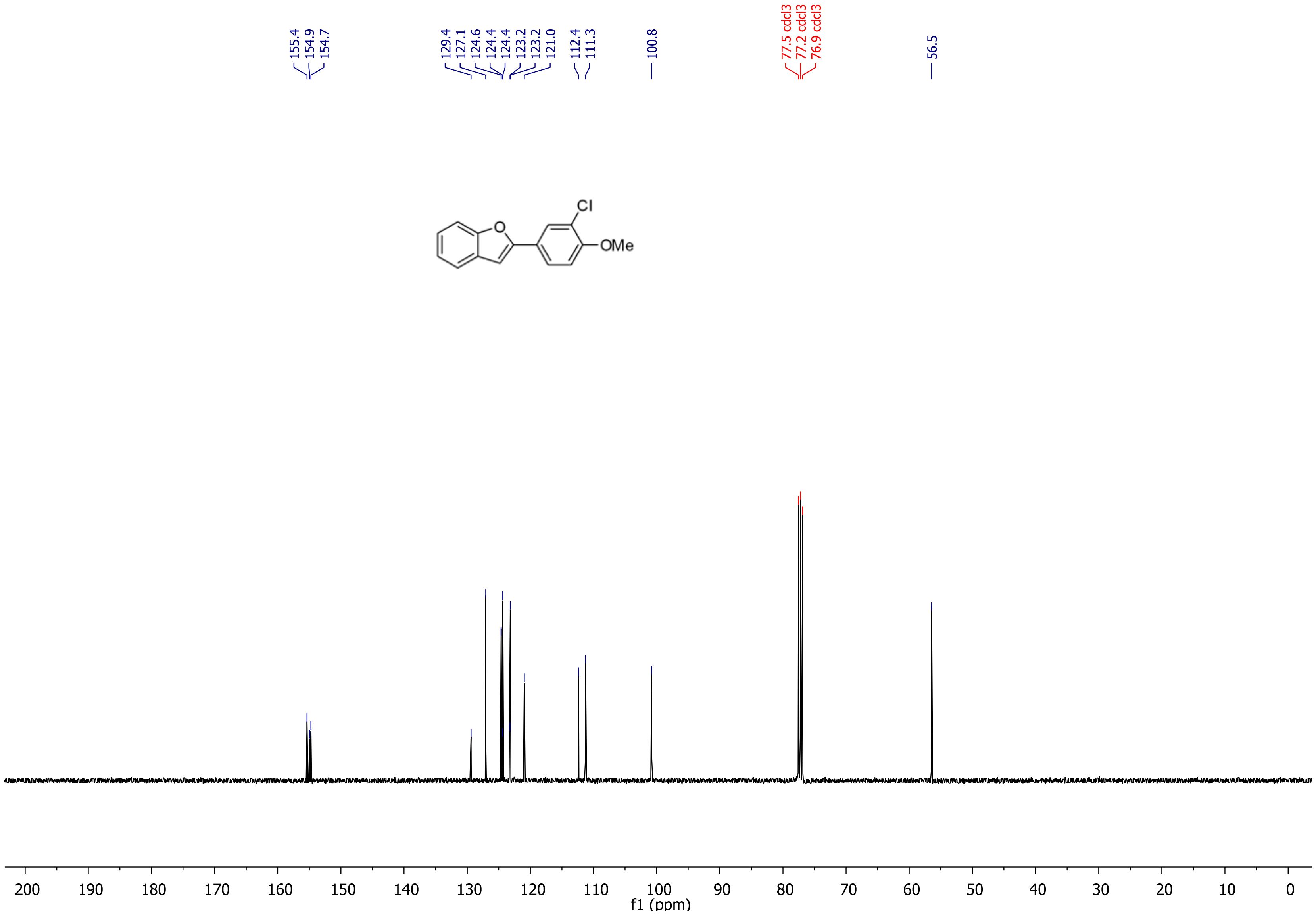
155.4
154.9
154.7
129.4
127.1
124.6
124.4
124.4
123.2
123.2
121.0
112.4
111.3
100.8
77.5 cdcl3
77.2 cdcl3
76.9 cdcl3
56.5
Cl
O
OMe
200
190
180
170
160
150
140
130
120
110
100
90
80
70
60
50
40
30
20
10
0
f1 (ppm)

2-(4-fluorophenyl)benzofuran (6c)

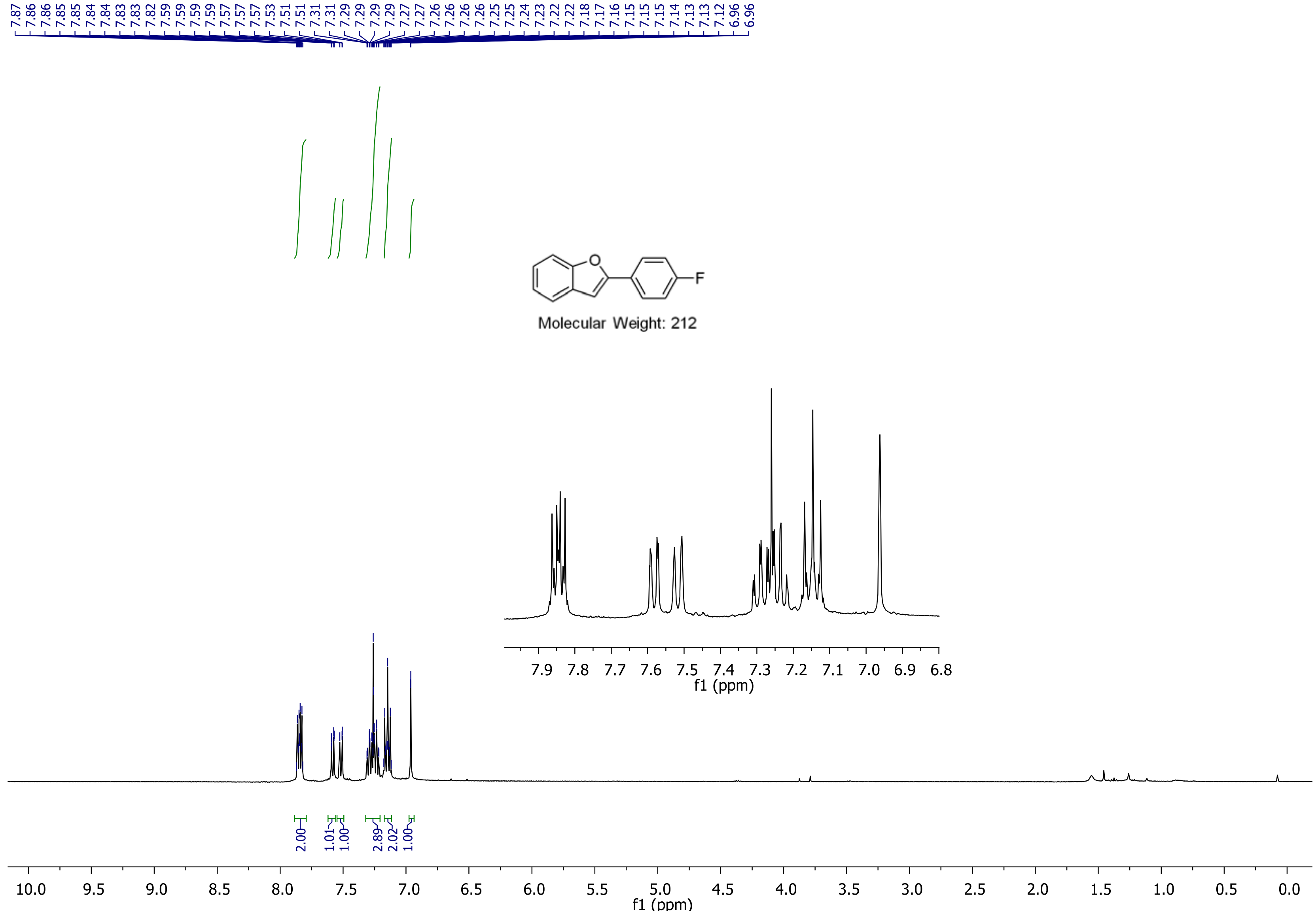

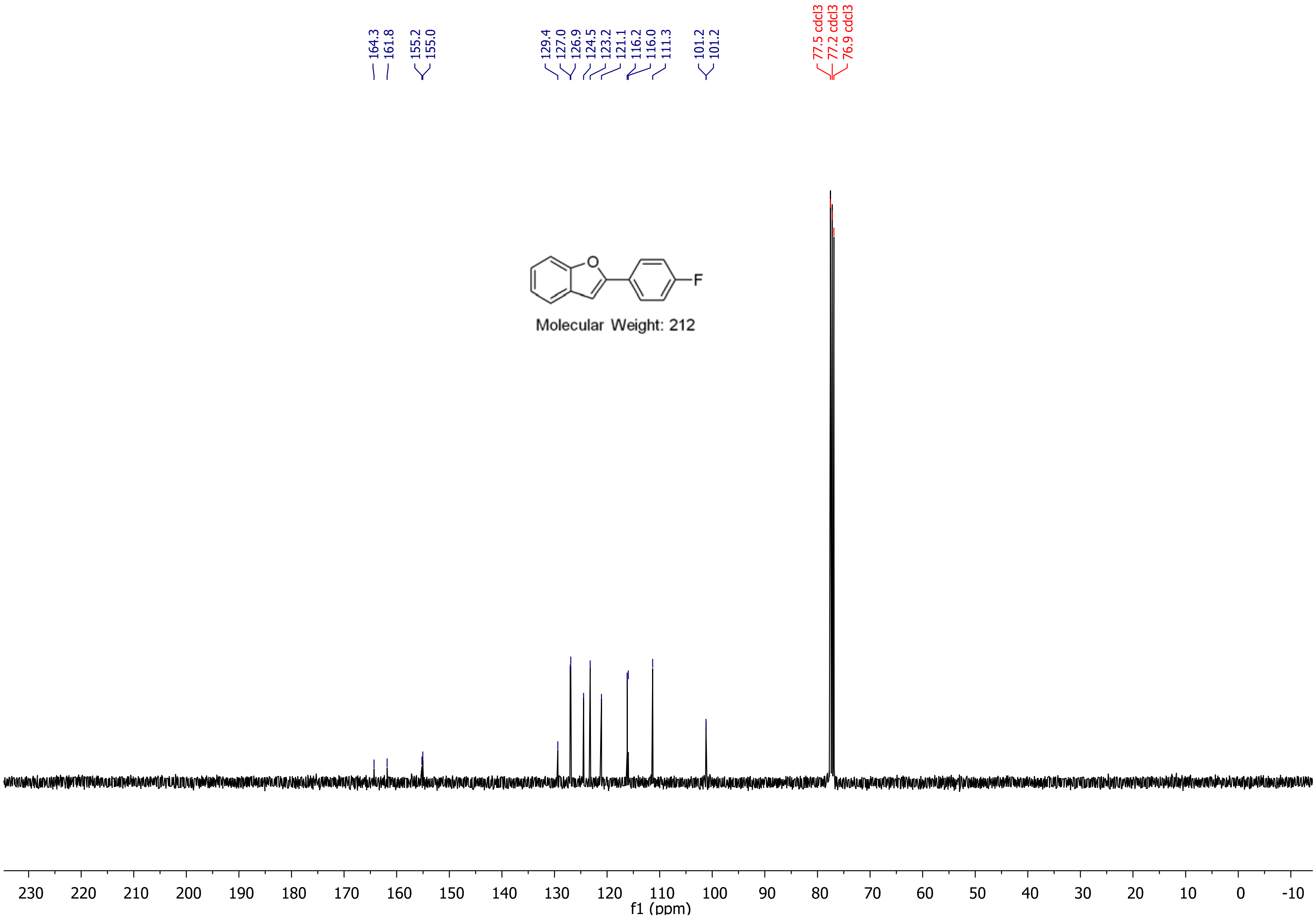

164.3
161.8
155.2
155.0
129.4
127.0
126.9
124.5
123.2
121.1
116.2
116.0
111.3
101.2
101.2
77.5 cdcl3
77.2 cdcl3
76.9 cdcl3
F
O
Molecular Weight: 212
230 220 210 200 190 180 170 160 150 140 130 120 110 100 90 80 70 60 50 40 30 20 10 0 -10
f1 (ppm)

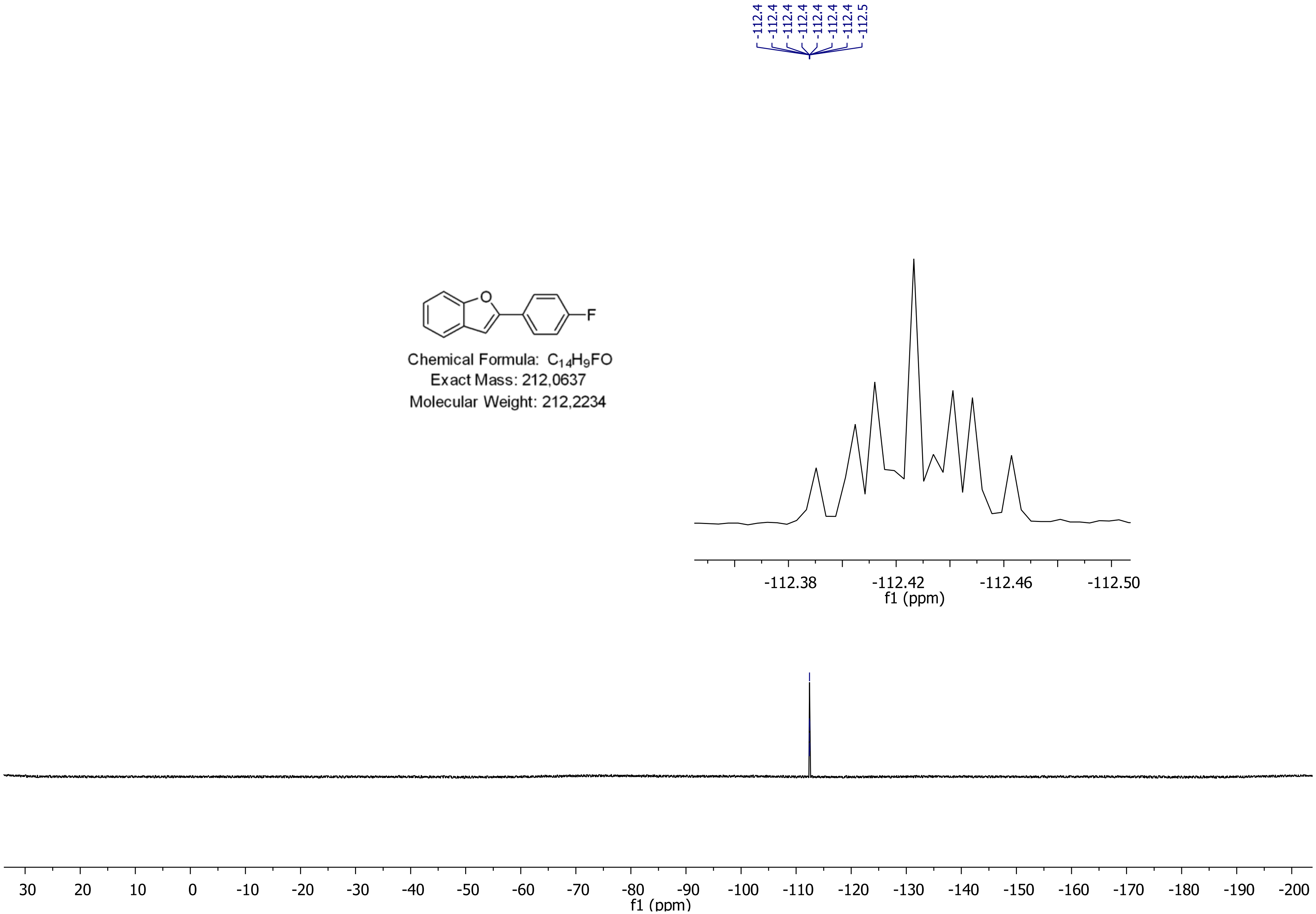

-112.4
-112.4
-112.4
-112.4
-112.4
-112.4
-112.4
-112.5
F
O
Chemical Formula: $C_{14}H_9FO$
Exact Mass: 212,0637
Molecular Weight: 212,2234
-112.38
-112.42
-112.46
-112.50
f1 (ppm)
30 20 10 0 -10 -20 -30 -40 -50 -60 -70 -80 -90 -100 -110 -120 -130 -140 -150 -160 -170 -180 -190 -200
f1 (ppm)

2-(4'-(trifluoromethyl)phenyl)benzofuran (6d)

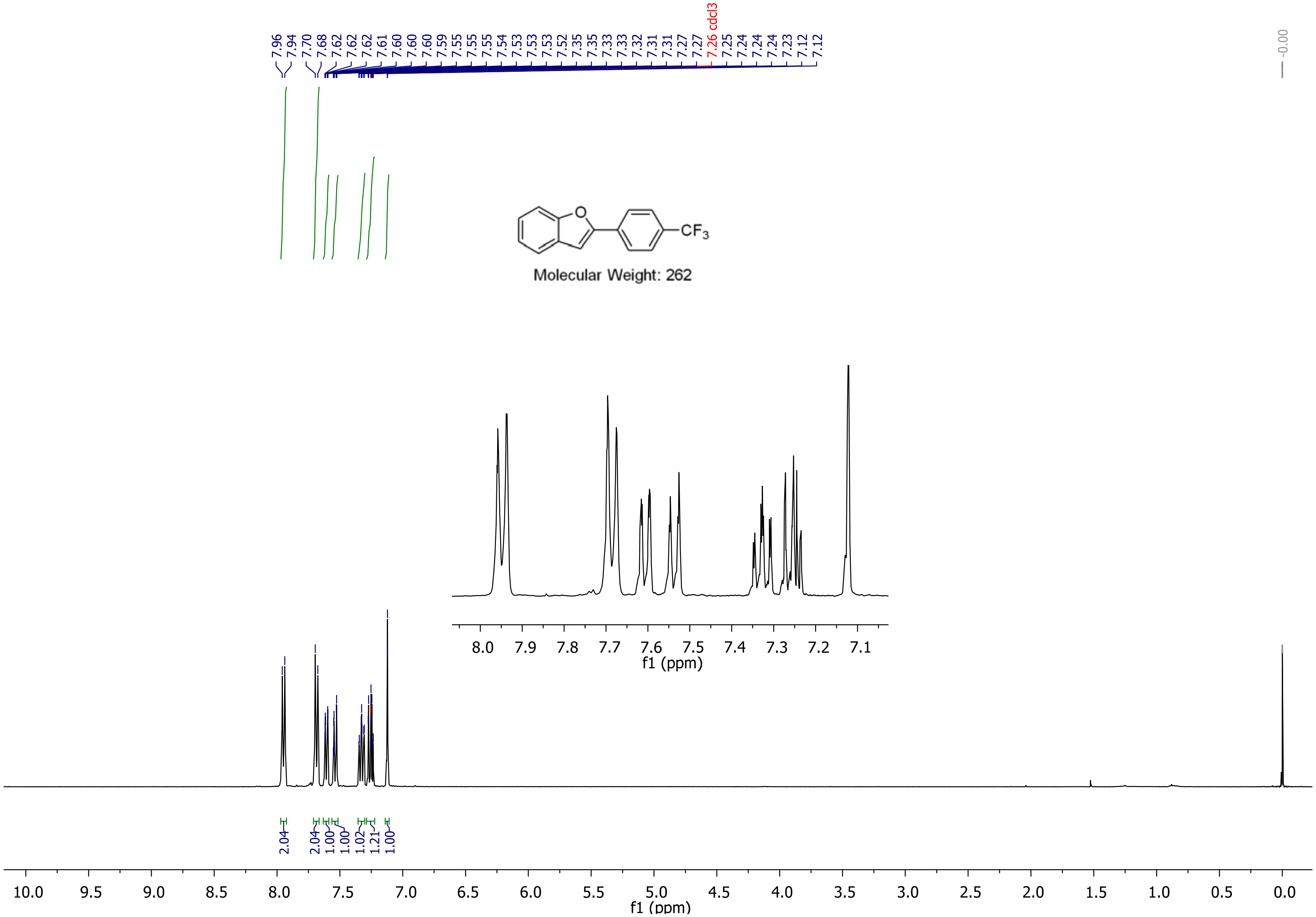

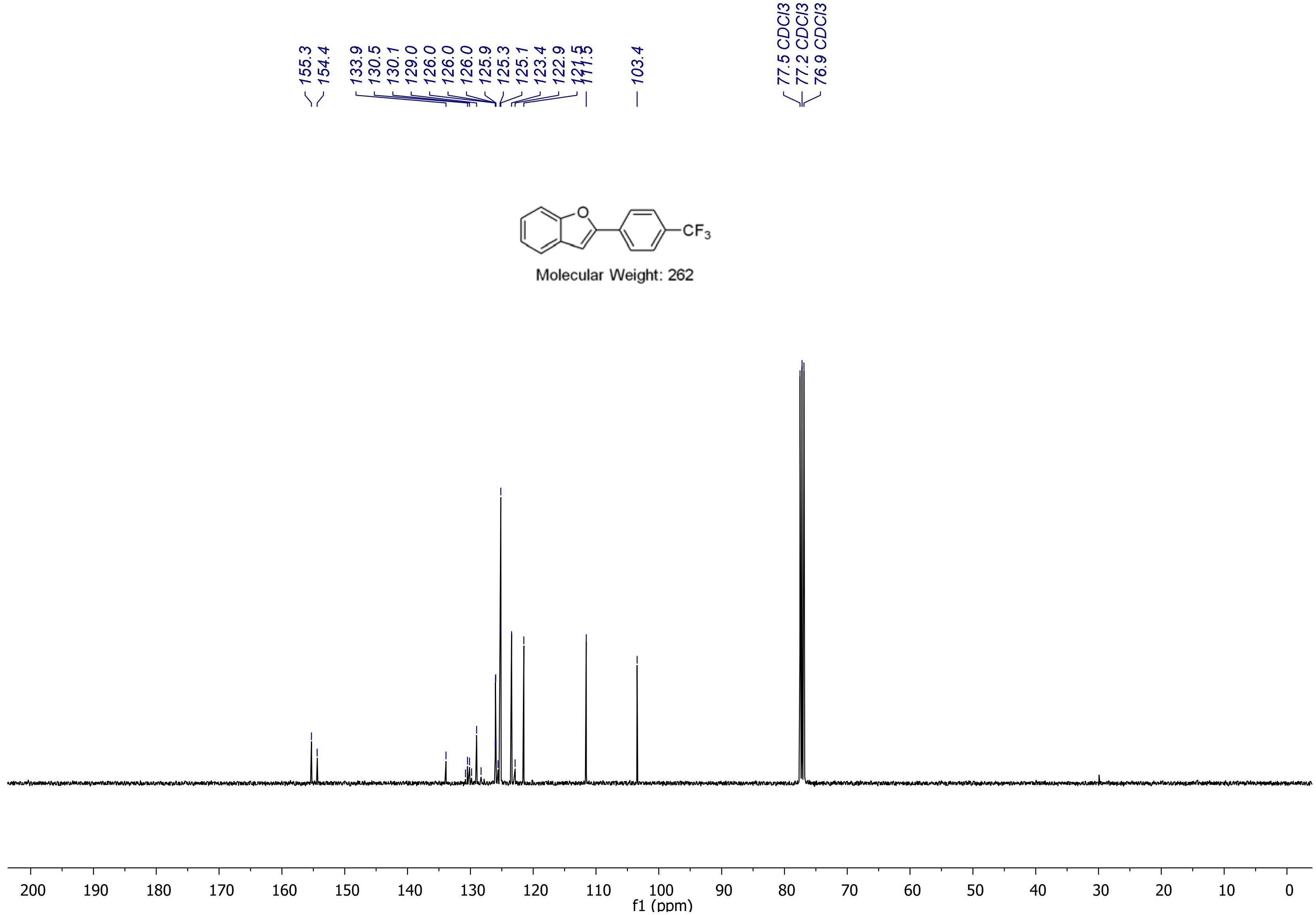
155.3
154.4
133.9
130.5
130.1
129.0
126.0
126.0
125.9
125.3
125.1
123.4
122.9
121.5
111.5
103.4
77.5 CDCl3
77.2 CDCl3
76.9 CDCl3
CF3
Molecular Weight: 262
f1 (ppm)

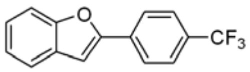

Molecular Weight: 262

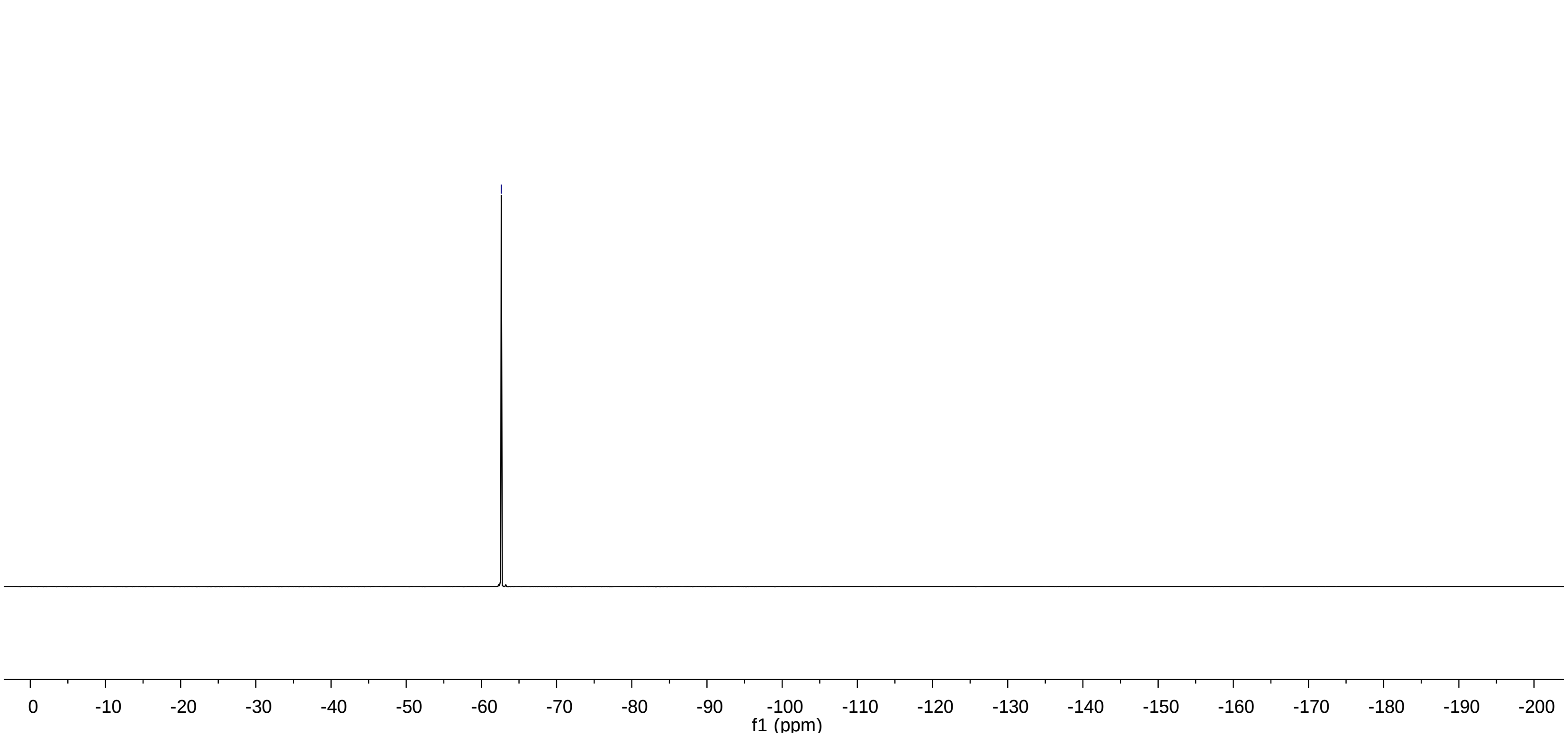

2-(3-(trifluoromethyl)phenyl)benzofuran (6e)

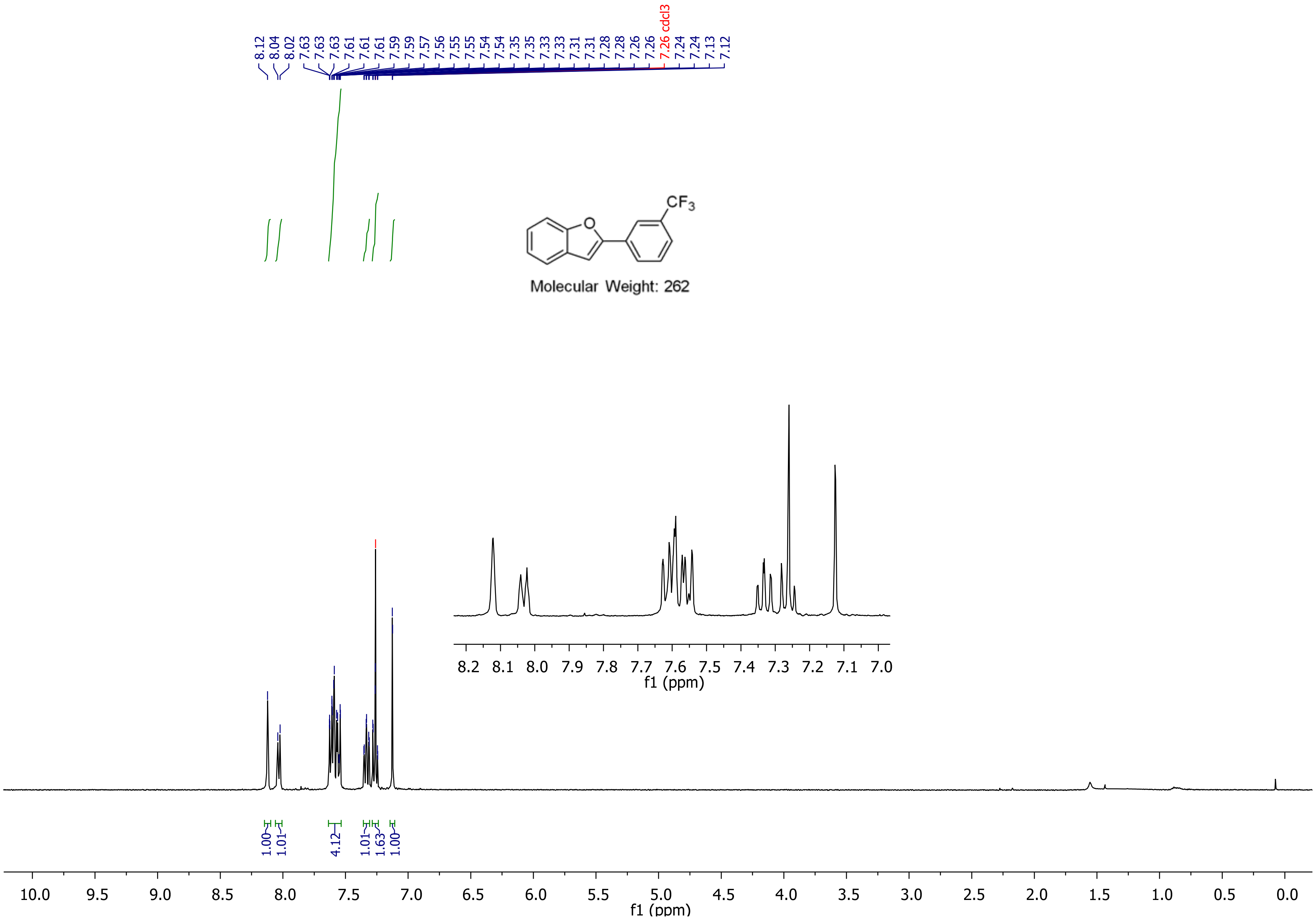

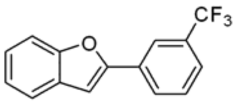

Chemical Formula: $C_{15}H_9F_3O$
Exact Mass: 262,0605
Molecular Weight: 262,2312

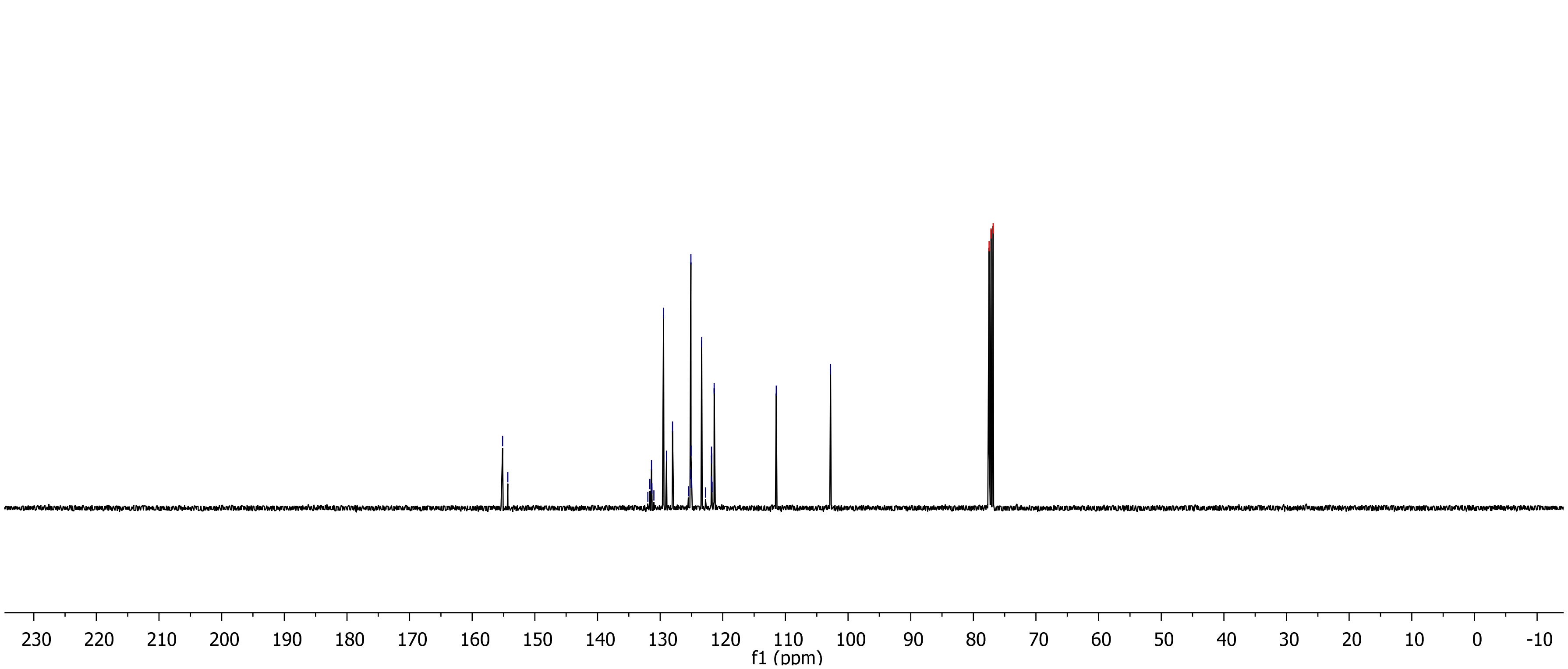

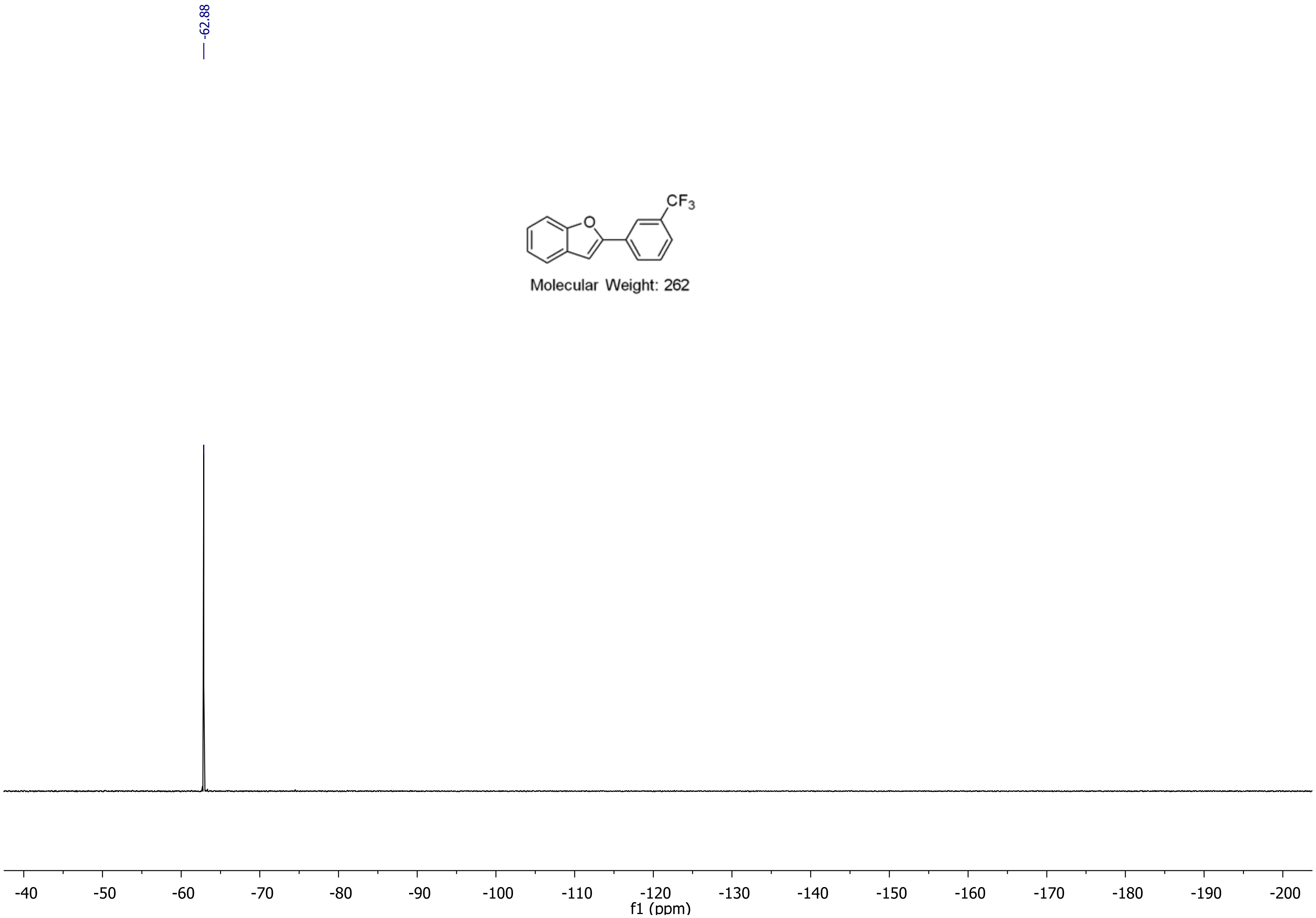
-62.88
CF_3
O
Molecular Weight: 262
-40
-50
-60
-70
-80
-90
-100
-110
-120
-130
-140
-150
-160
-170
-180
-190
-200
f1 (ppm)

2-(4-(trifluoromethoxyphenyl) benzofuran (6f)

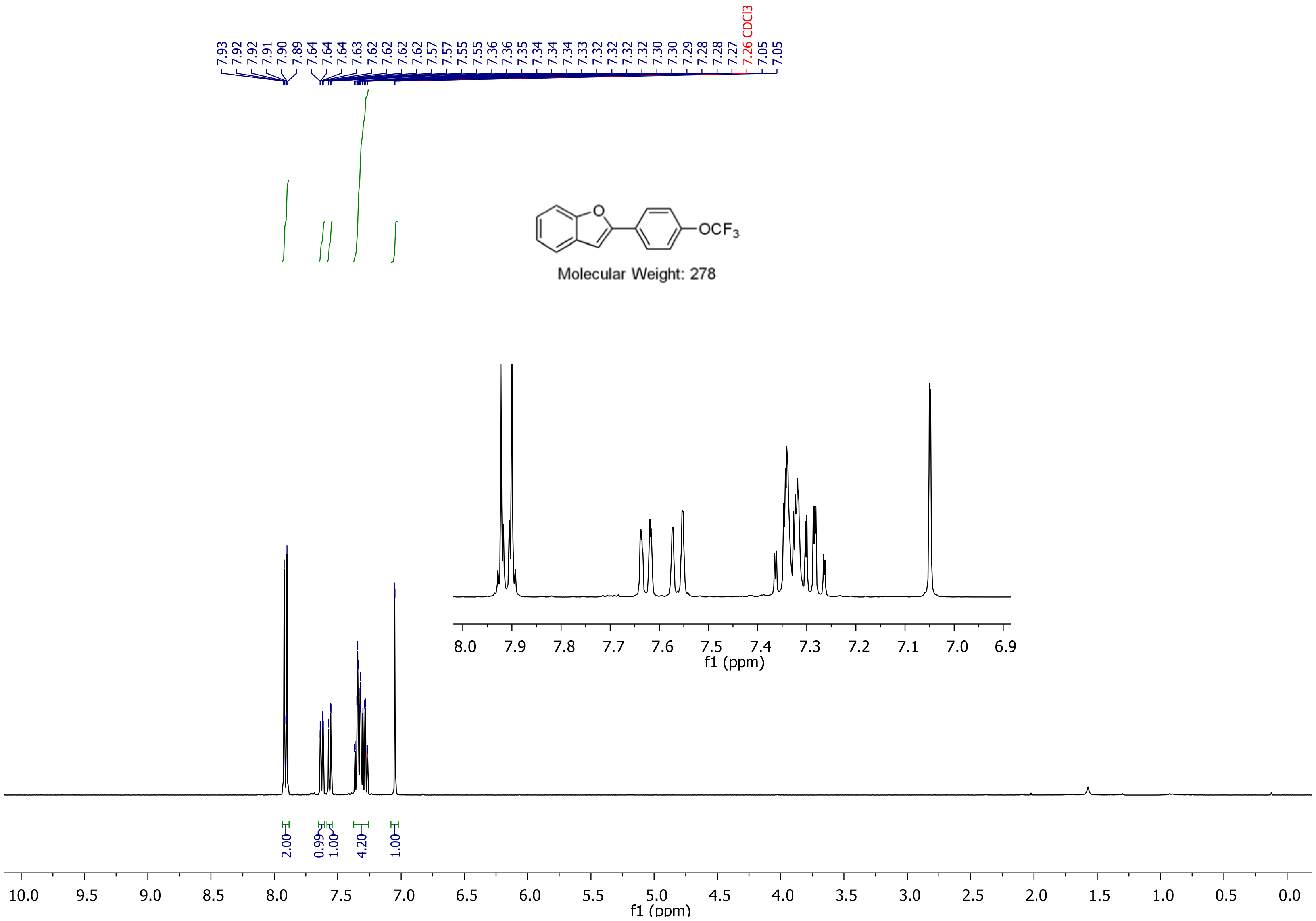

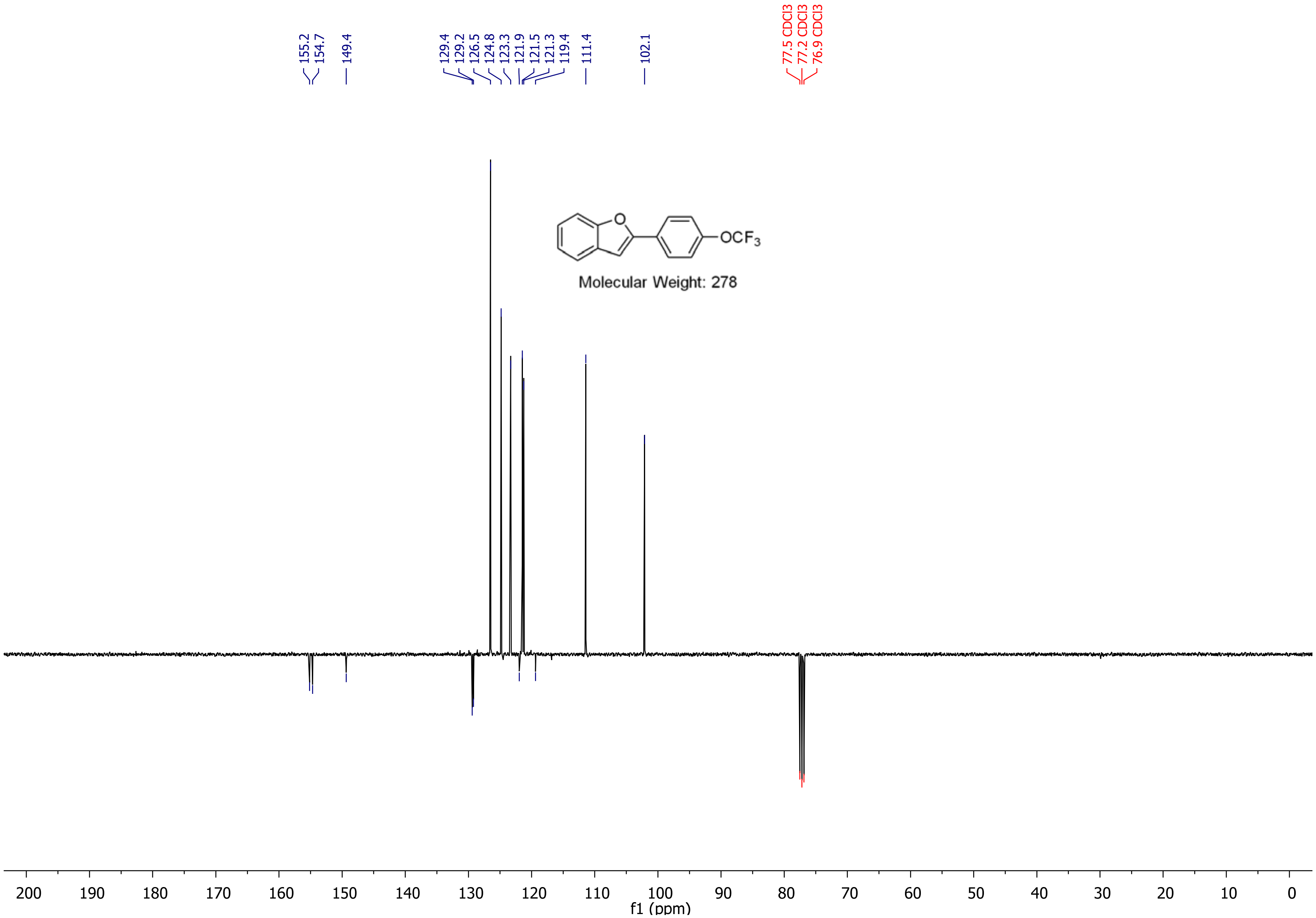
155.2
154.7
149.4
129.4
129.2
126.5
124.8
123.3
121.9
121.5
121.3
119.4
111.4
102.1
77.5 CDCl3
77.2 CDCl3
76.9 CDCl3
OCF3
Molecular Weight: 278
200 190 180 170 160 150 140 130 120 110 100 90 80 70 60 50 40 30 20 10 0
f1 (ppm)

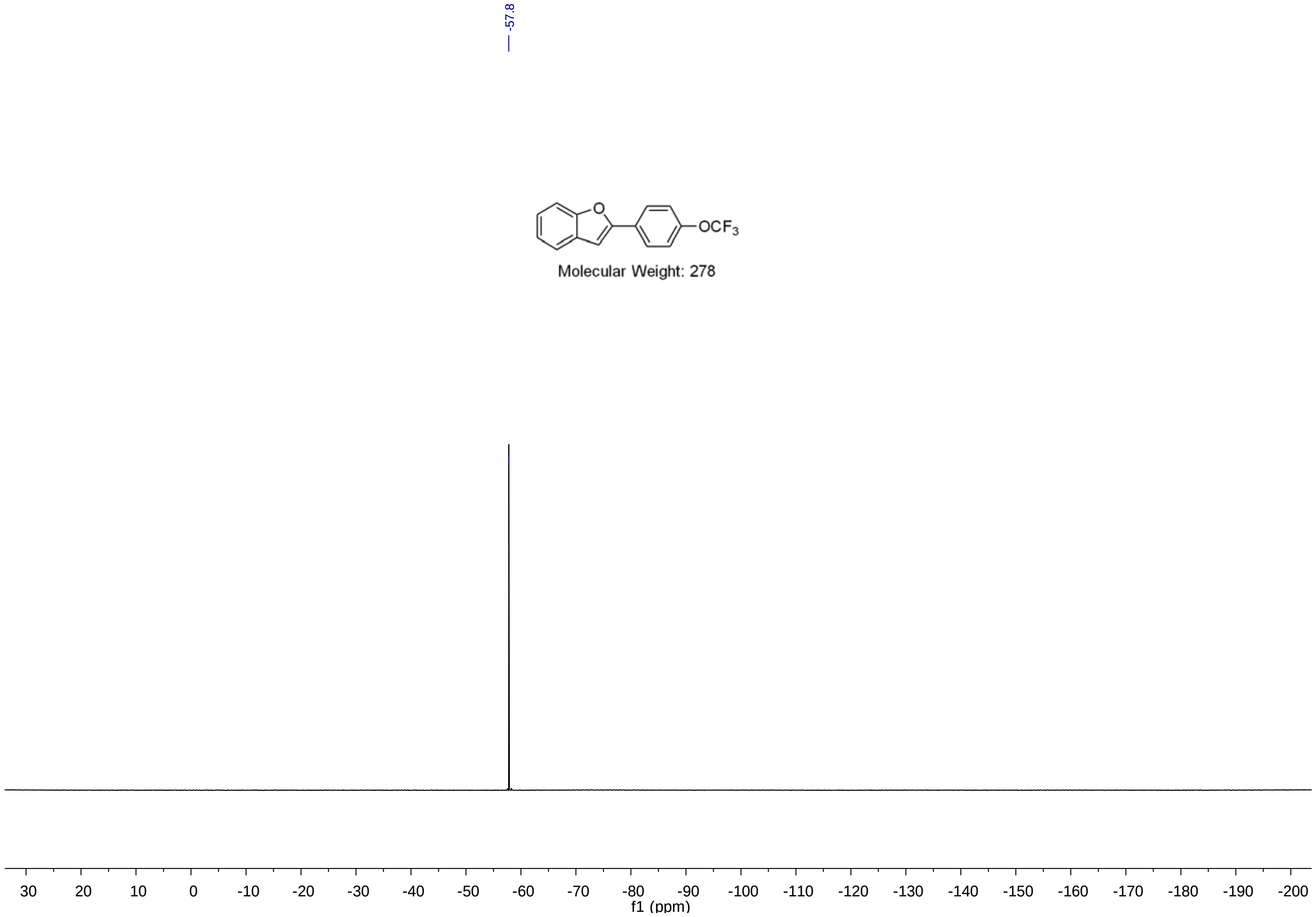
-57.8
OCF3
Molecular Weight: 278
30 20 10 0 -10 -20 -30 -40 -50 -60 -70 -80 -90 -100 -110 -120 -130 -140 -150 -160 -170 -180 -190 -200
f1 (ppm)

2-(2,3-dichlorophenyl)benzofuran (6g)

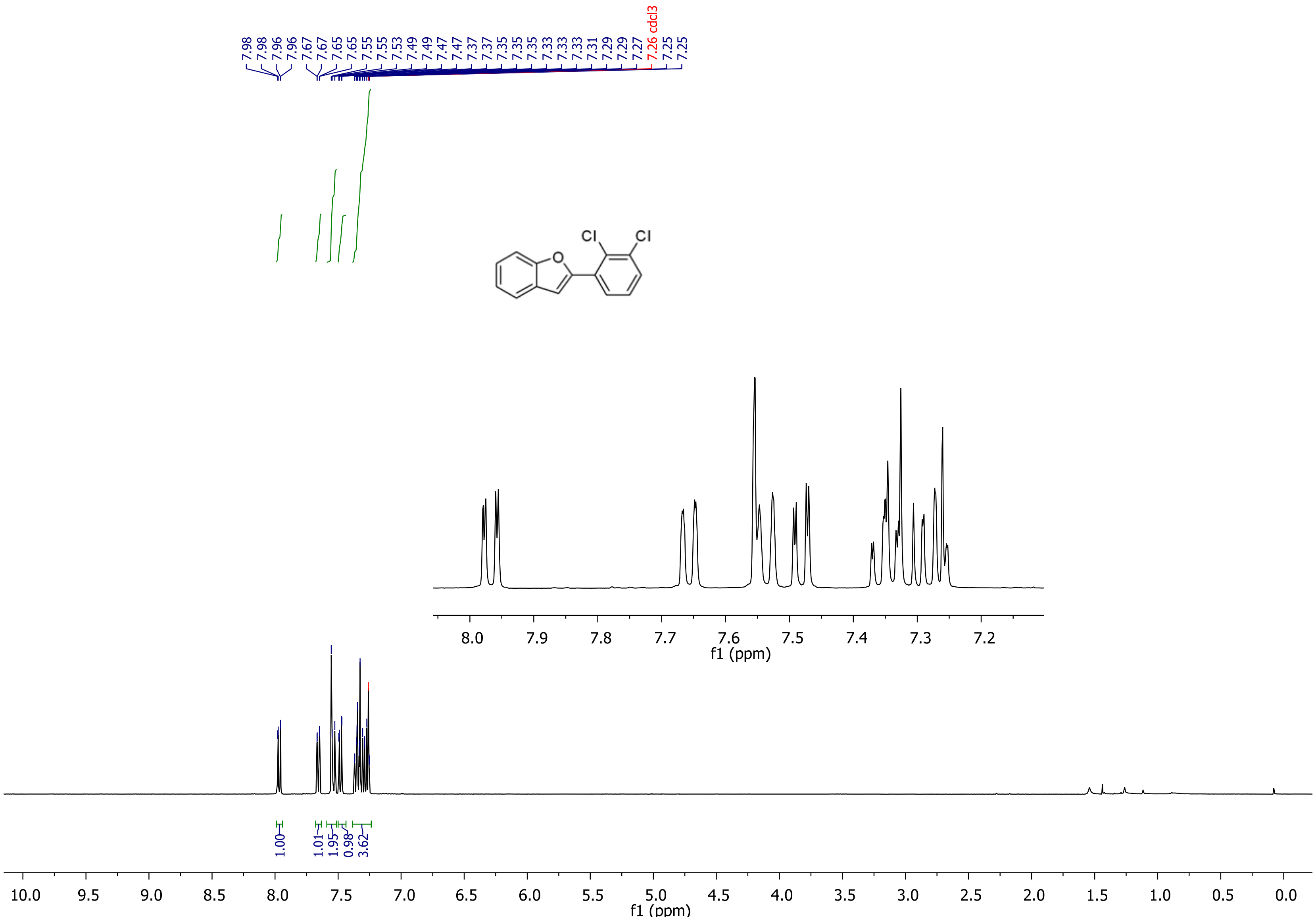

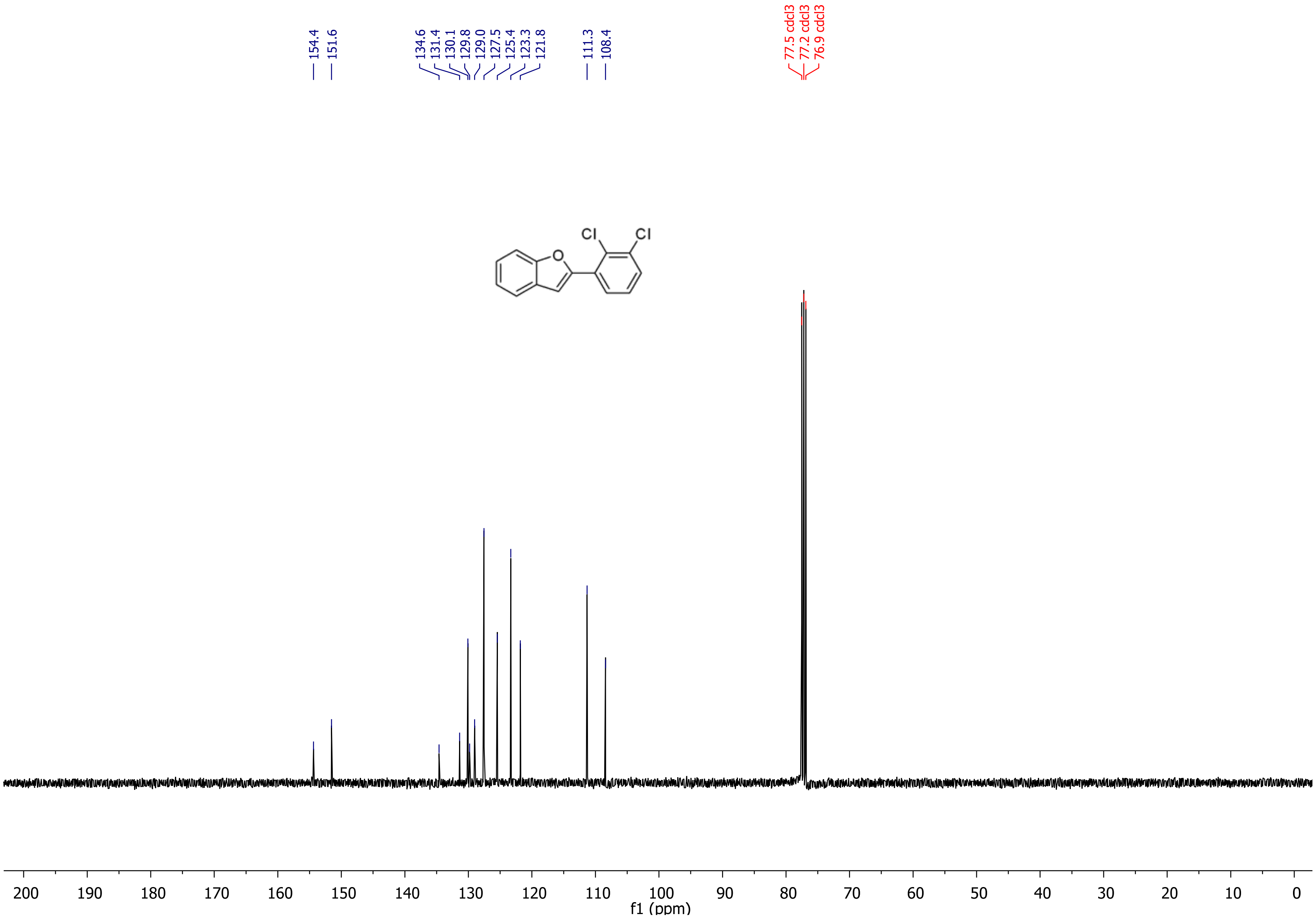
154.4
151.6
134.6
131.4
130.1
129.8
129.0
127.5
125.4
123.3
121.8
111.3
108.4
77.5 cdcl3
77.2 cdcl3
76.9 cdcl3
Cl
Cl
O
200 190 180 170 160 150 140 130 120 110 100 90 80 70 60 50 40 30 20 10 0
f1 (ppm)

2-(3,5-dichlorophenyl)benzofuran (6h)

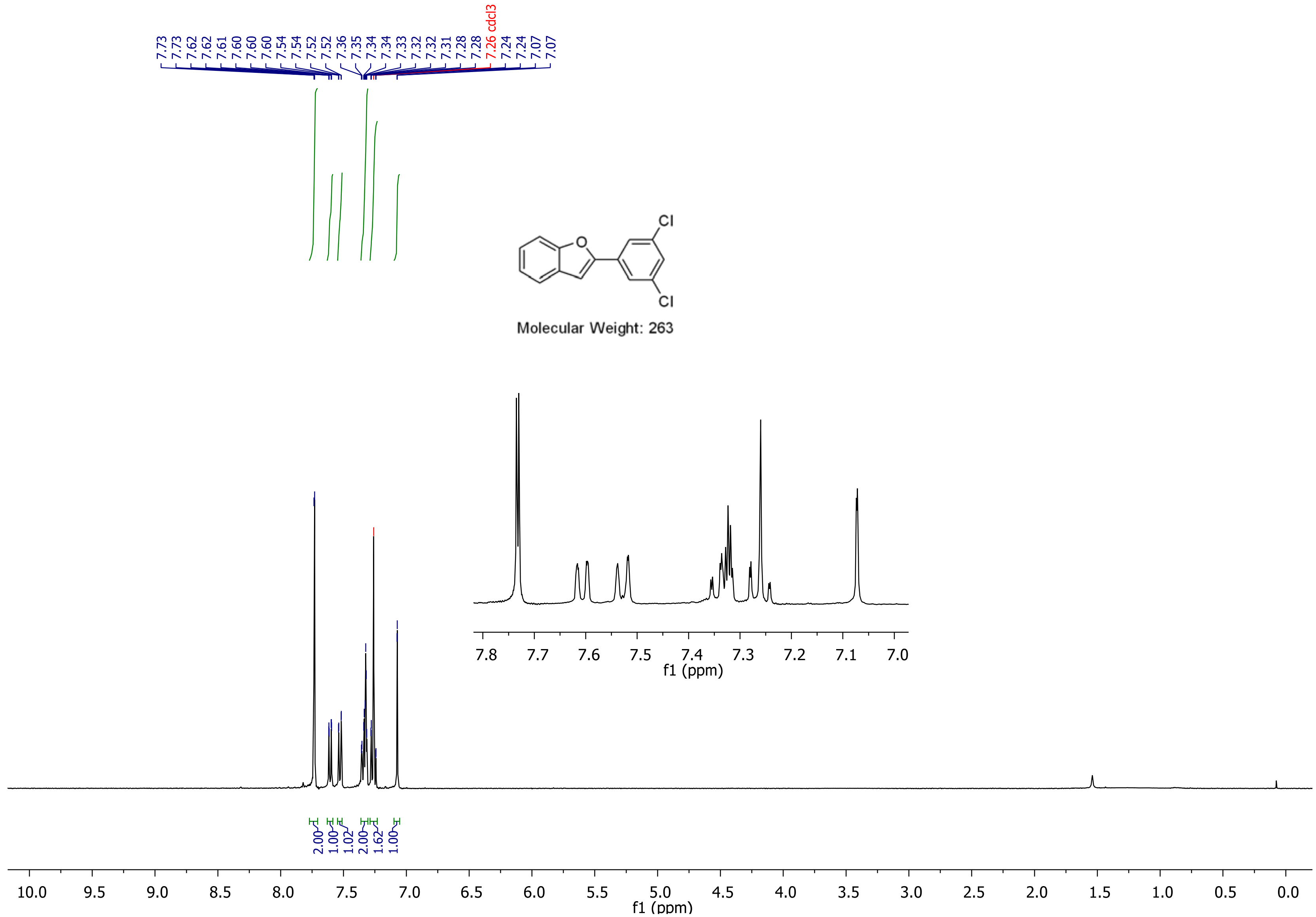

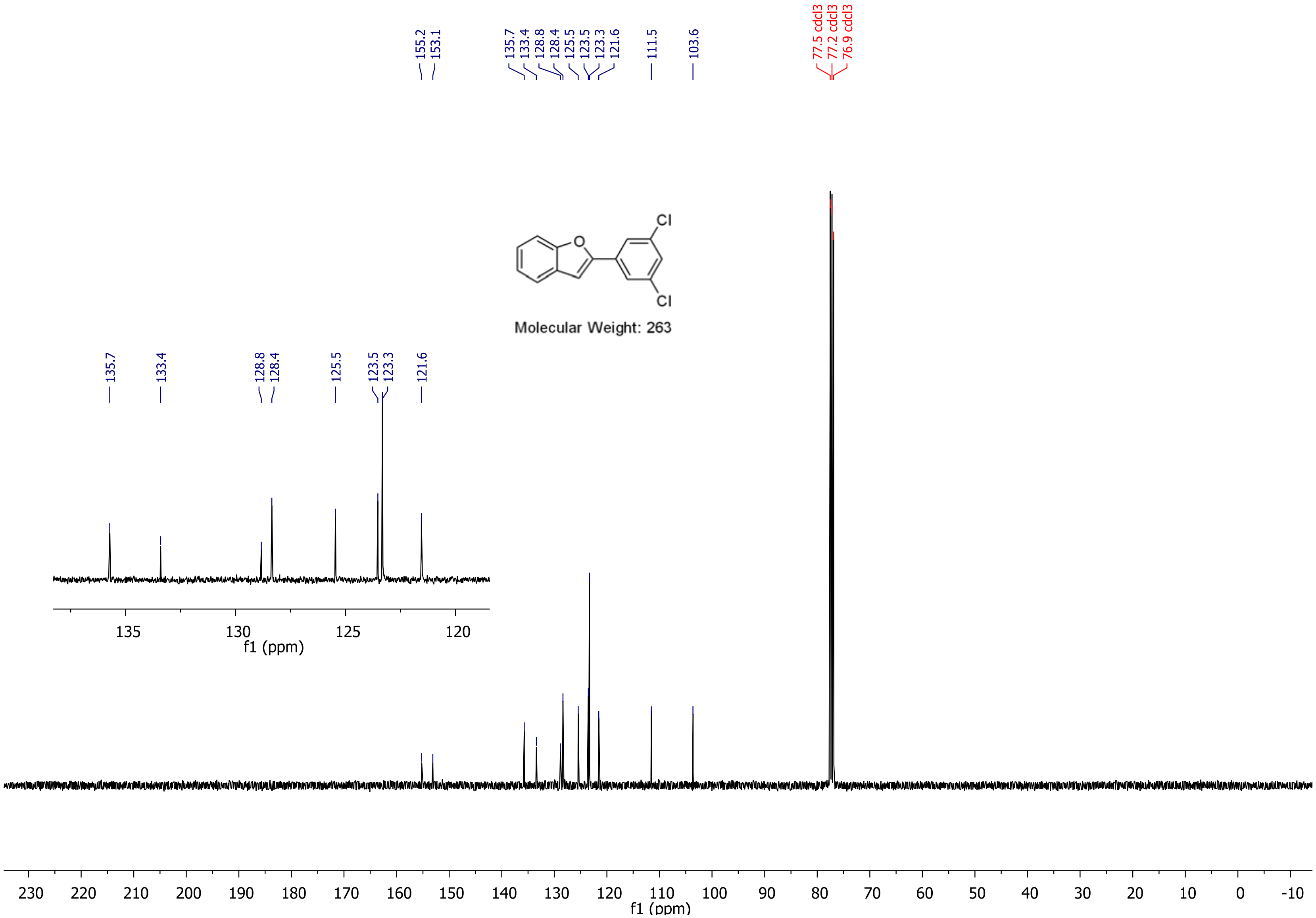
155.2
153.1
135.7
133.4
128.8
128.4
125.5
123.5
123.3
121.6
111.5
103.6
77.5 cdcl3
77.2 cdcl3
76.9 cdcl3
Cl
O
Cl
Molecular Weight: 263
135
130
125
120
f1 (ppm)
230 220 210 200 190 180 170 160 150 140 130 120 110 100 90 80 70 60 50 40 30 20 10 0 -10
f1 (ppm)

3-(3-(trifluoromethyl)phenyl)benzo[*b*]thiophene (7a)

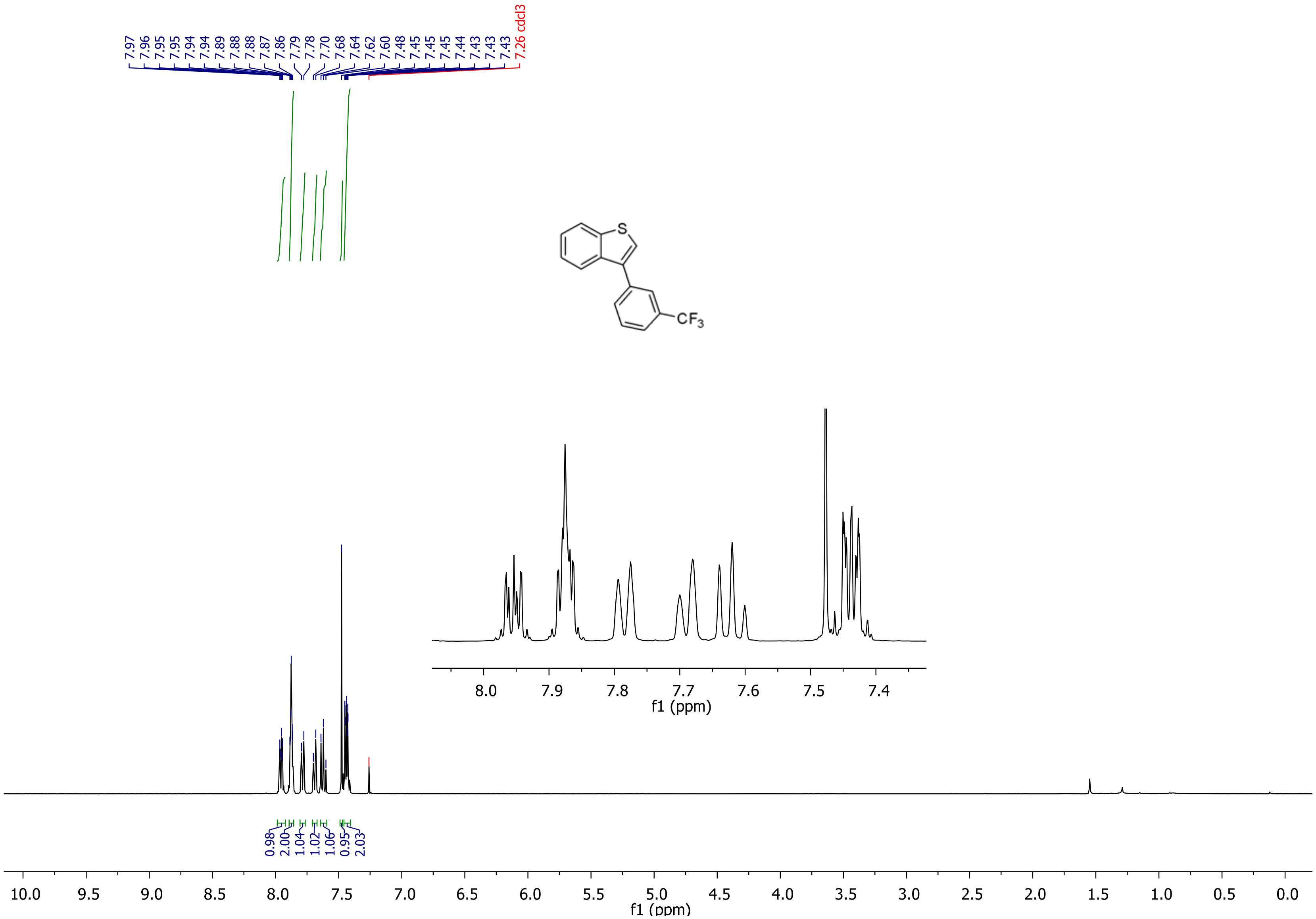

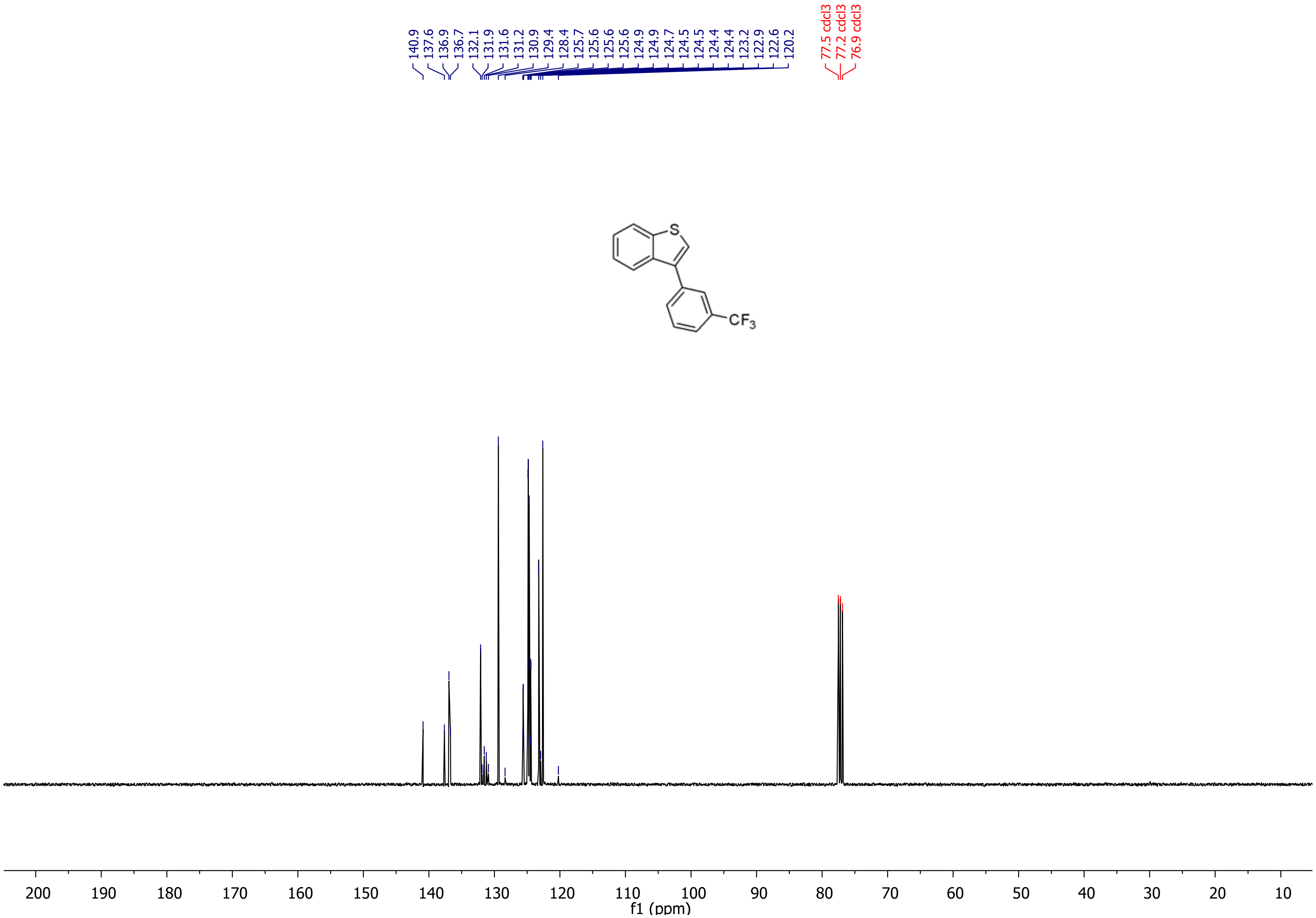

140.9
137.6
136.9
136.7
132.1
131.9
131.6
131.2
130.9
129.4
128.4
125.7
125.6
125.6
125.6
124.9
124.9
124.7
124.5
124.5
124.4
124.4
123.2
122.9
122.6
120.2
77.5 cdcl3
77.2 cdcl3
76.9 cdcl3
S
CF3
200 190 180 170 160 150 140 130 120 110 100 90 80 70 60 50 40 30 20 10
f1 (ppm)

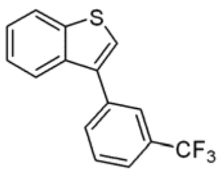

Chemical Formula: $C_{15}H_9F_3S$
Exact Mass: 278,0377
Molecular Weight: 278,2922

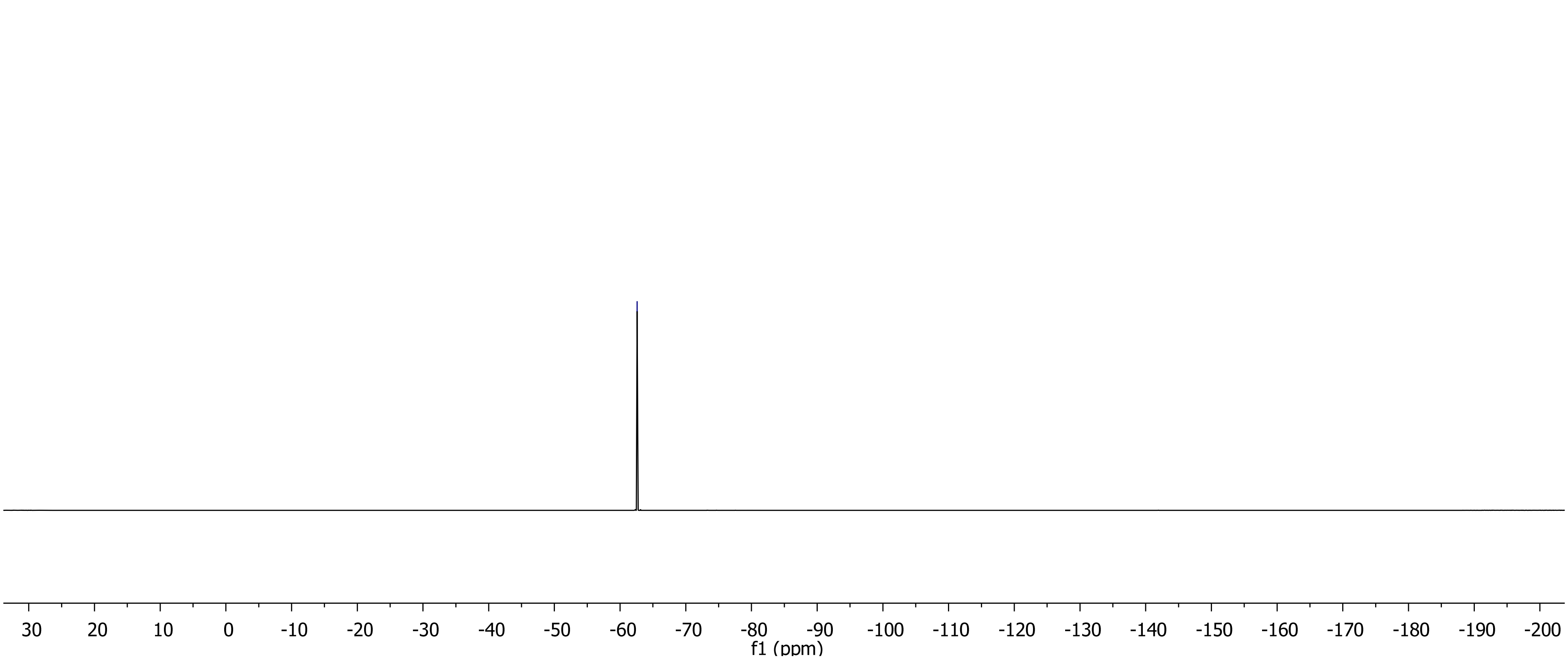

3-(4-(trifluoromethoxy)phenyl)benzo[*b*]thiophene (7b)

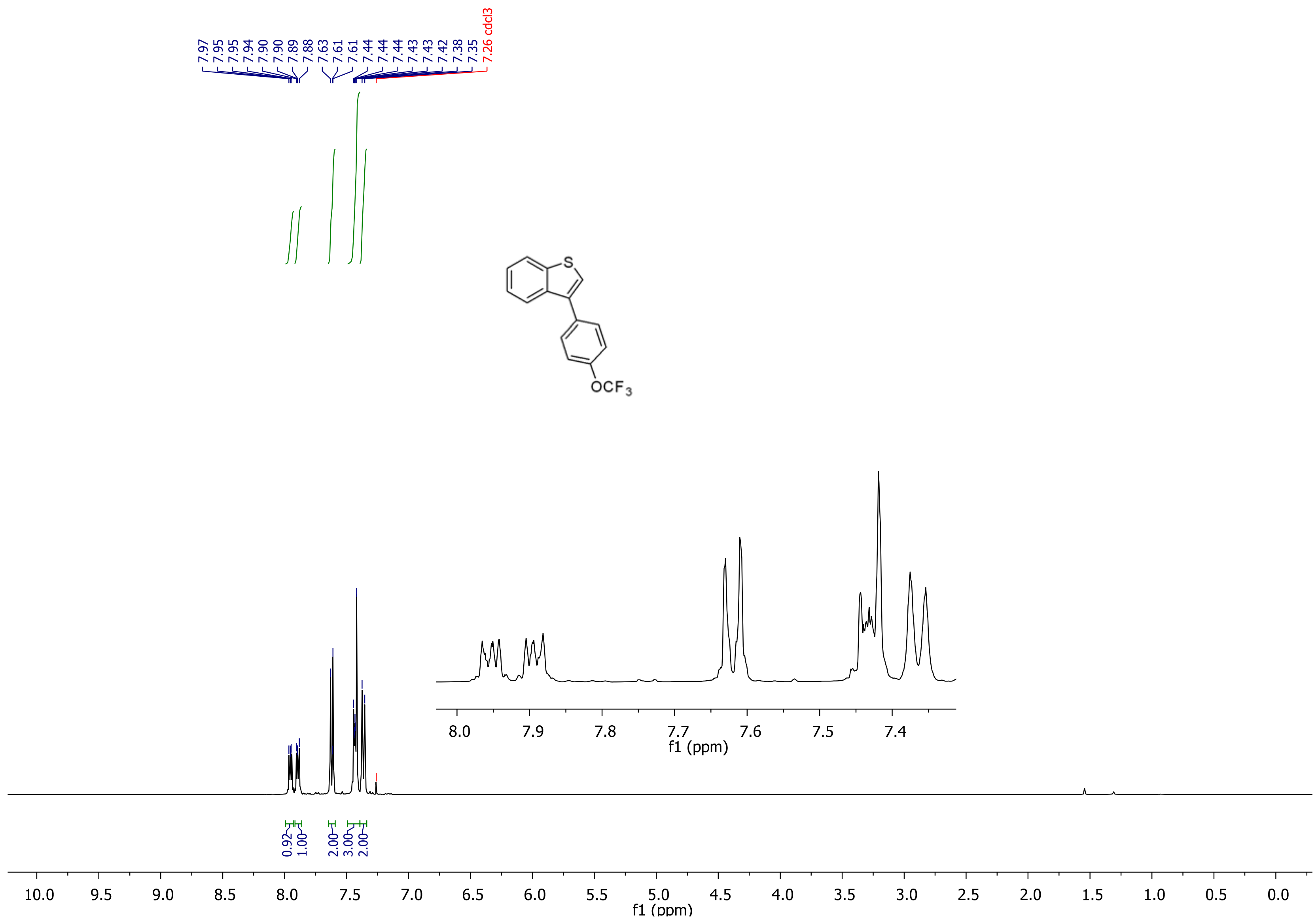

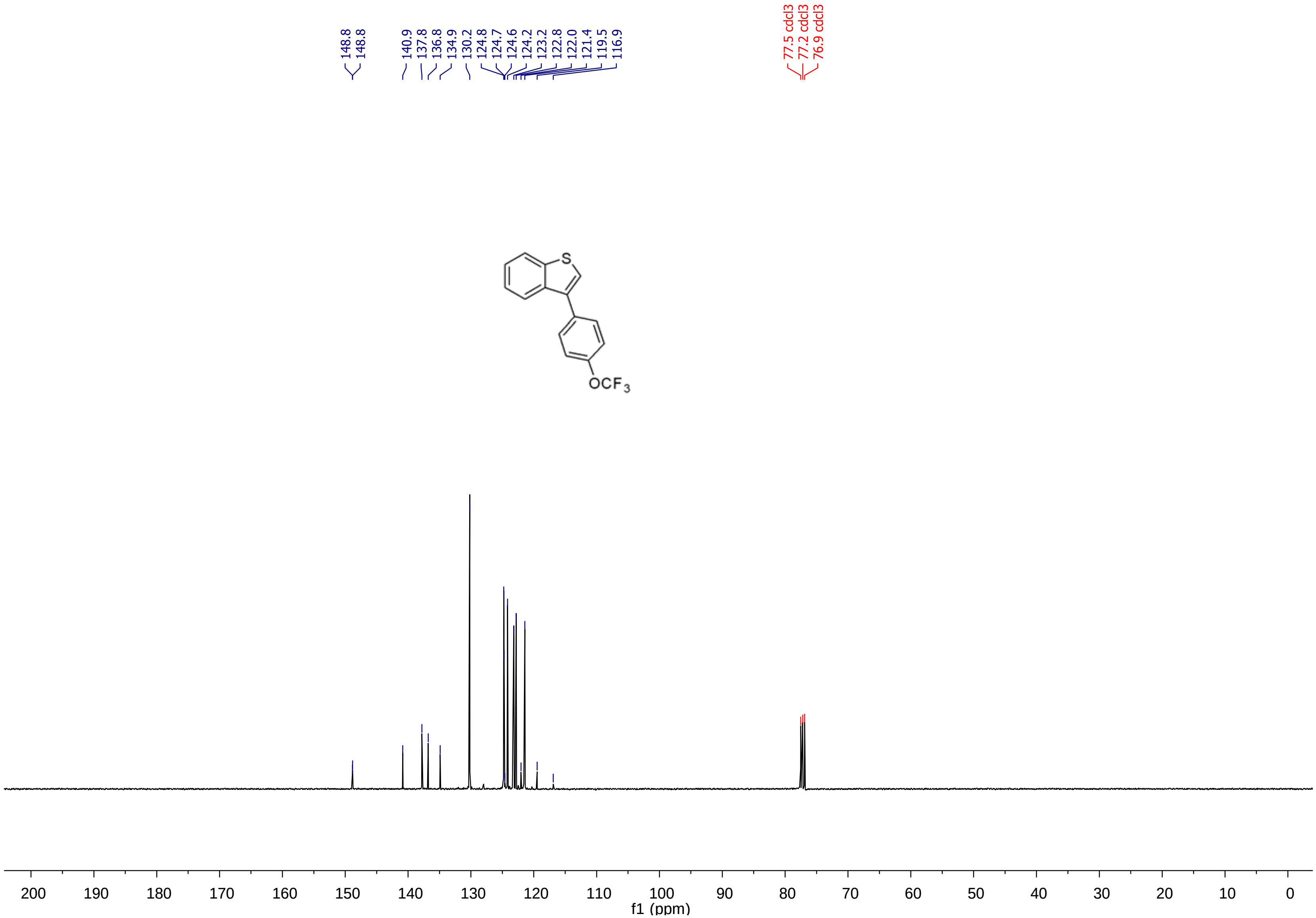

148.8
148.8
140.9
137.8
136.8
134.9
130.2
124.8
124.7
124.6
124.2
123.2
122.8
122.0
121.4
119.5
116.9
77.5 cdcl3
77.2 cdcl3
76.9 cdcl3
S
OCF3
200
190
180
170
160
150
140
130
120
110
100
90
80
70
60
50
40
30
20
10
0
f1 (ppm)

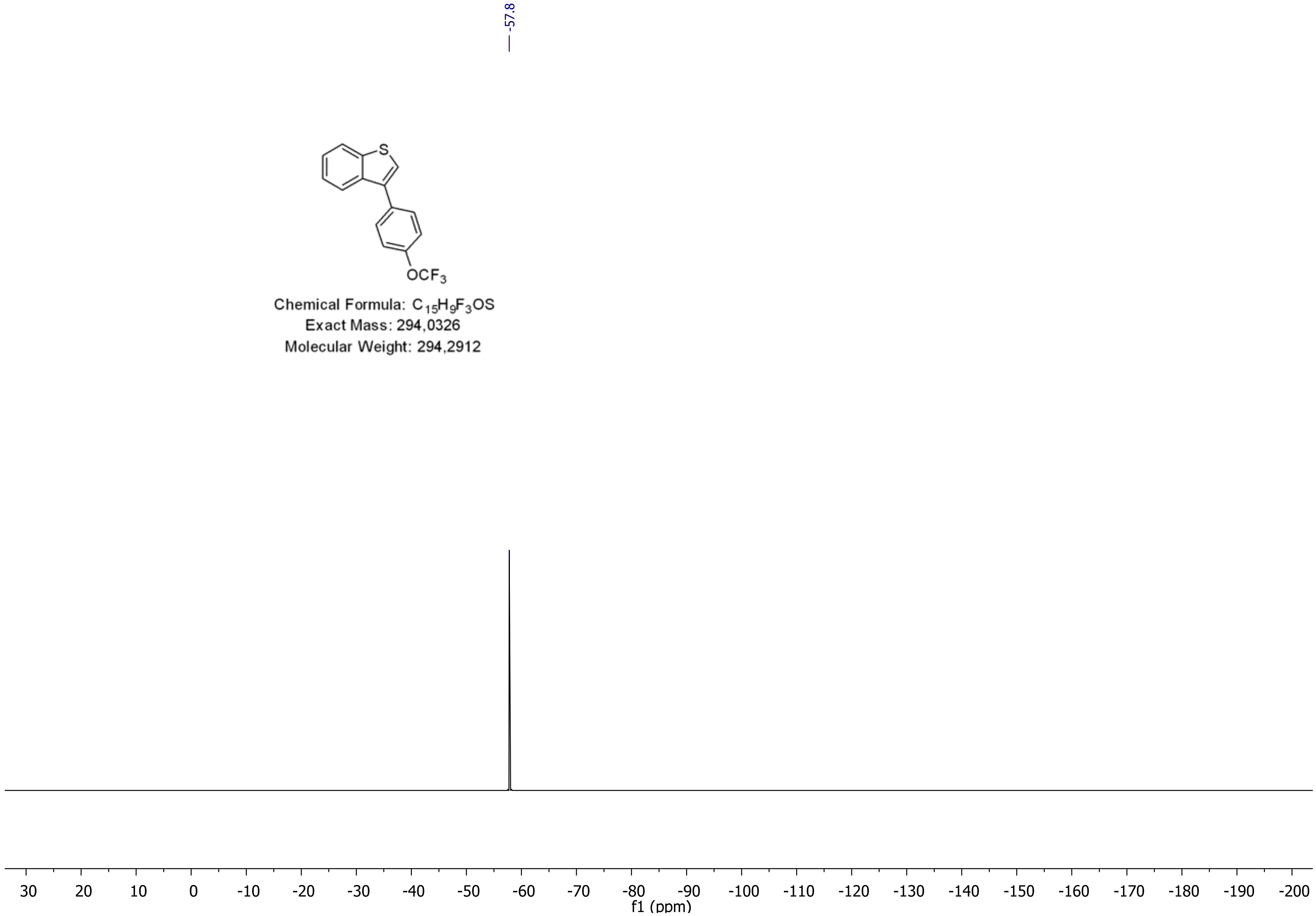
-57.8
OCF3
Chemical Formula: C15H9F3OS
Exact Mass: 294,0326
Molecular Weight: 294,2912
30 20 10 0 -10 -20 -30 -40 -50 -60 -70 -80 -90 -100 -110 -120 -130 -140 -150 -160 -170 -180 -190 -200
f1 (ppm)

methyl 2-(5-methylbenzofuran-2-yl)benzoate (8a)

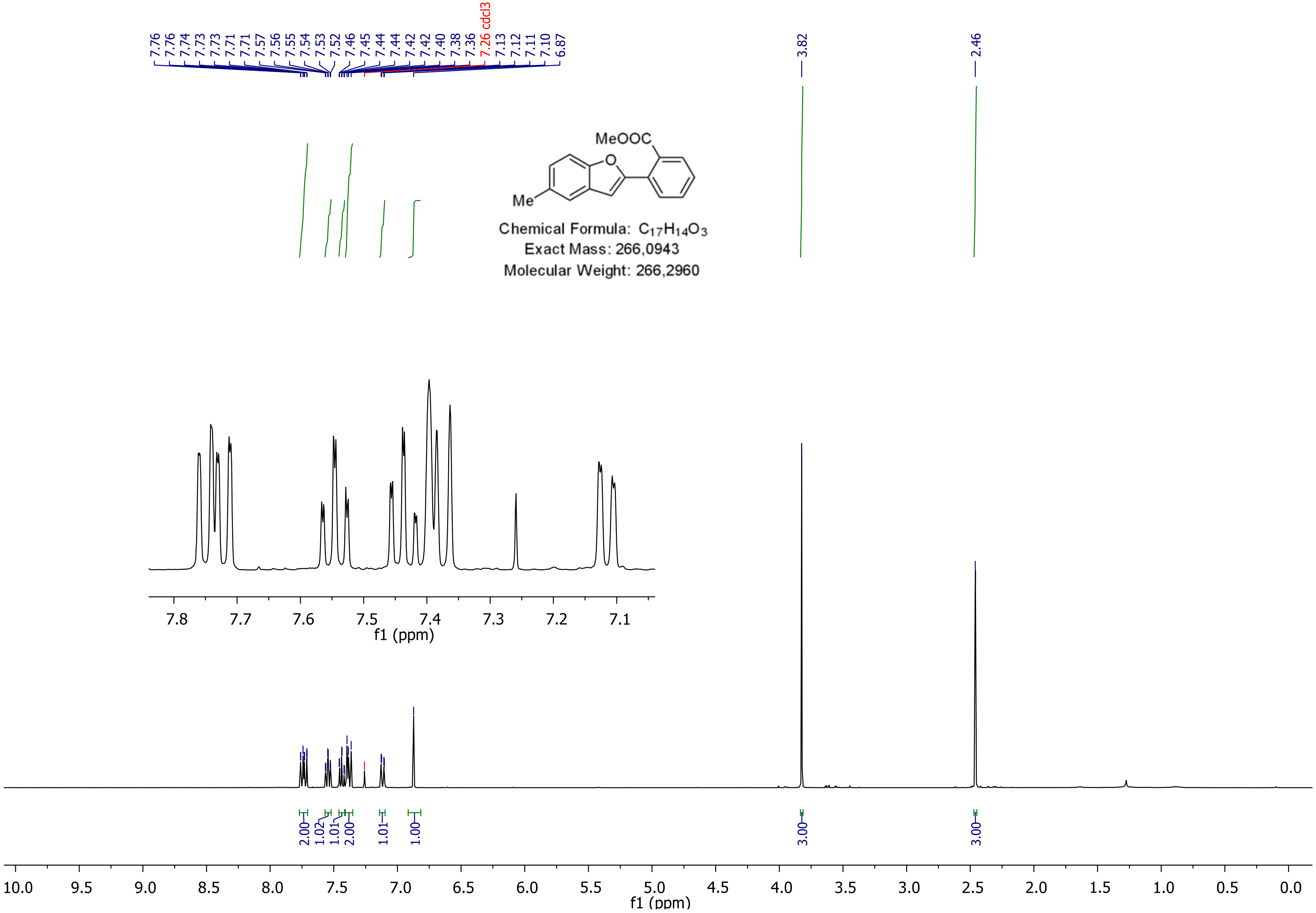

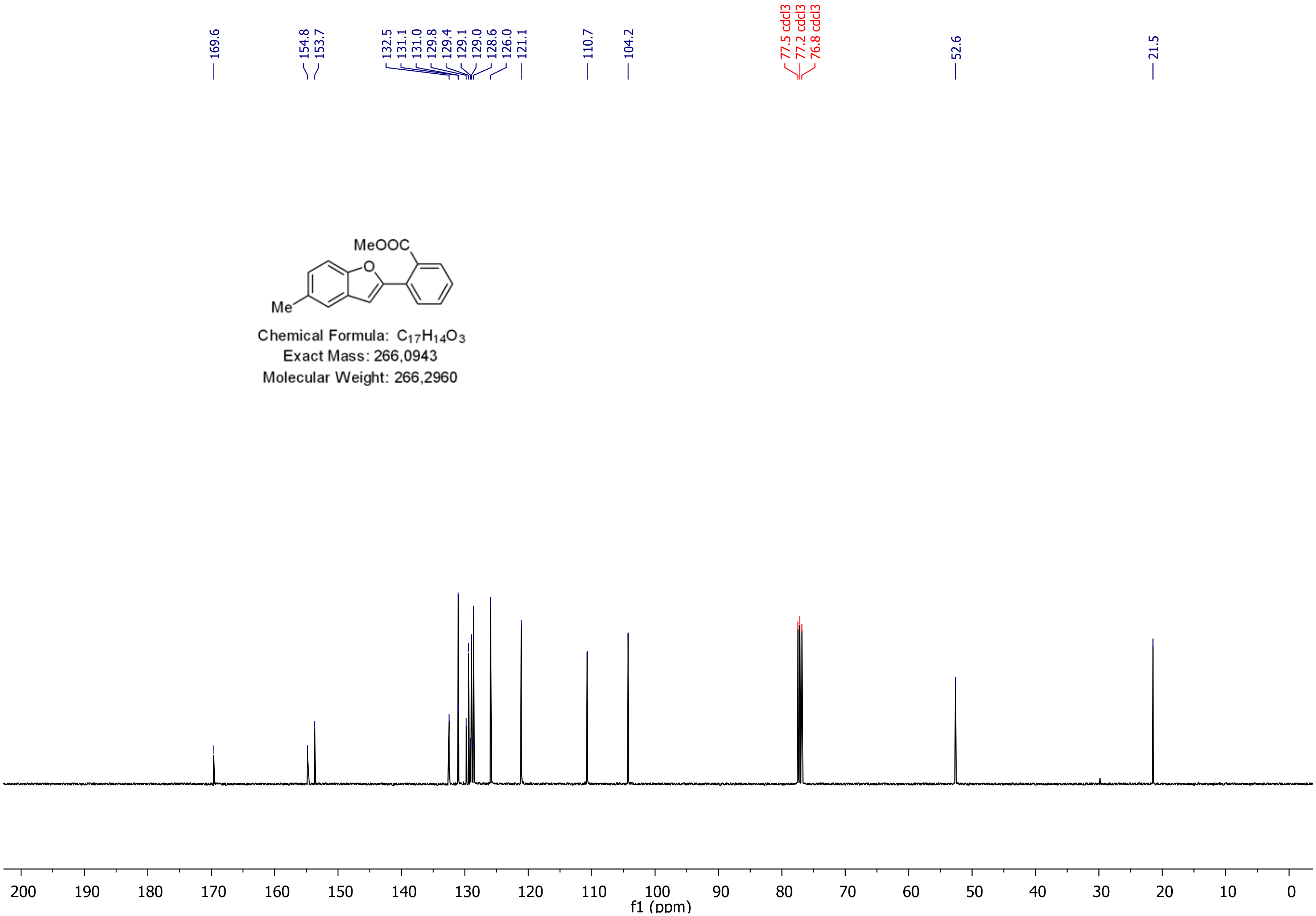
169.6
154.8
153.7
132.5
131.1
131.0
129.8
129.4
129.1
129.0
128.6
126.0
121.1
110.7
104.2
77.5 cdcl3
77.2 cdcl3
76.8 cdcl3
52.6
21.5
MeOOC
O
Me
Chemical Formula: C17H14O3
Exact Mass: 266,0943
Molecular Weight: 266,2960
200 190 180 170 160 150 140 130 120 110 100 90 80 70 60 50 40 30 20 10 0
f1 (ppm)

1,2-dimethyl-3-(phenyldiazenyl)-indole (1bb)

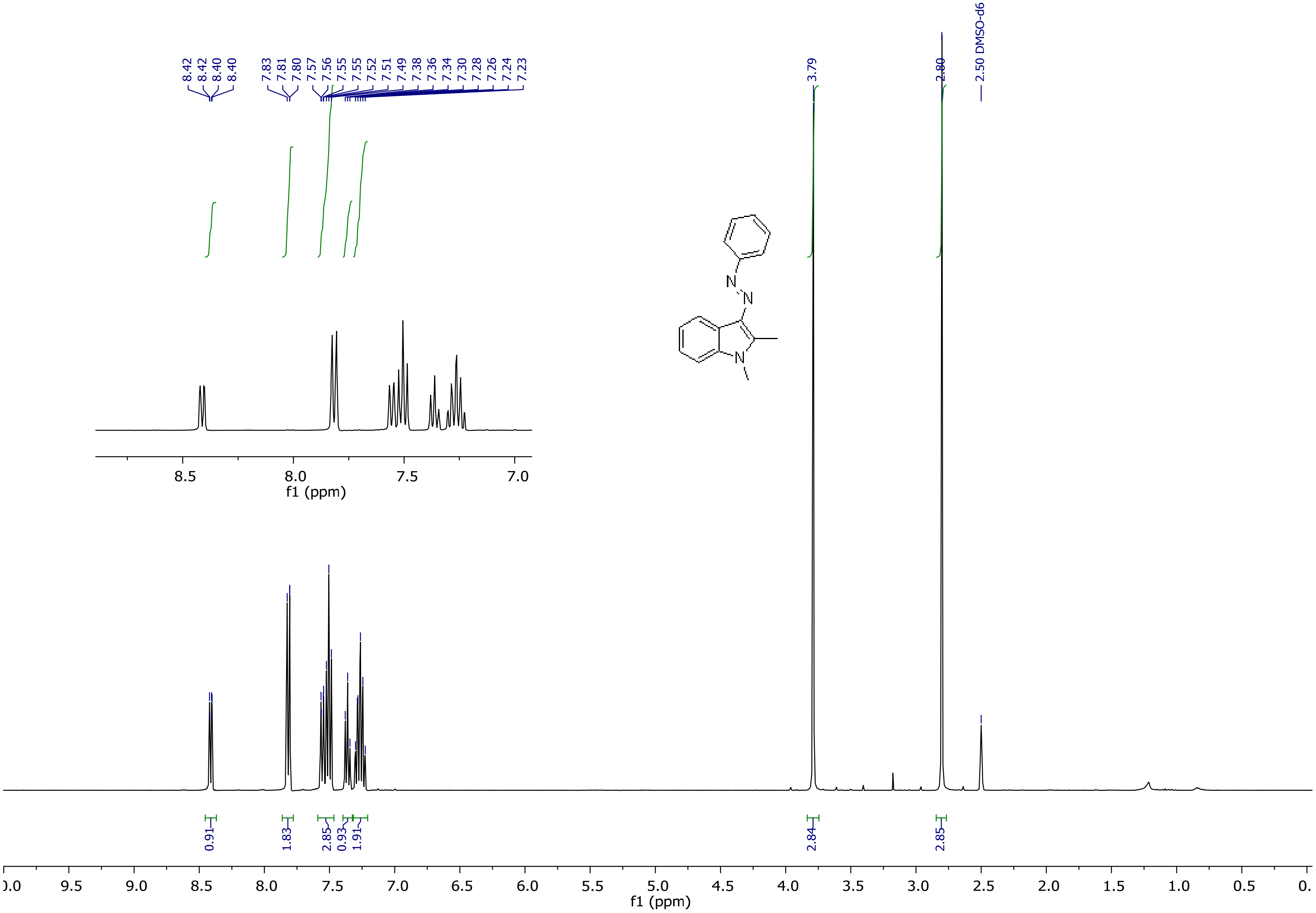

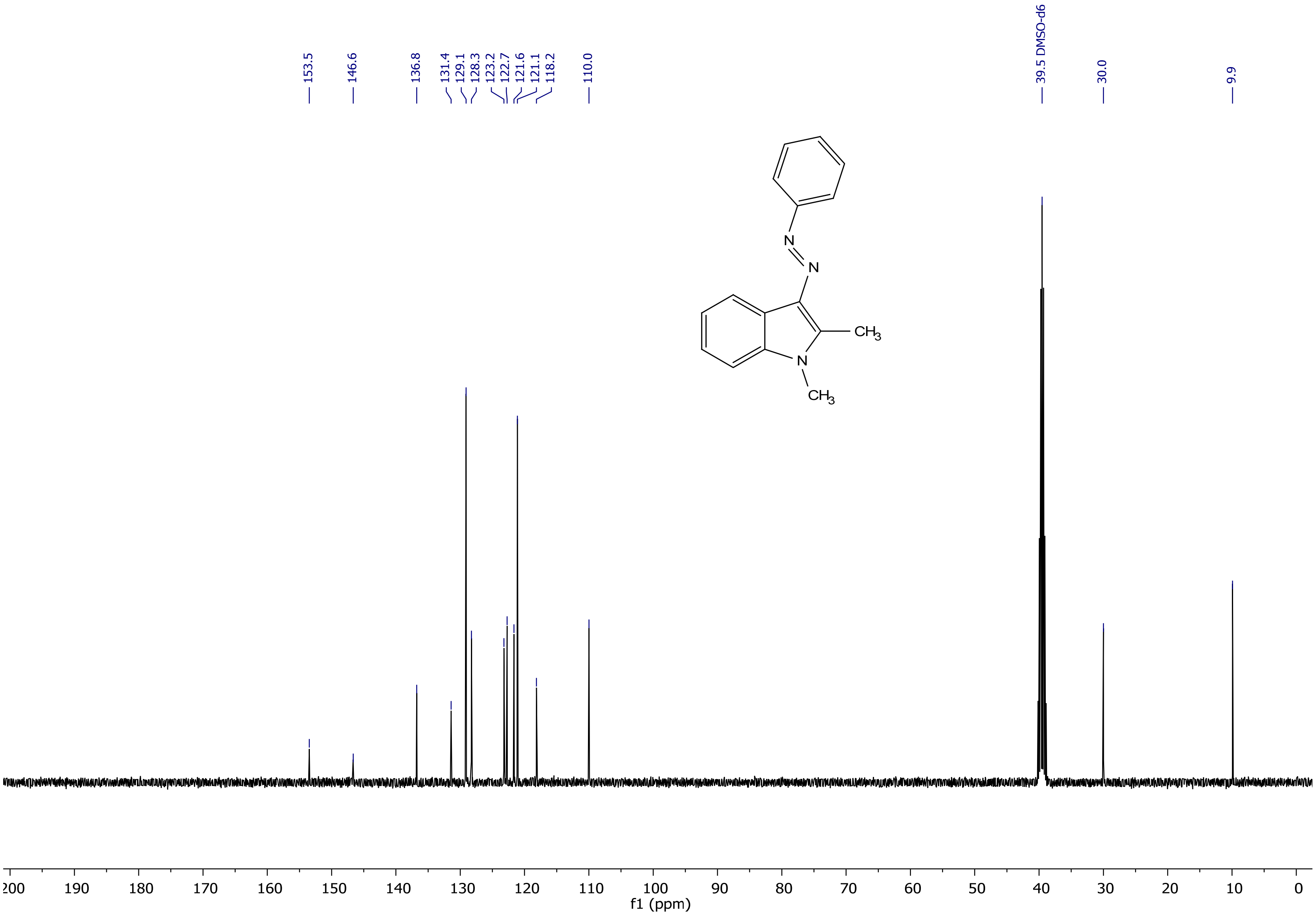

153.5
146.6
136.8
131.4
129.1
128.3
123.2
122.7
121.6
121.1
118.2
110.0
39.5 DMSO-d6
30.0
9.9
N
N
CH3
N
CH3
200
190
180
170
160
150
140
130
120
110
100
90
80
70
60
50
40
30
20
10
0
f1 (ppm)
